# CRBN is downregulated in lung cancer and negatively regulates TLR2, 4 and 7 stimulation in lung cancer cells

**DOI:** 10.1002/ctm2.1050

**Published:** 2022-09-27

**Authors:** Mi‐Jeong Kim, Ji Su Lee, Ji Young Kim, Bongkum Choi, Juhee Son, Yoon Min, Soo‐Kyung Jeong, Duk‐Hwan Kim, Joo Sang Lee, Eunyoung Chun, Ki‐Young Lee

**Affiliations:** ^1^ Department of Immunology and Samsung Biomedical Research Institute Sungkyunkwan University School of Medicine Suwon Republic of Korea; ^2^ Department of Medicine Sungkyunkwan University School of Medicine Suwon Republic of Korea; ^3^ R&D Center CHA Vaccine Institute Seongnam‐si Republic of Korea; ^4^ Department of Molecular Cell Biology Sungkyunkwan University School of Medicine Suwon Republic of Korea; ^5^ Department of Precision Medicine Sungkyunkwan University School of Medicine Suwon Republic of Korea; ^6^ Department of Health Sciences and Technology, Samsung Advanced Institute for Health Sciences & Technology, Samsung Medical Center Sungkyunkwan University Seoul Republic of Korea; ^7^ Single Cell Network Research Center Sungkyunkwan University School of Medicine Suwon Republic of Korea

Dear Editor,

Cereblon (CRBN) has been identified as a primary target of immunomodulatory drugs in multiple myeloma.^1^ Herein, for the first time, we demonstrate that CRBN expression is functionally involved in lung cancer progression through the regulation of autophagy by toll‐like receptor (TLR)2, TLR4 and TLR7. TLR signalling is associated with the induction of autophagy and plays a pivotal role in the progression and pathogenesis of lung cancer.^2^ Our study suggests that CRBN can be a potent prognostic marker for lung cancer and provides important implications in clinical and translational lung cancer biology.

To get insight into the association of CRBN with lung cancer, we utilized data from the TCGA (The Cancer Genome Atlas) (GEPIA, http://gepia.cancer‐pku.cn/detail.php?gene = CRBN) and a cohort of NSCLC patients (*n* = 18). GEPIA database revealed that CRBN was downregulated in lung adenocarcinoma and lung squamous cell carcinoma (Figures S1 and 1A, LUSC, *p < .05). Moreover, CRBN was downregulated in lung tumour tissues (LTTs) of 18 NSCLC patients compared to matched lung normal tissues (LNTs) (Figure 1B and, Table S1). To see whether CRBN expression is associated with a defined set of genes related to cancer in LTTs of NSCLC patients, we selected the top seven LTTs (<−1.5‐fold decrease of CRBN) (Figure 1B, red bars) and performed gene set enrichment analysis (GSEA). The 15 enrichment gene set plot related to oncogenic signature and cancer was enriched in the top 7 LTTs as compared to matched LNTs of NSCLC patients (Figure S2). To decipher the gene association between CRBN expression and lung cancer progression in the 7 LTTs, we aligned 500 downregulated or upregulated genes based on the data of LTT36 with the most downregulation (Figure S3A and Table S2, downregulated genes; Figure S4A and Table S3, upregulated genes). We further sorted downregulated or upregulated genes in all seven LTTs (Figures S3B–D and S4B,C). Then, we accessed PubMed to confirm whether these genes have been reported in lung cancer proliferation, migration or invasion and progression. Among downregulated genes (Figure S3B–D), 17, 11 and 21 genes have been involved in anti‐lung cancer proliferation (Figure 1C, Table S4), anti‐lung cancer migration or invasion (Figure 1D, Table S5) and anti‐lung cancer progression (Figure 1E, Table S6), respectively. Among upregulated genes (Figures 1F and S4B,C), 13, 10 and 4 genes have been involved in lung cancer proliferation (Figure 1G, Table S7), migration or invasion (Figure 1H, Table S8) and progression (Figure 1I, Table S9), respectively. We observed a similar pattern in the other 11 LTTs (Figures S5A–C and S5D–F). To examine the role of CRBN in lung cancer cells, we generated *CRBN*‐knockout (*CRBN*KO) A549 (Figure 1J) and H1299 lung cancer cells (Figure S6) and performed in vitro and vivo cancer progression assays. *CRBN*KO A549 cells significantly enhanced cancer migration (Figure 1K), invasion (Figure 1L), single‐cell migration (Figure 1 M), anchorage‐dependent (Figure 1N) and anchorage‐independent colony formation (Figure 1O). We also observed a similar pattern in *CRBN*KO H1299 cells (Figure S7A–D). Notably, as an in vivo model, NOD/SCID/IL2rγ^null^ (NSG) mice engrafted with *CRBN*KO A549 cells markedly increased the tumour sizes and masses (Figure 1P,Q, *CRBN*KO A549 vs. Ctrl A549). H&E staining data revealed that the number of neoplastic epithelial cells was higher in tumour and metastasized LTTs derived from mice injected with *CRBN*KO A549 (Figure S8A,B, tumour; Figure S8C,D, metastasized lung tumour) compared to those of Ctrl A549, demonstrating that (i) CRBN downregulation is associated with lung cancer; (ii) *CRBN*‐deficiency promotes in vitro and vivo lung cancer progression.

**FIGURE 1:**
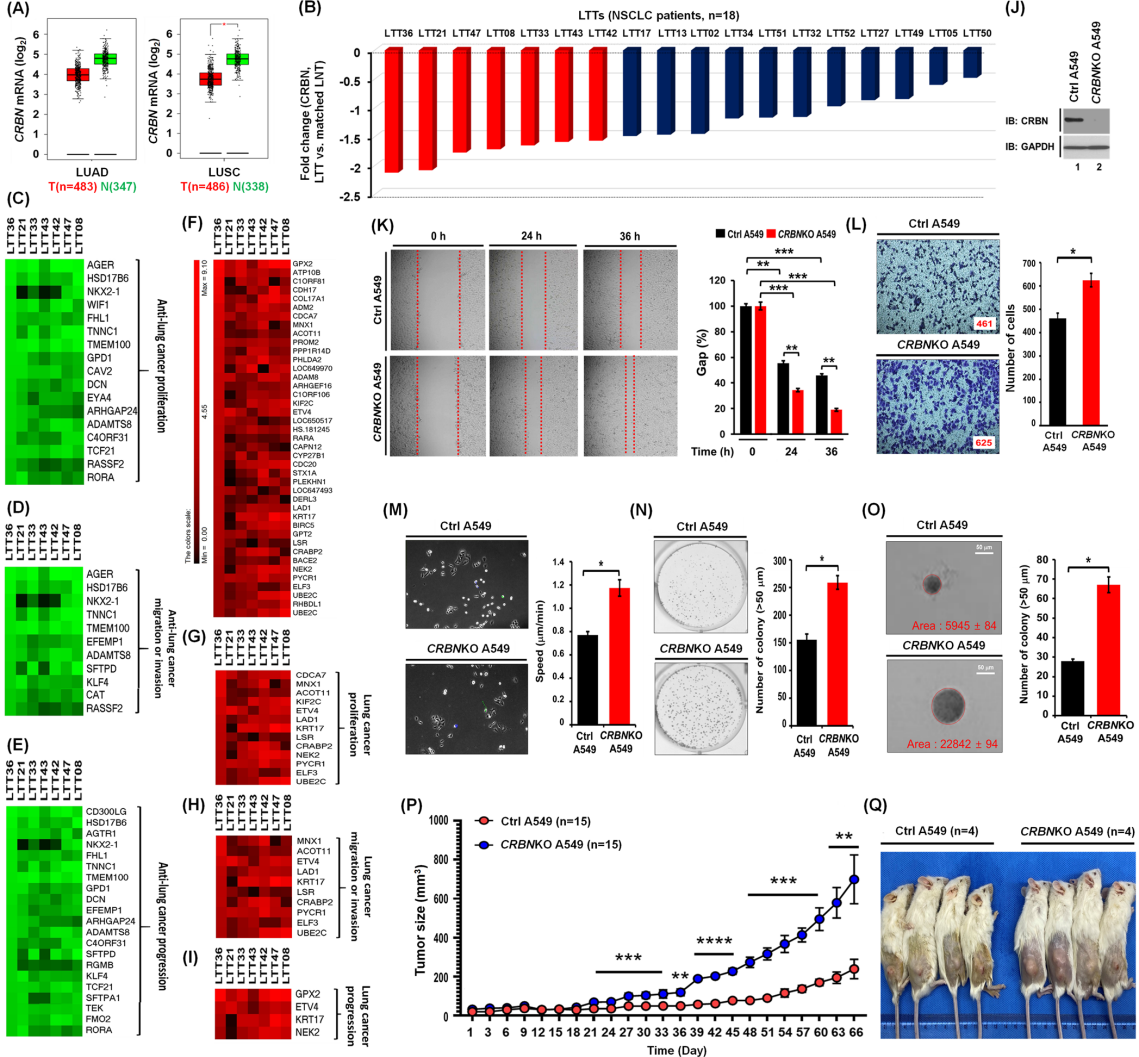
Cereblon (CRBN) expression is downregulated and associated with lung cancer progression: (A) The relative CRBN mRNA levels in non‐tumour and lung adenocarcinoma (LUAD) or LUSC tumour tissues were presented as scatter plots. The median expression level in each group is indicated by horizontal lines, and the values were shown (available at http://gepia.cancer‐pku.cn/detail.php?gene = CRBN). *p < .05; (B) microarray analysis was performed in 18 primary patients with NSCLC. CRBN expression was presented as a fold change in lung tissue tumour (LTT) versus matched lung normal tissue (LNT); (C)–(E) in downregulated genes in all seven LTTs, genes related to anti‐proliferation (C, *n* = 17), anti‐migration or ‐invasion (D, *n* = 11) and anti‐progression (E, *n* = 21) in lung cancer were represented; (F)–(I) upregulated genes in all seven LTTs were listed (F), and genes related to proliferation (G, *n* = 13), migration or invasion (H, *n* = 10) and progression (I, *n* = 4) in lung cancer were represented; (J) using the CRISPR/Cas9 gene‐editing method, *CRBN*‐knockout (*CRBN*KO) and control (Ctrl) A549 lung cancer cells were generated as described in the Supporting Information section; (K)–(O) cancer migration (K), invasion (L), single‐cell mobility (M) and anchorage‐dependent (N) and anchorage‐independent (O) colony formation assay was performed with *CRBN*KO A549 and Ctrl A549 cells, as described in the Supporting Information section. Data are mean ± SD (*n* = 3 plates, migration and invasion assay). The number of colonies was measured using Adobe Photoshop software (±SD, *n* = 3 plates). The size of the colony spheres was measured by ImageJ (±SD, *n* = 15 images). Data analysis of single‐cell mobility was performed following protocols provided by Bio‐protocol (www.bio‐protocol.org/e3586) and (P and Q) NSG mice were injected with Ctrl A549 (red circles, *n* = 15) or *CRBN*KO A549 (blue circles, *n* = 15) lung cancer cells, as described in the Supporting Information section. Tumour size was measured from xenografted NSG mice 66 days post‐injection, as indicated (P). On post‐injection day 66, NSG mice with Ctrl A549 (*n* = 4) and *CRBN*KO A549 (*n* = 4) xenograft cells were euthanized and represented (Q). *p < .05, **p < .01, ***p < .001, ****p < .0001

Growing evidence suggests that intrinsic and extrinsic factors, including spontaneous mutations of genes and TLRs, play pivotal roles in lung cancer development and progression.^2^, ^3^, ^4^, ^5^, ^6^, ^7^ Importantly, TLR2, TLR4 and TLR7 are expressed in lung cancer and associated with lung cancer progression.^5^, ^6^, ^7^ Additionally, TLR4‐ and TLR3‐ induced autophagy facilitates migration and invasion of lung cancer cells.^2^ We previously reported that CRBN negatively regulates TLR4 signalling through the attenuation of ubiquitination of TRAF6, and autophagy activation by inhibiting the ubiquitination of BECN1.^8^, ^9^ Therefore, we sought to determine whether CRBN is involved in lung cancer progression by TLR2, TLR4 and TLR7 signals associated with autophagy. We first examined if TLR2, TLR4 and TLR7 signals are associated with genes related to lung cancer progression and autophagy. We treated A549 and H1299 cells with vehicle as a control, TLR2 (heat‐killed *Listeria monocytogenes*, HKLM), TLR4 (lipopolysaccharide, LPS) or TLR7 (imiquimod, IQM) agonists and performed RNA‐sequencing analysis. The GSEA of transcriptional profiles revealed that 15 enrichment gene set plots related to cancer gene neighbourhoods and cancer modules, ontology and oncogenic signature were enriched in A549 and H1299 treated with TLR4 agonist compared to those with the vehicle (Figure S9A–C, A549; Figure S9D–F, H1299). To examine the association of genes related to lung cancer progression and autophagy in response to TLR stimulation, we arranged 500 upregulated genes (Figure S10A and Table S10 in A549; Figure S10B, Table S11 in H1299) or 500 downregulated genes (Figure S10C and Table S12 in A549; Figure S10D and Table S13 in H1299) based on the data of TLR4 stimulation. Then, we further sorted upregulated (Figure S11A and Table S14 in A549; Figure S11B and Table S15 in H1299) or downregulated (Figure S11C and Table S16 in A549; Figure S11D and Table S17 in H1299) genes in all three TLR stimulations. Among upregulated genes, 12 genes (Figure 2A and Table S18) in A549 cells and 21 genes (Figure 2B and Table S19) in H1299 cells have been involved in lung cancer progression. In the list of downregulated genes, 10 genes (Figure 2C and Table S20) in A549 cells and 11 genes (Figure 2D and Table S21) in H1299 cells have been involved in anti‐lung cancer progression. Notably, 13 genes upregulated in A549 or H1299 cells have been functionally associated with autophagy (Figure 2E, Table S22). Moreover, enrichment gene sets regulating autophagy, such as BECN1 (Figure S12A), MTOR (Figure S12B) and AKT‐MTOR (Figure S12C) were enriched in A549 treated with TLR4 agonist compared to those with the vehicle. These results suggest that TLR2, TLR4 and TLR7 stimulation increase gene signatures related to lung cancer progression and autophagy (Figure 2F).

**FIGURE 2:**
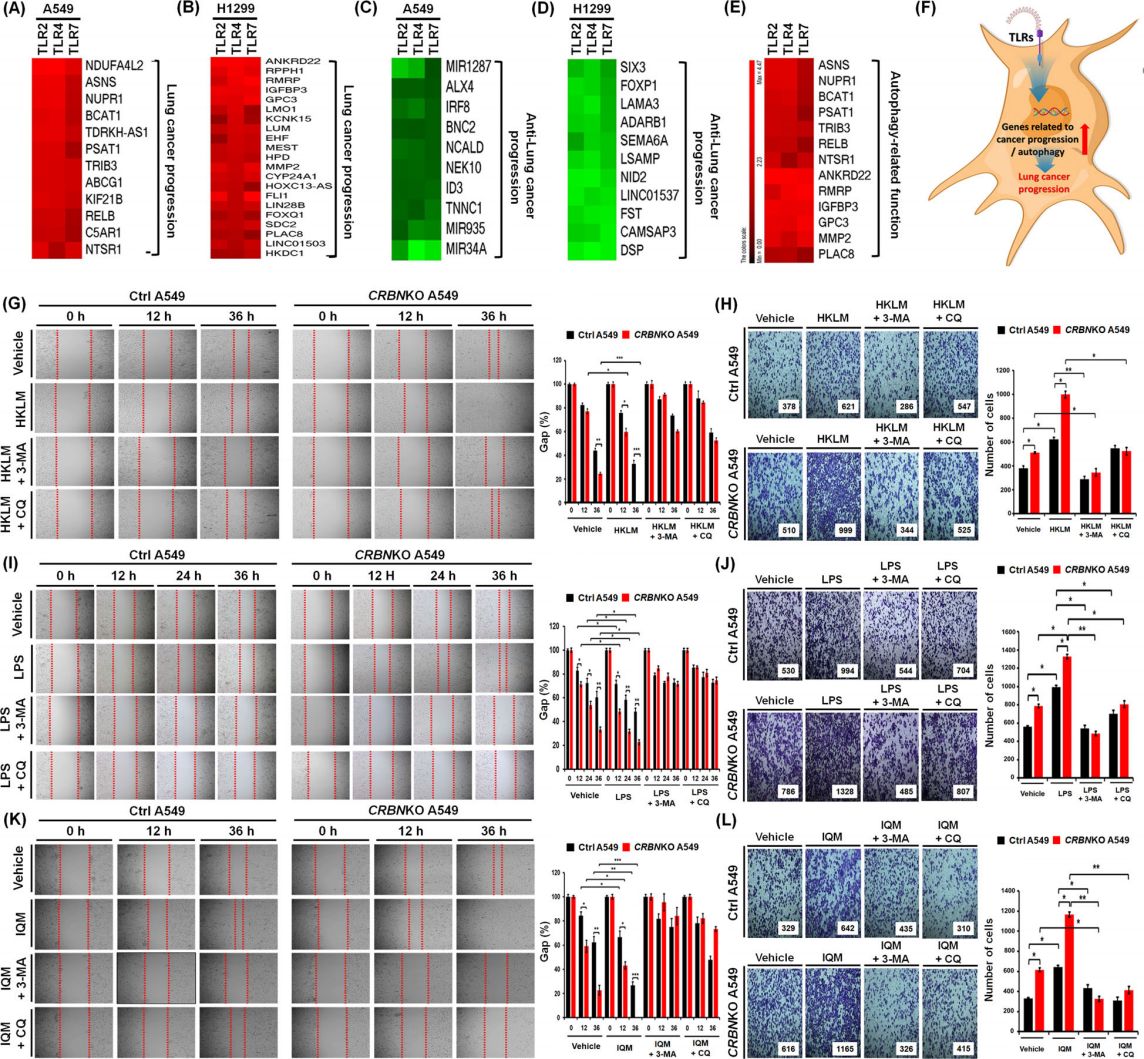
Toll‐like receptor (TLR)2, TLR4 and TLR7 are associated with genes related to cancer progression and autophagy and enhance migration and invasion in cereblon‐knockout (*CRBN*KO) A549 lung cancer cells. A549 or H1299 lung cancer cells were treated with vehicle (DMSO, .1% v/v concentration), heat‐killed *Listeria monocytogenes* (HKLM) (10^8^/ml), lipopolysaccharide (LPS) (10 μg/ml), imiquimod (IQM) (10 μg/ml) and RNA‐sequencing (RNA‐seq) analysis was performed, as described in the Supporting Information section: (A and B) in upregulated genes in A549 or H1299 cells by TLR2, TLR4 and TLR7, genes related to cancer progression were sorted and represented (A, A549 cells; B, H1299 cells); (C and D) in downregulated genes in A549 or H1299 cells by TLR2, TLR4 and TLR7, genes related to anti‐cancer progression were sorted and represented (C, A549 cells; D, H1299 cells); (E) in upregulated genes in A549 (A) and H1299 (B) by TLR2, TLR4 and TLR7, genes regulating autophagy‐related function were represented; (F) a schematic view of the association of TLR2, TLR4 and TLR7 with genes related to lung cancer progression and autophagy and (G)–(L) control (Ctrl) and *CRBN*KO A549 cells were prepared, treated with vehicle (DMSO, .1% v/v concentration), HKLM (10^8^/ml), LPS (10 μg/ml) or IQM (10 μg/ml), and cancer migration and invasion assays were performed, as described in the Supporting Information section (G and H, HKLM; I and J, LPS; K and L, IQM). The residual gap between the migrating cells from the opposing wound edge is expressed as a percentage of the initial scraped area (±SD, *n* = 3 different plates). The migrating cells were counted. The results are presented as the mean ± SD of three independent experiments. *p < .05, **p < .01, and ***p < .001

We next examined whether CRBN is functionally involved in lung cancer progression through the regulation of autophagy by TLR stimulation. Under the TLR2 (HKLM), TLR4 (LPS) or TLR7 (IQM) stimulation, cell migration and invasion were significantly enhanced in *CRBN*KO A549 cells (Figure 2G,H, HKLM; Figure 2I,J, LPS; Figure 2K,L, IQM: *CRBN*KO A549 vs. Ctrl A549). Marked inhibition was observed in the presence of autophagy inhibitors, 3‐MA or CQ (Figure 2G–L, HKLM, LPS or IQM agonist + 3‐MA or CQ vs. TLR agonist alone). Importantly, LC3‐II levels and LC3 puncta that represent induction of autophagy were significantly enhanced in *CRBN*KO A549 treated with TLR agonists (Figure 3A–C, LC3‐II levels; Figure 3D,E, LC3 puncta). TLR4 signalling induces autophagy through the BECN1 ubiquitination by TRAF6.^2^, ^8^, ^9^, ^10^ Then, we examined whether *CRBN*‐deficiency directly affects the BECN1 ubiquitination by TLR2, TLR4 and TLR7 stimulation. BECN1 ubiquitination was markedly elevated in *CRBN*KO A549 cells treated with HKLM, LPS or IQM as compared to those of Ctrl A549 cells (Figure 3F, lane 6 vs. lane 2 in HKLM; lane 7 vs. lane 3 in LPS; lane 8 vs. lane 4 in IQM). IL‐6, CCL2, CCL20 and MMP2 productions are necessary for enhanced migration and invasion of lung cancer cells upon TLR activation.^2^ We found that *CRBN*KO A549 cells markedly increased IL‐6, CCL2, CCL20 and MMP2 in response to three TLR agonists compared to those of Ctrl A549 (Figure 3G, IL‐6; Figure 3H, CCL2; Figure 3I, CCL20; Figure 3J, MMP2: *CRBN*KO A549 vs. Ctrl A549), whereas marked inhibitions were observed in the co‐treatment of autophagy inhibitors (Figure 3G–J, 3‐MA). Lastly, we found that *CRBN*KO A549 cells treated with TLR agonists significantly increased single‐cell mobility and the number of colonies compared to those of Ctrl A549 cells (Figure 4A,B, single‐cell mobility; Figure 4C,D, number of colonies).

**FIGURE 3:**
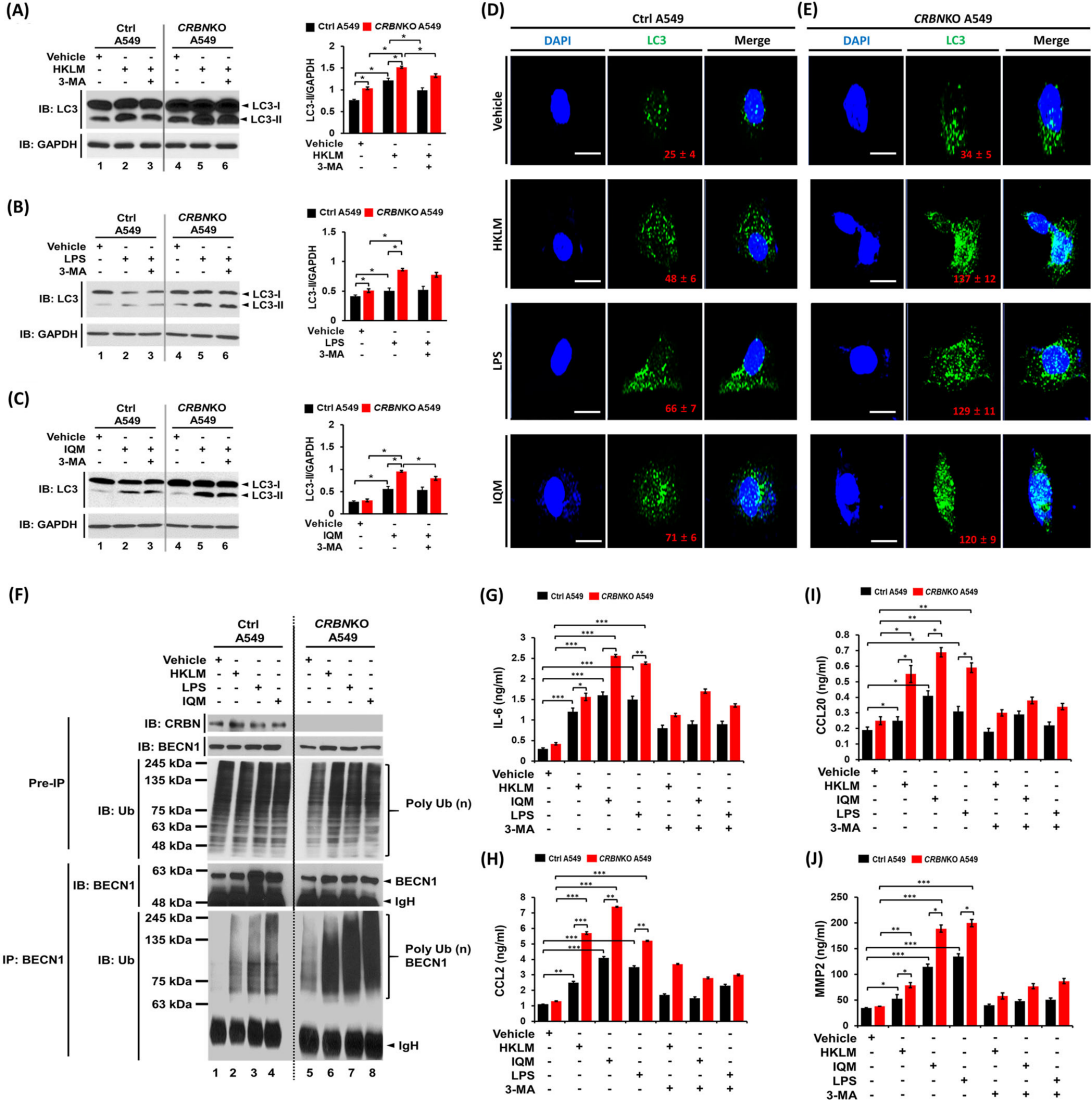
Toll‐like receptor (TLR)2, TLR4 and TLR7 enhance autophagy and cancer‐promoting cytokines in cereblon‐knockout (*CRBN*KO) A549 lung cancer cells: (A)–(C) Ctrl A549 and *CRBN*KO A549 cells were treated with vehicle (DMSO, .1% v/v concentration), heat‐killed *Listeria monocytogenes* (HKLM) (10^8^/ml) as a TLR2 agonist (A), lipopolysaccharide (LPS) (10 μg/ml) as a TLR4 agonist (B) or imiquimod (IQM) (10 μg/ml) as a TLR7 agonist (C) in the presence or absence of 3‐MA (5 mM). Cell lysates were immunoblotted with anti‐LC3A/B antibody. Anti‐GAPDH was used as a loading control. Band intensity was quantified using ImageJ software (±SD, *n* = 3). *p < .05, **p < .01; (D and E) ctrl A549 or *CRBN*KO A549 cells were grown on glass coverslips, treated with vehicle (DMSO, .1% v/v concentration), HKLM (1.5 × 10^8^/ml), LPS (15 μg/ml) or IQM (10 μg/ml) for 6 h and then fixed. Immunofluorescence assay was performed with anti‐LC3 antibody, as described in Supporting Information section. Digital images were captured with confocal microscopy, and the number of LC3‐puncta was scored. Quantification represents the mean ± SD of puncta per cell (*n* = 15) from three independent experiments. Scale bar: 10 μm; (F) ctrl A549 and *CRBN*KO A549 cells were stimulated with vehicle, HKLM, LPS or IQM for 60 min, lysed, and then immunoprecipitated with anti‐BECN1 antibody and probed with anti‐cereblon (CRBN), anti‐BECN1 and anti‐Ub antibodies and (G)–(J) Ctrl A549 or *CRBN*KO A549 cells were treated with vehicle, HKLM, LPS or IQM in the presence or absence of 3‐MA (5 mM) for 24 h, and the levels of IL‐6 (G), CCL2 (H), CCL20 (I) and MMP2 (J) were measured by ELISA (±SD, *n* = 3) as described in Materials and Methods. *p < .05, **p < .01 and ***p < .001

**FIGURE 4:**
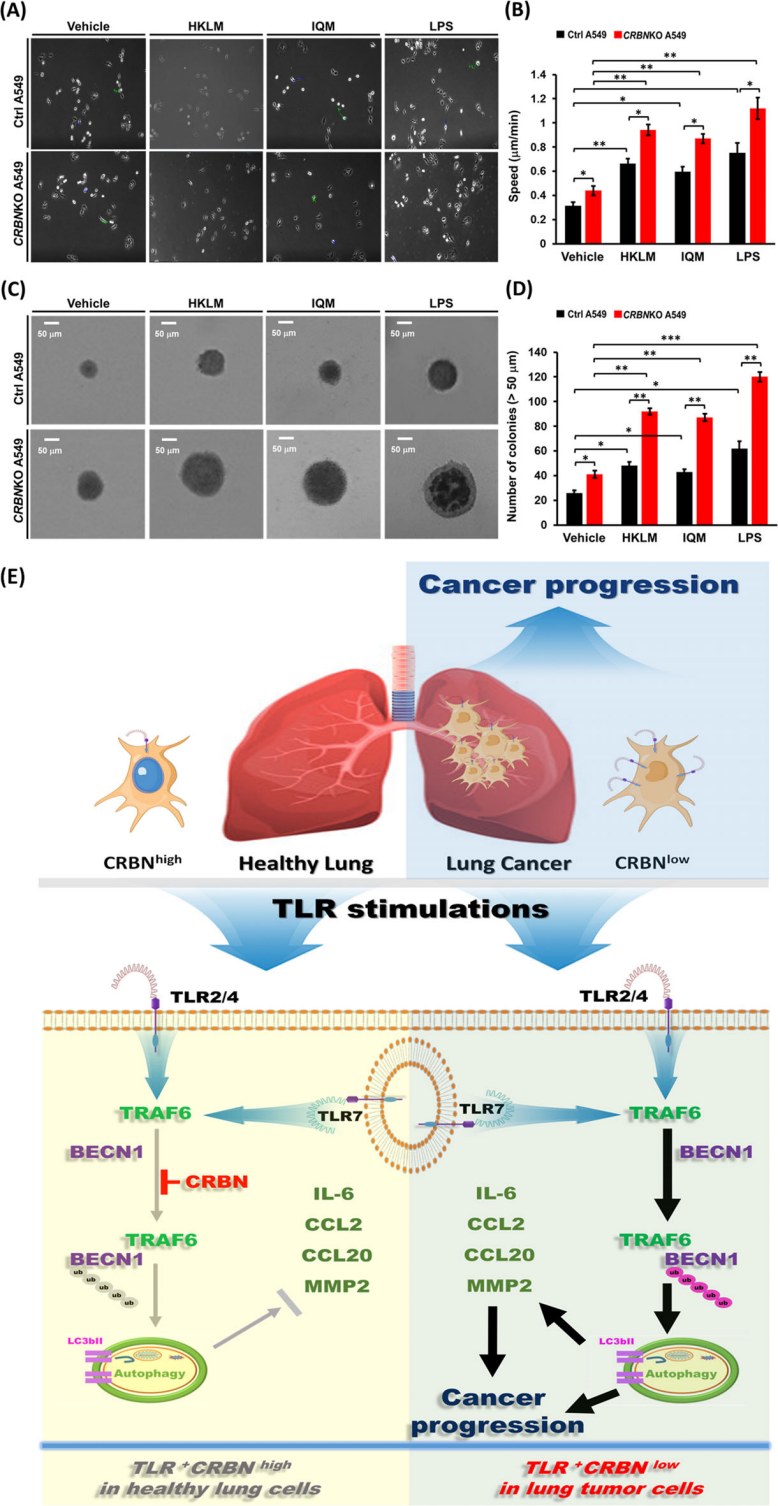
Toll‐like receptor (TLR)2, TLR4 and TLR7 enhance single‐cell migration and colony‐forming activity in cereblon‐knockout (*CRBN*KO) A549 lung cancer cells: (A and B) Ctrl A549 and *CRBN*KO A549 cells were treated with vehicle (DMSO, .1% v/v concentration), heat‐killed *Listeria monocytogenes* (HKLM) (10^8^/ml), imiquimod (IQM) (10 μg/ml) or lipopolysaccharide (LPS) (10 μg/ml), and time‐lapse imaging analysis was performed as described in Materials and Methods (A). Data analysis of single‐cell migration/mobility was performed following protocols provided by Bio‐protocol (www.bio‐protocol.org/e3586). Mean ± SD from three independent experiments (*n* = 12) (B). *p < .05, **p < .01 and ***p < .001; (C and D) ctrl A549 and *CRBN*KO A549 cells were treated with vehicle, HKLM (10^8^/ml), IQM (10 μg/ml) or LPS (10 μg/ml), and an anchorage‐independent colony‐forming assay was performed as described in Materials and Methods (C). The size of the colony spheres was measured by ImageJ (D, ±SD, *n* = 15 images) *p < .05, **p < .01 and ***p < .001; (E) schematic illustration of how cereblon (CRBN) regulates lung cancer progression. CRBN expression is distinctly different; relatively higher in healthy lung cells (*CRBN* ^high^) than in lung cancer (*CRBN* ^low^) (upper). In healthy lung cells, CRBN inhibits TRAF6‐induced ubiquitination of BECN1 for autophagy induction and attenuates the production of IL‐6, CCL2, CCL20 and MMP2 cytokines in response to TLR stimulations (down left). In lung cancer cells, CRBN expression is downregulated, thereby increasing the TRAF6‐induced ubiquitination of BECN1 for autophagy induction and the production of IL‐6, CCL2, CCL20 and MMP2 cytokines in response to TLR stimulations (down, right). Eventually, autophagy and these cytokines facilitate TLR2‐, TLR4‐ and TLR7‐induced lung cancer progression

In summary, CRBN is downregulated in lung cancer cells and associated with lung cancer progression (Figure 4E, upper right). Our study demonstrates the association between TLR stimulation and gene signatures related to lung cancer progression and autophagy. CRBN inhibits the BECN1 ubiquitination to induce autophagy and attenuates the production of IL‐6, CCL2, CCL20 and MMP2 cytokines in response to TLR stimulations in healthy lung cells expressing CRBN (*CRBN* ^high^, Figure 4E, down left). In lung cancer cells with downregulated CRBN (*CRBN* ^low^, Figure 4E, down right), engagements of TLRs enhance autophagy induction through the increases of BECN1 ubiquitination and the production of IL‐6, CCL2, CCL20 and MMP2 cytokines, eventually facilitating lung cancer progression. Taken together, our clinically comparative results and functional investigations of CRBN in lung cancer progression will potentially contribute to translational approaches for lung cancer intervention.

## CONFLICT OF INTEREST

The authors declare that they have no conflict of interest.

## CONSENT FOR PUBLICATION

All authors agree to publish this article.

## Supporting information

Supporting Information
