# Supplementary material for: CRBN is downregulated in lung cancer and negatively regulates TLR2, 4 and 7 stimulation in lung cancer cells
Source: Clin Transl Med. 2022 Sep 27;12(9):e1050. doi: 10.1002/ctm2.1050 (PMC9513676; doi:10.1002/ctm2.1050)
Supplement: Supplementary file 1 — Supporting Information [file CTM2-12-e1050-s001.pdf]

Supplementary Information

CRBN is downregulated in lung cancer and negatively regulates TLR2, 4, and 7 stimulation in lung cancer cells

Methods and Materials

NSCLC patient samples

NSCLC tissues and matched adjacent normal tissues were collected from the Samsung Medical Center (SMC), with informed consent from 18 patients with NSCLC in accordance with the ethical principles stated in the Declaration of Helsinki. This study was approved by the Institutional Review Board (IRB) of Samsung Medical Center (SMC) (IRB#: 2010-07-204), following the procedures previously described.[1-3]

Animal experiments

NOD/SCID/IL-2Rγnull (NSG) mice were purchased from the Jackson Laboratory (Bar Harbor, ME, USA) and maintained under specific pathogen-free conditions at the Laboratory Animal Research Center (LARC) of the Samsung Medical Center (SMC), Seoul, South Korea in accordance with the ethical guidelines for the care of these mice. All experimental procedures were approved by the Institutional Animal Care and Use Committee (IACUC) of the SMC (No. 20160617001). *CRBN*-knockout (*CRBN*KO) (5×10^6 cells per mouse, $n = 15$) or control (Ctrl) A549 cells (5×10^6 cells per mouse, $n = 15$) were resuspended in serum-free RPMI and injected under the skin of NSG mice (back area), and tumor volume was measured, following procedures previously described.[4]

Lung cancer cell lines

A549 cells (human lung cancer cell line; ATCC, CCL-185) and H1299 cells (human non-small cell lung cancer cell line; ATCC, CRL-5803) were maintained in RPMI 1640 medium (Sigma Aldrich, 31800-022) supplemented with 10% fetal bovine serum (FBS), penicillin (100 μg/mL), and streptomycin (100 μg/mL) in a 5% CO_2 humidified atmosphere at 37 °C.

Antibodies and reagents

Anti-CRBN (Ca# ab68763) antibody was purchased from Abcam (Cambridge, MA, USA). Lipopolysaccharide (LPS; serotype 0128: B12), chloroquine (CQ; C6628), dimethyl sulfoxide

(DMSO; 472301), puromycin (P8833), paraformaldehyde (P6148), Triton X-100 (T8787), 3-methyladenine (3-MA; M9281), gentamicin (G1272), deoxycholate (D6750), and Dulbecco's phosphate-buffered saline (DPBS; D8537) were purchased from Sigma-Aldrich (St Louis, MO, USA). Heat-killed Listeria monocytogenes (HKLM) (Cat. Code tlrl-hklm), and Imiquimod (R837) (Cat. Code tlrl-imqs) were purchased from InvivoGen (San Diego, CA, USA). Lipofectamine 2000 (11668019) was purchased from Thermo Fisher Scientific (Waltham, MA, USA).

Generation of CRBN-knockout (CRBNKO) lung cancer cell by CRISPR/Cas9

Guide RNA sequences for *CRBN* were designed as previously described.[5] Briefly, human CRBN gRNA sequences, 5′-CACCGATATGCCTATCGAGAAGAAC-3′/5′-AAACGTTCTTCTCGATAGGCATATC-3′, targeted to exon 2 of the CRBN gene were cloned into a Lenti CRISPR v2 vector (Addgene plasmid, Ca#52961). A549 and H1299 cells were transfected with Lenti CRISPR v2/gRNA using Lipofectamine 2000 according to the manufacturer's instructions. To generate control (Ctrl) A549 or H1299 cells, cells were transfected with Lenti CRISPR Universal Non-Target Control #2 Plasmid DNA (LV04 vector) (Sigma-Aldrich, CRISPR19-1EA). Two days after transfection, the cells were treated with 2 μg/mL of puromycin for 3 days. After 2 weeks, colonies were isolated in the 96-well plate, and the expression levels of CRBN were analyzed.

Western blotting and ubiquitination assay

Ctrl A549 and *CRBN*KO A549 cells were treated with vehicle (DMSO, 0.1% v/v concentration), HKLM (10^8/ml), LPS (10 μg/ml), or IQM (10 μg/ml) in the presence or absence of 3-MA (5 mM) for 6 h. Cell lysates were immunoblotted with anti-LC3A/B antibody. Anti-GAPDH was used as a loading control. For ubiquitination assay, Ctrl A549 and *CRBN*KO A549 cells were treated with vehicle (DMSO, 0.1% v/v concentration), HKLM (10^8/ml), LPS (10 μg/ml), or IQM (10 μg/ml) for 60 min, lysed in lysis buffer containing 150 mM NaCl, 20 mM Tris-HCl, pH 7.5, 10 mM EDTA, 1% Triton X-100, 1% deoxycholate, 1.5% aprotinin, and 1 mM phenylmethylsulfonyl fluoride, and immunoprecipitated with anti-BECN1 antibody. Immunoprecipitated complexes were separated by SDS–PAGE and probed with anti-CRBN, anti-BECN1, and anti-Ub antibodies.

Wound-healing migration assay

A wound-healing migration assay was performed following previous protocols.[1,2,4,6] Control (Ctrl) A549 and *CRBN*KO A549 cells were prepared and treated with vehicle (DMSO, 0.1% v/v concentration), HKLM (10^8/mL), LPS (10 μg/mL), or IQM (10 μg/mL) in the presence or absence of 3-MA (5 mM), or CQ (10 μM). Cell images were captured after culturing for different periods, as indicated in each experiment. Ctrl H1299 and *CRBN*KO H1299 cells were

seeded into 12-well plates and cultured to reach about 90 % confluence. Cell monolayers were gently scratched by the tip and washed with a culture medium. After removing floating cells and debris, cells were incubated for different times as indicated. Cell images were captured after the culture for different periods, as indicated in each experiment.

Transwell invasion assay

The transwell invasion assay was performed following previous protocols.[1,2,4-7] Control (Ctrl) A549 and *CRBN*KO A549 cells were prepared and treated with vehicle (DMSO, 0.1% v/v concentration), HKLM (10^8/mL), LPS (10 μg/mL), IQM (10 μg/mL), 3-MA (5 mM), or CQ (10 μM). The invaded cells were stained with 4,6-diamidino-2-phenylindole (Sigma-Aldrich, D9542) and quantified by counting the number of fluorescent cells. Ctrl H1299 and *CRBN*KO H1299 cells were suspended in a culture medium (200 μL) containing FBS. Cells were then added to the upper compartment of a 24-well Transwell® chamber containing a polycarbonate filter with 8-μm pores and coated with 60 mL of Matrigel (Sigma Aldrich, E1270; 1:9 dilution). After incubating for 24 h, cells in the upper compartment were removed, washed with PBS, and fixed. Invaded cells were stained with 4,6-diamidino-2-phenylindole (Sigma-Aldrich, D9542) and quantified by counting the number of fluorescent cells.

Microarray analysis

Microarray analysis was performed as previously described.[6-8] Briefly, total RNA was extracted from the tumor and matched normal tissues of 18 patients with NSCLC using Trizol (Thermo Fisher Scientific, 15596026) and purified using RNeasy columns (74106, Qiagen, Hilden, Germany) according to each manufacturer's protocol.

Transcriptome sequencing analysis

For RNA sequencing (RNA-seq), A549 or H1299 lung cancer cells were treated with or without HKLM (10^8/mL), LPS (10 μg/ml), or IQM (10 μg/mL) for 60 min. Transcriptome sequencing analysis was performed, as previously described.[9-12] Differential gene expression analysis was performed by the PermutMatrix software.[6-8]

LC3 puncta assay

LC3 puncta assay was performed as described in previous reports.[13,14] Briefly, Ctrl A549 or *CRBN*KO A549 cells were grown on glass coverslips, treated with vehicle (DMSO, 0.1% v/v concentration), HKLM (1.5 x 10^8/mL), LPS (15 μg/mL), or IQM (10 μg/mL) for 6 hr, and fixed with 4% paraformaldehyde (Sigma, P-6148), and treated with 0.2% Triton X-100 (Sigma, T9284) to permeabilize for 30 min on ice. Slides were mounted in VECTASHIELD mounting medium (Vector Laboratories, H-1000) and examined under a LSM 710 laser-scanning

confocal microscope (Carl Zeiss,Jena, Germany).

Cytokine measurements

Ctrl A549 or *CRBN*KO A549 cells were treated with HKLM (10^8/mL), LPS (10 μg/mL), or IQM (10 μg/mL) in the presence or absence of 3-MA (5 mM) for 24 h. The levels of IL-6 (D6050, R&D Systems, Minneapolis, MN, USA), CCL2 (DCP00, R&D Systems), CCL20 (DM3A00, R&D Systems), and MMP2 (DMP2F0, R&D Systems) were measured by enzyme-linked immunosorbent assay (ELISA) according to the manufacturer's protocols.

Anchorage-independent soft agar colony formation assay

Anchorage-independent soft agar colony formation assay was performed following previous protocols.[15,16] Briefly, Ctrl A549 and *CRBN*KO A549 cells (1×10^4 cells per well) mixed with 0.3% Difco Noble Agar (BD Biosciences, CA, USA) in a complete medium were plated on the top of the 0.5% agar layer in a 6-well plate with complete medium. Culture medium (1.5 mL) with vehicle (DMSO, 0.1% v/v concentration), HKLM (10^8/mL), LPS (10 μg/mL), or IQM (10 μg/mL) in the presence or absence of 3-MA (5 mM) or CQ (10 μM) was added on top of the layer and the cells were incubated at 37 °C for 4 weeks.

Colony formation assay

The ability of a single cell to grow into a colony was assessed by the colony formation assay as previously described.[15,17] Ctrl A549 and *CRBN*KO A549 cells were harvested with trypsin-EDTA and resuspended as single cells. Cells (1×10^3 per well) were plated in a 6-well plate. After incubation for 10 days, the colonies were stained with 0.5% crystal violet (Sigma) for 30 min at room temperature and the number of colonies was counted under a microscope.

Histological analysis

At the end of the experiment, tumor and lung tissues were isolated from NSG mice xenografted with Ctrl A549 and *CRBN*KO A549 cells and embedded in paraffin. Hematoxylin and eosin (H&E) were performed as previously described.[4]

TCGA data analysis

The expression of *CRBN* in 33-human cancers was analyzed using TCGA data (GEPIA, gene expression profiling interactive analysis; http://gepia.cancer-pku.cn/ and http://gepia.cancer-pku.cn/detail.php?gene=CRBN).

Gene set enrichment analysis (GSEA)

GSEA was performed using the GSEA software (https://www.gsea-msigdb.org/gsea/index.jsp,

Broad Institute, Cambridge, MA, USA) on the normalized gene expression data (FPKMs) from transcriptome sequencing data of A549 and H1299 cells treated with TLR agonists or vehicle, and the microarray data of 7 LTTs and matched LNTs of NSCLC patients.

Statistical analysis

All *in vitro* data are expressed as the mean ± SD of triplicate samples or 10 different cells. Statistical significance was analyzed by ANOVA or the Student's t-test using GraphPad Prism 5.0 (GraphPad Software, San Diego, CA, USA). The values represent the mean ± SD of three independent experiments. The P-values were marked as *$P<0.05$, **$P<0.001$, and ***$P<0.0001$ in all figures.

References

1. Kim MJ, Min Y, Jeong SK, Son J, Kim JY, Lee JS, et al. USP15 negatively regulates lung cancer progression through the TRAF6-BECN1 signaling axis for autophagy induction. *Cell Death Dis.* 2022; **13**(4):348.
2. Kim MJ, Min Y, Son J, Kim JY, Lee JS, Kim DH, et al. AMPKα1 Regulates Lung and Breast Cancer Progression by Regulating TLR4-Mediated TRAF6-BECN1 Signaling Axis. *Cancers (Basel).* 2020; **12**(11):3289.
3. Kim Y, Lee BB, Kim D, Um S, Cho EY, Han J, et al. Clinicopathological Significance of RUNX1 in Non-Small Cell Lung Cancer. *J Clin Med.* 2020; **9**(6):1694.
4. Kim MJ, Choi B, Kim JY, Min Y, Kwon DH, Son J, et al. USP8 regulates liver cancer progression via the inhibition of TRAF6-mediated signal for NF-κB activation and autophagy induction by TLR4. *Transl Oncol.* 2022; **15**(1):101250.
5. Kim MJ, Min Y, Shim JH, Chun E, Lee KY. CRBN Is a Negative Regulator of Bactericidal Activity and Autophagy Activation Through Inhibiting the Ubiquitination of ECSIT and BECN1. *Front Immunol.* 2019; **10**:2203.
6. Min Y, Wi SM, Kang JA, Yang T, Park CS, Park SG, et al. Cereblon negatively regulates TLR4 signaling through the attenuation of ubiquitination of TRAF6. *Cell Death Dis.* 2016; **7**(7):e2313.
7. Kim SY, Jeong S, Jung E, Baik KH, Chang MH, Kim SA, et al. AMP-activated protein kinase-α1 as an activating kinase of TGF-β-activated kinase 1 has a key role in inflammatory signals. *Cell Death Dis.* 2012; **3**(7):e357.
8. Min Y, Wi SM, Shin D, Chun E, Lee KY. Peroxiredoxin-6 Negatively Regulates Bactericidal Activity and NF-κB Activity by Interrupting TRAF6-ECSIT Complex. *Front Cell Infect Microbiol.* 2017; **7**:94.
9. Leem G, Park J, Jeon M, Kim ES, Kim SW, Lee YJ, et al. 4-1BB co-stimulation further enhances anti-PD-1-mediated reinvigoration of exhausted CD39+ CD8 T cells from primary and metastatic sites of epithelial ovarian cancers. *J Immunother Cancer.* 2020; **8**(2):e001650.
10. Kim D, Langmead B, Salzberg SL. HISAT: a fast spliced aligner with low memory requirements. *Nat Methods.* 2015; **12**(4):357-360.
11. Pertea M, Pertea GM, Antonescu CM, Chang TC, Mendell JT, Salzberg SL. StringTie enables improved reconstruction of a transcriptome from RNA-seq reads. *Nat Biotechnol.* 2015; **33**(3):290-295.
12. Pertea M, Kim D, Pertea GM, Leek JT, Salzberg SL. Transcript-level expression analysis of RNA-seq experiments with HISAT, StringTie and Ballgown. *Nat Protoc.* 2016; **11**(9):1650-1667.

13. Zhan Z, Xie X, Cao H, Zhou X, Zhang XD, Fan H, et al. Autophagy facilitates TLR4- and TLR3-triggered migration and invasion of lung cancer cells through the promotion of TRAF6 ubiquitination. *Autophagy.* 2014; **10**(2):257-268.
14. Min Y, Kim MJ, Lee S, Chun E, Lee KY. Inhibition of TRAF6 ubiquitin-ligase activity by PRDX1 leads to inhibition of NFKB activation and autophagy activation. *Autophagy.* 2018; **14**(8):1347-1358.
15. Son J, Kim MJ, Lee JS, Kim JY, Chun E, Lee KY. Hepatitis B virus X Protein Promotes Liver Cancer Progression through Autophagy Induction in Response to TLR4 Stimulation. *Immune Netw.* 2021; **21**(5):e37.
16. Borowicz S, Van Scoyk M, Avasarala S, Karuppusamy Rathinam MK, Tauler J, Bikkavilli RK, et al. The soft agar colony formation assay. *J Vis Exp.* 2014; **92**:e51998.
17. Franken NA, Rodermond HM, Stap J, Haveman J, van Bree C. Clonogenic assay of cells in vitro. *Nat Protoc.* 2006; **1**(5):2315-2319.

Supplementary figures and figure legends

Supplementary Figure S1

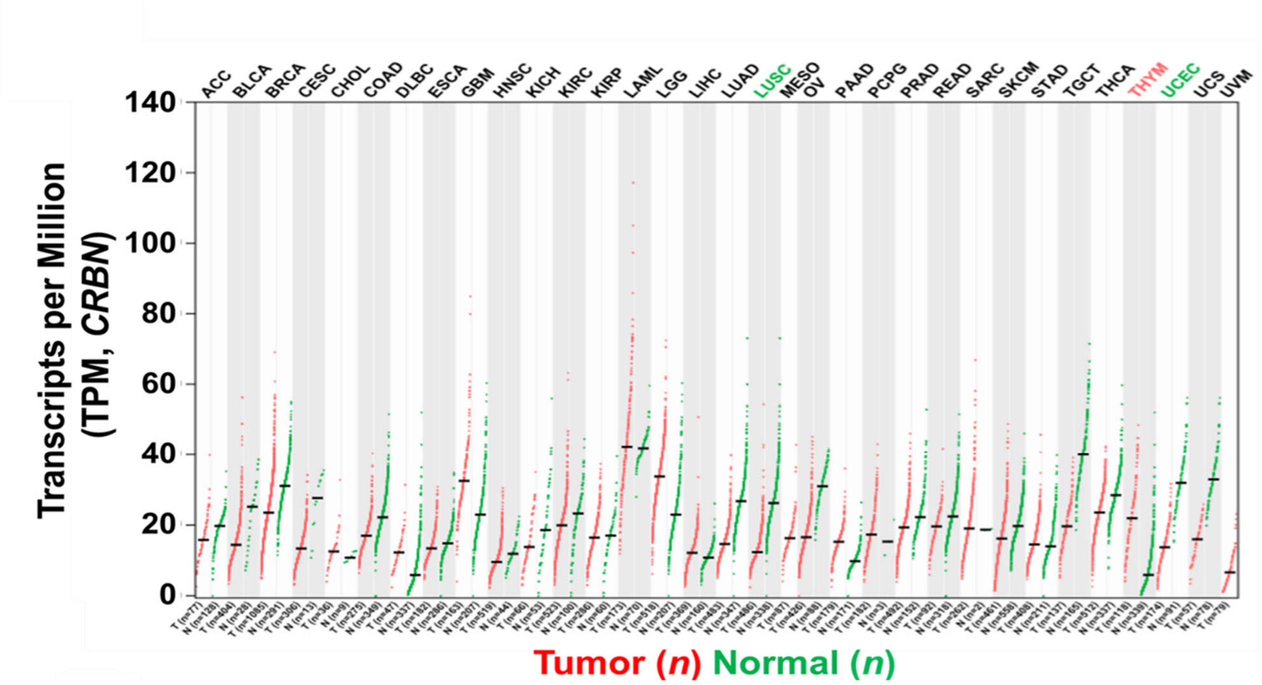

Figure S1. CRBN expression in 33 types of human cancer. CRBN expression in 33 types of human cancer, as indicated, was analyzed with data from The Cancer Genome Atlas through Gene expression Profiling Interactive Analysis (GEPIA, http://gepia.cancer-pku.cn/detail.php?gene=CRBN). Among 33 types of tumors, CRBN expression was significantly downregulated in LUSC and UCEC (upper), whereas upregulated in THYM (upper). No significant change could be observed in other tumors (upper black letters).

Supplementary Figure S2

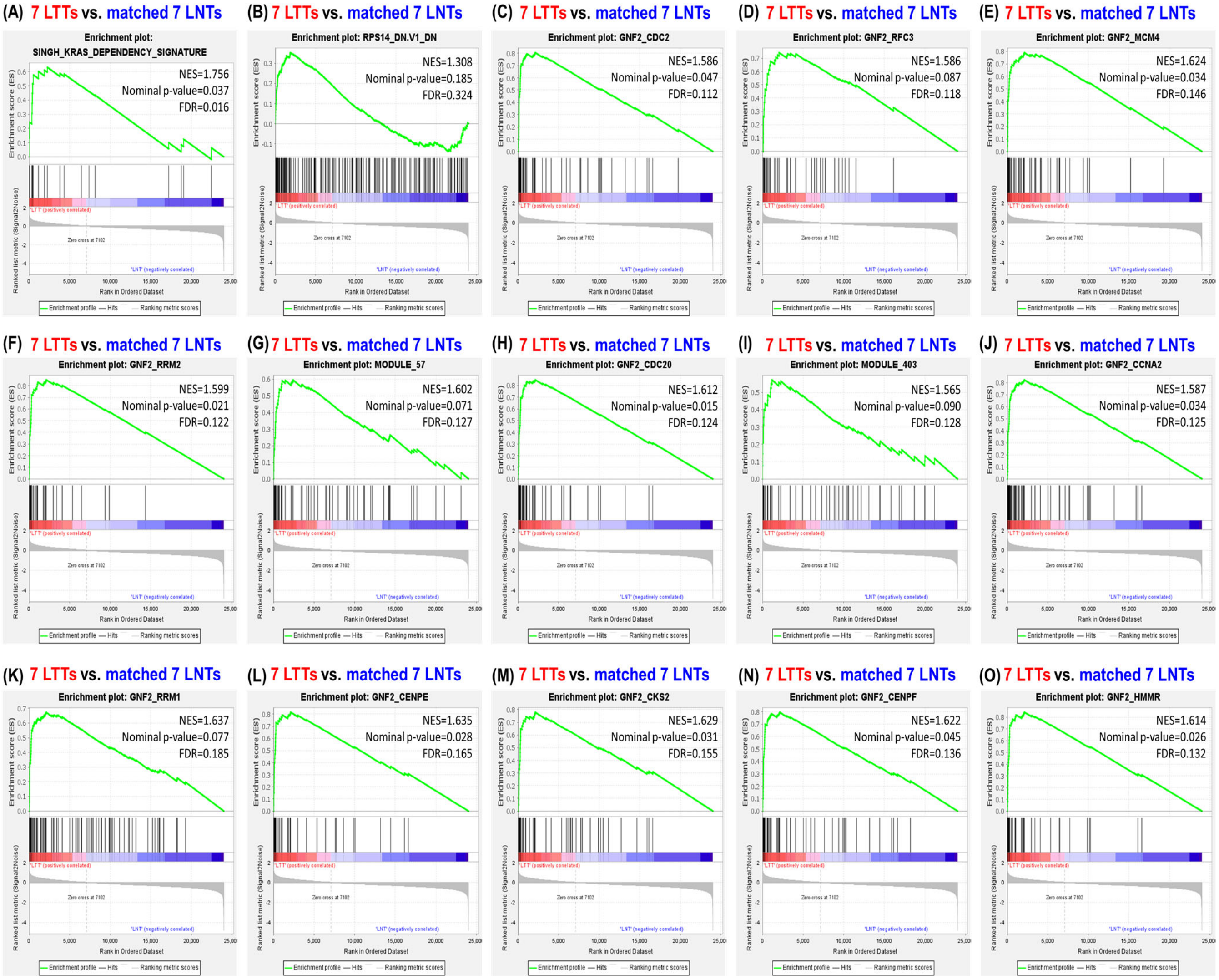

Figure S2. Gene set enrichment analysis (GSEA) between 7 LTTs and matched 7 LNTs of NSCLC patients. (**A**-**O**) GSEA was performed using the GSEA software (https://www.gsea-msigdb.org/gsea/index.jsp, Broad Institute, Cambridge, MA, USA) on the data (LTT versus matched LNT) in 7 LTTs and matched 7 LNTs of NSCLC patients. Enrichment plots related to oncogenic signature gene sets were represented in 7 LTTs vs. matched 7 LNT of NSCLC patients (**A** and **B**). Enrichment plots related to cancer gene neighborhoods and cancer modules gene sets were represented in 7 LTTs vs. matched 7 LNT of NSCLC patients (**C-O**). The top portion of each panel showed the normalized enrichment scores (NES), nominal p-value, and FDR.

Supplementary Figure S3

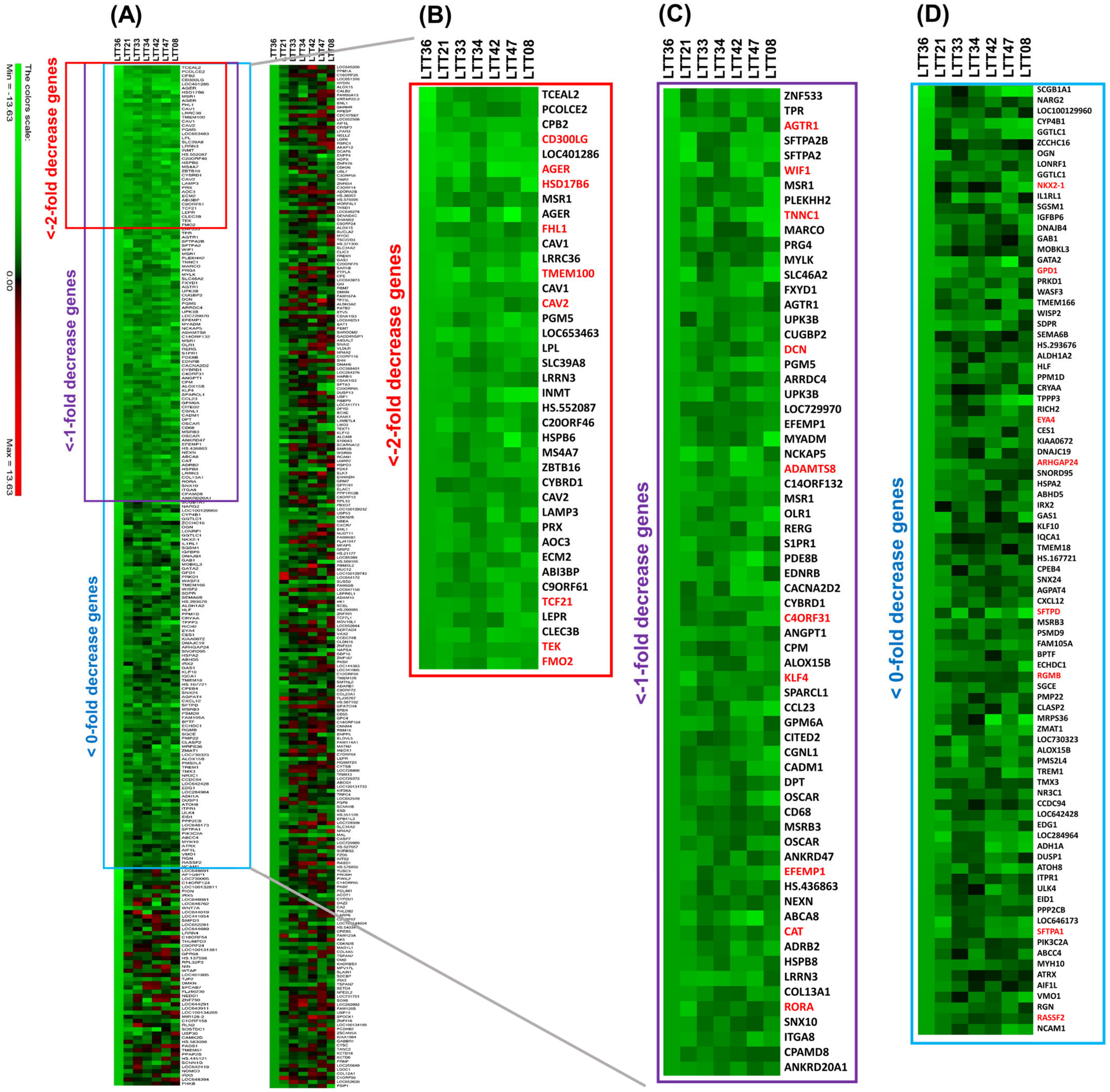

Figure S3. Downregulated genes are represented in 7 LTTs of NSCLC patients. (**A**) 500 downregulated genes based on LTT36 were aligned in 7 LTTs. (**B-D**) Downregulated genes in all 7 LTTs were sorted and presented (**B**, < -2-fold decrease genes; **C**, < -1-fold decrease genes; **D**, < 0-fold decrease genes). Genes related to anti-lung cancer proliferation, migration or invasion, and progression were indicated as red (**B-D**).

Supplementary Figure S4

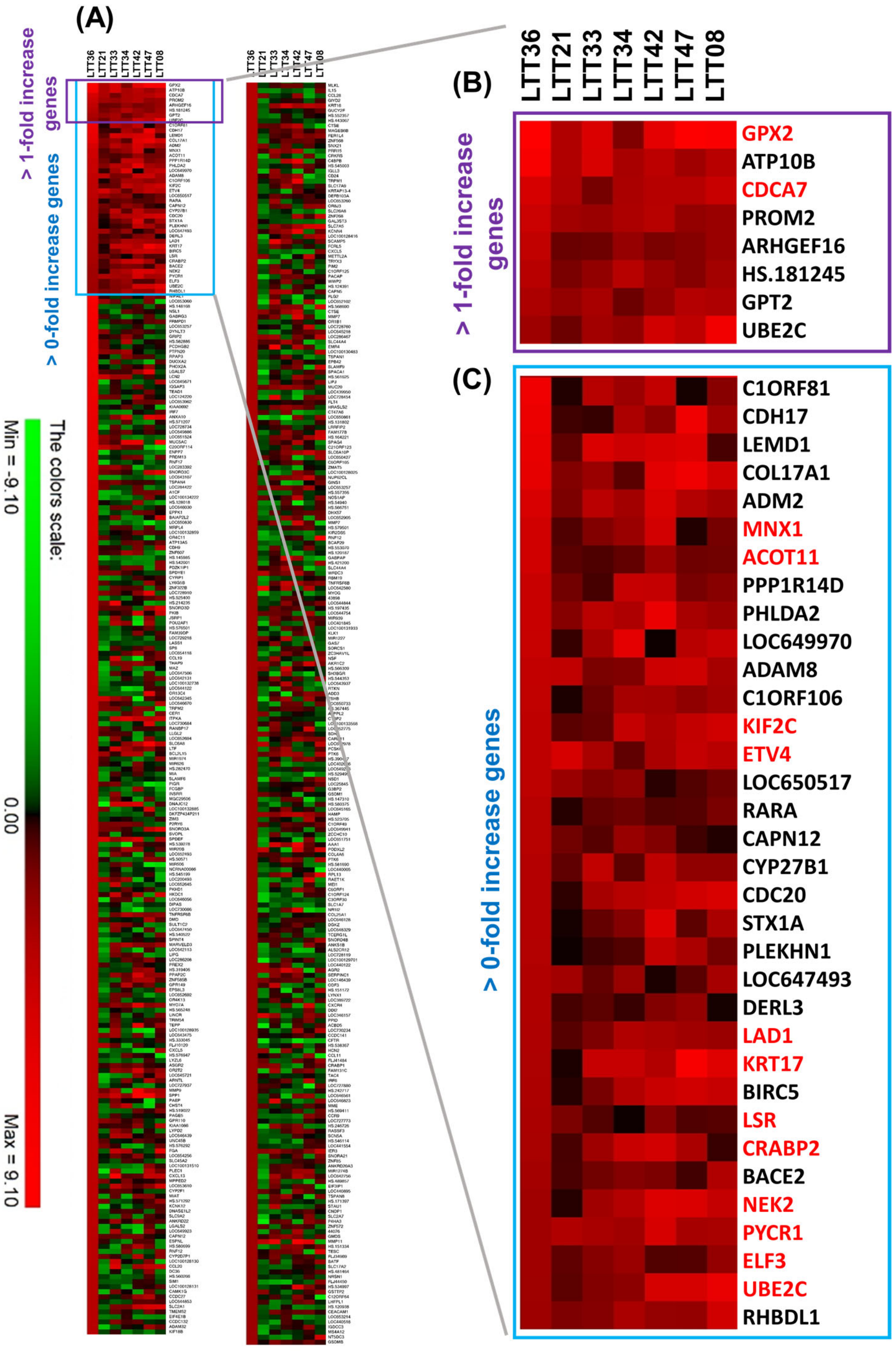

Figure S4. Upregulated genes are represented in 7 LTTs of NSCLC patients. (**A**) 500 upregulated genes based on LTT36 were aligned in 7 LTTs. (**B** and **C**) Upregulated genes in all 7 LTTs were sorted and presented (**B**, > 1-fold increase genes; **C**, > 0-fold increase genes). Genes related to lung cancer proliferation, migration or invasion, and progression were indicated as red (**B** and **C**).

Supplementary Figure S5

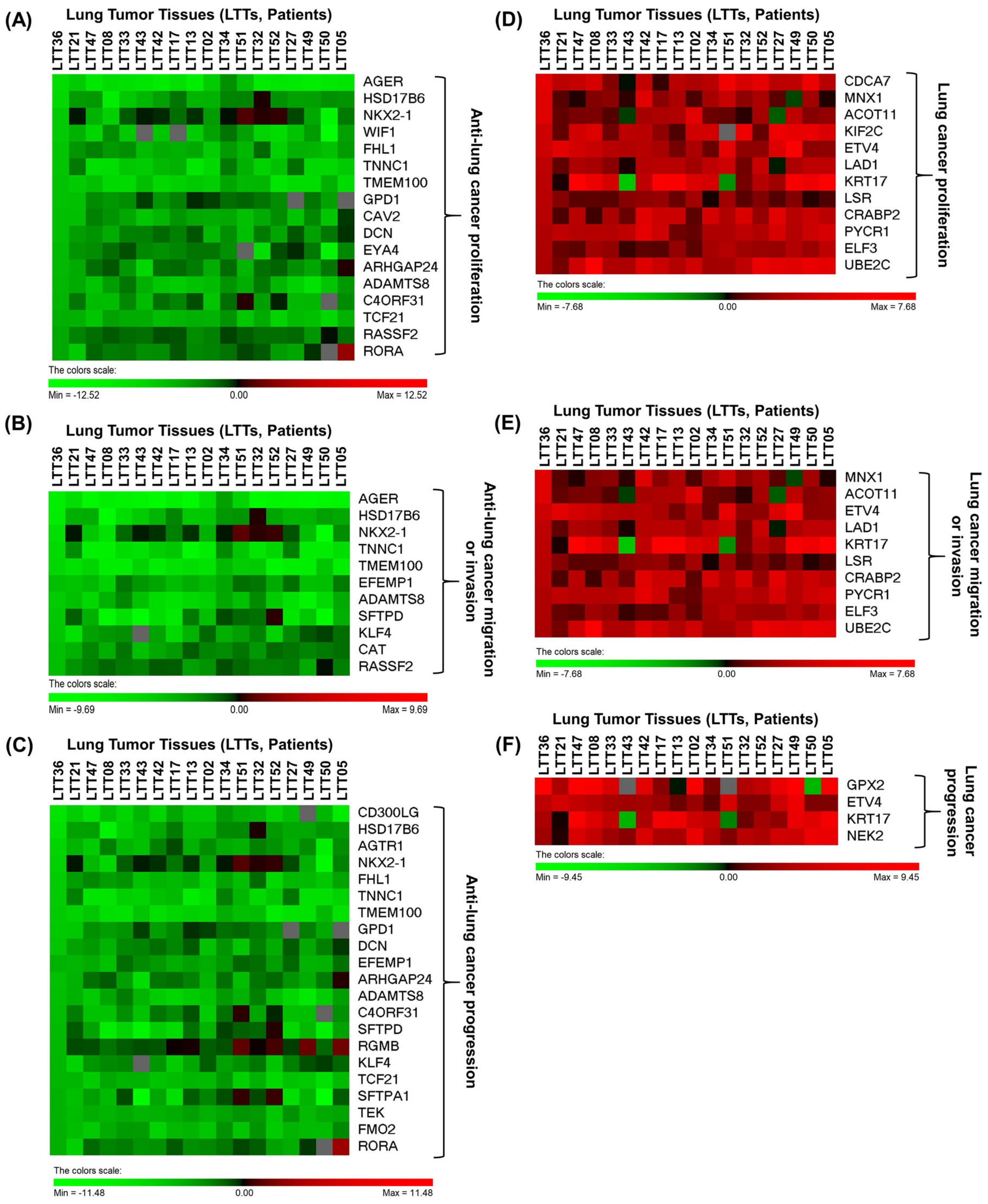

Figure S5. Heat map analysis of genes related to lung cancer proliferation, migration or invasion, and progression in 18 LTTs. (**A-C**) In 18 LTTs, genes related to anti-proliferation (**A**), anti-migration or –invasion (**B**), and anti-progression (**C**) in lung cancer were represented. (**D-F**) In 18 LTTs, genes related to proliferation (**D**), migration or invasion (**E**), and progression (**F**) in lung cancer were represented.

Supplementary Figure S6

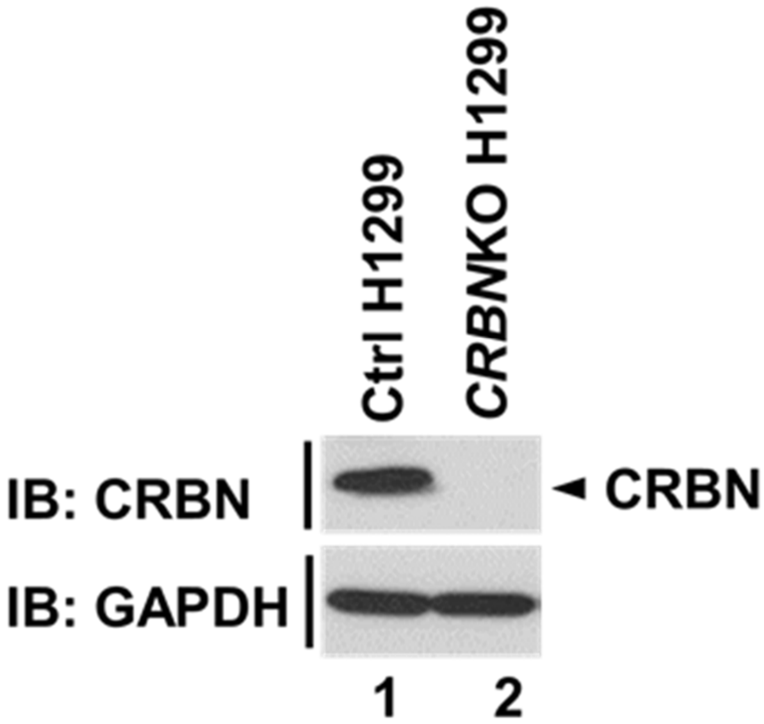

Figure S6. Generation of *CRBN*-knockout (*CRBN*KO) H1299 cells. Human CRBN gRNA sequences, 5′-CACCGATATGCCTATCGAGAAGAAC-3′/5′-AAACGTTCTTCTCGATAGGCATATC-3′, targeted to exon 2 of the CRBN gene were cloned into a Lenti CRISPR v2 vector. H1299 cells were transfected with Lenti CRISPR Universal Non-Target Control #2 Plasmid DNA (LV04 vector) or Lenti CRISPR v2/gRNA using Lipofectamine 2000 according to the manufacturer's instructions. Two days after transfection, the cells were treated with 2 μg/mL of puromycin for 3 days. After 2 weeks, colonies were isolated in the 96-well plate, and the expression levels of CRBN were analyzed by western blotting assay.

Supplementary Figure S7

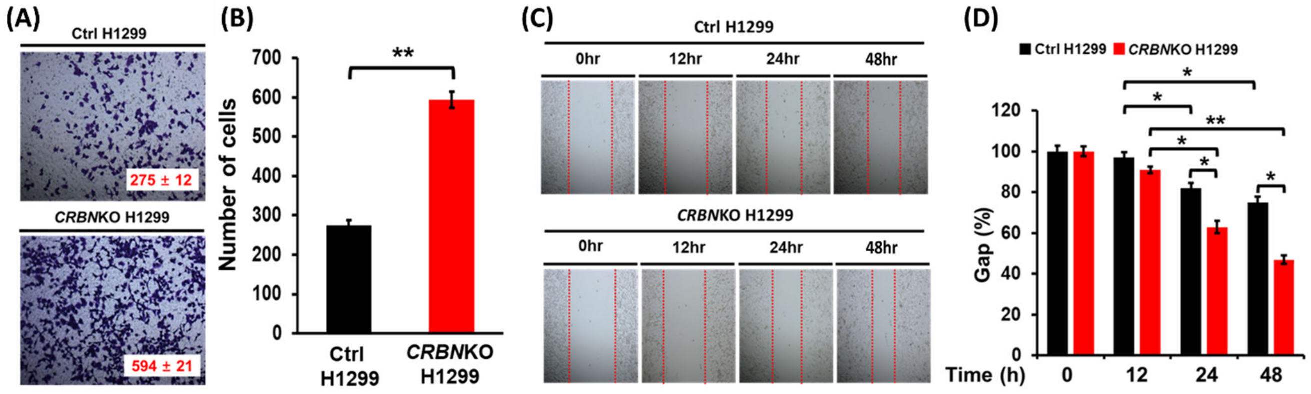

Figure S7. Enhancement of cancer migration and invasion in *CRBN*KO H1299 lung cancer cells. (**A** and **B**) Ctrl H1299 and *CRBN*KO H1299 cells were prepared, and invasion assay was performed (**A**), as described in Supplementary information. The number of cells was counted and presented (**B**). ± SD, n=3 plates. (**C** and **D**) Ctrl H1299 and *CRBN*KO H1299 cells were prepared, and migration assay was performed (**C**), as described in Supplementary information. The residual gap between the migrating cells from the opposing wound edge was expressed as a percentage of the initial scraped area (**D**). ± SD, n=3 plates, *p<0.05, **p<0.01.

Supplementary Figure S8

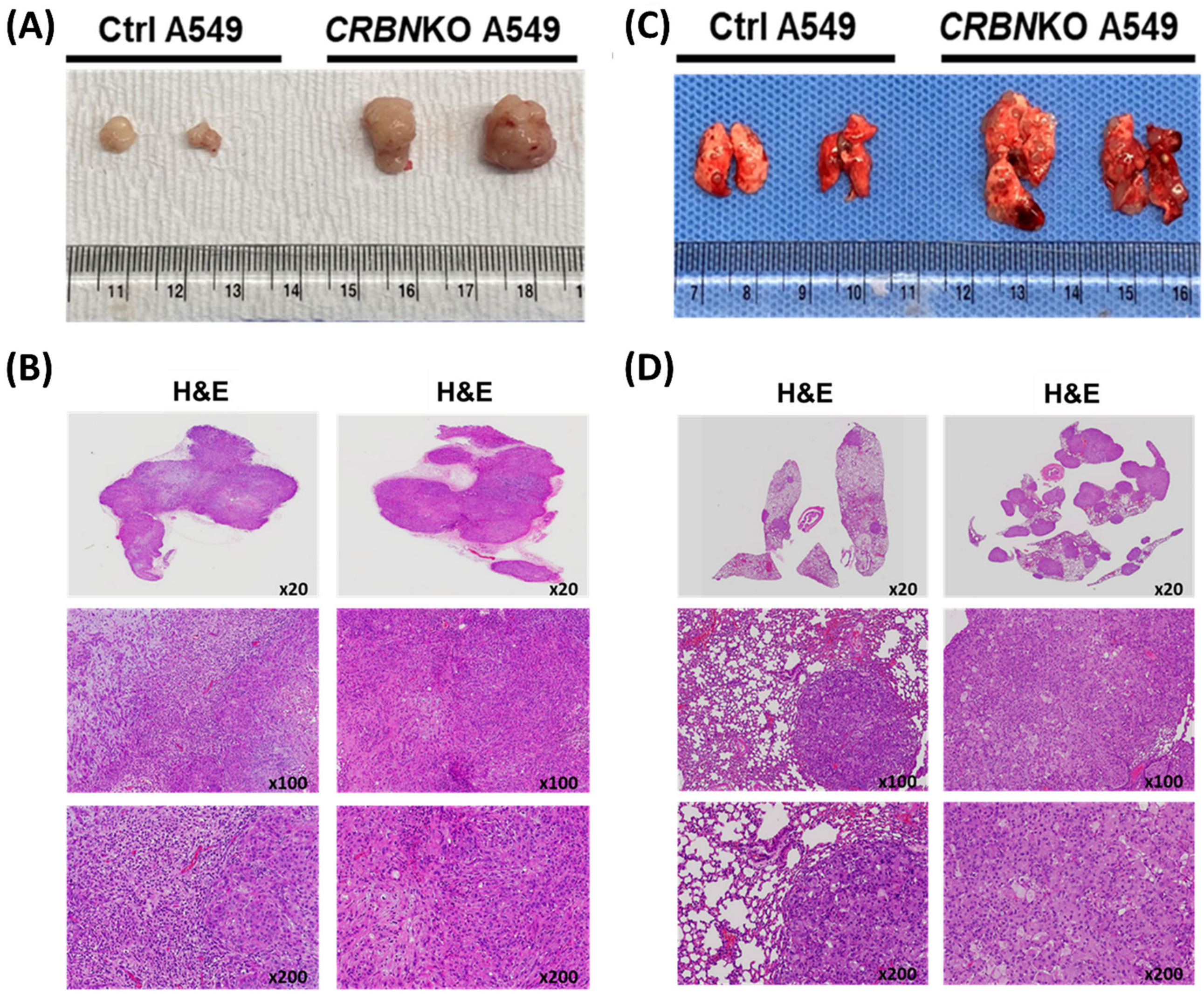

Figure S8. Hematoxylin and eosin (H&E) staining with tumors and metastasized lung tumors derived from NSG mice xenografted with Ctrl A549 or *CRBN*KO A549 cells. (**A** and **B**) Tumors were dissected from the NSG mice xenografted with Ctrl A549 or *CRBN*KO A549 cells (**A**, *n* = 2). Hematoxylin and eosin (H&E) staining was performed (**B**), as described in Supplementary information. (**C** and **D**). Lung tissues bearing tumors were dissected from NSG mice xenografted with Ctrl A549 or *CRBN*KO A549 cells xenografts (**C**, *n* = 2). H&E staining was performed (**D**), as described in Supplementary information.

Supplementary Figure S9

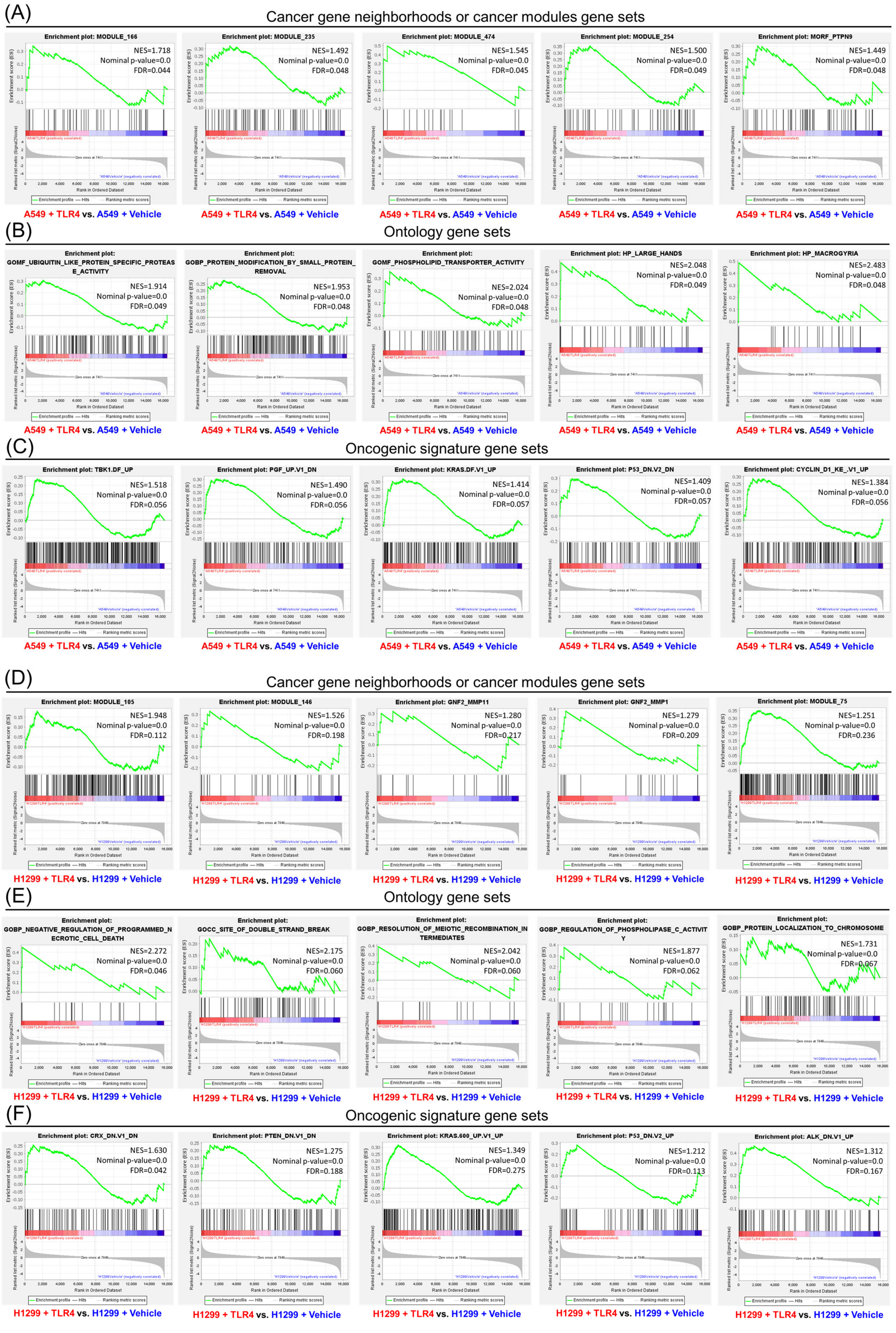

Figure S9. Gene set enrichment analysis (GSEA) of transcriptional profiles in A549 and H1299 cells treated with TLR4 or vehicle. GSEA was performed using the GSEA software (https://www.gsea-msigdb.org/gsea/index.jsp, Broad Institute, Cambridge, MA, USA) on the normalized gene expression data (FPKMs) from transcriptome sequencing data. (**A**-**C**) Enrichment plots related to cancer gene neighborhoods or cancer modules gene sets (**A**), ontology gene sets (**B**) and related to oncogenic signature gene sets (**C**) were represented in A549 plus TLR4 agonist versus A549 plus vehicle. (**D**-**F**) Enrichment plots related to cancer gene neighborhoods or cancer modules gene sets (D), ontology gene sets (**E**) and related to oncogenic signature gene sets (**F**) were represented in H1299 plus TLR4 agonist versus H1299 plus vehicle. The top portion of each panel showed the normalized enrichment scores (NES), nominal p-value, and FDR.

Supplementary Figure S10

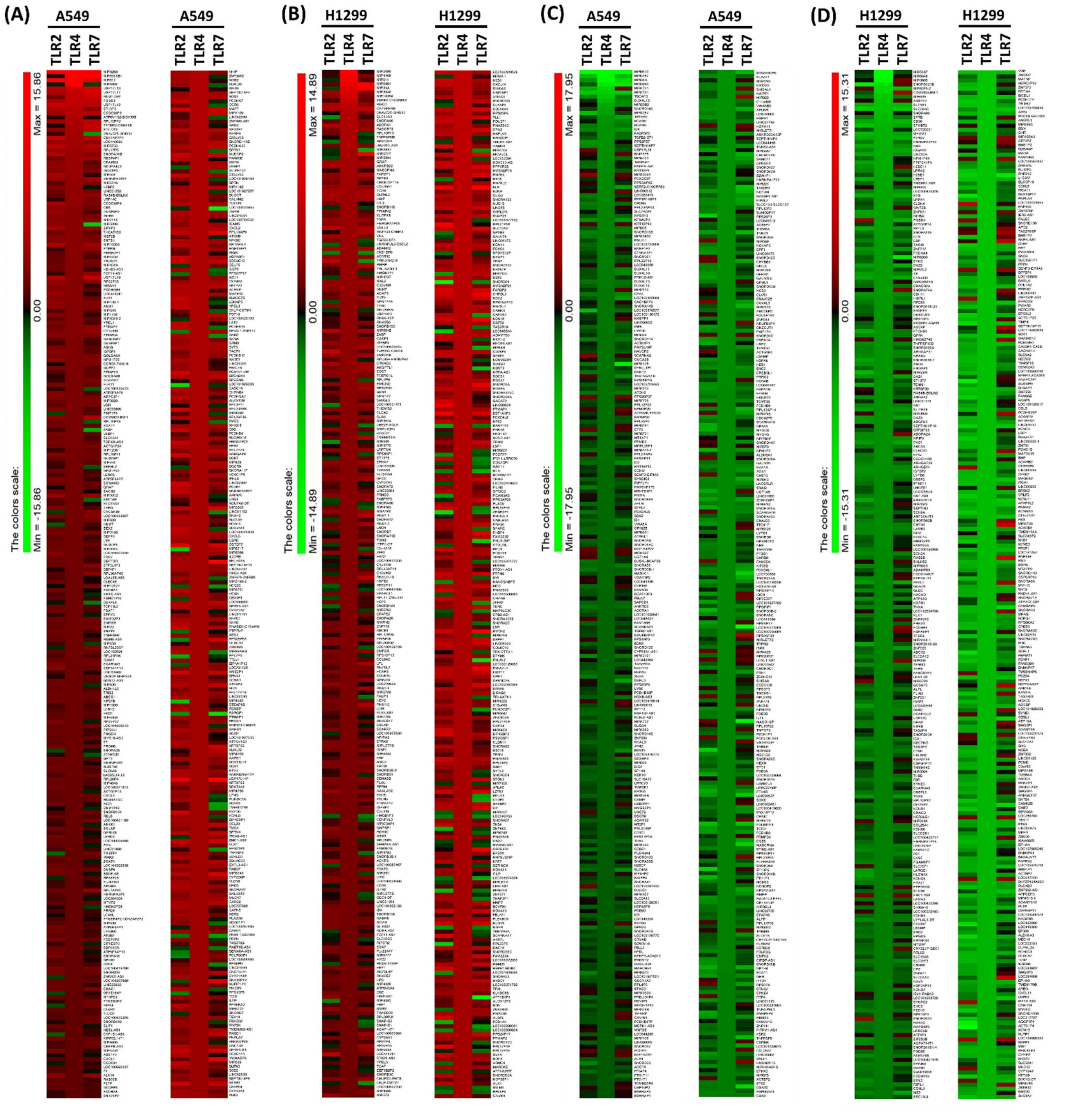

Figure S10. RNA sequencing analysis of A549 and H1299 lung cancer cells treated with TLR2, TLR4, and TLR7. A549 or H1299 lung cancer cells were treated with vehicle (DMSO, 0.1% v/v concentration), HKLM (10^8/mL), LPS (10 μg/mL), or IQM (10 μg/mL), and RNA-seq analysis was performed, as described in Supplementary information. (**A** and **B**) Based on the RNA-seq data of TLR4 stimulation, 500 upregulated genes in A549 (**A**) and H1299 (**B**) lung cancer cells were listed and represented. (**C** and **D**) Based on the RNA-seq data of TLR4 stimulation, 500 downregulated genes in A549 (**C**) and H1299 (**D**) lung cancer cells were listed and represented.

Supplementary Figure S11

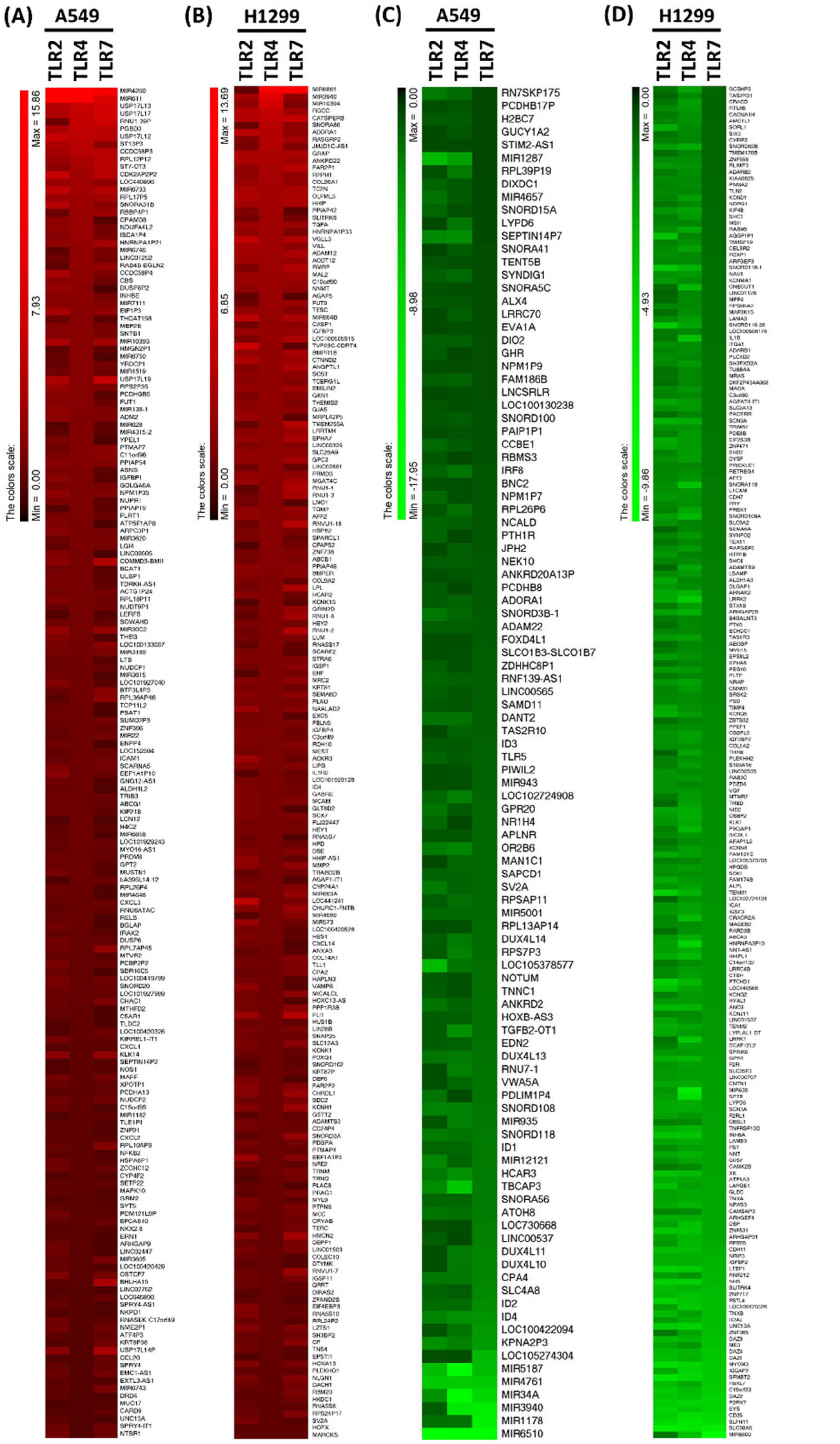

Figure S11. RNA sequencing analysis of A549 and H1299 lung cancer cells treated with TLR2, TLR4, and TLR7. A549 or H1299 lung cancer cells were treated with vehicle (DMSO, 0.1% v/v concentration), HKLM (10^8/mL), LPS (10 μg/mL), or IQM (10 μg/mL), and RNA-seq analysis was performed, as described in Supplementary information. (**A** and **B**) Upregulated genes in A549 (**A**) and H1299 (**B**) lung cancer cells by TLR2, TLR4, and TLR7 were represented. (**C** and **D**) downregulated genes in A549 (**C**) and H1299 (**D**) lung cancer cells by TLR2, TLR4, and TLR7 were represented.

Supplementary Figure S12

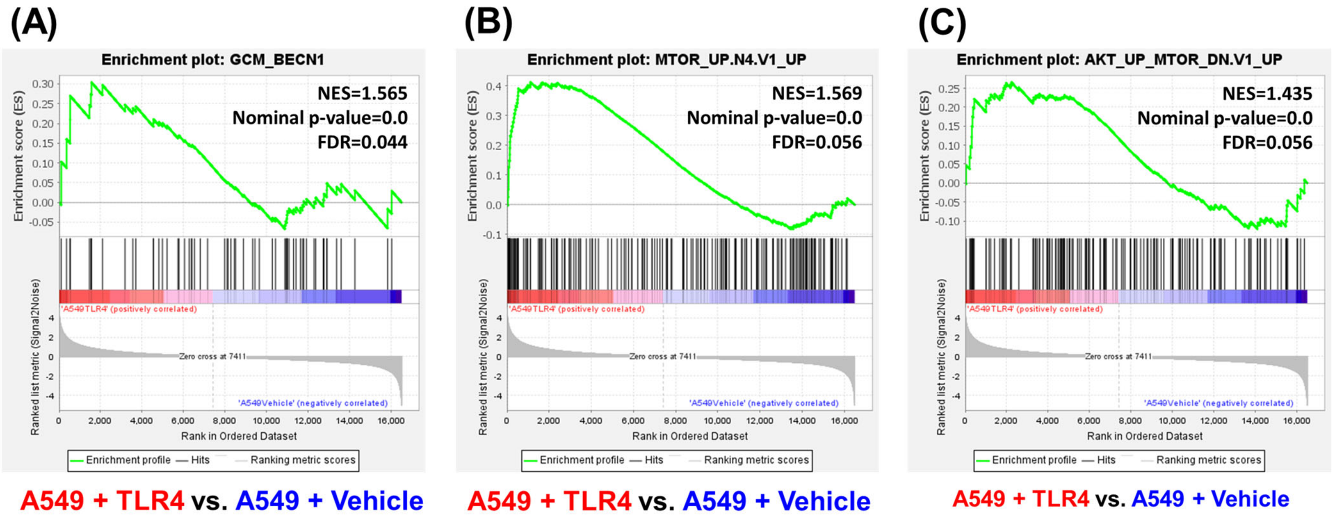

Figure S12. Gene set enrichment analysis (GSEA) of transcriptional profiles in A549 treated with TLR4 versus A549 treated with vehicle. (**A**-**C**) GSEA was performed using the GSEA software (https://www.gsea-msigdb.org/gsea/index.jsp, Broad Institute, Cambridge, MA, USA) on the normalized gene expression data (FPKMs) from transcriptome sequencing data. Enrichment gene sets regulating autophagy, BECN1 (**A**), MTOR (**B**), and AKT-MTOR (**C**) plots, were shown in A549 plus TLR4 versus A549 plus vehicle. The top portion of each panel showed the normalized enrichment scores (NES), nominal p-value, and FDR.

Supplementary Tables S1-S22

Supplementary Table S1. Fold change of CRBN expression in LTTs of NSCLC patients

Patient LTT	Fold change
	Lung tumor tissue (LTT) vs matched Lung normal tissue (LNT)
LTT27	-0.863500102
LTT33	-1.646955545
LTT34	-1.179597737
LTT13	-1.460577634
LTT17	-1.484184222
LTT21	-2.076891517
LTT36	-2.119426888
LTT43	-1.585721193
LTT51	-1.164481882
LTT52	-0.969219589
LTT32	-1.154538186
LTT42	-1.565448837
LTT47	-1.770636762
LTT50	-0.475331171
LTT02	-1.447108395
LTT08	-1.714492093
LTT49	-0.84275876
LTT05	-0.597754999

Supplementary Table S2. 500-downregulated genes above LTT36 data in 7 LTTs of NSCLC patients

Gene Symbol	Lung Tumor Tissue (LTT) vs. matched Lung Normal Tissue (LNT)						
	LTT36	LTT21	LTT33	LTT43	LTT42	LTT47	LTT08
LOC648691	-12.6973008	-1.26099842	-0.06813729	-0.23508075	1.413478094	-2.6605956	-0.10988619
AP1GBP1	-10.8005167	-2.48144281	-0.81831456	-0.7724109	0.603105286	-0.49963226	-1.68528331
TCEAL2	-10.3831616	-4.14291775	-2.96680924	-2.63962153	-3.35991968	-3.67610693	-4.68729027
LOC730005	-10.2867816	-1.77774953	-0.84218792	-1.35992777	-0.73140943	-1.69814072	-1.80416099
C14ORF124	-9.55994154	-0.09305866	-0.83077421	-0.42719356	-0.39557684	-0.44697994	0.439299397
LOC10013281	-9.49478586	0.245157455	-4.61568483	-0.06551473	1.376035038	0.606107483	-2.30805378
PION	-9.43901826	-1.469911	-1.16754361	-1.53091181	-1.76700671	-1.27053471	0.9361019
IRX5	-9.41493965	-1.36576139	-0.81927035	-2.28770373	0.767359812	-2.77943339	-4.26017896
LOC646981	-9.38286718	-1.65809373	0.519483335	1.754538685	-3.22189907	0.62759274	-0.8174691
PCOLCE2	-8.8129449	-3.51180388	-4.80993946	-2.6402914	-2.18166066	-2.37837091	-4.5393825
LOC646762	-8.61062021	-2.08570223	3.026821626	-0.05559064	-8.60363661	0.06116963	-5.15819855
WNT7A	-8.45742253	-5.25307914	-2.82238202	-1.50167406	-1.68565812	-2.29359962	-2.10205027
SCGB1A1	-8.42091358	-1.155864	-0.0754681	-1.7929834	-4.61084607	-9.00199924	-8.185961
CPB2	-8.1956778	-4.40233201	-3.71857142	-4.01385782	-5.06847018	-5.01468522	-4.84414793
LOC644019	-7.9733976	0.003616792	4.39428156	-7.26315604	0.176698835	0.023649674	2.306236395
LOC441054	-7.89783997	-1.09760818	-0.07362848	3.901328757	1.409364135	-1.86762507	-2.23682658
SMPD3	-7.79828524	-0.52944166	-1.25141376	0.096916825	2.017206927	-0.55262479	-0.81342025
NARG2	-7.60710974	-0.9627304	-0.33630323	-1.19727848	-2.61058237	-0.5465361	-1.52633718
LOC652281	-7.5774783	-0.78494875	-1.32011677	-0.52421751	0.926364449	0.29783406	-1.03122643
LOC644689	-7.56129661	-1.47006873	0.095825093	-5.22745819	1.071959171	0.113711771	0.318115246
LRRN4	-7.33052674	-1.64849423	-2.97710698	0.70144592	-1.94575309	-4.44416488	-3.6326408
C18ORF54	-7.32065221	-2.99662659	-1.46340385	-2.07673859	-0.31895413	0.363205323	1.847568809
THUMPD3	-7.22039239	-1.38453016	-0.38423341	-0.01691739	0.737128134	0.814381713	0.004033227
C9ORF24	-7.0888371	-2.03108475	0.057378264	0.077462863	-1.27047043	-4.31736402	-4.89996923
LOC10013138	-7.06165326	-2.23351784	0.52863767	0.396279418	-0.53631107	2.139827474	-0.41920784
GPR98	-7.05427047	1.624247821	-0.80349205	-0.93526363	1.062063958	2.25181369	-0.90682596
CD300LG	-6.95868337	-5.28105244	-5.75707988	-2.74988745	-5.78833015	-6.78364146	-4.40759153
HS.137598	-6.89911238	-0.26944488	-0.40571939	-0.95187212	-0.05504425	0.446470077	0.07664958
RPL32P3	-6.84431559	-1.80056055	0.828720967	-0.1144314	-2.71644202	-1.77807209	0.024840511
LOC401286	-6.83545103	-6.32052405	-5.68823565	-3.76141001	-6.35011039	-5.95349123	-6.19523347
AGER	-6.79145807	-5.50767663	-7.20881962	-2.60696733	-8.35609053	-7.95919559	-6.91237825
LOC10012996	-6.74479241	-2.28784432	-1.22646105	-1.914596	-0.60466087	-2.82006247	-0.23595197
NIN	-6.69755466	-1.12774352	1.158864909	-1.70712619	0.8119015	3.425507583	-1.52403529
WTAP	-6.60156399	0.456253836	-0.88647331	-1.40493794	-1.30260216	1.379032455	-2.82917567
LOC401805	-6.59650335	-3.75034675	-0.28624244	0.578986138	0.288426496	-0.034076	-0.5554128
HSD17B6	-6.54020106	-3.10024039	-5.38075662	-2.24286092	-3.30723154	-2.98534229	-8.47944038
TJP2	-6.46026674	-2.81086926	-2.20139421	-3.21508802	-1.7876828	-3.38752265	1.074109702
DMKN	-6.44389471	-1.86991176	-0.92054716	1.229566881	1.106368464	-1.32096329	-3.66072793
CYP4B1	-6.43044082	-0.9721183	-2.73565425	-2.42836751	-4.90487424	-5.46201243	-6.49628242
EFCAB7	-6.41076519	-0.74545705	-4.29842007	2.28600963	1.616091283	-0.66410283	0.78133031
FLJ46230	-6.41053867	-4.21344453	-1.91477869	-0.51606645	-2.52970905	-0.38968912	-2.2969849

NEDD1	-6.4088628	-2.37923132	0.895762167	-0.92035368	0.923580954	0.853722184	0.871257695
GGTLC1	-6.40390022	-0.7356767	-4.35437784	-2.06185441	-1.60339454	-5.27470138	-5.16780302
ZNF750	-6.39013508	-2.11226967	0.620215557	-7.80765144	2.265673259	1.659211318	-3.9359273
ZNF533	-6.38925604	-1.3368919	-4.2000458	-2.81528253	-3.96935918	-3.53197559	-4.46800664
TPR	-6.31681661	-2.17506948	-3.05900245	-1.89191584	-4.73730421	-2.47481587	-2.80513727
ZCCHC16	-6.26225358	-0.23560526	-0.42854338	-1.14300734	-1.02718728	-0.28462646	-0.01432412
LOC644291	-6.23033636	-1.49940747	-0.38966912	-3.18720512	-2.39871431	-4.14286431	0.219326303
LOC643911	-6.17269768	-0.7465597	0.421911937	-0.65832035	0.921186022	-2.33138495	-2.59128761
LOC10013426	-6.17182301	-4.66588938	-3.05314766	-2.44758261	-2.91905478	0.594199717	2.242400083
OGN	-5.94242476	-5.62483521	-0.50829618	-2.71835873	-2.08333438	-2.56077973	-1.6454477
MIR128-2	-5.9103681	-0.16555806	-0.82289597	-0.70822865	-0.4544619	-0.60420819	0.628602405
C1ORF158	-5.88384271	-2.12270273	0.199896168	-0.8411166	-0.61363199	-1.96022519	-4.64380316
MSR1	-5.85445206	-3.36275167	-2.1381223	-2.87155545	-2.00413845	-3.87086489	-3.03794663
RLN2	-5.82905583	-3.07455813	3.511969977	0.308476752	-0.07362412	-0.09658082	-1.7499005
SOSTDC1	-5.77402312	-5.01103696	-5.11994491	-3.30456457	-8.2871836	0.020201964	-2.1284137
USP30	-5.77178354	-2.15272167	-5.84831449	-1.27399715	-0.05782711	-0.42062118	2.209762259
CAMK2D	-5.76920451	-1.6364252	-0.63507004	-2.20988964	0.182187144	-1.35838468	-0.13593357
HS.583098	-5.75463652	0.518888084	-1.32135934	-1.66251498	0.126741357	-2.08217638	-0.5711035
AGTR1	-5.7474551	-3.83944735	-3.92458329	-3.4247347	-1.96239768	-5.05709199	-1.88403644
FADS1	-5.73567329	-2.50903601	-0.30120078	0.325422842	-0.71105477	2.657673043	1.927894996
TMEM61	-5.7347996	-0.56802548	0.629551778	0.157827493	1.094338681	-1.74165011	0.102885983
LONRF1	-5.73009152	-1.73943082	-1.41812981	-1.75040259	-2.7194526	-0.64304573	-2.06190967
SFTPA2B	-5.71960252	-7.61900337	-1.71834566	-1.59311595	-4.8339091	-4.79717514	-5.77552989
PPAP2B	-5.70555461	-2.770777	-1.04572031	-0.87134231	-1.95256846	-3.02190335	0.94316323
HS.445121	-5.6976767	-1.17449958	0.32619421	-0.36782723	-1.79680018	1.438655502	-0.83279846
SCNN1G	-5.67799626	-1.97450895	0.687218671	-1.88133708	-5.17589858	-2.25219044	0.562337912
LOC642419	-5.65269873	-0.53057046	2.125385502	-1.41575563	-0.95803691	0.706325376	-0.15752222
NOMO3	-5.63588819	-3.30042794	0.223338983	-0.51151175	-0.11097097	0.39822444	-0.6298931
IRX5	-5.62297207	-0.70905426	-0.64408336	-1.67958071	0.613254997	-3.11024988	-3.62567218
GGTLC1	-5.5957145	-0.99140492	-2.96214262	-2.15187752	-1.59638687	-5.03805889	-2.73703996
SFTPA2	-5.57467917	-8.21398368	-1.55545748	-2.02581199	-6.11385936	-5.01418019	-6.16179299
LOC648394	-5.57133739	0.34961136	-0.1131025	-0.23203838	-1.07097688	0.848175284	1.989975835
PHKB	-5.53980089	-1.41741364	-0.4887163	-0.39255601	0.47143207	-0.12273836	0.098064607
LOC645200	-5.51854013	-1.16697409	-0.11912854	0.32337279	4.793520303	-0.93221535	0.828235516
NKX2-1	-5.50716381	-0.06513499	-1.06704943	-0.04671198	-0.27088388	-4.13760292	-5.84334805
PPM1A	-5.487879	-2.18560923	0.003671828	-0.60979078	-0.16046964	0.549687921	-0.46730385
IL1RL1	-5.47071214	-0.4939973	-5.24505678	-2.12382097	-4.42149938	-2.55344736	-0.1481945
WIF1	-5.4647533	-4.11490621	-5.43042954	-1.7824146	-5.26298829	-6.27091216	-2.50501747
C18ORF26	-5.45347873	-1.04082777	0.460567009	0.506095297	0.811058024	-3.22085638	0.123469472
LOC651316	-5.4477967	-3.05244087	-0.96701689	-0.66111379	-0.14803325	-1.78808093	3.65646937
HYDIN	-5.42205515	-2.17424947	-0.1122095	-1.22917082	-0.42720372	0.364082274	-3.60004331
ALOX15	-5.40525103	-1.17629677	-0.39636108	-3.92618612	-0.18993043	-0.69424719	-2.66319471
CALB2	-5.36979127	-3.67287671	-0.71073168	-3.07640637	-1.47153352	1.339843296	2.141248556
FAM90A13	-5.35438136	-0.3925805	-0.71884619	-6.07345268	0.32926612	1.123669365	0.716224341
KRTAP22-2	-5.34513563	-0.92769757	-0.13145744	1.137425895	-0.74675291	0.713529129	-2.0828389
AGER	-5.34505444	-6.77661025	-8.12969774	-2.3275467	-5.7403151	-13.6265415	-4.14456595

EML1	-5.34050279	-3.22045935	-1.18676731	-1.53276781	-1.91282878	0.107810356	-3.3571168
SGSM1	-5.33770284	-1.24753758	-1.05313921	-0.40047358	-1.54404092	-4.42879302	-1.93991447
GHRHR	-5.33208288	-1.18865812	0.439639295	-0.1235269	-0.27504364	-1.22404678	3.352802501
RPESP	-5.33188171	-2.55537945	-1.94993758	-3.14246352	-2.24101901	2.232277154	-2.95780239
CDC42SE2	-5.33135406	-4.02204624	0.932707135	0.522391999	2.368101993	1.954933204	0.180346926
IGFBP6	-5.32857753	-1.96876577	-1.86737908	-2.80601816	-0.581887	-2.0463846	-4.37024867
LOC652508	-5.31752248	-2.43421811	-0.19065599	-1.61874661	-1.22664676	-0.45063677	1.018390249
AIF1L	-5.30817544	-4.47889234	-2.07466907	-0.61088445	-1.66787703	0.126674151	-4.31020165
CRISP3	-5.30035911	0.05564277	-0.11007753	1.013320588	0.329504257	0.380735724	1.173747828
LPAR3	-5.28807513	-2.09158663	0.588832902	-1.52021486	2.007072214	1.513759084	-0.5232544
NELL2	-5.26653499	-3.84634305	0.968636497	-1.69818236	-1.8364482	1.357826918	-3.2134496
DNAJB4	-5.24823231	-3.44463176	-0.95576876	-2.04229531	-0.92107467	-0.49833413	-3.89887607
FHL1	-5.2341466	-2.88266141	-2.59113639	-3.10816742	-3.84232856	-2.12692508	-2.96897811
MSR1	-5.23377045	-3.04769901	-1.56943861	-2.0134993	-2.30105325	-3.55787329	-6.66544709
LGR6	-5.2332979	0.698288335	-0.35602199	-0.13132716	-1.30743577	0.985200873	0.654478073
RSRC1	-5.22876099	-2.00189165	-0.46728706	-0.72627967	0.437181175	2.282914017	3.049307542
AKAP12	-5.21979573	-5.26089412	-2.37579313	-2.06538645	-1.68049309	-0.95033431	2.951278493
DCAF6	-5.21883073	-1.70542953	0.133006712	-1.28555142	-0.15111872	0.454152495	-1.36710945
PLEKHH2	-5.19782599	-3.39531585	-1.72181082	-3.79032329	-2.48339531	-2.37583847	-6.70791912
ENPP4	-5.18279168	-1.15918792	0.187260767	-0.75576051	-1.04619009	-10.2758242	-3.75463036
CAV1	-5.18190654	-4.7105099	-4.25352849	-2.70460559	-3.50572815	-3.0604721	-3.80338373
HOPX	-5.17475341	-0.84323217	-1.18373315	0.599514744	-0.72826966	-2.99922747	-2.28697145
TNNC1	-5.17323456	-1.90728894	-5.34210417	-1.88558226	-4.47418134	-7.07228914	-6.96373625
ZNF470	-5.16687911	-0.90234326	-0.08171408	-1.1485608	0.205649162	-1.80926482	-0.76672961
MARCO	-5.14326737	-3.28415297	-4.8940604	-1.10021477	-1.07295139	-3.3139831	-7.37912743
CDH26	-5.1344595	-2.82117659	0.717901705	0.495726558	-2.40841778	-2.39967599	-1.68466406
UBL7	-5.12314251	-3.12030897	-6.58786593	2.184832044	0.515699587	-1.38080534	-0.43979411
C3ORF58	-5.11860629	-3.01647099	-1.04496849	-2.47825242	-2.37642244	1.214347801	-1.24499814
PRG4	-5.11800694	-1.91920808	-2.53353513	-1.40439642	-1.80582887	-1.56777534	-2.473373
TINF2	-5.10378822	-1.79060797	-0.61971384	-0.63927678	-1.04073154	-0.32688872	-1.27641393
GAB1	-5.09206363	-3.01766181	-0.90892646	-1.89948403	-0.48667921	-0.03339658	-1.94662622
MYLK	-5.08571851	-3.24994157	-1.02690644	-2.70214555	-1.50005957	-1.93048718	-5.45738703
MOBKL3	-5.06369245	-1.44394656	-0.16799684	-0.89700455	-0.3198463	-0.8097779	-1.50490708
ZNF654	-5.05238255	-2.42715537	-1.27646533	-2.47956755	0.142107652	-1.96419778	-0.99209464
C3ORF14	-5.04084938	-1.39561604	-0.24597069	1.504889847	0.882994884	-1.05019197	-0.99976005
ADORA2B	-5.03635994	-0.27808793	1.775853791	1.214214663	2.025809889	2.698656906	1.145254784
SLC46A2	-5.03152223	-6.39268579	-1.40988117	-2.83837308	-4.40366813	-4.29999574	-7.31563791
HS.36053	-5.0248508	-0.72784182	-0.16938938	-1.26794998	0.070428131	-0.46178183	-1.24171545
HS.575095	-5.02241676	-1.66654014	-2.51013532	-0.65142689	-0.30408153	0.16848172	0.113425499
GATA2	-5.01621552	-4.46336145	-3.96885823	-1.75317925	-3.49396041	-9.43049626	-0.48485597
MORF4L1	-5.01119615	-4.01839482	-0.33458223	1.200779147	0.485623848	1.141329842	-1.87616816
LRRC36	-5.00636055	-5.59487451	-2.67007942	-3.62230499	-4.30214457	-3.56072931	-6.79525549
THSD1	-5.00402679	-3.5272804	-3.60845964	-1.06812371	-2.58450245	-0.89135299	0.179380044
TMEM100	-5.00056052	-5.62474307	-6.68112286	-3.52007985	-6.51332337	-4.89437077	-6.59518728
FXYD1	-4.98610378	-3.48559936	-2.90928734	-2.95028974	-4.34892015	-5.36860863	-1.62329553
GPD1	-4.97943635	-4.9470724	-2.53868369	-1.52307607	-2.89722409	-2.4682942	-2.94342328

CAV1	-4.96514454	-5.34244281	-4.1607789	-2.82566571	-3.08951903	-3.59166466	-3.63839265
PRKD1	-4.96263505	-2.27013365	-1.66451879	-3.05083561	-1.01745345	-3.37986292	-0.8213416
AGTR1	-4.96196135	-4.15376063	-3.63573805	-2.27343793	-2.52434247	-2.90920927	-1.35615879
WASF3	-4.92939696	-1.10558495	-2.67919726	-1.78352029	-3.92058912	-4.29610904	-0.38374581
UPK3B	-4.92540108	-1.07809183	-3.53033884	-1.70898599	-2.75665395	-3.42014375	-3.13900227
LOC646278	-4.92111845	0.898271118	-0.49584257	-0.04212549	1.20849454	0.2356079	1.432790326
DENND4C	-4.92066756	-3.12296705	-1.31986508	-1.59786233	1.661689509	-1.70101008	-3.08832428
SHANK2	-4.89999399	-1.072242	-1.01158398	0.119404569	0.231667549	-0.83255084	-0.31611489
C9ORF24	-4.86223119	-1.87436704	0.125825055	-0.25085909	-0.95248644	-4.31803184	-5.40975068
ALOX15	-4.84544376	-1.90278701	-0.64064349	-0.60474633	-0.79992502	-1.83801731	-2.20936386
SUCLA2	-4.8400943	-1.28197955	-0.03913088	-0.56610443	0.771240089	-0.67596769	-0.42903629
MYOC	-4.83648799	-2.12077819	-1.60866657	-0.25590324	0.650122402	-1.36006999	0.75372381
TSC22D3	-4.82811675	-2.66820778	-1.67057916	-2.29067218	-3.35691682	-2.06397103	2.228304828
HS.371300	-4.82275923	-1.85033109	-0.46245027	0.231183228	-1.07042964	-0.69970712	1.216444477
SLC34A2	-4.81656827	-1.07128109	-0.31607708	-0.24309702	1.150373013	-4.46073794	-3.10340916
TMEM166	-4.814182	-0.7901836	-1.40056825	-2.42495323	-0.10898699	-3.47451873	-3.8758874
CLIC3	-4.80614174	-2.54782779	-5.45113457	-1.01690826	-4.48459935	-4.59713776	-2.13848516
FREM1	-4.80451339	-1.95647454	-1.11969018	-1.16397378	-1.04014324	0.236898661	-1.63972627
GAS1	-4.79760247	-5.0694619	-0.52254708	-3.07835653	0.142378763	-0.78612268	-1.02893533
CAV2	-4.79453441	-4.82006364	-3.62757704	-3.60855218	-4.29112711	-2.14361962	-2.84270266
WISP2	-4.78256696	-3.01230437	-1.90844001	-2.30759429	-2.1427919	-4.05742732	-0.80645469
C20ORF75	-4.78093703	-1.20147653	-3.18729953	0.503867275	-1.84559764	-3.27542278	-4.92642354
CUGBP2	-4.77751434	-3.99110434	-2.53393893	-1.69959679	-3.46242183	-4.7678108	-2.62940005
SAR1B	-4.77260862	-0.92512749	3.194183146	1.356799665	2.828339184	1.024562516	0.360960648
DCN	-4.76678742	-3.14423235	-2.12279808	-2.56509566	-1.45351816	-2.75738764	-3.79938333
PTPLA	-4.76479233	-2.63151534	-1.97652306	0.554417323	-0.91556942	-2.35081698	0.334761053
CPE	-4.75916049	-3.50388511	0.297821734	-0.32514802	2.147608994	-0.45694978	1.573309611
SDPR	-4.75864938	-4.56647022	-2.87483783	-3.25771385	-5.51648085	-2.23385441	-0.38135538
LOC643873	-4.7572679	-3.26345816	-2.23531875	1.064786872	0.015703473	-0.51567421	-0.02960826
QKI	-4.74932288	-2.7856336	-2.52741495	-1.04603266	-1.20585589	-0.83808214	0.326869734
RBM7	-4.74276416	-2.53802007	0.204913752	-1.19029543	0.021430921	-2.12980278	-2.00325138
PGM5	-4.73997008	-3.78473908	-1.80098763	-3.21003854	-4.34169634	-5.57916463	-4.50964204
DMKN	-4.73932124	-1.68379438	-0.48641295	1.562671628	0.544350123	-1.02242023	-1.93437613
FAM167A	-4.72810694	-0.24539659	-2.03404649	0.917052736	-0.90479075	-5.15918975	-0.26505895
TP73L	-4.71246268	-1.53137882	1.511167826	-1.27303453	0.810220673	7.624164808	0.224607874
PGM5	-4.71219729	-4.17400791	-2.25507724	-3.12147238	-4.82749693	-6.7853024	-4.64215625
LOC653463	-4.69996744	-5.99591145	-5.24254787	-3.58112285	-5.82854383	-4.91803679	-5.67065417
ALDH3A2	-4.67998748	-1.39519421	-0.71881947	0.840270178	-2.44000073	1.064987441	0.114857835
SEMA6B	-4.67852695	-0.3077297	-0.87601209	-1.44558274	-0.54342484	-0.0942963	-1.34644956
PATE2	-4.66644219	0.720154202	1.784398034	0.698498466	0.22393572	-1.1616931	-0.13404161
ETV5	-4.65581984	-2.43696988	-1.06270391	-3.04098983	-1.4023368	-2.20921607	-5.39152198
LPL	-4.64502191	-3.55607362	-4.77172344	-2.22566475	-3.51080222	-3.90940051	-4.71520894
HS.293676	-4.64328598	-1.83340467	-0.40475429	-2.10109991	-0.37145591	-1.35413456	-1.01632689
ALDH1A2	-4.64036577	-3.40997443	-1.26057185	-0.16915514	-2.23426039	-2.6442315	-1.51988393
HLF	-4.622557	-3.78361408	-0.31404624	-3.25279344	-1.985468	-0.71530443	-3.77406017
PPM1D	-4.62032114	-2.81823795	-1.63713946	-2.02770014	-1.29015275	-0.72120144	-3.20605595

CRYAA	-4.61827904	-1.4800114	-0.01138959	-0.11323228	-0.90108633	-3.27860112	-0.52634401
CSNK1G3	-4.61812188	-2.61486026	0.517227987	0.697916081	0.616751827	1.203467968	0.400131513
LOC648251	-4.6147649	-0.05553705	-0.52096034	-0.07254028	-4.68000373	0.555910752	-0.08286191
BAT1	-4.61421922	0.226000398	0.263157635	-0.26103283	0.436392574	0.259059622	-1.34392398
PEMT	-4.6040354	-0.79775159	-1.34299795	-0.26317859	-2.63950471	-1.11114779	1.522046586
SHROOM2	-4.60306051	-0.82754898	-0.55659884	-0.8295562	-0.77235774	0.633927255	-1.69298413
GADD45GIP1	-4.58218904	-1.692051	-0.41810238	-0.24307594	0.23591755	1.415284738	0.425869611
A4GALT	-4.57897789	-1.77131	0.269131011	-2.40248683	-1.07945254	0.91151728	1.409369512
TPPP3	-4.57419746	-3.22394455	-0.5331501	-1.7993437	-3.53634152	-5.9428082	-7.96232194
SNAI2	-4.56507742	-3.63457294	-1.55385024	-2.42393334	1.841811701	2.68103672	-0.70580994
VLDLR	-4.56190049	-2.14963274	-1.07896426	-0.88849586	0.642978576	1.464535961	-4.06534089
NR4A2	-4.55700416	-4.26727754	1.814252695	0.966798807	-3.75700797	-1.09483647	3.68032773
C1ORF116	-4.55350308	-1.3606325	-2.90301187	0.035422555	-1.00143846	-3.76603025	-2.77886086
RICH2	-4.55329487	-1.75629921	-0.54810227	-0.31454704	-2.22008254	-3.79606703	-1.63222882
SHH	-4.53025307	-0.48188785	-0.05342159	0.963492879	0.863365766	0.834812652	1.266284491
EYA4	-4.52982649	-2.84708451	-0.86742152	-2.6414716	-2.69623312	-2.52807997	-1.59341341
DNAH6	-4.52537752	-1.75700296	-0.36514133	1.286470552	-0.74093733	-1.88898982	-2.13454727
ARRDC4	-4.51965309	-3.02233993	-3.39393936	-2.76344064	-1.70552431	-2.13867484	-3.26794613
CES1	-4.51721209	-2.3186638	-2.46244382	-3.20297208	-2.80851045	-0.31875604	-2.0487725
LOC388401	-4.51714857	-0.88381903	-0.7209535	-0.15393629	-0.82096791	-0.74315904	1.171504537
SLC39A8	-4.51514046	-4.41432721	-2.45255926	-2.00897629	-3.12294133	-4.39468313	-3.24726495
UPK3B	-4.51348069	-4.05228117	-4.83450069	-1.73923857	-2.7916511	-4.67032835	-3.85146276
LOC284276	-4.51119446	-2.66980602	0.133214122	-3.4000864	-0.16379928	-3.52645595	-3.81707148
HARBI1	-4.50753545	-0.77540771	-1.11703776	-0.03009098	0.848229532	-0.67395917	2.384849374
CSNK1G3	-4.50325591	-2.15329959	0.225009235	-0.09226041	-0.65398655	-0.57244806	-1.58383727
KIAA0672	-4.50248605	-2.10070804	-0.86635551	-0.40775963	-2.24298097	-3.87615773	-2.13127375
SFTA3	-4.50128859	-0.89485643	-1.19501952	0.11983449	0.235516456	-4.86803773	-7.7556237
C20ORF85	-4.50039207	-1.22500312	0.22729667	-0.08941179	-0.0919678	-5.37329645	-7.15058305
LOC729970	-4.49991565	-1.85650611	-1.86225003	-2.27920479	-1.83079913	-2.19457188	-3.03131018
DUSP13	-4.4970588	-1.28153325	-2.14121848	-2.37583992	0.857580213	2.555002849	-4.9236198
USP1	-4.49447914	-2.26012999	-0.93680144	-0.07828981	0.113213693	-1.06103185	0.793157448
RBBP9	-4.49247025	-2.40299547	-0.50211323	1.4992236	0.512780608	-0.73247032	-1.72612145
EFEMP1	-4.49062803	-3.24743902	-1.45033323	-2.09952091	-2.11195844	-2.99852979	-3.31955295
DNAJC19	-4.48734657	-1.7394943	-0.92971174	-0.12379365	-3.99371127	-0.50668788	-1.30477082
LOC441711	-4.48474213	-2.32460656	-0.40739815	0.459827647	0.28506577	0.174536235	-0.99795723
LRRN3	-4.48218936	-4.31106575	-3.5269921	-2.99982753	-3.62531887	-2.85899672	-2.22099503
ARHGAP24	-4.47917164	-3.74450495	-2.92183033	-1.5091272	-1.58225499	-1.52754894	-0.99304065
DPYD	-4.46922187	-1.36378557	-1.53161023	-1.68290144	-0.2269891	-2.2878692	-2.39815194
BCHE	-4.45479428	-4.3174107	-1.81095865	-3.43015766	-4.31907876	1.360235953	-3.71189045
KANK1	-4.4500123	-1.02144116	-1.67194928	-1.43683414	-0.1132192	-0.15666333	-1.30856122
L3MBTL4	-4.44524105	-1.72853288	-1.04057512	-0.12389826	-1.84450144	1.014048608	-1.14970041
MYADM	-4.44490053	-3.37710614	-1.8186375	-2.17974902	-1.52903938	-3.05288192	-8.84306164
LMO3	-4.43945058	-0.33908739	-1.71518011	0.167737928	-0.53988364	-4.96986189	-6.0592025
NCKAP5	-4.43424287	-6.32493018	-4.60589784	-2.81072424	-7.07705585	-5.02003001	-1.8765556
ADAMTS8	-4.43229628	-3.76337217	-1.82057314	-2.79729472	-5.25074918	-5.11669655	-3.21147956
C14ORF132	-4.43176649	-4.35628184	-2.93618697	-3.16783178	-2.59167139	-4.09496017	-4.00112128

TEKT1	-4.42968415	-1.93500223	0.351847482	0.445124345	-1.38566037	-4.45827104	-5.74240808
MSR1	-4.42840045	-3.44103782	-3.57921019	-2.32579046	-1.88978001	-3.66274669	-2.97077976
SNORD95	-4.42655529	-2.62491579	-1.88094629	-3.37919364	-0.70239505	-0.76839576	-0.46583105
HSPA2	-4.40744884	-2.28226712	-2.13465936	-1.62163612	-2.89276913	-0.50790926	-2.22411785
OLR1	-4.39778316	-3.25622192	-1.7046316	-2.66374254	-3.87251618	-3.93167168	-6.73698171
KLF10	-4.3956026	-3.43794661	-1.7133315	-2.05880171	-0.39078713	-1.14157428	1.306027281
ALCAM	-4.39295557	-1.35315589	-0.08946349	-0.37445286	0.36730625	-1.00598999	-1.73132048
S100A3	-4.38944543	-8.37596582	-3.35727119	-2.90912114	0.200862534	-1.19942884	-2.0085522
RERG	-4.37222021	-2.86745631	-3.0660254	-2.92468416	-1.38008384	-5.05921718	-4.07763236
SCARNA12	-4.36993043	-0.70191669	1.34982371	-0.68577804	1.059783084	0.965065686	0.103480549
SMR3B	-4.36982093	-2.51237812	1.847485755	-0.06885749	0.371882441	-0.42349694	0.204025654
ABHD5	-4.36936614	-2.90767105	-1.9350721	-1.66257389	-0.17239046	-0.99638748	-3.29354332
INMT	-4.36468372	-4.13090542	-4.4917688	-2.5770271	-5.82683429	-7.90942225	-8.24182736
S1PR1	-4.35896195	-4.94967098	-2.43784549	-2.59213185	-2.96121322	-3.59353634	-1.26682299
WDR89	-4.35446418	-1.79247705	-1.17553285	-0.5039676	-1.87293945	0.427290313	-4.36354831
RCAN1	-4.34996427	-4.1781626	-0.53669162	-0.03287823	-3.2890753	-1.60045336	1.237843645
UHRF2	-4.34981741	-0.98311458	0.495643574	-1.7993253	1.541070998	0.419936694	0.220165978
PDE8B	-4.34068541	-4.1185855	-1.43136213	-2.97118471	-5.01671686	-7.8739522	-3.42187417
RSPO3	-4.33875985	-4.24769808	-0.05847585	-2.60406487	-2.35308428	-0.83740572	5.693916242
IRX2	-4.33391325	-0.03032763	-1.17577508	-2.51057353	-0.35713551	-2.56601024	-2.3859087
PDK4	-4.33146313	-3.88251632	-2.75435843	-2.2402089	-5.37338855	-6.09243218	0.095841001
ELK3	-4.32667582	-2.15038315	0.954687386	1.359497091	-0.11497402	4.237993613	-0.6285888
GAS1	-4.32448823	-4.91510508	-0.49615698	-2.68539237	-0.02141516	-0.58661229	-5.18587987
EHHADH	-4.32246489	-1.59660787	-1.22227681	0.189194882	-0.55531881	0.649277632	-0.63753005
GRM7	-4.32243075	-1.22472326	-0.09699676	-0.80122064	-3.76739778	-0.10415227	-0.82007702
GPR161	-4.32085014	-2.19441902	-0.4608448	-2.12370334	0.606096826	1.06999334	-1.12002446
ELAC1	-4.31885611	-2.12166138	-0.09664702	-1.11763128	-1.20732616	-0.68213767	-2.5618007
PPP1R12B	-4.31672997	-2.77755426	1.239629412	-0.69010857	-0.28304436	-0.1809511	-1.17737175
EDNRB	-4.31551211	-4.14874124	-3.62791765	-2.76851219	-5.48695975	-4.53205745	-1.13330174
HS.552087	-4.31471546	-4.3999135	-2.81222937	-2.43396416	-3.21754753	-4.38472761	-2.95685497
C8ORF13	-4.31135596	-0.21538089	-1.91824996	1.200808177	-1.37629159	-4.20388487	-0.89163191
RPL10	-4.30848131	-0.6716657	0.54987423	2.069223934	-0.1038887	-0.4592965	2.383444996
FBXO7	-4.3045653	-0.49967633	-0.21576043	-0.12062403	0.493551523	-3.73571463	-5.31870568
LOC10012923	-4.30443607	-2.15445505	-5.96878796	-0.96420142	-1.67541565	-0.76953971	-1.14751612
KLF10	-4.30374119	-4.3846063	-0.88092245	-1.29578692	-0.90697131	-0.65720449	-0.1532219
CACNA2D2	-4.29849209	-1.4458225	-3.5570073	-1.37971981	-5.47339927	-5.08847967	-6.04606565
USP53	-4.29356585	-1.88754712	-1.64236157	-1.98585905	-0.12044493	-1.37699315	2.080582802
IQCA1	-4.29166863	-1.47957775	-0.13411121	-0.24276739	-0.85199127	-1.86920071	-1.24866424
CDKN2B	-4.29048916	-2.41506483	-2.22561418	-2.2789523	-3.23956572	-1.51473333	-2.64110121
NBEA	-4.29010408	-2.81719143	0.931785764	-0.34667778	-0.31915649	-2.70416809	-3.36563091
C20ORF46	-4.28119813	-5.61964692	-3.78423587	-3.16852898	-5.71720169	-4.79518366	-2.67337804
CXCR7	-4.28039372	-3.67373129	-0.38564131	-1.29922566	-0.96625424	3.463615905	2.227391952
CYBRD1	-4.27874558	-1.60968292	-2.6858276	-2.98523548	-2.60977302	-2.49633982	-4.09853853
EML1	-4.27254501	-3.24190318	-0.9459345	-1.65068709	-1.6113875	0.100950602	-0.95717171
NUDT11	-4.26817121	-2.72147911	-1.90485391	-1.02451444	1.468609372	3.885104965	0.34035197
TMEM18	-4.2638052	-2.22382559	-0.72053368	-0.45015811	-1.23999802	-0.03712477	-1.51056395

HSPB6	-4.26296212	-4.32516051	-2.05556752	-3.39636441	-3.51655678	-10.1456686	-3.89549007
C4ORF31	-4.25797108	-1.81959023	-2.36008342	-1.52652324	-1.63398771	-4.61575863	-4.63933852
FAM86B1	-4.25505873	-0.85947877	-0.44223062	1.363604919	0.60268448	-0.0784925	-0.37923135
FLJ41047	-4.24965122	-0.85905706	3.018179399	0.552747725	-1.15466777	-0.53061996	-0.33327749
MFAP5	-4.24911926	-3.90555577	-1.49255555	-1.12731642	1.992862137	-1.03965641	1.24068938
MS4A7	-4.23641484	-3.96414107	-2.17628333	-3.59240116	-4.26585059	-2.92318103	-5.38999407
GRIP2	-4.23564105	-2.27248081	-1.04799133	0.411570263	-0.42316704	0.318791332	-3.78559828
HS.167721	-4.23197107	-1.12879981	-2.3849902	-0.95598363	-1.96304423	-2.33541116	-0.38814521
HS.21177	-4.23013341	-1.47333418	-1.75731462	-2.31803322	-1.11616791	-0.21415763	0.584334577
CPEB4	-4.2263918	-3.15152738	-0.08878535	-2.14447665	-0.58706861	-2.28917041	-2.44498517
LOC85389	-4.22562184	-3.5630734	-0.05581485	-0.8813656	-0.25965995	-0.62893303	2.230465139
HS.509165	-4.22132654	-0.98328221	-1.13101847	-0.25779317	0.275465411	-6.05225383	-6.20376356
RBMXL2	-4.21063154	-0.69334169	-1.67852505	-2.22427976	7.106466705	0.595429196	-0.00208119
MUC12	-4.21007137	-1.20495151	0.340493048	1.829365654	0.751649964	-2.54909155	0.44634885
LOC10012974	-4.20813171	5.690299154	-0.37098192	0.687398775	0.935120325	-2.35499677	0.977301617
SNX24	-4.20540541	-2.75807012	-1.70918783	-0.79291037	-0.74185201	-0.87717808	-1.9134544
LOC644172	-4.20232324	6.251872835	0.855885188	-0.9378088	-0.17408599	0.057483765	-3.24108388
SUSD2	-4.19869929	0.115315407	-4.42539674	-2.29153915	-4.57669426	-4.13875509	-6.03100148
FAM69B	-4.19805729	-2.80141037	-1.9035892	-1.36117418	-0.7210499	0.35833741	-0.35999825
LOC647150	-4.19627915	-1.80356375	2.261935859	0.01390832	1.056156912	1.239990345	3.319353063
LEPREL1	-4.19176281	0.385321149	-4.11192728	-1.9312623	-2.69367829	-1.62494658	-2.78651875
AGPAT4	-4.19169246	-3.91796753	-1.37807169	-1.7134107	-0.45020034	-1.9495777	-1.02527354
ADAM10	-4.19028365	-3.15017964	0.124965251	-0.43923955	-1.20884198	-1.33041976	-0.69415942
HK1	-4.18851833	-1.61378185	-0.13886217	-0.17711528	1.08049126	1.844009996	0.360766087
SCEL	-4.18558567	-2.27901725	-2.28885368	0.786765021	0.338305543	-3.64244833	-1.00504367
HS.200085	-4.18466922	-2.75885038	-1.43561608	-12.0557725	-0.65098427	-0.95093074	-0.51319148
ZNF491	-4.18134509	-1.84119077	0.053877485	-0.29136987	-1.56401934	-2.45853788	-4.35528396
TCF7L1	-4.18023008	-1.10945291	-0.84062246	-1.34126956	-1.19902992	-1.56793617	1.072175439
CXCL12	-4.17484959	-3.47434509	-0.99935886	-2.41423156	-0.92696262	-1.56815496	-2.89574303
MOV10L1	-4.17461994	1.103202372	-0.94545554	-2.00440713	2.514771106	-3.4474047	1.072439467
SFTPD	-4.17039684	-0.94711417	-5.6249588	-0.57407578	-5.30010047	-3.11983288	-7.83050279
LOC652644	-4.15482634	-1.5414496	0.402158135	-0.30671055	0.322215137	-1.55543877	1.422336259
ANGPT1	-4.15458579	-4.0324167	-3.1050986	-2.79320858	-3.64713864	-3.11834516	-1.68101873
SERTAD4	-4.15292583	0.226114826	0.869829753	1.121376584	1.653618985	1.08347275	-2.03834804
MSRB3	-4.14525325	-3.60483022	-1.71254262	-2.88627394	-1.55767198	-2.04200932	-0.87680298
PSMD9	-4.13963633	-1.51337164	-0.84209117	-2.91962656	-0.62051176	-2.27651214	-2.09366401
FAM105A	-4.13667203	-2.62260987	-1.56643089	-1.48688047	-2.33685125	-3.59614955	-2.63854385
CPM	-4.13578797	-2.42782641	-1.23588548	-1.1572671	-2.69804055	-3.40326739	-2.97667988
ZBTB16	-4.13488935	-4.12206834	-3.79019824	-2.37471144	-4.20844066	-4.74138566	-4.51872061
CYBRD1	-4.13429443	-2.02178949	-2.22651931	-3.00206688	-2.73550582	-2.45051888	-3.80890237
VAX2	-4.12629745	-1.19539695	-0.19527375	0.391029155	-2.30450611	0.520719284	0.26596924
CAV2	-4.12387063	-3.97865247	-3.29332129	-2.80123841	-4.50067731	-2.71551959	-2.9955378
CCDC74B	-4.12357364	-2.41109021	-0.07684194	-0.05952544	0.401258142	1.462807419	-4.38974738
LAMP3	-4.12248915	-4.59666887	-2.3361677	-2.26716343	-5.94913642	-2.86414182	-5.83692672
PRX	-4.12040187	-4.45182507	-3.95177193	-2.19240816	-3.89194004	-3.95273588	-2.41199468
CLDN16	-4.11854447	-2.81705537	-0.1105606	1.139274144	0.063831834	0.473217098	-0.88801327

ZNF331	-4.11084411	-1.7779302	-1.04958662	-0.36372823	-1.0200524	-1.24773889	0.024444766
NAPSA	-4.10766554	-0.01670595	-0.33940225	0.025998784	0.130916939	-3.22784657	-5.75697251
GDF10	-4.0969594	-3.41836912	-3.00200271	-2.58293026	-3.78669472	-4.85274314	1.111649055
ZNF167	-4.09292719	-0.61687924	-2.89814861	-0.18456917	1.033848939	0.436652368	-2.40057577
BPTF	-4.09267399	-3.12345616	-2.52203913	-1.94963085	-0.15819789	-1.65350945	-1.91420786
PKD2	-4.0844128	-2.08398219	-0.80264362	-2.50417604	-0.82993609	0.228999313	-1.58863465
LOC144383	-4.07869568	-0.26330399	-0.41108343	-1.51885134	-0.75367019	0.857025995	1.433664967
LOC341965	-4.07750811	-1.43104403	-0.13370764	0.935102261	1.166874281	0.875959101	0.067759584
ALOX15B	-4.07569635	-3.73658903	-3.06216493	-1.51900135	-1.41431169	-4.00911104	-3.02704916
ECHDC1	-4.07523405	-1.90854381	-1.94992433	-2.02997536	-1.55977932	-1.13641565	-6.07605614
C12ORF59	-4.0672651	-3.36829654	1.495131329	-1.35311221	-0.46542695	-0.25976155	-0.84519797
TMEM128	-4.06085341	0.513783442	-0.58682112	0.511970747	-0.63906	-1.18400849	-0.60135286
SMTNL2	-4.05991132	-5.30115982	-0.27016494	-0.35412126	0.142716073	-2.28745506	-0.03941391
ADARB1	-4.05973009	-3.37579058	-3.34525718	-2.69442918	-2.52180174	-3.91314157	0.341152943
AOC3	-4.05959653	-4.64443702	-3.65820056	-2.7984648	-3.8802609	-5.74970851	-2.95614734
C9ORF72	-4.05600421	-2.71860258	0.184709308	-2.78788223	-0.50151487	-0.0536879	-1.40493814
COL23A1	-4.05484195	-1.54794398	-0.44472031	-0.44928512	-0.77410029	0.343399348	-0.9926928
RGMB	-4.0540221	-0.68851785	-1.02697938	-0.79991505	-0.87390551	-0.74402878	-1.57004482
FLJ35767	-4.05381794	5.301533673	0.149811571	0.575115706	0.663624108	-0.38292617	1.07486885
HS.567192	-4.05355128	-2.55139651	-0.82198227	-2.11537241	-1.41018547	2.004085442	-0.45368726
SGCE	-4.05076413	-3.69378945	-1.74950852	-2.70335452	-0.80312859	-0.9072395	-3.10068373
KLF4	-4.04112779	-5.6639975	-2.03411951	-3.04716465	-2.65706194	-2.61937342	-2.22291268
PMP22	-4.03175979	-3.43427562	-1.96497888	-0.24920453	-1.51799515	-3.21408004	-1.12567796
CLASP2	-4.03166593	-2.85778862	-0.87451198	-1.59710862	-0.16600847	-3.359991	-0.72405943
ECM2	-4.03035026	-3.30357474	-2.10289743	-3.72555074	-2.61434806	-2.96824972	-5.20062466
ABI3BP	-4.02936843	-3.95069585	-2.30646223	-3.33017532	-5.18854524	-4.46315192	-4.32571259
GPATCH4	-4.02863747	-0.87096813	1.274537743	2.133045088	2.694444216	0.858327964	0.696952518
C9ORF61	-4.02291425	-2.70869378	-2.89486216	-2.35886424	-3.70336298	-5.17411938	-3.1332848
BRD4	-4.02052859	-1.53751818	-0.33881138	-1.27050572	-0.06167281	0.014641317	-0.00384823
CD55	-4.0184217	-2.43917891	-0.63235923	0.213309775	-1.64207105	-2.80586976	-0.34985325
GPC4	-4.00928374	-0.22291033	-0.10676371	-0.64564924	0.540288564	-0.77004321	-0.92063328
SPARCL1	-4.00845086	-3.16537169	-1.44566232	-2.69105029	-2.81183933	-1.8898369	-3.81748256
MRPS36	-4.00515917	-1.84997554	-0.84562259	-0.08964073	-8.48231766	-2.3655187	-8.06763293
C14ORF104	-4.00139456	-1.40592593	1.071424885	-0.24989119	0.644245964	0.949705938	0.300180823
ZMAT1	-3.99699161	-4.73920135	-0.84351561	-0.84349344	-3.86768851	-3.11941609	-4.56688325
CNNM4	-3.99427536	0.161925167	1.057192609	-0.26280933	0.10303496	1.152484358	-0.40795111
LOC730323	-3.99387049	-0.74731659	-3.98471773	-1.45519664	-2.5290276	-3.57441033	-1.34335895
ALOX15B	-3.99320647	-2.54464785	-4.98130504	-1.23834603	-0.86649336	-3.25454584	-2.70265128
PMS2L4	-3.9912654	-0.98927073	-3.63463948	-1.31404043	-2.61043425	-3.4801403	-1.46888443
CCL23	-3.99097352	-2.4741402	-2.99515649	-1.50876841	-4.78561768	-4.59853549	-6.58769421
RBM18	-3.98181652	-2.65608392	-2.43832537	-1.10147231	2.760822883	2.018126382	-0.1068946
TREM1	-3.98034912	-2.53346366	-2.47882792	-2.40660154	-0.59184547	-5.01149556	-3.77702523
ENPP5	-3.97902327	-0.33970318	0.692549879	-0.77425503	0.113578527	-5.31528789	-2.81430481
GPM6A	-3.96744271	-4.10403211	-3.74468465	-1.853965	-3.05270849	-4.28609761	-1.32252955
ELOVL5	-3.96571737	-1.488367	-0.16089835	-1.2424885	0.058299743	-0.03416174	-1.59449237
FAM114A1	-3.95587324	-1.92759462	0.051387991	0.205351852	2.076120272	-0.03998975	-1.74311287

TMX3	-3.95421849	-1.14902149	-0.87803636	-2.17806814	-0.65088633	-0.96745424	-0.8133111
MATN2	-3.94949706	-3.109176	-0.61334125	-1.057211	-0.66565741	1.80608418	-1.79655167
MEOX1	-3.94870839	-1.27926507	3.117325344	-0.45052283	1.498875954	1.092928747	-2.8401576
CITED2	-3.94854237	-2.42417749	-1.15298817	-1.39116021	-2.42458798	-3.45754177	-1.82924029
NR3C1	-3.94585067	-3.91250932	-1.20299448	-1.37358104	-1.83524235	-1.018588	-2.87413031
C7ORF64	-3.94235969	-0.13299546	0.644217293	0.156962715	0.692492173	-0.72934177	0.623226692
TCF21	-3.94180744	-4.04992827	-3.81382975	-2.75739747	-4.68157947	-5.22349997	-3.3759856
CCDC94	-3.93831376	-1.05241651	-1.07229468	-0.12147906	-1.25902347	-0.41565396	-1.34431582
LOC642428	-3.9353422	-4.43748717	-0.87514703	-0.91940808	-3.06930497	-2.27374363	-3.63872418
LEPR	-3.93498711	-4.49533999	-2.32493169	-4.29292272	-0.94437746	-4.9335032	0.710286123
EDG1	-3.92741695	-3.67380587	-1.95150272	-2.35984374	-2.9391963	-2.95962971	-0.69270262
RG9MTD1	-3.92576717	-1.84363553	-1.64448918	-0.14072434	0.128176417	1.444787193	-0.21847215
CYTSB	-3.92288916	-2.04138437	0.564199673	0.719480655	0.843263786	0.221087154	1.26668476
LOC728866	-3.91826159	-0.98880067	1.859825933	1.488994301	1.017834091	-0.84029126	-0.3374355
TRIM13	-3.91518982	-0.72562109	-0.58988313	-1.43470593	1.220259118	-1.70413829	-1.56818852
LOC729372	-3.91121263	-1.72015529	0.773839763	1.464312356	0.143841944	-1.74120634	-0.43861585
ABCG1	-3.90629202	-1.88613093	1.068415457	-1.22740577	-0.4422969	-0.75282523	-0.0998411
LOC10013173	-3.90626485	-2.40106007	-1.5635692	0.804472692	-0.04501924	0.422130226	0.19361025
LOC284964	-3.90377445	-6.16237286	-0.95695873	-1.01270829	-4.03333602	-3.61107203	-4.95051657
LEPR	-3.89391009	-4.12093066	-2.41989757	-2.72211117	-2.10260759	-4.91703986	-5.48549367
KIF26A	-3.89096005	-3.29581433	-1.53991349	0.562867158	-1.5656834	-0.36454425	-4.73418756
TRPC4	-3.88822012	-2.99940248	0.013630459	0.825711105	-0.28818123	-0.24863656	-0.10198027
LOC652519	-3.88788386	2.215856339	-1.21533029	0.754760946	-0.22295758	-1.03186275	-0.99143434
FGF9	-3.88470893	-0.63952558	1.339154416	-1.32189267	2.276092377	-0.47862143	-0.76994881
SCNN1B	-3.88119361	0.014077473	-1.7172615	-0.95365951	-1.78544751	-3.09877831	-2.77038784
ESD	-3.8807106	-1.41269388	-1.30354887	-0.83364572	0.098193666	-1.85550775	-2.16902409
CGNL1	-3.87537909	-1.72401626	-2.21220415	-2.13598864	-3.15306689	-3.43098928	-5.65713683
HS.551128	-3.8734088	-5.52617532	-2.13431519	-1.67111919	-1.7763568	1.140514671	-1.75715877
EPB41L3	-3.86876049	-3.33050717	-1.63961668	-0.9166395	-1.90887308	1.669983357	-1.71210922
LOC728509	-3.8652158	-1.43423379	-2.78432384	2.346600749	3.524847804	0.065299163	3.424061503
ADH1A	-3.86264071	-3.80099052	-1.05569206	-0.94805273	-5.69010172	-3.67275644	-5.37187166
DUSP1	-3.86119801	-3.00283315	-0.86596572	-1.11278914	-2.9786599	-2.18585916	-0.31314419
SLC34A2	-3.86026218	-0.4342199	-0.04717108	-0.61486774	0.810177442	-4.00448163	-3.96208287
NR4A2	-3.84510772	-3.66538293	1.66818243	1.03948825	-2.60421006	-1.2465346	3.753194863
MAL	-3.83772762	-4.4443216	-2.27555265	-3.04637492	-4.78187978	-4.11487692	-2.85032281
CASP7	-3.83403086	-2.27952765	-0.22445132	0.371384495	-1.0975515	2.268921803	0.102610287
LOC729960	-3.83168892	-0.11909517	-0.96515481	0.372834575	2.795520158	-0.73682022	-2.14923374
HS.527657	-3.8285933	-2.17778644	-0.54546488	-1.15193279	-1.07828479	-0.39891851	1.375163075
SORBS2	-3.82858287	-1.97998145	-0.17425568	1.018739123	0.79549125	-4.22819568	-2.43690893
FZD6	-3.82749657	-1.87933887	-0.25135073	-1.2790912	-1.25867829	1.263602269	-2.07727163
ATOH8	-3.8270579	-2.39639106	-3.38639345	-0.6747494	-3.49156368	-5.11102586	-3.40123857
ITPR1	-3.82311245	-2.4093437	-0.33478587	-1.55705428	-0.76859149	-1.81637586	-0.70422268
CADM1	-3.82163523	-1.67080577	-2.96270924	-2.23546952	-1.58550668	-4.44111821	-4.80492204
INTS2	-3.80986529	-2.33044232	-0.13965983	-0.88898672	-0.50684137	0.038656115	-0.05467715
DPT	-3.80658619	-2.46118161	-3.4675292	-3.17131875	-1.19347825	-1.9841126	-2.60728332
OSCAR	-3.8062112	-2.34805963	-2.87431375	-2.58082577	-1.44662641	-4.10946505	-6.19828869

ULK4	-3.80353099	-1.34820748	-0.50994162	-0.00244092	-4.35533171	-2.37028394	-0.81691335
RASD1	-3.79869563	-3.71146771	0.160822496	2.08248406	2.827803384	-1.82383732	5.619555863
HS.578850	-3.79566001	-0.81459639	-0.21481451	0.985552114	-1.2779261	-0.97623392	-1.86047302
TUSC3	-3.79168562	-0.08787994	0.235917474	-2.28424829	0.488334862	0.980482874	-0.47644127
PRODH	-3.79153003	-0.06526066	1.258283373	-0.81574234	1.023488035	-2.98110193	-0.79978887
EID1	-3.79079711	-2.66539234	-0.57626773	-1.26843276	-1.55798647	-2.44038542	-1.99748805
PIWIL2	-3.78909308	-2.15769521	-0.12408086	-0.17886066	0.518392079	-0.47228293	-0.51507288
C14ORF65	-3.78711739	-0.88856842	-0.9389365	-0.40561478	1.023683187	0.477067532	-1.72304526
CD68	-3.78710669	-2.82133858	-1.59820984	-1.93124776	-1.16673764	-1.66667107	-2.55642667
PPP2CB	-3.78642778	-2.80199835	-0.9171748	-1.05686425	-1.69755756	-1.10308626	-0.98534541
PKD2	-3.78341274	-2.80638481	-0.18631811	-0.77319509	-0.07498558	0.49912011	-0.30621725
PDLIM3	-3.78328396	-3.45590807	-0.66829965	-2.74164027	-0.74999871	0.264435582	-1.53027361
ACOT1	-3.78195106	-0.57957293	-0.82069773	0.119442819	-0.93253427	-5.76933006	-3.29570739
CYP2U1	-3.78135254	-1.97621494	0.504193249	-1.49977575	-0.94907082	-1.30416012	-1.56123196
MSRB3	-3.77935763	-3.59400857	-1.93354495	-2.04089873	-1.45800124	-1.89559679	-1.46517043
CLEC3B	-3.77890856	-4.79835556	-4.76222736	-2.82674851	-5.48923021	-4.96992728	-4.48521207
LOC646173	-3.77848902	-5.92414485	-0.7025526	-1.11305849	-3.0232157	-2.67028469	-4.51922871
DAZ2	-3.77648365	-0.15304758	0.471290898	0.121217058	1.315369885	-0.42545758	1.587779842
CA2	-3.77117796	-3.52746802	-3.75445134	-2.41295564	-3.58607627	-0.31280432	-1.24760507
PHLDB2	-3.77049633	-2.78307634	-1.2025591	-1.70153388	-0.37910634	0.834688607	0.412090971
LARP6	-3.76820721	-3.22266339	0.195292201	-3.58501181	-0.52891576	1.067372733	1.241210008
C2ORF67	-3.76714093	-0.8057502	0.583146208	-0.80105654	-0.43172059	-0.28995611	-2.29743654
SFTPA1	-3.76646888	-4.35821257	-0.71552711	-0.72690127	-3.24767798	-2.89896442	-4.14878384
LOC10014460	-3.76558979	-2.29081823	-2.30704616	0.8209147	-4.29314457	-2.79216356	-5.19414168
HS.540341	-3.76408224	-3.49023062	-0.68300732	0.704505589	-0.6069537	0.079280055	0.745588408
CREB5	-3.75527218	-1.09117638	-1.59036115	-1.63736735	2.09437649	-0.20629355	-0.35350117
OSCAR	-3.75390018	-2.41236901	-3.03484475	-1.79405331	-1.18799434	-3.46479347	-3.86382545
ANKRD47	-3.75185856	-3.90057259	-3.04346301	-1.49158678	-3.63073649	-1.33099308	-2.2207671
EFEMP1	-3.74547081	-3.95672787	-1.43747587	-2.7240028	-2.72511926	-3.46805899	-4.51482093
TEK	-3.73925775	-4.2145642	-3.40662169	-3.11704344	-3.70761272	-3.58629036	-3.83398206
FAM123A	-3.73919217	2.541035564	-5.2124296	-1.26374747	-2.19236668	0.189433356	-2.6144965
AK5	-3.73874829	-0.63627036	1.117207673	-0.11848262	-0.1454825	0.046581051	2.877823453
CDKN2B	-3.73775244	-2.59711388	-2.12041407	-1.45502223	-3.11068572	-1.59811209	-1.52312212
PIK3C2A	-3.73549459	-2.02647351	-0.87481256	-1.86776738	-0.45595234	-0.70027134	-0.95733289
HS.436863	-3.73426711	-3.15905564	-1.21796337	-2.91464078	-1.84221583	-2.66092798	-1.46988781
ABCC4	-3.73206917	-1.57041177	-0.08718728	-3.1400742	-0.58445419	-0.66713353	-2.89156154
MAD1L1	-3.72657192	-0.35361134	-0.23110474	-0.87734102	3.053828902	-1.35399625	1.72729381
COL4A5	-3.72533541	-3.86502507	-0.68514769	-0.194259	-1.33111337	1.129950917	1.290791001
TSPAN7	-3.72462557	-2.88702632	-3.2459434	-2.40455868	-3.27768642	1.106574924	-2.37144863
OMD	-3.72211553	-2.35002144	-1.01518666	-2.1264456	0.736124125	-2.0878014	-0.58013757
KHDRBS3	-3.71918395	-1.42419232	-0.50556904	-0.57762146	-0.31550278	-2.35074642	-1.14554588
MPV17L	-3.71694359	0.001317972	0.423193318	-1.34553061	1.374334309	-2.23166033	0.853648351
SLAIN1	-3.71351644	-0.88977598	0.616403601	-1.65321589	-0.41066535	-2.52973185	-0.63394514
NEXN	-3.71235542	-2.75685251	-2.00560043	-1.63141457	-2.301455	-1.39938773	-2.34408773
SDCBP	-3.70598516	-1.84261998	-0.55354427	-1.69366238	0.01760897	-1.36809241	-1.95609152
MYH10	-3.70287781	-2.65678228	-2.43307675	-2.73602215	-1.88539807	-1.23017843	-0.95386649

ATRX	-3.699568	-2.65791066	-0.82611324	-0.07207054	-0.17960077	-0.78501239	-0.2670867
IRX3	-3.69060387	-2.0113843	-0.49411373	-1.15831783	-0.42540824	-2.14737	-3.64650965
AIF1L	-3.69009844	-3.00254894	-1.68511038	-0.17179051	-1.67908063	-0.11814982	-0.64798968
TSPAN7	-3.68963529	-2.43625067	-3.29371925	-2.81743464	-3.69954344	1.109020264	-2.67936179
FMO2	-3.68752007	-4.32087161	-2.8119758	-2.49412322	-5.60595995	-5.04665788	-3.49911053
SETD4	-3.6839855	0.192620055	-0.12840999	-0.17747764	0.981316437	0.669939439	-0.86239268
ABCA8	-3.6822724	-4.55730626	-1.98429852	-2.91107127	-5.50163946	-7.98978819	-2.5406493
NFE2L2	-3.6742548	-2.27045032	-0.02049238	-0.82609875	-0.48700715	1.521027151	-0.12113378
CAT	-3.66983539	-3.14109391	-1.34676843	-1.96996139	-2.79725089	-1.76339778	-3.02326354
VMO1	-3.66886948	-1.47247563	-0.07473314	-2.34525035	-1.1199906	-2.94944666	-5.33626811
ADRB2	-3.66554801	-3.5059976	-2.17791529	-1.87030598	-4.42473453	-2.53100872	-4.39391757
LOC731751	-3.66479295	-1.74582151	0.636257957	0.4194819	0.49997329	1.377059932	0.195857355
SOX6	-3.66265434	-2.19387743	0.339657544	2.622636258	-1.49040484	1.181291186	-2.41338198
LOC282992	-3.65928855	-1.10718496	0.543009447	4.219015262	-0.52076411	0.582794721	1.535995243
FAM126B	-3.65860258	-2.22947796	0.554079862	-0.49097095	-0.13431956	1.155075989	0.018266429
HSPB8	-3.65679157	-3.53976499	-2.86308228	-1.71501673	-3.42992933	-3.43579919	-1.78073453
USP13	-3.65677535	0.064314322	-0.9425113	-0.13901435	-1.27164314	-1.48425191	-2.10426784
LRRN3	-3.6558891	-4.53436586	-4.75023074	-4.35375449	-3.7175693	-4.80196104	-1.60055026
SPOCK1	-3.65449969	-0.68893905	0.993420906	-0.51103223	5.045948302	2.352205189	0.566354741
ZNF416	-3.65405894	-0.86720274	-1.67758278	-1.06184149	0.717353599	-0.58929649	-1.16913064
LOC10013418	-3.65289938	-3.28396658	-0.91926489	-0.42913993	-0.34241166	1.783623318	-1.90431789
PCDHB2	-3.65226858	-1.2567147	0.234781045	-2.65052202	2.142805786	-2.12820309	3.678905051
ZSCAN5A	-3.65225697	-3.03379117	-0.09254759	-1.29246278	-2.25516338	0.285922294	1.890512893
COL13A1	-3.64941118	-3.59015775	-2.78916753	-1.50493113	-1.45908972	-3.6820508	-3.16513449
KIAA1984	-3.64795772	-1.4365622	-0.34440023	-0.90776932	-1.30777547	-0.54960989	2.248874356
GABBR1	-3.64331758	-1.0091136	-2.59299823	-0.85525646	-0.57389909	0.130596038	-1.29718505
CTSC	-3.63985566	-1.43185924	0.027983499	-1.53418426	0.933939458	-0.10015818	-2.44046341
RGN	-3.63932932	-2.61380856	-2.58525971	-1.48267875	-1.21436947	-2.88493032	-0.47363562
RASSF2	-3.63573185	-2.1609443	-1.49349806	-0.62165599	-0.80715884	-1.60711165	-1.066026
TANC2	-3.63424529	-2.17152982	0.569211089	0.762105073	0.80795724	-0.60482529	-1.24741227
KCTD18	-3.63391119	-1.78051768	-0.29117157	-0.56519592	-0.02828671	0.329115445	-1.96786832
KCTD6	-3.63268434	-1.18291166	-0.36102824	-0.70920549	0.505853734	-1.34817113	0.288845164
PRNP	-3.63214904	-2.43039238	-0.82982799	-1.51843254	-0.60136204	0.944962047	-1.92902966
LOC255649	-3.63076513	-0.97568052	0.965210752	-1.90821491	-1.49576777	0.84380537	0.925273987
RORA	-3.62993517	-5.40821986	-1.82307317	-1.70043269	-2.47328627	-1.34415237	-2.00239661
LDOC1	-3.62977614	0.180315665	-0.26876585	0.093251497	1.017001072	-2.74593837	0.818336627
COL12A1	-3.62777503	-2.24683959	0.065774487	-2.64433364	1.58554205	0.754358393	-2.5385736
C1ORF99	-3.62725359	4.148857678	0.619474534	-0.15928902	0.385113144	0.094491721	-0.57614023
LOC652630	-3.62722564	-2.00743623	2.274499179	0.785811385	-3.30324829	-0.58214812	1.237673647
SNX10	-3.62423386	-3.55308836	-1.63641016	-2.69483648	-1.38430524	-1.17254326	-2.9949781
PSIP1	-3.62296937	-3.46637714	-1.15946187	-2.28197034	-0.29295019	0.965089278	-6.08681352
ITGA8	-3.61656553	-3.79709464	-2.06896734	-2.47999702	-1.92197671	-4.85737666	-3.51887421
CPAMD8	-3.60499818	-1.89060927	-2.65567711	-1.86443679	-1.64529166	-1.22980383	-1.23551787
ANKRD20A1	-3.60214447	-3.29652517	-3.04997448	-1.98173922	-3.23763301	-1.60631248	-2.46458578
NCAM1	-3.5984661	-2.1662317	-0.68413282	-1.8933251	-1.30706039	-4.75421189	-3.72471977

Supplementary Table S3. 500-upregulated genes above LTT36 data in 7 LTTs of NSCLC patients

Gene Symbol	Lung Tumor Tissue (LTT) vs. matched Lung Normal Tissue (LNT)						
	LTT36	LTT21	LTT33	LTT43	LTT42	LTT47	LTT08
GPX2	9.100938754	2.915810786	4.943764547	1.503770889	5.592049258	8.599964224	7.962866552
NIPAL1	7.906296069	0.178889241	-0.38354505	0.921784691	-0.01553964	1.617280704	1.079769612
LOC653060	7.803925504	2.226752717	0.954290607	-0.82025918	-0.88995439	1.554481157	-3.94490862
HS.148168	7.687155317	-1.08771685	-0.86842429	-0.11541375	0.546694349	-1.06071566	0.445422494
NSL1	7.535276576	-0.2171683	-1.8534704	1.052338246	0.475895387	0.142880043	-0.08356706
GABRG3	7.446256004	0.584853843	-0.34616196	-2.1941929	-0.6897561	0.902425743	-0.83850494
ATP10B	7.408320876	2.961831918	4.790035399	2.609411862	3.369601597	2.361504504	3.213673296
FRMPD1	7.101062521	-0.57979993	0.382358472	0.667479779	0.046985233	0.885959778	1.171315028
LOC653257	7.095377129	-1.64064719	-0.46059716	-0.2968094	0.353585691	-1.37676905	-0.72619522
DYNLT3	6.980055144	1.828654994	-0.25589873	0.56270675	0.88455145	0.390611814	-0.22336236
GRIP2	6.976081109	0.198374468	0.44178977	1.970952836	0.424617754	-1.26371061	-0.93304805
HS.582886	6.745499194	1.127768085	-0.34166454	3.326352133	-0.92539577	0.07576859	0.21534048
PCDHGB2	6.700524773	1.16255738	-0.05963658	-0.71651917	0.078049935	-0.1285371	0.129191507
PTPN20	6.692825305	1.93271545	1.574266183	2.595574756	0.006313983	-0.62038068	-0.1455289
RPAP3	6.657238269	0.044449655	-0.90351884	1.803129793	0.738376938	0.706840614	-0.59312788
DUOXA2	6.654368948	-1.56903923	1.380573733	-0.43335028	-0.91340196	-3.1639938	0.784189919
PHOX2A	6.56724851	-0.02748857	-0.55590059	0.708343243	0.113492955	1.901581275	-0.13046845
C1ORF81	6.511613583	0.138472668	3.172134956	2.268784695	3.674807072	0.04841055	1.729353538
LGALS7	6.506362795	0.678096637	0.429590471	0.494545251	2.309832224	6.202289434	-0.56072623
LCN2	6.419920681	4.08964123	2.355381229	2.495633837	-0.310135	0.633382836	-0.14727502
LOC645671	6.387340173	-3.59476447	0.27695757	-0.9432658	-1.18555641	-0.2994254	2.653620415
IQGAP3	6.337922248	-0.98952495	0.369160459	-0.61434789	1.278854218	-0.21576335	-0.06001028
TEAD1	6.321220357	-0.67895895	0.411809773	-0.02155286	-0.13645078	1.223209025	-0.13115434
LOC124220	6.301495608	1.254516707	1.174124799	-0.88076751	2.735294793	0.386630113	-0.00751291
LOC653962	6.247624718	-1.39684742	-1.63926245	0.017601666	1.200305209	0.975799485	1.313577355
KIAA0692	6.235874527	-0.15525724	0.046772583	0.986897684	-0.420796	0.118665821	-3.16807737
CDH17	6.226868112	0.418767067	0.318400326	2.756534381	1.810819516	6.654452811	2.373623604
IRF7	6.14841716	-0.81418401	-1.79437404	-2.08070176	0.085466825	-0.20119902	0.831815209
ANXA10	6.057764232	-0.39857989	1.825470363	5.000048203	-0.95111542	5.792571919	4.646347343
HS.571207	6.026825403	-0.98253325	-0.93414729	0.963666243	-1.36044869	-2.00165661	0.184603901
LOC728734	5.949344693	-2.83093615	0.945689409	-1.72103482	-0.21868861	0.875460353	-0.21479629
LOC649886	5.928882382	-0.41097945	0.615456373	1.215072262	-0.0377037	0.605208589	0.107125024
LOC651524	5.881550066	1.69741398	0.924383704	-1.31660525	-1.82472955	-0.75455341	-1.13431761
MUC5AC	5.827080462	1.449541587	0.588293276	5.943881915	6.155328648	-0.66412714	7.174485368
C20ORF114	5.825361573	3.675193878	2.392967432	1.091436219	1.002016009	-3.51426238	-5.55398517
LEMD1	5.812392891	0.848480722	4.917332697	4.071225357	4.665385181	4.978403707	0.509315088
ENPP7	5.805642438	-4.17155611	-0.19827822	-0.58947246	-0.67924627	-0.90307054	-1.68341853
PRDM13	5.786572523	-0.3652326	-0.09221237	1.041724522	1.38677985	-1.930339	1.134651238
RNF17	5.77512623	-4.02893058	-0.37546244	-0.75330147	-0.08880983	-1.2444422	-0.41630194
COL17A1	5.761168068	1.595922879	2.219269452	0.750474151	5.450673084	6.559073245	4.285565345
LOC283392	5.653739504	1.751482061	2.14248673	0.945984595	-3.28606455	-0.46864354	-0.78310712
SNORD3C	5.582442935	1.241972953	0.799045578	0.290089354	-0.78372997	4.047969254	5.922325659
LOC643107	5.479899914	-2.23883917	-2.1954518	-0.71286295	-0.51202171	-1.48752513	-2.31028032

TSPAN4	5.470685558	1.040497487	1.603348057	0.535888707	0.36117033	0.499334203	-0.29160088
LOC284422	5.272229996	1.226619619	-0.42912407	-0.47310101	0.986171187	-1.61261113	-0.74066575
A1CF	5.255480332	-3.09355305	-2.68910034	-0.67979685	1.106818629	1.213591052	1.516854191
LOC10013422	5.245894463	-2.816334	-1.22395443	-1.77314243	-0.68754408	1.561810987	-0.10513778
HS.128018	5.14693859	0.068476937	-0.23998495	-0.49927649	2.34422952	-1.25386252	-2.65387951
LOC646030	5.142321645	-1.53908229	1.094568952	1.131708239	-0.75188306	-0.33595995	0.200501666
EPPK1	5.137631845	-2.01010258	-0.25393785	-0.87556809	1.31277341	0.971810804	0.053251125
BAIAP2L2	5.033596107	-0.16211304	1.676618687	-1.69082082	-1.51298206	0.093908754	4.339169878
LOC650830	5.026369858	-0.65015798	-1.97206377	-0.91575016	-1.68313059	-5.72234904	-1.77720586
MRPL4	4.986355414	-0.82588639	0.64507779	0.698189385	-1.05121994	0.852880367	-1.51119664
LOC10013285	4.975675393	-0.46866316	0.005521405	0.187413007	1.365930046	0.647460237	0.484411788
OR4C11	4.930032913	0.984128883	-0.12826849	0.260798864	-0.16050906	-3.01970521	-0.22272823
ATP13A5	4.923122912	-1.60634824	-0.48234745	0.857638725	1.218932322	0.324485755	1.086496322
CDH9	4.899404385	0.277359376	0.277295341	-0.48491561	1.333122976	-0.73510973	2.967216074
ZNF607	4.886846725	0.42104424	0.556869696	2.637019427	1.725715188	-1.18094167	-2.51840181
HS.145985	4.882395071	-3.08895964	-1.03598757	-1.4055415	-1.84273556	-1.75699864	-0.45202128
ADM2	4.769421379	1.436584657	3.297623996	2.455196568	4.834088349	3.023440487	2.413921239
HS.542001	4.755143246	-0.58982292	-0.37995515	-1.82225366	-1.5089888	-1.7841202	1.754350555
PDZK1IP1	4.749835358	0.549965218	-0.27168155	1.378954889	1.336370868	-2.02451593	1.808534135
SPDYE1	4.726703735	1.60355424	1.62817491	0.490965976	-0.06899413	-0.48466007	-0.45851537
CYFIP1	4.682153768	-2.01395224	-1.54326249	-0.30576977	0.557099703	0.066871603	0.415224367
LY6G5B	4.679896191	0.902324922	-0.15952884	-0.08575171	-0.57464118	2.175258247	0.258310031
CDCA7	4.676491171	2.853486069	1.298789755	2.728261646	3.112244588	3.131761019	3.958604662
ZNF322B	4.675467122	-1.16544403	1.056074353	0.710031408	0.773841876	-1.41667504	0.838934937
LOC728910	4.666939383	1.615353217	0.975545322	0.105295576	1.851459394	5.927228469	-0.88455858
HS.525400	4.656168324	-1.87367487	-0.44097842	-1.37954797	0.137197083	0.700114525	1.29430312
HS.214235	4.634327475	1.301009993	6.412228949	-0.01827323	0.042769477	-2.46309753	-1.81752361
SNORD3D	4.621374357	0.910890286	0.874478626	0.728774582	-0.98723731	2.567406217	5.259948548
PKIB	4.59090303	-0.29851317	0.117286736	0.52612469	4.56493405	0.127344289	3.95970294
JSRP1	4.565267474	-2.16317987	2.174643267	-0.21720384	-4.19526934	1.642265673	-0.93289393
POU2AF1	4.554480114	-1.27486862	3.099617713	2.019453114	-5.58289744	2.619725558	-0.86874823
HS.576501	4.527500536	-0.45972793	-1.19229546	-0.11762797	0.143957272	0.946082927	-0.3717651
FAM39DP	4.477735534	-0.69152604	-1.2527129	-0.23968443	1.613298888	-0.1335934	0.713239626
LOC729218	4.457059454	-0.78228523	-1.91623987	-0.50426107	-0.31081486	-1.35645208	0.413071963
LASS1	4.455573388	-1.67367786	-0.2599301	-0.51222383	-0.6861237	-0.44975222	-0.44553848
SP8	4.45202974	-3.04188256	0.813679799	-0.16162144	0.584333516	1.48737685	-0.55732465
LOC654118	4.427940411	-0.09157376	-0.18709522	1.000268469	2.46326381	0.378107367	1.347928306
CCL19	4.42230347	-0.18103156	4.670151807	-1.16874784	-5.25365364	0.647684126	1.368595978
THAP9	4.416066116	-2.50417126	1.662814451	1.575329725	2.088841064	1.252474558	0.007553914
MNX1	4.364286107	0.613200388	1.560926848	2.225036592	4.393063031	0.114987842	1.313706098
ACOT11	4.356010474	0.760779962	0.579610321	0.908924505	2.192797712	1.121167394	1.206062022
MAZ	4.335556811	-3.08683032	-0.70280895	1.721508411	-1.39522457	-2.13608473	-0.01683538
LOC647506	4.32551745	-2.35155868	0.908295344	-0.00181925	-2.33971191	1.101518484	-1.79550922
LOC642131	4.303600232	-1.89836059	-1.47620682	0.34349613	-0.39734485	0.980451106	1.384477585
LOC10013273	4.299938692	-1.61771819	-0.19791553	-0.15562197	-0.25328693	0.61434304	-0.79686015

LOC644122	4.277122085	-1.24936646	-0.5886043	-4.84361734	-3.66172299	0.555762522	0.434679487
OR13C4	4.244944732	-0.74974584	-0.87726545	-1.13933059	-0.89106592	5.907548398	0.677589977
LOC642345	4.23400371	4.273842013	2.249675161	1.289294368	-7.00968782	0.664738404	0.294985534
LOC646670	4.208626015	1.469786494	0.327707916	0.567363039	-0.9529334	-0.92566716	3.260436439
TRPM2	4.207538447	1.548072844	1.532932536	-0.37282332	1.768131484	1.543610239	-5.19054493
CER1	4.203856045	-1.44023929	-1.03332262	0.790153918	1.942343251	-2.90997129	1.502191219
ITPKA	4.203496846	-0.37751154	4.069692425	5.142566481	3.808165512	3.239743563	4.016759766
LOC730684	4.126167613	-2.26228322	-0.76173058	-0.96598335	-0.04806232	5.327831133	-1.00534715
RANBP17	4.12296562	1.028553879	0.317678423	-1.01823222	0.412168118	0.36848869	0.403115999
LLGL2	4.108610765	0.650029015	0.712843339	0.601355756	-2.1179101	-0.20987791	-0.88753156
LOC652694	4.080818034	-2.31540389	1.801401331	-0.09258513	-2.40493127	1.899311845	-0.41702102
SLC6A8	4.07709961	-1.18800383	-0.0742695	1.877907281	3.274800387	8.233507975	3.306678378
LTF	4.075043907	0.042346025	4.444592469	2.356563175	1.223795746	2.207441752	0.384411612
BCL2L15	4.064653699	2.393891902	0.29120808	3.11572613	3.030018966	-1.66510814	0.181872177
MIR1974	4.060565315	0.617538251	-0.450575	-0.48246681	-1.90634026	-0.53289103	2.015491176
MIR626	4.0494573	0.430321942	0.016021848	-1.41794543	1.485944612	-0.04484199	-0.27189528
PROM2	4.040906141	2.42692735	2.196090778	2.770357796	3.192291114	2.013363496	2.192879011
HS.282470	4.035985396	-1.28957202	-0.81067421	0.009042853	1.130553349	-0.03746754	0.338745781
MIA	4.032815183	-2.19213681	0.653760344	-0.11766798	0.626420204	-0.35279936	-1.11819169
SLAMF6	4.005063518	-1.90263679	0.105800548	-0.82035303	-0.21433974	-0.06576828	-0.31783971
PIGR	3.983264492	0.909397614	1.476271341	-0.50279214	-1.05521654	-5.17595749	-3.8754937
FCGBP	3.965949977	-0.41324715	-0.85255543	-1.54810931	2.458593219	-0.37551326	-2.51907543
INSRR	3.960724318	-0.69637149	1.972552984	-1.19089054	-0.56669635	-0.22040619	-1.38357765
MGC29506	3.946585749	-2.06696208	1.306236211	0.03383825	-2.43238676	1.036706701	-0.96521103
DNAJC12	3.945799359	-7.20465903	4.334279495	4.479233131	5.100001822	-1.41532346	0.605090075
LOC10013288	3.941051987	1.357903645	-1.70608606	-0.82560778	-0.08452665	4.774331077	-0.72636385
DKFZP434P211	3.935580615	-0.72580987	-0.94368586	-0.61228806	-0.95832974	0.466039226	-1.1506941
ZIM3	3.93476506	-0.44410009	0.032085832	0.169894118	-0.36800909	-0.50417237	0.381433914
P2RY6	3.917799707	1.361439994	1.491032944	1.481097214	1.22514501	-0.61108867	-1.05122498
SNORD3A	3.909972816	1.296682169	1.142605435	0.366271783	-0.51339801	3.434544527	5.427101828
SVOPL	3.907476664	0.527492139	-2.31938478	0.317988928	-0.08904712	-0.12269894	-0.18201386
PPP1R14D	3.903932456	1.222690827	0.476955755	3.832773307	3.751587721	1.321144406	1.747059918
PHLDA2	3.896840082	1.652660532	3.331106889	2.854887737	5.980190156	2.624763262	2.211798374
SPDEF	3.891962392	4.832554217	1.988085236	3.437250375	4.868662792	-1.01900671	2.775232711
HS.539278	3.881623982	-1.61064608	-1.33871068	-6.44633662	0.99118543	0.294349845	0.302424877
LOC649970	3.879584859	0.286112503	2.525821097	5.622375757	0.024229098	3.254746117	3.830397988
MIR20B	3.873387169	-0.67628643	0.518653246	2.555361815	2.253985081	-0.1197296	0.771081257
LOC652493	3.863762575	-2.7651234	1.549722655	0.572492107	-3.14912809	0.854768451	-1.74142594
HS.50571	3.854508944	0.235459122	-0.99980121	-1.31871332	-0.6800745	1.868437236	1.597373581
MIR506	3.854094599	-0.63853548	0.845188508	1.722918984	-0.24004136	-5.91101585	-4.90113243
NCRNA00086	3.830893874	-0.55180105	0.11999103	-1.66102124	-0.17141296	-0.10024641	-1.55913126
HS.545199	3.823129029	-1.07120774	0.364211101	-0.51473056	-0.15884267	0.508217092	-3.29314336
LOC200493	3.814234925	2.448232046	1.174328712	2.535122329	-3.29855779	-0.00827005	-3.03069448
LOC652645	3.812782666	-1.0073645	4.123297655	-0.66770772	-0.23683948	-1.15050368	-0.12081132
PKHD1	3.804316367	-0.87375537	-0.17035154	1.087423422	-0.0267879	0.58193644	-0.82812654
HKDC1	3.793369183	0.362235163	3.939820326	0.339967799	2.705942058	-2.19987458	4.643786573

LOC646056	3.785585805	-4.85333448	0.812976116	0.765862131	-2.55989183	1.509493642	-1.69740282
DIPAS	3.782361323	-0.26272292	0.125525554	-2.5900329	-0.79911471	-0.21092978	-0.25600075
LOC730086	3.781540306	-0.87449714	-0.07589535	-2.20969188	-5.53876968	-1.06349958	-1.0652619
TNFRSF6B	3.780664105	-1.68293144	1.241909883	3.101436631	2.120075453	1.579168574	1.945742737
DMD	3.776367909	0.824197266	-0.14150069	-0.00436164	-0.10813618	3.022965961	0.733057942
SULT1C2	3.774040937	-0.90782515	2.065137267	1.367149586	0.261543204	-1.71749493	-1.24585852
LOC647450	3.769506806	-2.5363021	1.699031791	-0.07112227	-2.89847503	1.010920385	-1.84335713
HS.540522	3.736335515	-0.86100123	-0.16231224	-0.4094241	0.062692112	-1.22154304	1.428603915
SPINT4	3.73029612	-2.80656706	-1.39261357	-0.13236574	0.209733227	-0.09603628	0.341697957
MARVELD3	3.727542509	-1.59346895	1.974718542	3.020157327	1.618926658	1.442262991	2.422144462
LOC642113	3.725160012	-2.733637	1.701657253	-0.87227083	-3.86170943	0.641217538	-1.52058109
LIPG	3.719408672	-1.66768819	1.25808684	0.548532272	1.119954908	-2.25686821	-1.5234654
LOC286208	3.713096274	-1.30322998	-0.14407252	0.980624792	0.565150776	1.808590419	0.140266173
PREX2	3.712252011	0.688689516	-0.40099853	-0.68173911	0.788769667	0.040270429	-0.08389446
HS.319406	3.70868804	-0.64569597	-0.30665209	-0.36933845	2.002533594	2.441274006	-0.05048413
ADAM8	3.704134483	3.537528966	1.39255582	1.518904269	4.121847953	4.191428122	2.561619782
PPAP2C	3.683463264	-0.39848273	2.081123055	0.911554488	4.723177588	2.006378566	1.859007547
ZNF585B	3.671542754	-0.78102583	-1.11545948	1.163570868	2.788578903	2.869655363	0.81484549
GPR149	3.668706633	-1.26229991	0.397452397	3.237886636	0.54554744	1.378221407	-0.38655753
EPS8L3	3.661148901	-2.34857321	-0.41331823	0.31337694	-0.81703361	-0.38573217	2.498735281
LOC652692	3.651261627	-0.93717018	1.204380176	0.980481437	-0.43416574	-2.45334163	-0.91134641
OR4K13	3.646373545	-0.58685968	1.174230905	-1.14622528	-0.21373386	-0.19397374	-0.62356875
MYO7A	3.643048536	0.837022512	1.922639204	1.373302474	5.464102792	1.656903014	2.241606847
HS.565248	3.641496881	-1.02927977	0.002789373	0.364419935	-0.8577155	-0.55449361	-1.60408171
LINCR	3.640032405	-1.82340819	0.748242442	-1.46654734	0.113873517	-0.59288532	1.434155853
TRIM54	3.638463486	-1.00242555	1.171438039	-0.4393043	-0.09053459	0.500524819	0.550256408
TEPP	3.634318192	-0.03795587	1.507142491	-0.31793169	6.189625234	0.407945395	2.070104677
LOC10012893	3.6221018	-2.53593678	2.46821664	-1.02706381	-4.57805639	-5.38209731	0.688196836
LOC643475	3.619563502	1.477961663	2.497716637	-0.63272949	1.390450016	-2.16892931	0.394962788
HS.333045	3.612539213	-0.5615695	-0.02114717	-0.2004825	-0.28345152	-0.05367413	0.710289797
FLJ10120	3.607541835	2.884961246	0.929148786	-2.89270742	0.740543508	-0.01971417	-0.31735363
CXCL5	3.599181795	-2.42281208	-0.84618704	-0.65004134	-3.75152257	-4.31035814	2.756997207
HS.576947	3.592904579	-0.23250694	-0.94377984	1.102138994	0.300626893	0.104685006	-5.25568838
LYZL6	3.586626171	-0.99441545	-1.52461152	-0.53791268	-1.45318729	0.545350534	-0.70130559
ARHGEF16	3.581090336	1.068541568	1.155037883	2.077960957	1.423343971	1.649100313	2.182321347
ASGR2	3.574321792	0.335439239	1.332430381	0.760774887	1.376201479	0.306797828	0.154500609
C1ORF106	3.569776618	0.084111191	1.096792776	2.600333501	2.509124802	1.89420692	2.362628931
OR2T2	3.568696923	-0.15729164	2.072127029	1.637348807	4.700614861	1.061514483	2.073818064
LOC645721	3.560212466	-3.53076576	-1.78255438	-1.48243803	1.985070627	0.088475865	2.699657168
ARNTL	3.542755683	-3.08722551	-0.40178082	1.647452633	-0.15179984	3.02425432	1.14597271
LOC727937	3.527262865	-1.41759031	-0.14762892	0.181817815	-1.56182544	-2.03152367	-0.15325085
MMP9	3.523116816	1.114805232	2.756863159	-2.97600054	7.580743932	3.037950951	2.51274839
SPP1	3.521753294	2.517924774	4.133379377	1.680868254	8.48400862	7.781512952	2.895811957
PAEP	3.521475705	-0.25820374	1.304198353	0.044947385	0.076187571	-5.11594792	-0.5002857
CHST4	3.521179049	-1.29803611	1.838819995	3.726337155	-1.54982912	-4.63422451	0.676453919
HS.519022	3.520606716	-1.43071719	-0.98042655	-0.7874878	1.141093419	0.283695113	1.54516302

PAGE5	3.517185006	-0.77413116	0.655579605	-0.21036968	0.203869799	-0.04764577	0.098173451
KIF2C	3.513028161	0.761306515	0.973122889	3.713896549	2.762217242	3.676446125	4.314281616
GPR110	3.509771852	1.712893643	1.305507385	0.281603388	1.06946908	-0.18345513	-1.3180024
KIAA1086	3.502582286	0.307377244	0.434751682	0.381150136	1.869067843	-0.18073833	3.254954286
ETV4	3.500610402	4.609223471	2.655796664	0.868547098	2.688190556	3.781023444	3.264086862
LYPD2	3.487384515	-1.03666004	2.087727016	1.10435348	-1.76311314	0.233575225	-1.30201136
LOC646439	3.481621942	-0.1751568	3.051915064	2.742226223	0.003272334	0.363861082	0.550606979
UNC45B	3.47695512	0.795104773	-0.10005576	-0.01202385	0.93743881	0.565314409	0.090679466
HS.576292	3.454837281	1.326070159	0.295046491	-2.32668933	-3.10287712	-0.47747408	1.07536153
FGA	3.439954307	-0.82047545	3.834988575	3.025746154	-0.22257778	-0.61672132	6.041397532
LOC654256	3.434562095	-0.04413129	1.302335548	0.337072886	1.885320031	-0.0946636	0.752149829
SLC45A2	3.428549295	-0.12663694	-0.725433	0.634239273	0.380819019	-0.44029722	-1.62159843
LOC10013151	3.422272564	-1.42327972	-1.0775627	-1.40044329	-2.12361755	1.787456868	0.36983172
PLEC1	3.419947053	-5.32687274	2.662040776	-2.50650731	-2.57185115	3.253587137	1.153532571
CXCL13	3.418853548	-1.06922898	8.956355111	0.646141617	0.807837445	3.882969761	3.66724958
MPPED2	3.418345422	1.486570355	-0.09805169	1.464304069	0.080472007	0.177808294	0.646112378
LOC653610	3.414345622	-0.39159925	3.857452417	1.92523829	0.612313514	1.786326764	4.563691895
CYP2F1	3.404883611	0.334230588	0.978941661	0.755572516	0.039594532	-0.58078901	-0.63332548
MIAT	3.401878881	-2.40638363	0.884945403	-2.99072044	1.703098988	1.652386045	0.11738249
LOC650517	3.392148397	2.231361732	1.513803109	1.956494771	0.213908553	0.670738653	1.124808095
HS.571292	3.381209241	1.304692988	-0.71413358	-0.50762656	0.095301706	0.666853577	2.240997079
KCNK12	3.363424139	-4.12941317	-0.03637526	-2.36281237	-0.20667869	0.854217349	0.608334328
DNASE1L2	3.358267235	-0.9046941	0.3451317	0.594947798	0.405255195	-1.02956484	0.717757929
SLC9A2	3.358115067	-0.76686468	-1.44758655	1.096512649	-1.83533538	0.655739977	0.109388228
ANKRD22	3.358093487	0.159131857	2.334840799	0.169950803	1.842144847	0.502976287	0.673736301
LGALS2	3.350593105	-2.38083684	-0.11793542	-2.68263462	-3.87860734	0.942392116	-2.26366218
LOC649923	3.334447673	-2.31570774	2.678290301	-0.56646019	-4.14283099	1.870863076	0.288889189
CAPN12	3.333674533	0.78020123	0.766305592	1.334168877	1.374865419	0.267437113	-0.02505999
ESPNL	3.330840192	0.316557209	-0.56144208	-1.85720839	0.705931363	0.685049981	-1.77064666
HS.580699	3.324378257	-0.1517734	-2.41483213	0.926265187	2.962533011	1.179778423	-1.69314685
RNF12	3.323500568	-1.95528528	-1.77150428	1.4646373	-1.11764754	-0.62109457	1.833015238
CYP2D7P1	3.310995164	1.108888655	0.382926716	-0.59772058	3.31499832	-0.15375782	1.72272481
LOC10012813	3.305127972	-1.42944812	4.101434084	0.025585089	-0.35568952	1.580469802	-3.15722999
CCL20	3.277751785	-2.12374368	5.461859186	-1.03069274	2.549396528	3.663424578	4.003984095
DC36	3.275064303	-1.91387337	0.118654757	-2.27193833	-0.30279107	1.95835499	-1.49050031
HS.560266	3.273310851	-0.37426789	-1.26323008	-0.84815834	-0.5774462	0.583715921	-0.81220365
SIM1	3.272092294	-0.73068163	-0.82377573	-0.75097683	0.266372073	0.325208146	-0.49116862
LOC10012813	3.271085408	-0.20688824	-0.43376879	-2.48863355	0.250465204	0.16704532	0.752090392
CAMK1G	3.268676531	-3.22174817	1.406200499	0.007063093	-2.4963231	1.34413846	-0.68070271
CCDC27	3.263484482	0.510041846	-0.1609095	0.550769092	2.237570898	3.151864791	0.37829339
LOC644853	3.256879919	-0.18521053	-0.87238424	-0.48029752	0.852750454	0.856370896	0.596724021
SLC2A1	3.256228528	-1.16886111	1.476373857	1.379481651	3.95170871	4.901190373	4.946022523
TMEM52	3.244690607	-0.44629323	0.985089383	0.234372244	-0.48375806	1.491209194	0.392784874
EIF4E1B	3.243099375	-1.45058306	-1.15451386	0.296450693	-1.99953888	-1.89026763	-1.70314577
HS.181245	3.24299126	1.368216768	1.979715006	3.171747296	2.226249824	1.662725647	2.469756351

CCDC132	3.237186056	-0.36465702	1.727995907	0.485548483	0.755401937	-0.23560926	-0.1150129
ADAM32	3.235288048	-3.95386128	-0.73223292	0.176793525	-0.01203123	2.324418268	1.081125489
KIF18B	3.235193196	-0.4863737	0.36657188	-1.67037737	-0.57566994	-1.62006273	-0.33054225
MLKL	3.216176025	-0.3474319	0.477047297	1.180227795	1.137611673	-0.39087577	0.625903227
IL15	3.211509215	-1.72178623	-1.85448393	-0.53569327	-0.87808298	-2.26483804	1.155441202
CCL28	3.21124845	1.471078159	-0.48852832	-0.1324754	2.103279595	-2.5945618	-0.72343915
GIYD2	3.20636648	-1.8168431	-0.13337824	0.22675828	0.351650755	-0.68542865	0.51914205
KRT16	3.202325408	-0.7617012	2.157168458	4.514889951	2.63431818	3.392024858	2.813043109
RARA	3.186547455	0.150606997	0.932523204	1.174293659	0.652714656	0.718037657	1.386132025
GUCY2F	3.183267111	-1.05818707	0.302829678	-0.07127861	-0.26966118	0.214785573	0.884217309
HS.552357	3.177507152	-1.07361364	-1.52341033	0.30721493	-0.79614256	0.227161925	0.008778134
HS.443067	3.17165273	-0.47119024	-0.41124594	0.304996484	-0.14733435	-0.69604614	-0.29846793
CAPN12	3.16154168	1.20122966	0.742661798	2.138097394	1.571610103	0.415860495	0.137881715
CTSE	3.156110989	0.905910603	0.767113239	2.239235149	3.492696142	-0.9763943	-3.76475353
MAGEB6B	3.14615912	-0.16916868	0.867523466	0.598612085	1.545600059	0.236893173	1.240104045
FER1L4	3.138163861	-0.92180224	0.628732832	0.358062973	2.045523384	1.390937369	1.861021274
ZNF568	3.130556933	-1.52383319	0.238567145	1.122045185	0.999044675	1.125626807	0.221424791
SNX21	3.129691404	1.694084238	-0.52436146	1.473824878	-0.29487189	0.78652997	-0.01946781
PRR15	3.11945731	0.575709228	2.413726874	1.570601795	3.317755777	-1.96903244	-0.92075785
CRKRS	3.118560034	-3.20942569	1.604983792	-0.16371342	-1.44625535	1.358968633	-2.28194538
C4BPB	3.100552453	-1.1169023	-1.22158103	3.234210255	6.270397593	-1.64550457	0.381116437
HS.545003	3.095621051	-0.90175081	-0.07526721	-0.14736863	1.535926534	0.955994384	0.177387645
IGLL3	3.092227513	-3.21156507	1.97488913	-0.33137816	-2.96168372	0.68793584	-1.80486536
CD24	3.089827013	0.218525163	1.855793113	1.161466348	2.833449253	-3.62881029	-1.34177411
CYP27B1	3.088646795	0.865468291	3.603433717	0.81769147	3.456843609	1.946363334	1.928349389
CDC20	3.087740043	0.231952174	2.599872737	3.167575145	3.543744037	4.37302273	4.844760965
TRPM1	3.079790535	-0.10948834	0.67808222	1.369074481	6.526332245	-1.50938851	-1.57260614
SLC17A9	3.079308706	-0.89107194	0.366636561	2.280218322	0.955024527	-0.33967127	0.47943735
STX1A	3.078927265	0.050845341	0.935341061	1.627478483	5.071383131	0.789641087	2.729440633
KRTAP13-4	3.071055349	-0.84596494	-0.34119422	-2.92156996	1.214210885	0.002927515	0.258988438
DEFB103A	3.051567717	-0.03927306	1.058294646	0.532586735	0.198003768	0.552269175	0.034979068
LOC653260	3.047992123	-0.70517538	2.36699682	-1.09024153	-0.17802791	0.536568049	0.566398067
OR8J3	3.046401666	-1.4958503	0.91989673	1.350025812	-0.70732422	1.929444209	2.076390175
SLC26A8	3.041637094	-1.69535703	-0.37518956	-0.05104535	-1.74750606	-0.92480119	-2.9031838
ZNF268	3.036353052	-0.85817006	-0.47727681	-0.40976543	2.922036535	0.490251878	3.948571719
GAL3ST3	3.036246001	0.069685081	-0.43719383	-0.59318073	-1.05086957	-0.92539495	-1.36268379
SLC7A5	3.031822296	-0.74097402	1.990314192	1.025649222	2.691079878	4.89117934	5.243497301
KCNN4	3.023445021	0.251648986	1.547875854	2.293287596	6.700289301	-1.1132405	1.138556176
LOC10012841	3.02168102	-2.968251	0.059660829	1.257318134	-0.51980204	2.300224784	2.966337011
SCAMP5	3.020356531	-1.22308311	6.139087342	2.162977682	0.572919685	-0.14871492	1.416892125
PLEKHN1	3.013044182	0.015118299	0.436779472	0.648296884	3.614000979	2.676862667	1.277894375
FCRL5	3.01199352	-0.61011775	1.561110665	0.466042473	-0.39508641	0.335886316	-0.32256739
CXCL5	3.009964747	-0.44724399	-1.37453851	-0.87601772	-2.33083816	-2.19747004	1.595551991
METTL2A	3.002610094	1.69591439	-0.4492174	-3.61636495	0.044510406	3.854134089	0.119329415
TRYX3	2.987497547	-4.55155805	-1.95241179	0.66821034	-0.86125094	0.831967855	-0.41234257
PIM2	2.987192526	-0.69063067	1.30563007	-0.14254819	-1.00677106	1.476162868	-0.89531135

C1ORF125	2.977339876	-0.53248056	1.665717286	2.774746485	0.268006407	-2.63252342	-0.43222077
PACAP	2.97571208	-3.87192502	-0.69150332	1.076216103	1.273642416	0.219356722	0.341693761
WWP2	2.971602705	-0.67934944	0.240788166	1.910785027	-0.43699107	0.983060353	0.082843004
HS.124391	2.969643013	-0.60021156	0.581003605	-1.27766621	0.588371806	0.198013676	-1.93912231
CAPN5	2.947276144	-0.97662079	2.635074817	3.034824847	0.76831388	-1.19442182	0.937046715
LOC647493	2.943328753	2.45583075	0.953536675	2.142326801	0.082463839	0.770417135	2.581241104
FLG2	2.942045742	-2.12383204	0.962923039	-0.62188547	-4.01334717	-0.14967504	-0.41357523
LOC652102	2.942034821	-4.15339133	3.506333461	-0.13274468	-2.74507317	2.588153717	-4.83155796
HS.568690	2.940121843	-1.06445867	1.213436176	1.692809252	1.953163554	-2.85196738	4.757637133
CTSE	2.938836307	1.938602139	0.607384824	2.330010818	3.851252613	-0.60925	-3.4528766
MMP7	2.938422104	0.452696139	1.519273445	-1.42086888	4.988355698	0.83836768	-3.16178876
DERL3	2.9356966	0.498275783	0.811542177	0.340345731	1.572050861	1.999150943	0.06918067
OR1B1	2.932585058	-0.75814332	-0.86273162	-0.12507166	0.7138108	-0.21800873	0.277924626
LOC728760	2.927644887	0.009665098	0.144183022	-1.400915	0.321718972	3.938722388	-1.52288904
LOC645218	2.915664003	-1.18413549	-2.14279966	-0.17469006	0.562875604	1.01058163	1.483990078
LOC286467	2.910430294	1.98318643	2.599646514	-0.89145087	1.401181895	0.367940205	3.427863953
SLC44A4	2.882683166	0.644023741	0.748487477	2.705014203	0.847386679	-3.35834216	-1.56972272
EMR4	2.880430807	0.197416077	7.803627807	-0.20625673	2.976462576	0.926056494	-2.37010353
LAD1	2.875715953	0.728419753	1.498899534	2.903648697	2.647091815	2.411495736	2.206360903
LOC10013048	2.875365048	-0.65755322	-0.21768395	-2.12191245	-1.18848321	-0.53236149	3.143552727
TSPAN1	2.871984953	-0.42997178	0.192323828	1.699833275	-0.14198649	-2.13571897	-0.99651203
EPB42	2.86952633	-2.08461372	-0.65059057	-0.00785441	1.280173185	0.143873063	-0.15577613
SLAMF9	2.866356915	-1.36894426	-0.51454688	0.729616896	1.536949256	-3.01213573	4.294821689
SPACA1	2.865383361	-5.00854292	0.321922112	-0.85097289	-0.30596334	0.506993384	1.034922138
HS.561625	2.861937103	-1.28562989	0.078585215	-0.44079433	2.394488165	1.117272797	-0.32199238
LIPJ	2.861305278	-1.96321315	1.12407534	-0.06342263	0.892237083	0.302717421	1.741414738
MUC20	2.861190718	1.087207878	1.784906852	2.456422044	1.891968826	0.878259793	-0.45990988
LOC439950	2.852605223	-1.36634338	-1.17870957	-0.34074702	-2.54038482	-0.86217788	-0.23106069
LOC728454	2.842673953	-2.13609772	5.993151093	-0.57250412	-3.30138035	1.218823016	1.570800587
FLT4	2.82728029	0.008070116	-0.03227172	0.31993506	0.173753335	0.660158206	0.326044313
HRASLS2	2.823102753	-0.1794483	1.483442659	2.195050344	1.597798081	0.828539703	-0.55195291
CT47A6	2.816073571	0.795589956	-0.7417315	1.752488762	-2.72290527	-1.67585804	-1.26835951
LOC650861	2.809938748	-4.11959991	-2.10960804	-6.29926858	1.689743502	-0.4604754	2.004916791
HS.131802	2.804206776	0.111870868	0.927353334	-1.76769406	-0.96599862	-0.959591	0.441112613
LRRFIP2	2.803697974	-0.06541991	0.389438201	0.669950224	-0.56682043	0.24211121	-0.2060965
FAM177B	2.799373547	-1.40191382	-0.20605639	2.116608147	0.644562694	-0.10534714	3.858453886
KRT17	2.789832829	0.072403699	3.849844991	3.807123833	2.964578656	7.675459528	5.205609537
HS.164221	2.785916933	-0.56434453	1.227309414	1.757926667	0.449311367	2.480085725	4.365426112
BIRC5	2.777906872	0.177867841	1.346801142	2.997484875	3.954331778	4.291942062	3.823201266
SPAG4	2.776276546	-0.45756699	0.920923962	0.32525864	3.071065381	0.130208379	-1.64892854
C21ORF123	2.776237115	-0.5128019	-0.14002518	1.447799435	-0.51770032	0.150377009	-0.6813858
SLC6A10P	2.775653713	-0.15358271	0.398515133	1.686313729	2.549545981	4.015085291	2.177718123
GPT2	2.770097192	2.007724997	2.615398983	1.099851093	2.000245053	3.100601764	2.825887854
LOC650427	2.76657271	1.08555281	0.354963368	1.107033392	-3.57156269	-0.93295965	2.370114433
C6ORF165	2.760947435	-2.14064002	0.044925836	0.683688117	1.271294383	-0.07067881	-0.25490064
ZMAT5	2.754108922	0.494485077	-1.03616722	-0.61643578	-2.98226129	0.072278922	-0.06990662

LOC10012802	2.748102416	-0.17834765	-2.79188778	-1.07486197	1.79539381	0.854547769	-0.82660577
NUP62CL	2.744819071	-1.01427304	1.162267004	1.66665489	2.157401766	3.877818131	0.743815982
GINS1	2.740271157	-0.91308559	0.237439031	-3.30739073	-2.41279115	0.001420534	1.178909955
LOC653257	2.739494797	1.391230268	1.695788653	1.512451266	0.927577396	2.611620464	0.012807831
HS.557356	2.736088866	-1.15032003	-0.75042093	-0.96388039	1.012280241	-0.31551719	-0.08113216
NOS1AP	2.734717652	1.086349497	-0.02942448	0.664964693	3.475419623	-0.25259988	1.682564992
HS.54940	2.730108897	-1.62013522	-1.75786455	0.909567279	-6.08649826	-0.7598039	0.676443021
HS.566751	2.725892927	-1.04120864	-0.58131249	0.388755553	0.534617354	-0.24433128	0.659857397
DHX57	2.721997435	-1.4856157	0.978767883	-0.20858731	0.708790519	0.966959646	1.010152413
LOC652905	2.717432378	-0.78582008	-1.10558187	2.307131526	1.100082885	-2.29897045	1.220428925
LSR	2.712097241	1.414526827	0.652828334	0.034189798	1.620900573	0.63533299	0.714986193
MMP7	2.702957731	0.327365365	1.890977825	-1.47568658	4.592859051	0.85970194	-3.76707757
HS.579501	2.699030338	-0.65815131	-0.33393999	0.462595021	0.647761711	1.117337457	-0.19864183
KIR2DS5	2.698475129	-2.90979253	-0.94571494	-0.72558388	-0.9459963	-1.67779695	-0.66517033
RNF12	2.694759513	-2.42640834	-1.75968905	0.197023908	0.491174692	-1.32074287	7.596455047
BCAP29	2.691609323	-0.72063101	-1.21087952	-0.73497058	-0.37992886	0.281505182	-0.61321013
HS.553070	2.69132233	-1.58420224	1.197289615	-1.29545008	-0.20673877	1.40827671	-0.22599291
HS.120187	2.688308632	-0.3485039	0.506609753	-1.33310439	-2.46265057	-0.69906117	-0.07152003
GABPAP	2.684508251	-0.85698999	0.178491176	4.898151526	2.267316759	-1.93986972	-2.89435692
HS.421200	2.680742438	-2.14737186	0.31753881	0.964210855	-0.29939454	-1.09703425	2.221353653
SLC44A4	2.680074588	0.342355913	0.667922222	2.56486453	1.043859803	-3.39901331	-1.47341859
WFDC3	2.665037335	3.215262318	0.791814081	1.452454211	3.132070486	0.53596033	-1.17024074
RBM19	2.654333674	-2.38196588	-0.60745681	-0.26330446	-0.4575331	0.046966864	0.27080108
CRABP2	2.653509287	0.50107621	2.31434291	3.906041118	4.428537337	2.722232515	0.336408701
TNFRSF6B	2.652897626	-1.79859858	0.864931974	3.940305212	2.465921375	1.31042468	1.796280963
LOC642580	2.652117288	8.232636967	0.148603811	3.224420151	-0.80594159	3.170227616	-0.16393558
MYOG	2.649775478	-3.74560987	-0.78272823	-1.53685665	0.233944644	-2.07251094	-0.84411448
43898	2.646396497	-0.48020146	-0.9072782	0.657064678	0.71577209	-0.14830506	0.070862439
LOC644844	2.646225721	-0.53970206	-0.24587347	1.801660471	0.350522884	0.454668127	1.400454308
HS.197435	2.631507986	-0.18422253	1.136947788	0.553132521	0.78946258	-0.6565953	0.072361575
LOC644754	2.631302238	1.007147424	1.020437754	0.839059082	-1.35868253	2.760602625	-1.56324076
MIR939	2.630364656	-1.07074429	1.485167676	0.291120133	1.00037578	0.841792902	0.576411739
LOC401845	2.627726917	-3.28359885	1.099669086	-0.64290566	-1.05000021	1.712512482	0.357050721
LOC10013193	2.626427844	-0.11974305	-1.63432532	1.092782027	0.387435289	1.55417629	-0.56843027
KLK1	2.622463851	-1.52345041	-0.20827131	-2.1014218	0.741824305	1.45277079	-0.49486994
MIR1227	2.619805798	1.357250335	0.028800027	3.556961447	-1.01320073	-1.23942327	-0.47950017
GAS7	2.613398009	-0.01183155	1.720966918	-0.20665975	-1.17612086	1.257844435	-0.80962752
SORCS1	2.605901581	-0.67880461	-2.21370007	0.428048878	0.508306429	0.020911144	-0.92227517
ZC3HAV1L	2.598950301	-2.18999168	-0.40647462	1.53869201	0.390114031	1.960733197	1.305194674
NSF	2.596095729	-0.51782702	2.4226556	-0.04135822	-0.55649366	-0.72349504	2.072719088
AKR1C2	2.594738411	2.747070961	0.649776944	-2.28562969	0.813718281	4.667430631	1.387815726
HS.566309	2.594064193	1.915471452	-0.5936447	4.353531523	0.182389075	0.007069567	-0.2488776
SH3BGR	2.591611565	-0.17472924	-3.56184531	2.436383452	-1.2734339	-3.73407367	-0.24727416
HS.544353	2.59057448	0.115621981	1.751100327	-0.14027311	0.811862186	-0.74016927	1.833196479
LOC643937	2.587748798	-0.52788646	0.334761165	3.307826746	-0.2424717	-1.01254876	-2.52002308
RTKN	2.586046538	-1.21157543	-3.48228874	0.902397071	1.301431596	-1.42860611	-0.78469228

ADD3	2.584035016	-1.22782056	0.314549602	-5.30891703	0.684460214	1.836396015	1.500186577
FSHB	2.580235287	-4.47238295	0.924336557	2.382692621	-0.69122545	2.498247905	0.231386572
LOC650733	2.57892586	-0.52209312	-2.47495134	1.064623225	1.139533785	-0.98541329	1.216510395
HS.367445	2.578614099	-0.43036841	0.408414272	0.883766518	0.815774419	0.394209895	-0.66413319
ALPPL2	2.575276457	1.669971385	-1.45107558	-0.19574335	0.082185998	-0.04069506	-0.52435698
CTBP2	2.566816159	-0.8089332	0.260793786	0.144631438	-0.13211942	-0.21713461	-0.2450267
LOC10013356	2.565245841	0.288241926	1.688913518	-0.25109343	0.384282739	0.678493295	-3.40101028
LOC652775	2.562934408	-2.40387378	1.460965731	-0.20248441	-0.03001361	4.522153133	-0.53618432
BDH1	2.562688722	1.910637221	0.560344687	-0.11819582	0.958721971	0.511987575	-0.10033692
CARD11	2.559578467	-2.2694143	0.030912353	0.537994806	2.49259594	1.592987354	1.549710256
LOC642978	2.554862366	3.34243289	-0.26661882	1.093496766	-0.82588366	-0.13558341	3.704026882
PCSK6	2.554673692	-1.48253678	0.973419026	2.507782981	-0.55881905	-0.43517408	0.819158822
PTK6	2.553349602	0.951942449	1.514606831	2.733841068	2.201316491	-0.74324964	2.981491373
HS.390407	2.551786737	-1.51919824	0.158676285	-1.0818638	1.370777388	1.933130096	0.699310703
LOC402066	2.547230809	-0.63735445	2.135469055	-2.207632	-1.04682249	-0.62303215	-0.779882
LOC649246	2.546569406	-0.96545204	-0.33717671	-0.20840697	0.556404912	-0.32800121	-0.34119485
HS.529499	2.543476373	-0.70936596	-1.72347624	-1.03284716	-0.72298425	-4.0586227	-1.48394817
NSD1	2.542100273	-3.48531341	1.205977529	-0.68388046	-1.36603775	0.762337054	2.162020103
LOC25845	2.53614629	-0.43550956	0.258146105	0.039491152	0.041522723	0.263261939	0.898919506
G3BP2	2.535417848	-0.2325004	-0.03938594	0.016197108	0.343843644	2.03569488	-0.43602995
GSDM1	2.533082983	1.122068124	0.443325739	0.153400303	-2.44952005	-0.06301731	-0.01702174
HS.147310	2.533076838	-1.81164141	-0.93839886	1.003288072	0.940027419	-4.62689504	-0.85133013
HS.580375	2.525950835	-0.42563251	-2.13402571	-0.36130014	0.223278079	-1.48990588	0.715855452
LOC645165	2.519379805	-0.92366207	0.164449677	1.687143081	-0.07387833	0.323356379	-0.72994341
HAMP	2.514393592	2.318374629	1.77391619	3.106395449	3.574795913	2.367428997	1.301789535
HS.523705	2.513811897	-0.94742259	0.39190141	-0.01920286	-0.84851937	2.896021406	0.194569821
BACE2	2.512564169	0.569208157	1.189745078	1.631107519	1.442545101	0.463309921	2.893346714
C1ORF49	2.512214219	0.844915704	-0.1349417	0.982690339	0.578583638	0.441472699	1.103119308
LOC649941	2.51207316	-1.31626171	-0.63363498	0.528194519	0.311727761	-1.77957444	0.55160471
ZCCHC10	2.509510357	-1.48848502	1.282977852	-1.47493084	-0.63806925	0.064505906	-0.94738774
LOC651751	2.507648059	-2.72371131	0.02905038	-0.04061239	-3.11020162	1.690443285	-2.84335042
AAA1	2.504850839	-2.00596573	4.544394894	2.06539812	6.348474137	0.928279072	0.136100527
PODXL2	2.503780742	3.814702858	-0.14239703	-3.45762855	4.70479931	0.737246869	4.625383907
COL4A6	2.500799795	0.586863311	0.596803776	-0.35763959	0.265470121	0.386797271	-0.05807734
PTK6	2.48572885	-0.10449707	0.626134648	2.529582332	1.442659815	-1.25475879	3.241392338
HS.581690	2.485568872	-0.87945413	-0.10317692	0.594377957	0.363663967	-1.4221221	-1.66675722
LOC440005	2.479836978	-1.29825166	-0.68619614	-1.79323291	0.138929715	-0.0104894	-5.8672241
RPL13	2.476142906	-0.44276511	1.316257306	2.679632522	-0.00320565	1.459432493	6.217490424
RAET1K	2.475169745	-2.45532973	-1.74930786	-0.28987853	-1.13947415	5.330110534	-1.85261521
MEI1	2.473268582	-2.27677812	0.683304881	-1.42213054	-1.5173629	0.573414088	-1.18585323
C6ORF1	2.471860975	-2.11883959	-0.00119195	-0.38190203	2.179654453	1.134130355	0.294183216
C1ORF124	2.470138726	-3.98261881	-1.42851073	-0.36631034	-1.91470099	0.445627183	0.350753011
C3ORF30	2.463497338	-4.01327959	-0.71019963	-0.76576673	-0.40648948	-0.84273995	-0.41288581
SLC1A7	2.463096146	-0.20189739	-1.25748626	-0.60447819	-1.67066488	-1.63680555	-0.25328325
NR1I2	2.462929919	-2.75388755	-0.45932568	1.368769664	5.253227254	-4.46198351	-2.88474344
COL25A1	2.459767132	0.25039337	-0.45561304	-1.1706795	0.003697061	0.071835584	0.246690098

LOC646128	2.453918553	-0.1927793	-1.18798934	0.429750508	0.419033911	0.567095929	0.485627274
DGKZ	2.453496951	-0.56973278	-0.04611883	0.325357229	1.107705311	0.24341734	-0.33333528
LOC648329	2.449802655	-0.9653484	0.217484464	0.690660025	0.295940478	-1.47765198	-0.39242349
TCERG1L	2.444736635	0.181907283	0.875080489	-0.69110119	0.446191176	-0.8592279	-1.06958907
SNORD4B	2.442942169	0.598933909	0.588577195	0.604758367	-0.34395156	0.586179666	2.652120514
ANKS1B	2.441221689	-2.4319579	0.584298338	-1.4379828	-1.6803308	1.890286854	0.292113847
ALS2CR12	2.438887901	-2.05788532	-0.55821768	0.357306823	-3.71076887	-3.38613644	0.1173217
LOC728119	2.438342522	-1.60423134	-0.39575038	-0.46538226	-1.00259389	-0.98903748	-0.79129542
LOC10012970	2.437517982	-6.28407114	-0.87554269	1.002802483	0.072593525	0.928953895	-0.67898419
LOC440122	2.436613703	2.413090107	0.323529365	1.476392426	-1.95285331	-0.26855867	-0.38048247
AGR2	2.429495726	2.538635751	1.841544841	4.794262678	2.921485468	-3.96816009	1.41866806
SERPINC1	2.427146567	-2.08468406	1.771743982	0.556953999	1.730762468	0.060664737	-0.7923222
LOC146439	2.426417288	1.535197333	0.632016378	0.838726606	1.029263363	-1.34376244	1.359839328
ODF3	2.426137292	0.520137633	3.791416651	-1.76404551	-1.22249542	-0.04623912	-1.74893681
HS.151172	2.422902638	-1.56565198	0.26005745	-4.71848494	0.339092597	-0.93320838	2.754928098
LYNX1	2.415533717	-1.63907904	0.346396267	0.31931248	-2.23513508	-0.5522462	-0.1346705
LOC389722	2.413497238	-0.18290148	1.100833955	-0.07639516	1.308414616	-1.04774203	0.689398048
CXCR4	2.413483856	-0.67999536	-0.85251745	1.500833788	-2.44282595	4.794397487	-1.62643842
DDI2	2.413094273	-0.17155076	-1.04495611	-0.35481981	-0.4522656	1.384523554	-0.76063916
NEK2	2.409643059	0.144533205	2.391134599	3.038473465	4.974180153	4.908578083	3.779098275
LOC346157	2.407895289	4.846163594	2.192566971	-1.50742217	-0.34711312	1.971268466	1.111723408
PPID	2.407878627	-1.1995726	0.495528767	-0.58954438	0.474330398	1.609019802	-0.27953421
PYCR1	2.406324273	2.893048979	2.612748336	2.424999261	4.701379625	2.571259352	2.648329252
ACBD5	2.402956206	-6.17513535	0.258911863	-3.04730966	0.669467064	-0.24773062	0.84261784
LOC730234	2.398938449	0.202337421	-1.49370941	-0.38254583	2.596284434	0.131369115	2.91297179
CCDC141	2.398208313	0.10720492	-0.08201971	0.041667034	0.448076104	0.965571864	-0.08664638
CFTR	2.398185109	-5.58742352	-0.41268094	-1.41259404	-5.55180677	-2.80225068	-2.90879021
HS.538367	2.396895214	-1.13725961	-0.1335116	0.052324636	1.530168606	-1.00182407	0.273120339
HCN2	2.395782152	2.113207469	-3.21515452	1.907485097	-0.15954945	-2.19359701	0.653744345
CCL11	2.395532451	0.010398771	0.542324117	-0.47336236	-1.31124287	2.231066958	-5.19423879
FLJ41484	2.390729538	1.062224811	-0.86246234	-0.66993457	1.095407024	0.289922807	2.003596312
CRABP1	2.390251419	1.483883542	1.898636068	-0.98604561	-2.17821314	0.35840789	1.085399373
FAM131C	2.370934866	-3.47776979	2.646166888	-2.24290587	1.099232888	1.66488757	-0.90085609
ELF3	2.370370423	0.863678836	1.656679594	2.196002395	0.667625025	0.685638495	2.023091757
TAC4	2.367310074	0.199775777	-0.40525714	-0.10968212	-0.18827901	0.061563084	-0.23536824
IRF6	2.366453852	-0.11865717	0.248138877	1.180579244	-0.86086039	3.630892433	-0.36413647
LOC727880	2.364151661	0.581861985	0.477955719	-0.02220506	-0.62725562	-6.70944917	1.240352518
HS.242717	2.35919686	0.32749326	-0.28436863	-1.32456824	-0.90273445	-0.43526584	0.289066133
LOC646561	2.356586688	-0.66557136	0.357583585	-3.68679819	1.770556414	2.354395371	-1.33468864
LOC646823	2.355624068	-0.48513148	5.155810145	-0.25296325	-1.74821808	-0.84395809	1.812509674
MME	2.354971917	0.783742933	-0.34702039	0.386947046	-0.29901585	0.917185784	0.217310945
HS.569411	2.353387218	-0.79316747	0.014994029	-0.13433165	0.970325127	-0.81333113	1.159677851
CCR9	2.35305993	-1.03979416	-1.16364796	-0.92600665	-1.72897875	1.85289171	0.956667044
LOC727773	2.352368249	-0.02145971	-0.66761533	-0.4958734	-0.76182113	0.512855576	0.066936813
HS.246726	2.349079627	-3.71017109	2.128686595	0.839537786	1.74755094	-1.48548907	-1.29241492
RASSF3	2.347609451	-0.97494926	-0.55614728	0.538578246	1.658744004	1.210914009	0.102607866

UBE2C	2.341659313	0.815181738	1.598122036	1.793168409	4.585832873	4.602083762	5.868619784
SCN5A	2.339261566	-0.72856269	-0.67785831	-0.7088179	0.624080559	0.029391809	-0.09510736
HS.546114	2.338583474	-0.67841874	-1.9290568	-0.38524701	-2.47562226	0.634815156	0.32032165
LOC441554	2.335457575	-0.22315777	-0.34245885	-0.78305314	-0.58699241	0.47001959	0.564738007
IER3	2.335159663	1.841720485	2.255694429	1.177373292	0.663440948	1.738402177	3.757194305
SNORA21	2.33410112	-1.8516035	0.667655405	-0.05462593	0.948984679	-1.66414749	1.351505708
ZNF85	2.333926443	-1.2145287	-0.59613937	-0.30891522	0.448815987	0.274176493	0.910879472
ANKRD20A3	2.331112125	-1.49881414	0.549806174	0.978179957	0.739617405	0.151558767	-0.25248252
MIR1274B	2.330488696	-2.43929067	0.612193245	0.93820686	2.390797637	2.421621347	-0.47476288
LOC642756	2.329805212	-2.88679991	2.536715198	0.778963645	3.088634247	1.887837769	0.363322737
HS.489857	2.328726555	-1.31714661	1.644438386	-0.93503554	-0.668997	-3.26729018	0.920873044
EIF3IP1	2.321513717	-1.54927581	1.190446907	-0.19642672	0.998272414	0.859488093	-4.18865809
LOC440895	2.315395442	1.032588911	0.379202342	-1.85187129	1.96914921	0.897221834	-0.20530181
TSPAN8	2.31521404	-2.0353366	1.107469111	2.362762105	0.141417483	-3.87527779	1.482505467
RHBDL1	2.309951458	1.487571462	1.609038817	2.688366918	2.051979145	2.021559226	4.347951612
HS.171397	2.3087049	-0.52877845	1.04034819	0.53018946	-0.24229007	-0.05270049	-0.69046652
STAU1	2.307800126	-1.60861429	3.285638908	-1.30528838	-1.40285752	-0.8753476	0.338289706
CNDP1	2.307631165	-0.92778573	-1.35420007	-0.29690929	0.388149108	0.915262676	0.028006509
SLC2A7	2.307524837	-3.54558007	-0.04943415	-0.96545636	-0.09404061	1.654355591	-0.51030056
P4HA3	2.307226373	-6.54775868	0.737241689	1.157516475	-2.73622981	1.830604824	1.065732013
ZNF572	2.305789288	0.95559349	-1.15252497	0.555618331	0.820010259	0.456191401	-1.22088564
44076	2.305728594	-2.17342552	0.914242383	-0.50370456	2.750765227	-0.81206706	-0.24649298
UBE2C	2.304506462	1.082897425	2.436357808	1.987912804	4.320749047	3.511894014	6.704892132
GMDS	2.302184928	0.291829381	1.41443803	2.075910759	1.48227244	-0.39419369	1.13296665
MMP11	2.3016686	3.452293626	6.007459072	-0.64105966	7.382636643	5.393270435	-2.73620927
HS.151334	2.301453511	-2.00898886	-0.86955253	1.600921302	1.820995731	0.350003602	1.419794107
TESC	2.301128739	0.10704684	1.211248459	1.142422245	1.535337882	-3.3225982	4.997304755
FLJ34969	2.299066865	0.171394296	1.357861188	-0.41672101	0.401049076	0.676934298	-0.39736382
BATF	2.297376523	-0.97207828	0.890659226	-1.42163954	0.591096621	0.300255189	2.259426377
SLC17A2	2.29489435	-0.61210113	-1.02147737	-1.06807367	0.14013566	0.164477919	-0.4561185
HS.481464	2.294730742	-0.69530529	0.082238728	0.279888325	-0.02575167	0.244228391	1.008152673
NRSN1	2.292036613	-0.20682819	-0.47797029	-0.19091095	-0.45677354	0.494870402	-0.50562108
FLJ44450	2.287494478	-1.90461002	-2.74531959	-3.32895625	-0.27134813	-0.84167279	1.772615831
HS.534997	2.284757891	-0.63872312	-0.1517134	0.125701174	5.875392686	1.768304276	0.360097485
GSTTP2	2.282831856	1.828008545	-0.10718115	-1.15392759	-0.23969943	0.558775638	1.734821781
C12ORF64	2.281577993	-0.09186471	0.576468173	-0.31583462	1.705540587	0.200949641	-5.66975764
LHFPL1	2.280593661	0.529645606	-1.55689191	-0.751311	0.966602313	1.401905804	-0.12650547
HS.120938	2.280419391	-0.6933833	-1.45611419	0.169089836	-1.69508036	-1.43314123	-0.83946701
CEACAM1	2.280384274	-0.54369809	1.419345757	0.544252907	0.999375186	1.136339723	0.178841697
LOC653214	2.279680027	-0.43989492	-0.23829462	0.674969123	-1.87753179	-1.48963141	-0.87138321
LOC440518	2.278356208	-0.73066867	-2.3890847	0.573364868	0.163923658	-0.36846238	-0.83861846
IGDCC3	2.275900835	-2.36442221	-0.53417839	0.337373454	0.12026379	1.933046596	-0.24394986
MS4A12	2.271057199	-2.72685047	0.126313302	-0.41577684	-0.16809865	0.307029087	-0.35576218
NT5DC3	2.268247865	-0.42112498	-1.54225272	-1.91493658	-0.1953598	0.129719017	4.693262012
GSDMB	2.267550881	0.059108693	1.636497744	0.717034656	0.400406296	1.174587615	0.382718462

Supplementary Table S4. Genes related to anti-lung cancer proliferation

Gene Symbol	Lung Tumor Tissue (LTT) vs. matched Lung Normal Tissue (LNT)						
	LTT36	LTT21	LTT33	LTT43	LTT42	LTT47	LTT08
AGER	-6.79145807	-5.50767663	-7.20881962	-2.60696733	-8.35609053	-7.95919559	-6.91237825
HSD17B6	-6.54020106	-3.10024039	-5.38075662	-2.24286092	-3.30723154	-2.98534229	-8.47944038
NKX2-1	-5.50716381	-0.06513499	-1.06704943	-0.04671198	-0.27088388	-4.13760292	-5.84334805
WIF1	-5.4647533	-4.11490621	-5.43042954	-1.7824146	-5.26298829	-6.27091216	-2.50501747
FHL1	-5.2341466	-2.88266141	-2.59113639	-3.10816742	-3.84232856	-2.12692508	-2.96897811
TNNC1	-5.17323456	-1.90728894	-5.34210417	-1.88558226	-4.47418134	-7.07228914	-6.96373625
TMEM100	-5.00056052	-5.62474307	-6.68112286	-3.52007985	-6.51332337	-4.89437077	-6.59518728
GPD1	-4.97943635	-4.9470724	-2.53868369	-1.52307607	-2.89722409	-2.4682942	-2.94342328
CAV2	-4.79453441	-4.82006364	-3.62757704	-3.60855218	-4.29112711	-2.14361962	-2.84270266
DCN	-4.76678742	-3.14423235	-2.12279808	-2.56509566	-1.45351816	-2.75738764	-3.79938333
EYA4	-4.52982649	-2.84708451	-0.86742152	-2.6414716	-2.69623312	-2.52807997	-1.59341341
ARHGAP24	-4.47917164	-3.74450495	-2.92183033	-1.5091272	-1.58225499	-1.52754894	-0.99304065
ADAMTS8	-4.43229628	-3.76337217	-1.82057314	-2.79729472	-5.25074918	-5.11669655	-3.21147956
C4ORF31	-4.25797108	-1.81959023	-2.36008342	-1.52652324	-1.63398771	-4.61575863	-4.63933852
TCF21	-3.94180744	-4.04992827	-3.81382975	-2.75739747	-4.68157947	-5.22349997	-3.3759856
RASSF2	-3.63573185	-2.1609443	-1.49349806	-0.62165599	-0.80715884	-1.60711165	-1.066026
RORA	-3.62993517	-5.40821986	-1.82307317	-1.70043269	-2.47328627	-1.34415237	-2.00239661

Gene	References
AGER	Mol Med Rep. 2020 Aug;22(2):810-818.
HSD17B6	Cell Death Discov. 2021 Nov 8;7(1):341.
NKX2-1	Oncogene. 2022 Jan;41(2):293-300.
WIF1	J Thorac Cardiovasc Surg. 2007 Mar;133(3):733-7.
FHL1	Int J Cancer. 2012 Jun 1;130(11):2549-56.
TNNC1	Mol Cells. 2020 Jul 31;43(7):619-631.
TMEM100	Oncol Rep. 2021 May;45(5):63.
GPD1	Cancer Res. 2020 Jun 1;80(11):2150-2162.
CAV2	Int J Oncol. 2011 May;38(5):1395-402.
DCN	Am J Physiol Lung Cell Mol Physiol. 2019 Apr 1;316(4):L630-L643.
EYA4	Oncogene. 2014 Sep 4;33(36):4464-73.
ARHGAP24	Carcinogenesis. 2020 Jul 10;41(5):711-721.
ADAMTS8	Biochem Biophys Res Commun. 2022 Apr 2;598:1-8.
C4ORF31	JCI Insight. 2019 Dec 19;4(24):e129344.
TCF21	Proc Natl Acad Sci U S A. 2006 Jan 24;103(4):982-7.
RASSF2	Mol Biol Int. 2012;2012:705948.
RORA	Cell Death Dis. 2022 May 3;13(5):427.

Supplementary Table S5. Genes related to anti-lung cancer migration or invasion

Gene Symbol	Lung Tumor Tissue (LTT) vs. matched Lung Normal Tissue (LNT)						
	LTT36	LTT21	LTT33	LTT43	LTT42	LTT47	LTT08
AGER	-6.79145807	-5.50767663	-7.20881962	-2.60696733	-8.35609053	-7.95919559	-6.91237825
HSD17B6	-6.54020106	-3.10024039	-5.38075662	-2.24286092	-3.30723154	-2.98534229	-8.47944038
NKX2-1	-5.50716381	-0.06513499	-1.06704943	-0.04671198	-0.27088388	-4.13760292	-5.84334805
TNNC1	-5.17323456	-1.90728894	-5.34210417	-1.88558226	-4.47418134	-7.07228914	-6.96373625
TMEM100	-5.00056052	-5.62474307	-6.68112286	-3.52007985	-6.51332337	-4.89437077	-6.59518728
EFEMP1	-4.49062803	-3.24743902	-1.45033323	-2.09952091	-2.11195844	-2.99852979	-3.31955295
ADAMTS8	-4.43229628	-3.76337217	-1.82057314	-2.79729472	-5.25074918	-5.11669655	-3.21147956
SFTPD	-4.17039684	-0.94711417	-5.6249588	-0.57407578	-5.30010047	-3.11983288	-7.83050279
KLF4	-4.04112779	-5.6639975	-2.03411951	-3.04716465	-2.65706194	-2.61937342	-2.22291268
CAT	-3.66983539	-3.14109391	-1.34676843	-1.96996139	-2.79725089	-1.76339778	-3.02326354
RASSF2	-3.63573185	-2.1609443	-1.49349806	-0.62165599	-0.80715884	-1.60711165	-1.066026

Gene	References
AGER	Mol Med Rep. 2020 Aug;22(2):810-818.
HSD17B6	Cell Death Discov. 2021 Nov 8;7(1):341.
NKX2-1	Sci Rep. 2018 Sep 26;8(1):14418.
TNNC1	Mol Cells. 2020 Jul 31;43(7):619-631.
TMEM100	Am J Transl Res. 2017 May 15;9(5):2567-2578
EFEMP1	Zhongguo Fei Ai Za Zhi. 2015 Feb;18(2):92-7.
ADAMTS8	Biochem Biophys Res Commun. 2022 Apr 2;598:1-8.
SFTPD	Oncogene. 2017 Nov 16;36(46):6432-6445.
KLF4	J Cancer. 2017 Sep 27;8(17):3480-3489.
CAT	Exp Cell Res. 2014 Apr 15;323(1):28-40.
RASSF2	Mol Biol Int. 2012;2012:705948.

Supplementary Table S6. Genes related to anti-lung cancer progression

Gene Symbol	Lung Tumor Tissue (LTT) vs. matched Lung Normal Tissue (LNT)						
	LTT36	LTT21	LTT33	LTT43	LTT42	LTT47	LTT08
CD300LG	-6.95868337	-5.28105244	-5.75707988	-2.74988745	-5.78833015	-6.78364146	-4.40759153
HSD17B6	-6.54020106	-3.10024039	-5.38075662	-2.24286092	-3.30723154	-2.98534229	-8.47944038
AGTR1	-5.7474551	-3.83944735	-3.92458329	-3.4247347	-1.96239768	-5.05709199	-1.88403644
NKX2-1	-5.50716381	-0.06513499	-1.06704943	-0.04671198	-0.27088388	-4.13760292	-5.84334805
FHL1	-5.2341466	-2.88266141	-2.59113639	-3.10816742	-3.84232856	-2.12692508	-2.96897811
TNNC1	-5.17323456	-1.90728894	-5.34210417	-1.88558226	-4.47418134	-7.07228914	-6.96373625
TMEM100	-5.00056052	-5.62474307	-6.68112286	-3.52007985	-6.51332337	-4.89437077	-6.59518728
GPD1	-4.97943635	-4.9470724	-2.53868369	-1.52307607	-2.89722409	-2.4682942	-2.94342328
DCN	-4.76678742	-3.14423235	-2.12279808	-2.56509566	-1.45351816	-2.75738764	-3.79938333
EFEMP1	-4.49062803	-3.24743902	-1.45033323	-2.09952091	-2.11195844	-2.99852979	-3.31955295
ARHGAP24	-4.47917164	-3.74450495	-2.92183033	-1.5091272	-1.58225499	-1.52754894	-0.99304065
ADAMTS8	-4.43229628	-3.76337217	-1.82057314	-2.79729472	-5.25074918	-5.11669655	-3.21147956
C4ORF31	-4.25797108	-1.81959023	-2.36008342	-1.52652324	-1.63398771	-4.61575863	-4.63933852
SFTPD	-4.17039684	-0.94711417	-5.6249588	-0.57407578	-5.30010047	-3.11983288	-7.83050279
RGMB	-4.0540221	-0.68851785	-1.02697938	-0.79991505	-0.87390551	-0.74402878	-1.57004482
KLF4	-4.04112779	-5.6639975	-2.03411951	-3.04716465	-2.65706194	-2.61937342	-2.22291268
TCF21	-3.94180744	-4.04992827	-3.81382975	-2.75739747	-4.68157947	-5.22349997	-3.3759856
SFTPA1	-3.76646888	-4.35821257	-0.71552711	-0.72690127	-3.24767798	-2.89896442	-4.14878384
TEK	-3.73925775	-4.2145642	-3.40662169	-3.11704344	-3.70761272	-3.58629036	-3.83398206
FMO2	-3.68752007	-4.32087161	-2.8119758	-2.49412322	-5.60595995	-5.04665788	-3.49911053
RORA	-3.62993517	-5.40821986	-1.82307317	-1.70043269	-2.47328627	-1.34415237	-2.00239661

Gene	References
CD300LG	Monoclon Antib Immunodiagn Immunother. 2016 Apr;35(2):94-9.
HSD17B6	Cell Death Discov. 2021 Nov 8;7(1):341.
AGTR1	Cancer Manag Res. 2021 Nov 13;13:8535-8550.
NKX2-1	Nature. 2011 May 5;473(7345):101-4. (progression, metastasis)
FHL1	Int J Cancer. 2012 Jun 1;130(11):2549-56.
TNNC1	Mol Cells. 2020 Jul 31;43(7):619-631.
TMEM100	Oncol Rep. 2021 May;45(5):63.
GPD1	Cancer Res. 2020 Jun 1;80(11):2150-2162.
DCN	Biomed Res Int. 2015;2015:654765.
EFEMP1	Zhongguo Fei Ai Za Zhi. 2015 Feb;18(2):92-7. Chinese. (invasion, progression)
ARHGAP24	Carcinogenesis. 2020 Jul 10;41(5):711-721.
ADAMTS8	Biochem Biophys Res Commun. 2022 Apr 2;598:1-8.
C4ORF31	JCI Insight. 2019 Dec 19;4(24):e129344.
SFTPD	Oncogene. 2017 Nov 16;36(46):6432-6445.
RGMB	Oncotarget. 2016 Mar 29;7(13):15678-89.
KLF4	Cell Death Differ. 2016 Feb;23(2):207-15. (progression)
TCF21	Oncotarget. 2016 Dec 27;7(52):87081-87090

SFTPA1	Am J Pathol. 2013 May;182(5):1843-53.
TEK	Cancer Biother Radiopharm. 2021 Jan 25.
FMO2	Oncotarget. 2017 Sep 18;8(62):104831-104854.
RORA	Cell Death Dis. 2022 May 3;13(5):42

Supplementary Table S7. Genes related to lung cancer proliferation

Gene Symbol	Lung Tumor Tissue (LTT) vs. matched Lung Normal Tissue (LNT)						
	LTT36	LTT21	LTT33	LTT43	LTT42	LTT47	LTT08
CDCA7	4.676491171	2.853486069	1.298789755	2.728261646	3.112244588	3.131761019	3.958604662
MNX1	4.364286107	0.613200388	1.560926848	2.225036592	4.393063031	0.114987842	1.313706098
ACOT11	4.356010474	0.760779962	0.579610321	0.908924505	2.192797712	1.121167394	1.206062022
KIF2C	3.513028161	0.761306515	0.973122889	3.713896549	2.762217242	3.676446125	4.314281616
ETV4	3.500610402	4.609223471	2.655796664	0.868547098	2.688190556	3.781023444	3.264086862
LAD1	2.875715953	0.728419753	1.498899534	2.903648697	2.647091815	2.411495736	2.206360903
KRT17	2.789832829	0.072403699	3.849844991	3.807123833	2.964578656	7.675459528	5.205609537
LSR	2.712097241	1.414526827	0.652828334	0.034189798	1.620900573	0.63533299	0.714986193
CRABP2	2.653509287	0.50107621	2.31434291	3.906041118	4.428537337	2.722232515	0.336408701
NEK2	2.409643059	0.144533205	2.391134599	3.038473465	4.974180153	4.908578083	3.779098275
PYCR1	2.406324273	2.893048979	2.612748336	2.424999261	4.701379625	2.571259352	2.648329252
ELF3	2.370370423	0.863678836	1.656679594	2.196002395	0.667625025	0.685638495	2.023091757
UBE2C	2.341659313	0.815181738	1.598122036	1.793168409	4.585832873	4.602083762	5.868619784

Gene	References
CDCA7	Pathol Res Pract. 2019 Nov;215(11):152559.
MNX1	J Cell Biochem. 2018 Dec 2.
ACOT11	Transl Lung Cancer Res. 2020 Oct;9(5):1885-
KIF2C	1903. Cancer Biomark. 2019;24(3):371-382.
ETV4	Biochem Biophys Res Commun. 2019 Aug 13;516(1):278-
LAD1	284. Cancer Cell Int. 2021 Feb 3;21(1):85.
KRT17	Med Sci Monit. 2018 Jul 11;24:4782-4790.
LSR	Comput Math Methods Med. 2021 Mar
CRABP2	8;2021:6651907. Sci Rep. 2019 Jan 29;9(1):845.
NEK2	Int J Biol Sci. 2021 May 11;17(8):1995-2008.
PYCR1	Biomed Pharmacother. 2019
ELF3	Mar;111:588-595. Int J Biochem Cell
UBE2C	Biol. 2018 Jan;94:98-106.Oncogenesis.
	2018 Jun 13;7(6):49.

Supplementary Table S8. Genes related to lung cancer migration or invasion

Gene Symbol	Lung Tumor Tissue (LTT) vs. matched Lung Normal Tissue (LNT)						
	LTT36	LTT21	LTT33	LTT43	LTT42	LTT47	LTT08
MNX1	4.364286107	0.613200388	1.560926848	2.225036592	4.393063031	0.114987842	1.313706098
ACOT11	4.356010474	0.760779962	0.579610321	0.908924505	2.192797712	1.121167394	1.206062022
ETV4	3.500610402	4.609223471	2.655796664	0.868547098	2.688190556	3.781023444	3.264086862
LAD1	2.875715953	0.728419753	1.498899534	2.903648697	2.647091815	2.411495736	2.206360903
KRT17	2.789832829	0.072403699	3.849844991	3.807123833	2.964578656	7.675459528	5.205609537
LSR	2.712097241	1.414526827	0.652828334	0.034189798	1.620900573	0.63533299	0.714986193
CRABP2	2.653509287	0.50107621	2.31434291	3.906041118	4.428537337	2.722232515	0.336408701
PYCR1	2.406324273	2.893048979	2.612748336	2.424999261	4.701379625	2.571259352	2.648329252
ELF3	2.370370423	0.863678836	1.656679594	2.196002395	0.667625025	0.685638495	2.023091757
UBE2C	2.341659313	0.815181738	1.598122036	1.793168409	4.585832873	4.602083762	5.868619784

Gene	References
MNX1	J Cell Biochem. 2018 Dec 2.
ACOT11	Transl Lung Cancer Res. 2020 Oct;9(5):1885-1903.
ETV4	Biochem Biophys Res Commun. 2019 Aug 13;516(1):278-284.
LAD1	Cancer Cell Int. 2021 Feb 3;21(1):85.
KRT17	Cancer Manag Res. 2019 Aug 7;11:7485-7497.
LSR	Comput Math Methods Med. 2021 Mar 8;2021:6651907.
CRABP2	Sci Rep. 2019 Jan 29;9(1):845.
PYCR1	Mol Carcinog. 2020 May;59(5):503-511.
ELF3	Int J Biochem Cell Biol. 2018 Jan;94:98-106.
UBE2C	Oncogenesis. 2018 Jun 13;7(6):49.

Supplementary Table S9. Genes related to lung cancer progression

Gene Symbol	Lung Tumor Tissue (LTT) vs. matched Lung Normal Tissue (LNT)						
	LTT36	LTT21	LTT33	LTT43	LTT42	LTT47	LTT08
GPX2	9.100938754	2.915810786	4.943764547	1.503770889	5.592049258	8.599964224	7.962866552
ETV4	3.500610402	4.609223471	2.655796664	0.868547098	2.688190556	3.781023444	3.264086862
KRT17	2.789832829	0.072403699	3.849844991	3.807123833	2.964578656	7.675459528	5.205609537
NEK2	2.409643059	0.144533205	2.391134599	3.038473465	4.974180153	4.908578083	3.779098275

Gene	Reference
GPX2	Tumour Biol. 2017 Jun;39(6):1010428317700410.
ETV4	Mol Carcinog. 2020 Jan;59(1):73-86.
KRT17	Med Sci Monit. 2018 Jul 11;24:4782-4790.
NEK2	Int J Biol Sci. 2021 May 11;17(8):1995-2008.

Supplementary Table S10. 500 ea up-regulated genes based on TLR4 in A549 cells

Gene_Symbol	Fold change (TLRs vs. vehicle)		
	TLR2	TLR4	TLR7
MIR4260	14.30908363	15.85570351	13.09135709
MIR6511B1	0.321928095	14.85197469	13.61423535
MIR611	15.72162792	14.62601698	8.816343705
MIR6861	-0.222392421	14.20440739	-0.807354922
USP17L13	5.300804803	6.756439956	7.823441302
USP17L17	4.953113612	6.481001494	8.012786926
RNU1-39P	1.99619843	6.310875827	7.147820815
PGBD3	4.826213949	6.267448306	5.746755268
USP17L12	6.601450624	5.899409014	5.721963431
ST13P3	4.847739994	5.479757738	3.176166199
CCDC58P3	4.314642605	5.465956809	5.569290762
ATP6V1G2-DDX39B	0.451283971	5.141831484	0.392182148
RPL12P12	3.79824497	4.67930415	5.839817905
TPTEP2-CSNK1E	0.224695305	4.494502385	6.581870441
ST7-OT3	3.099142158	4.488409839	5.726410155
DNAJC25-GNG10	0.591106481	4.302196081	-3.84390875
CDK2AP2P2	6.637216313	4.283790775	5.70986452
LOC440896	3.729999266	4.135036958	3.121027357
MIR6733	6.028201354	4.124542849	5.252424896
RPL17P5	3.740205159	3.915968004	4.150681107
SNORA31B	5.491853096	3.906890596	3
RBBP4P1	1.908928997	3.735072563	3.945328379
CPAMD8	3.146657547	3.731274046	1.286646068
NDUFA4L2	3.338800279	3.728079614	2.115470007
ISCA1P4	2.804583348	3.727063295	2.3467195
MIR661	4.41957683	3.499963811	0.31723239
HNRNPA1P21	3.327146939	3.434487339	4.247306688
MIR6746	1.474242341	3.418737352	2.37351761
H2BP2	2.750454812	3.410491236	-0.117887736
LINC01252	2.02790356	3.396550065	2.215164504
RAB4B-EGLN2	1.518567999	3.340628963	3.540005517
UTP14C	0.613709532	3.321128358	2.305916519
CCDC58P4	4.641696819	3.291017582	2.524919173
CBS	3.067855578	3.173325725	1.996986135
DUSP8P2	3.711630659	3.089711881	1.055764549
INHBE	2.904142312	3.088724936	2.279592604
MIR7111	2.923264194	3.083944626	2.772171864
MIR5006	3.298655145	3.06716848	-7.298525732
EIF1P3	4.479796481	3.051073576	3.397031986
THCAT158	1.905357655	3.027083602	1.457967609
MEF2B	2.847304618	3.023100092	2.254849549

SNTB1	2.692287097	3.009457312	2.481829205
MIR10393	4.75470263	2.969563597	4.047806947
PTPRN	0.679952707	2.954806401	3.252451503
HMGN2P1	1.552144939	2.952670709	1.596334145
MIR6750	1.800691002	2.926653897	1.062199854
YRDCP1	1.65397347	2.918368868	2.272779543
MIR4519	2.128931118	2.917785823	1.865645791
HDHD5-AS1	1.838250395	2.913153925	0.725093109
PCF11-AS1	0.682989868	2.900372026	3.358670391
USP17L19	4.953113612	2.89566334	6.727287277
RPS2P35	3.68740977	2.863784022	2.582566429
NIBAN1	2.954269803	2.837884495	0.602099557
PCDHGB6	2.910991838	2.822532072	1.317173565
LOC441241	3.773354251	2.820935713	0.215451467
FUT1	2.821208457	2.804692933	1.882317037
MIR138-1	3.316820946	2.788009	2.654025245
ADM2	3.13743589	2.777131042	2.602196484
MIR628	1.245464429	2.774696491	1.585693464
MIR1181	2.798271817	2.753321665	0.425562679
MIR4315-2	1.5978162	2.746762554	1.444052897
YPEL1	2.508176001	2.731606447	1.037753436
PTMAP7	2.032203882	2.71461441	2.992102988
C11orf96	2.547088721	2.705947557	3.167993416
PPIAP54	2.91881819	2.681664414	2.639158911
NANOGP7	1.435305305	2.641713619	0.916347593
DUSP8P1	0.792566995	2.599558892	0.864915077
ASNS	2.540844374	2.581906163	1.461765621
IGFBP1	3.068320255	2.568683147	1.244697304
GOLGA6A	2.348698749	2.549787865	2.351929739
NPM1P35	3.400729663	2.539062237	2.771932199
CORO7-PAM16	0.130395411	2.536773694	2.51420705
NUPR1	2.481264874	2.529977966	1.387542595
PPIAP19	1.245705839	2.495392535	1.52692251
GOLGA6B	0.415168576	2.4803713	1.523459366
TCAF2P1	0.614262057	2.479519804	2.340679485
FLRT1	2.466591475	2.475419703	1.831229878
LOC100130673	0.430266941	2.470860878	-1.333727456
ATP5F1AP8	2.649982074	2.393318959	1.002385953
ARPC3P1	2.91791599	2.39171991	2.224183477
MIR3620	1.914090357	2.390235329	2.497212365
LGI4	1.535234812	2.363010346	2.231590465
LINC00605	2.671466636	2.359965347	1.026536488
PSIP1P1	-0.802053727	2.355681549	1.317349389
COMMD3-BMI1	3.004214806	2.34336234	5.087890846

RPL15P18	1.972018713	2.336702801	-1.390641928
BCAT1	2.153024313	2.335229414	1.343981952
ZASP	0.796158025	2.327558377	-4.788341352
ULBP1	2.412397518	2.319197351	1.402530394
SLC43A1	2.123484388	2.317108115	0.960310258
TDRKH-AS1	2.366850566	2.316328082	1.563330048
ACTG1P24	2.110176197	2.311025415	2.263296593
MIR1236	0.391366658	2.265108799	1.87175294
RPL18P11	2.697601049	2.261855452	2.016519473
NUDT9P1	1.746927481	2.260103063	1.929532879
MIR761	0.727812859	2.234964123	1.453717967
SERHL2	0.664121786	2.231039244	-1.282876432
NPM1P32	2.174412169	2.225247792	0.693925056
LERFS	1.191996868	2.221066057	1.288285383
ATP5F1AP7	2.464794458	2.218861631	0.568932906
SOWAHD	2.045408021	2.214466783	1.750177706
GRAP	0.170261754	2.21021668	1.308333293
BACH2	1.934750979	2.200664853	0.068690368
MIR30C2	3.506222064	2.199400295	3.562247971
SEC16B	1.328010116	2.199002838	-1.822177754
SLC13A3	1.172681933	2.187734349	0.193038001
THEG	2.673021343	2.181749485	1.194602169
CYCSP39	-0.659908936	2.178405359	2.673451131
LOC100133007	2.307305772	2.16245584	2.862588888
MIR600	0.605980282	2.160822794	2.166897309
HNMT	-1.308470476	2.14795501	0.811696469
BEX2	1.967120422	2.141373498	0.520957971
MIR3189	2.278338022	2.125713521	2.349822198
DEPP1	1.601651737	2.124855445	0.605511344
LTB	1.898298268	2.1116942	1.304911357
NUDCP1	1.840840225	2.086894002	1.698829681
MIR3615	2.094312607	2.080612863	1.104151424
LOC101927040	2.78268923	2.079368522	1.373053252
PCK2	2.026403661	2.078690874	0.836717062
DEPTOR	1.910661958	2.066545391	0.761795255
BTF3L4P3	1.774215426	2.066093157	2.89673918
DBF4P1	0.220494638	2.064860394	1.681167531
RPL36AP46	1.366338849	2.057651706	3.166857147
LGALS8-AS1	1.194979365	2.046304447	0.157115437
CLEC4E	1.828284527	2.026882611	0.135554888
MIR12127	-0.048156038	2.019899557	2.302858132
PCDHA1	2.166652755	2.014476819	0.007809209
KIF1C-AS1	1.08925484	2.010627824	-0.315867693
PSMC1P10	1.327610375	2.006902206	0.734476358

DUX4L8	-0.0719729	2.006125028	-2.45467108
TCP11L2	1.786161761	2.004439293	1.102226713
PSAT1	2.004954936	1.994283386	1.037021676
SPRY3	0.534101267	1.982510415	-0.125316404
SUMO2P3	1.822013609	1.982088013	2.229153663
ZNF396	1.621761271	1.981921142	1.752257339
MIR22	1.587921876	1.970823425	2.107021
ENPP4	2.049199364	1.968117777	1.045880052
TNFRSF9	1.852792731	1.967881609	0.073492696
RGMB-AS1	1.540278859	1.953529035	0.994908469
MIR658	0.835213722	1.947246558	-0.716942634
RN7SL566P	-0.275441066	1.942622515	2.35766444
LOC152594	1.665783032	1.93843114	1.793903469
RPL21P46	1.053788456	1.931145753	0.994937228
ICAM1	1.783736853	1.930853369	2.051574251
SCARNA5	2.536666362	1.923386887	2.205617247
EEF1A1P10	3.032209335	1.920448701	1.76380364
LINC00663	0.807206683	1.915324739	0.227821163
URGCP-MRPS24	0.201326082	1.912521762	1.608065059
GNG12-AS1	1.936880385	1.902228619	1.16156493
MIR936	1.839535328	1.901819606	-0.440572591
ALDH1L2	1.766549328	1.900985158	1.099858405
TRIB3	2.02901893	1.896375009	1.750801687
ABCG1	1.899072494	1.89231458	1.718546066
KIF21B	2.049119323	1.890770657	1.528099465
MIR1909	0.931181815	1.885636018	0.966547542
LCN12	1.165442391	1.881925456	1.146586563
H4C2	1.494470389	1.880032877	1.41260628
MIR6858	1.565528078	1.877159557	1.126002824
RPS2P37	2.508817414	1.876233547	-0.215813554
LOC101929243	1.661703477	1.876002411	1.820938723
PIP5KL1	1.860294926	1.861657447	0.686566535
PHGDH	1.837848889	1.855034589	0.841452726
MYO16-AS1	1.581860674	1.851177685	1.646370195
TF	1.562764537	1.847615418	0.319007137
PRDM8	1.969047848	1.847513313	1.703057403
SNORA28	-0.948025362	1.845967232	-0.149685343
ZC3H12D	0.409595414	1.832508782	-1.145018996
GPT2	1.898564595	1.831033699	1.339627355
HNRNPKP2	1.748000864	1.831016108	0.131409538
MUSTN1	2.225541256	1.817456577	1.291025314
SLC6A9	1.783131146	1.81559054	0.875411772
bA395L14.12	1.028038724	1.805949206	1.846306762
RPL26P4	2.048109166	1.802864934	1.152064268

MIR4648	1.799553895	1.79662549	1.075885514
LOC105371814	2.014001624	1.78019418	0.385826009
ACTG1P14	1.477092956	1.778671211	-0.222609309
CXCL3	1.626171015	1.777565301	1.865190939
RNU6ATAC	1.494963479	1.777310218	1.603375559
FEZ1	2.304522823	1.774599013	0.258309104
DNM1P46	0.029338852	1.77180404	-0.308422733
SNORD134	0.884079911	1.771207093	1.897549975
RELB	1.683565233	1.768867442	1.075113262
LOC100421160	0.747295462	1.768116971	2.225234014
ANKK1	0.569911369	1.767893364	-0.230530688
BGLAP	2.076206015	1.765437405	1.091912777
GPR146	1.105591095	1.754979513	-0.004918847
LBHD2	2.052818633	1.748880176	0.476587154
LOC100506688	1.628290316	1.742441976	0.976028013
AVIL	1.547849937	1.739153144	-0.159743921
LINC01869	0.889622215	1.737540424	0.832429828
TMEFF1	-1.792583285	1.735722705	2.134249169
IRAK2	1.626983413	1.734761725	1.384299424
ZMAT1	1.343451967	1.733505669	-0.173156781
LOC101929536	1.886921859	1.725973982	0.794697787
DUSP6	2.203441824	1.725013986	1.649385456
BDNF-AS	1.915091681	1.723287518	-0.067420998
RPS6P25	-0.333886117	1.720289704	1.296806142
FLJ42351	0.667417048	1.709122594	0.416369055
APOBR	0.834412703	1.707126557	0.481448436
RPL7AP45	2.029445768	1.705960541	3.408150407
HNRNPA3P1	1.196620764	1.698810667	0.490773129
LOC284009	1.092629568	1.696365178	-0.105725671
MTVR2	1.625503377	1.694627908	1.459962575
HMGN2P28	0.337530725	1.692818883	-0.650463624
PRR26	0.971967455	1.692696508	0.684318389
LCA5L	0.132299176	1.692459751	0.54592058
PTGER4P2-CDK2AP2P	0.898642993	1.684622924	0.148278418
MIR636	1.454847204	1.673595221	0.953120379
ATP5F1AP1	2.227468025	1.669625958	0.925062488
LY6G6C	1.80501336	1.667848808	0.801967219
AP3B2	1.571827274	1.656622537	0.814705422
PCBP2P2	2.407122395	1.654128863	1.770995039
EIF4EBP3	0.607514948	1.65323476	-0.218413094
SDR16C5	1.919583779	1.650013527	1.002212666
ATP5F1AP10	2.137925704	1.645763134	-0.063089783
SNORA31	2.277905371	1.64379655	0.55673452
NPHS1	1.136942133	1.638963775	0.218750925

OTOF	1.376802629	1.632631228	0.973112583
LOC100419799	2.335344032	1.629143995	2.086477965
SNORD20	2.155618726	1.628830863	1.745510459
ZNF205-AS1	-0.659401117	1.624611787	1.112063567
LOC101927989	1.740688932	1.623242063	1.637108102
LINC00933	1.946779977	1.621561796	0.416968338
CHAC1	1.915635069	1.620662067	2.607663509
OR7E154P	-0.843645917	1.612463327	0.407105229
MTHFD2	1.713166808	1.599431866	1.104579374
PTGES3P3	0.423462136	1.596166821	2.024924729
HIPK4	-0.975889464	1.595786703	1.989188402
C5AR1	1.559090362	1.594127457	1.025866458
TLDC2	1.179664012	1.583286679	1.226728798
LOC100420326	1.179017031	1.577458866	1.88582606
SNORD102	0.214621237	1.576801444	1.722059483
SLIT1	1.781829369	1.574737669	-0.08419288
NEBL-AS1	1.066168138	1.557372473	0.667995221
CYP1B1-AS1	-1.441529655	1.555261121	0.630672081
KIRREL1-IT1	2.276593267	1.55519795	1.643327472
MIR4800	0.825869248	1.548943278	2.000987809
CEBPB-AS1	0.939005452	1.548190002	1.686462597
MIR6759	2.440014165	1.538246002	0.512450001
ASS1P2	0.176114936	1.53777118	-0.052852228
CXCL1	1.264081372	1.537416094	1.482991413
CCDC81	-0.169945316	1.536178981	0.382163504
LOC100996437	1.203106193	1.532665541	0.730523206
F7	1.12918945	1.530515016	-0.057210379
KLK14	3.078171321	1.527513602	3.358683974
RAB39B	1.585785317	1.524502842	0.699823926
PLTP	1.565188591	1.524381947	0.889200162
NCCRP1	1.718238576	1.523361184	0.966783972
PCDHA8	0.610443052	1.518374873	0.977769496
SNX29P2	0.460305755	1.518329408	0.980230341
HHIP	1.310505068	1.514221565	0.797078014
ZNF385C	1.270205405	1.513900799	-0.121852688
NOS2	1.300540133	1.513406188	0.094385505
KLHL30	1.092708227	1.512466578	-0.418738269
KIF4B	0.005265957	1.512448188	0.117964064
SEPTIN14P2	1.083257764	1.5115339	1.265451206
NOS1	1.234469456	1.510861641	1.312550932
PCDHA7	1.782459348	1.508451679	-0.100679968
CCR6	0.656090017	1.50812634	-0.4346874
MAFF	2.125486796	1.507089729	2.130612528
MIR7109	0.296125728	1.505946752	1.859627009

LINC02649	0.318233792	1.504803622	-0.365413922
ZNF460-AS1	0.996342199	1.504801051	0.242769687
ARG2	0.706620828	1.504636803	-0.029990111
XPOTP1	1.724906226	1.500151182	1.54657931
PARP8	0.810906937	1.499259151	0.88305985
C22orf23	0.986215899	1.497671468	0.891850418
SNORD141B	0.791295666	1.496041127	1.692806678
PCDHA13	1.343542976	1.495987531	1.721447043
SPRY1	0.966547405	1.495512944	-0.49533204
NUDCP2	2.066347312	1.49511039	2.085882567
FAM83E	1.559545555	1.494117028	0.031975633
ESPN	0.272270777	1.492060571	1.338212209
UBBP1	-0.442099101	1.491779644	1.361657585
GLIPR1L2	0.517572978	1.491547385	-0.581064597
C15orf65	1.775319794	1.486866171	1.073473831
LOC101929188	0.857067771	1.485282188	-0.249966232
SPRN	0.169675382	1.484900107	0.247233121
MIR1182	1.099276461	1.484630039	1.482691055
LOC101927267	1.172722578	1.484219137	-0.198838676
SUGCT	1.452682321	1.4784835	0.569448366
CALHM2	1.192168465	1.478328593	0.511111999
TLE1P1	1.465444313	1.471584902	1.216285714
LOC100131465	-0.118359398	1.469674796	-3.426829395
ZNF91	1.472952291	1.467978619	1.178259779
LINC01694	1.271482715	1.467825383	0.967778211
LOC102724562	1.096599273	1.46748654	-0.014910861
ICAM5	0.895661731	1.463865976	1.239140807
CXCL2	1.438890085	1.463175711	1.487052114
RPL10AP9	1.790289676	1.462910915	2.737677481
ATG9B	0.545636181	1.462696323	-0.08752947
NFKB2	1.450281584	1.462125915	1.17625291
KRTAP4-1	1.642446375	1.456562304	0.782188311
RNF138P1	0.585808904	1.45645978	1.141834733
PIGR	0.469550242	1.452882057	0.08215519
HSPA8P1	1.96037858	1.452185766	1.278583233
ZCCHC12	1.948969891	1.451940047	1.860467606
CELF5	-0.193242924	1.451888371	0.603711721
GGT5	1.236518559	1.449862817	-0.520153732
RPS27P17	-0.636298543	1.44634907	-0.39799507
AZU1	0.532063732	1.445005392	1.008184366
CYP4F2	1.167885449	1.442775866	1.264447971
SETP22	1.50825146	1.440428239	1.61091004
ACRBP	1.391423787	1.438092761	0.261416658
MAPK10	1.509368816	1.433413218	1.270916311

KLHDC7B	1.977483451	1.432374588	-0.081886958
LDHAP5	1.63275884	1.428876832	-0.309873482
YPEL3	1.88569369	1.425599603	0.055803661
CKLF-CMTM1	0.53505502	1.424889451	-0.458039534
FGF18	0.248444966	1.423616137	1.590655456
LOC100420109	-0.161189886	1.421199336	0.965263736
LAT2	1.732041542	1.417023068	0.169836529
PCDHB16	1.757761137	1.416287254	-0.609888333
ERV3-1-ZNF117	-0.769455116	1.414274439	1.169605109
ANK2	1.35344397	1.413442318	-1.044534284
AKNA	1.126283613	1.413207692	0.740976139
GRM2	1.138191694	1.412898107	1.286475729
SYT5	1.379451328	1.411637016	1.328497612
TACR1	0.64144001	1.411605467	1.17644144
PCDHB10	0.84470148	1.405617687	0.989456249
NATD1	1.31919667	1.400832942	0.165640161
LINC00261	1.06347666	1.397754425	0.143817416
FBXL16	1.022149904	1.396704479	0.226358788
POM121L9P	2.1712179	1.39356795	1.398740205
EFCAB10	1.54159862	1.389447433	1.791563205
SPEGNB	0.070400121	1.387714448	0.565756997
LOC101929066	-2.675984614	1.384324753	-0.178673199
CASC19	1.61335686	1.3836603	0.501259168
CHRNB4	1.100109392	1.383056605	0.887134511
PCDHGA7	1.551195371	1.378828822	0.247377624
ALOX12B	1.016101756	1.373369898	0.845093158
RPL9P11	0.066623128	1.372980118	-0.099409097
RPL23AP8	2.048402264	1.369004691	0.583102176
MIR4690	0.381609314	1.367308412	-0.229218335
RPL26P36	1.430445338	1.366628887	0.751750004
FMO5	0.036245378	1.362886659	-0.629004874
NKX2-8	1.268659913	1.36173891	1.262029989
C8G	1.862335965	1.357315181	0.785730696
PCDHB4	0.375557172	1.356161104	0.216371611
SLC26A10	0.858345322	1.353270362	1.188911346
HMGN1P29	1.876568114	1.352816325	0.387049301
ERN1	1.428587199	1.351903486	1.005281383
RPL7P24	0.78188493	1.345988483	1.501315001
ARHGAP9	1.965589124	1.344849076	1.043168649
DOK7	0.570594018	1.343555504	0.427759683
MIR429	0.120476437	1.342126524	1.537358896
DGCR9	-0.278899752	1.341216707	1.212874148
SNORA11F	0.834617947	1.339268898	0.485340745
VDAC1P8	0.41372299	1.337792477	1.373493064

IFNL4	0.756400577	1.337217879	0.658792762
LINC02447	2.024102159	1.336543515	1.008013046
PCDH1	1.619779327	1.334896935	0.696603811
HNRNPA1P27	1.841303193	1.334511629	0.458637283
GRIN2C	0.916970308	1.333440722	-0.132390691
LRG1	0.297841275	1.333346801	-0.000454993
NDUFA6-DT	0.096705196	1.332021452	0.689019185
MIR3605	1.634242032	1.329636178	2.402995002
LINC01152	0.746629284	1.3292117	-0.896458592
DNAH2	1.408444822	1.325529117	0.204533156
SLC1A5	1.332106205	1.319608934	0.596823819
HDAC5	1.20711485	1.319089724	0.227124588
SLC52A3	1.812987787	1.318904219	-0.303344593
LOC100420429	1.653923148	1.318137745	1.192813655
CXCL5	1.386896213	1.317475502	0.917232481
LGR6	1.173491505	1.313759219	0.212705911
OSTCP7	2.611914282	1.313660479	2.218736892
MIR6717	-2.089915511	1.31272662	1.47661034
MIR6766	0.813827069	1.311570695	1.110798948
IL20RB	1.214944801	1.30949591	0.073355504
BHLHA15	1.339573344	1.306951375	4.70889103
SEPTIN14P16	1.381371725	1.306052715	0.868634199
LINC02762	1.231511648	1.305429716	1.052762917
ITFG2-AS1	0.668329004	1.30317229	0.647612288
ZNF670-ZNF695	1.094392295	1.302791321	-0.510190643
MIR3198-2	0.71456104	1.301794169	0.290361421
HCG25	-0.400865031	1.301213613	-1.054030961
MIR6751	1.653746355	1.299604956	0.044192008
VCAN	1.144718139	1.298600167	0.685192565
CBX1P1	-0.280294158	1.297740417	0.725617699
LOC646890	1.404849038	1.297260673	1.682953923
SPRY4-AS1	1.623835445	1.294982025	1.085848902
CYP21A2	0.140034617	1.291707819	-0.048147339
LINC01144	0.906598011	1.28569207	0.320273869
NKPD1	1.238978441	1.285422473	1.146242893
XKR5	1.666182303	1.284080163	0.403774249
RNASEK-C17orf49	1.872866551	1.284018277	1.397349471
PTPRVP	-0.605535831	1.281792619	-0.01708463
ASS1	1.036522928	1.280864747	0.506712361
RPS26P53	-0.86345703	1.280046345	2.267986025
CCDC30	0.143342846	1.279488815	0.777134962
ZSWIM4	1.216356945	1.278938388	0.918334868
MIR99AHG	1.475130663	1.274219639	-0.88675445
RPL7P6	-1.473512623	1.27340108	-0.139595303

TTLL1	0.983880097	1.269300011	-0.015338766
EIF4A1P13	-0.389242819	1.268606448	0.872434235
LOC791126	0.871916924	1.2668162	0.785209822
NME2P1	2.157325214	1.265764924	1.966120785
EFNA1	0.909202406	1.265001883	0.246362349
CCNA1	1.279767077	1.260556145	0.758763062
ATF4P3	1.479837274	1.259159714	1.731850964
HIC1	0.795892122	1.258568569	0.393070226
RPL17P18	0.97192036	1.258338397	1.106454985
LINC00540	1.405717682	1.257230992	0.729209869
MIR4685	-1.791873906	1.256546746	0.429000138
STEAP1B	0.994916356	1.255621375	0.089775903
RENBP	1.243520069	1.255196373	0.498702365
PARGP1	-1.094253574	1.255014229	-2.633831742
PTMAP1	1.164525613	1.253344448	0.827555299
PROX1	0.655867512	1.253278582	1.06871275
RNF103-CHMP3	0.856913423	1.252585862	-0.127311798
GARS1	1.355462003	1.248918173	0.805787197
SOD2	1.117555508	1.248561064	0.00519234
LOC101927635	1.021381519	1.247475667	0.05089637
ATP6V1G2	1.436658657	1.246378047	0.399881514
KRT8P36	1.135372444	1.246313681	2.128588416
KLHL32	0.051269834	1.245940885	1.516709677
MIR4709	0.368593383	1.245561311	1.264383146
AARS1	1.197908954	1.243737822	0.774772945
DDX11L10	0.250501016	1.243487067	0.883346968
DQX1	0.704256224	1.241726827	0.429146829
ETV5	1.350647701	1.241659233	0.992019968
ANKRD20A17P	1.376406288	1.240369702	0.760980102
USP17L14P	2.954381711	1.238315919	4.678685471
KRT8P33	1.139338244	1.237999901	0.897228132
SPATA41	0.556392373	1.236495255	0.8223038
MIR4750	1.052719557	1.233715533	0.442435788
LFNG	1.007978578	1.232520003	-0.335332168
RUNDC3A	1.245479228	1.232409839	-0.162810702
H2AB3	0.348952028	1.23142192	-1.255400378
TMEM121B	0.388656096	1.230564388	-1.099624309
FXYD6	2.12969753	1.228774044	0.645715253
FCRLB	1.136585747	1.2160328	-0.288768032
EIF4EBP1	1.29947436	1.214432209	0.430930471
CCL20	1.131146007	1.212615577	1.768227231
TMC4	0.844841097	1.212611991	0.363255155
SPRY4	1.564561723	1.212556233	1.709167461
ITGA9-AS1	-0.15187192	1.210381266	1.128190531

EMC1-AS1	1.891377696	1.209893184	2.125875666
ULK1	1.128399022	1.209351032	0.625183685
RPS21P1	0.212775996	1.20834412	-0.941012001
TNFAIP3	0.966467012	1.205822381	0.597918279
UBALD2	1.328811271	1.202728858	0.203399669
ZDHHC22	0.527734585	1.200693927	1.47820497
EXTL3-AS1	1.311290892	1.200058326	1.177724922
RAB37	0.91325913	1.195729132	0.666562214
MIR6743	1.5669352	1.195546405	2.303477881
CYP2D8P	0.775674104	1.193505476	1.272656274
CLDN1	1.331740463	1.193341994	0.283790972
DRD4	1.397005781	1.193140302	1.072844714
SNORA22	1.190257982	1.192508408	0.56104423
GAL3ST2	0.799800083	1.192350718	0.975051074
MUC17	1.016861036	1.191898961	1.262523947
CARD9	1.279031533	1.189665689	1.661096363
LOC727896	1.400849964	1.188929905	0.414094869
CAPN5	0.994187246	1.187756197	0.161252562
NBR2	1.21472485	1.185650896	0.599102527
PLA2G6	0.87922466	1.185000424	-0.052162643
SDAD1P1	0.892431446	1.184540075	-0.580062193
LOC100287846	-0.059221202	1.184226999	0.516899056
CARS1	1.238112827	1.183483302	0.795983834
PRH1-TAS2R14	-0.705240373	1.183305075	-1.185855614
PRPH	0.873974715	1.183112711	-0.18140672
TAS2R19	0.349084162	1.181901624	0.3172197
RAET1E-AS1	-0.202154274	1.180989808	-0.749151345
SEMA6A-AS1	-0.753636371	1.17726143	0.560870567
POLR3GP1	-0.877448899	1.175557015	0.895091356
LOC100505585	0.150974049	1.175212378	0.53177692
RPS6P9	0.610272314	1.174560568	-0.890582429
LINC01569	0.952180656	1.174510908	0.135273957
CNOT6LP1	0.954370967	1.172116864	1.512843472
CYP21A1P	1.307619529	1.171360931	0.005046909
SNX18P1Y	-0.343560047	1.165360184	2.727763241
NUFIP1P1	0.441154661	1.163429895	0.644606839
PIK3IP1	1.27935563	1.163247079	-0.381117554
RPS10P5	-0.833189547	1.161555464	0.011759592
TCIM	1.007135489	1.15711324	0.826493245
IL9R	0.464308521	1.157023947	0.188643107
RPS6KA2	1.446706782	1.156951041	0.388276587
MIR6772	1.020232813	1.156526426	0.343366819
SNORD7	-0.14244569	1.155280248	0.118952653
TEX19	0.816857719	1.153721509	1.027389807

FBXO32	1.05023974	1.153229447	-0.19444172
RN7SK	1.062241487	1.153003571	0.104891101
TMEM202-AS1	0.646725727	1.152920541	1.410492578
RASD1	0.745861164	1.148120402	1.811994393
PNPLA7	0.982611981	1.14775654	0.316591413
HMGN2P46	0.692402897	1.144970236	-0.085433867
UNC13A	1.490637717	1.143303287	1.026072376
SPRY4-IT1	1.850981156	1.142908078	2.047799637
CCDC114	1.434453823	1.141672715	0.054569427
YWHAQP6	0.534477734	1.137918509	2.761584895
MIR639	1.826814357	1.136922797	0.572226605
SLFN5	1.26495596	1.134299761	0.823708358
G0S2	0.562499071	1.133895355	0.293396507
LINC02535	1.019961797	1.1333422	0.175086613
SEPTIN14P6	0.863104284	1.132501784	1.09073926
NTSR1	2.103830202	1.132301797	1.883410509
CFAP54	0.955817811	1.132114351	0.842435106
CYP39A1	1.313745179	1.129428378	0.642627882
PLK3	1.303079382	1.127692663	0.867372857

Supplementary Table S11. 500 ea up-regulated genes based on TLR4 in H1299 cells

Gene_Symbol	Fold change (TLRs vs. vehicle)		
	TLR2	TLR4	TLR7
MIR5090	0.447458977	14.76949508	0.299560282
MIR4469	0.584962501	12.42010897	2.237989276
MIR611	-0.446790558	12.19040344	1.002508517
MIR6861	1.321928095	10.83819094	13.69359585
MIR34A	-0.683961127	8.87599143	14.88834235
MIR3940	3.703350844	7.95368076	2.814854986
MIR10394	2.861530197	7.186663328	2.10203985
SERF2-C15ORF63	0.65720901	6.404445568	1.55452311
RGCC	4.506800817	5.587387632	3.409382002
CATSPERB	4.548904951	5.258747891	5.572652256
DNAJC25-GNG10	0.102158001	4.769055089	2.092467193
SLC13A3	-3.119205944	4.732344934	0.720477471
SNORA86	1.569443083	4.724714464	2.922197848
ADORA1	4.70110749	4.683755215	2.816682636
RASGRF2	2.284561835	4.676123533	3.689372168
RPL10P13	2.025464628	4.485127066	-0.821486247
TNFRSF6B	0.167566769	4.471039087	-0.343916001
RPS12P3	-1.869896859	4.426005867	-0.72502471
JMJD1C-AS1	2.026365143	4.377881937	2.395240818
MIR29B2	-0.378675296	4.344682851	-4.34410581
MIR6717	-0.337034987	4.189960581	9.809342562
MIR5001	-0.833323167	4.106096904	2.051053867
GRAP	4.104498992	4.06491423	4.066941074
ANKRD22	4.216921296	4.036535747	3.400686485
MASCRNA	0.304854582	4	0.434402824
FAR2P1	2.921162794	3.876255369	4.876789786
RPPH1	2.795572485	3.862917075	2.880286076
CHKB-CPT1B	1.888182466	3.861007682	0.601608517
COL26A1	3.277047706	3.839431112	2.995194802
TC2N	3.974802525	3.779394119	3.987394994
OLFML3	4.277099042	3.776344223	4.109535313
HHIP	3.749292816	3.668328287	4.048338873
H1-3	2.449299406	3.637293161	0.000617741
SNORD118	-0.370022383	3.576239082	-0.629154074
PPIAP42	2.319551016	3.570981308	1.167030423
SLITRK6	1.901689192	3.555651109	1.927280903
TGFA	3.463682548	3.553403968	3.229606397
HNRNPA1P33	2.930235002	3.518128206	2.113525665
VGLL3	5.34335333	3.482383661	4.424480687
RNF103-CHMP3	0.277495276	3.480862865	4.663771975
VILL	3.732761472	3.45319649	2.756011566

TGFB2-OT1	2.507717926	3.446704571	-0.95790646
HNRNPUL2-BSCL2	-1.482952583	3.443561666	-0.483326663
ADAM12	3.800634487	3.42564738	4.176119892
CKS1BP6	-2.505922933	3.411103032	-3.094353007
ACOT12	3.371201219	3.399137652	3.003857713
TRS-AGA2-6	5.584962501	3.387023123	-3.532495081
RMRP	2.154526003	3.38534358	4.466412598
TRL-AAG3-1	0.849301828	3.378460325	1.556104683
HMGN2P1	0.491483846	3.335927468	-0.472749434
MIR4737	-0.731004036	3.318383388	1.818919674
MAL2	3.14517484	3.294041855	3.624418878
C10orf90	3.294961503	3.275774932	3.086863298
NNMT	2.815201813	3.230155659	2.797932209
AGAP5	1.341458956	3.228627263	1.162486712
FUT9	1.713578284	3.209117317	3.377180366
NPM1P35	-1.021224507	3.197981354	2.417507854
TESC	2.965102106	3.16525331	2.392320758
RPL29P24	-0.56262876	3.136406459	-0.508136529
USP10P2	0.103355568	3.131976266	0.887554852
RNU6-45P	0.709752425	3.103201847	0.038336802
RNA5S6	0.456744601	3.101502118	2.487929315
SNORD153	0.839145989	3.069280841	0.52756019
MIR664B	5.023846742	3.065643616	4.209453366
ZASP	1.349558685	3.030412824	0.79062996
CASP1	1.785339094	3.021508308	2.739729731
IGFBP3	3.309400737	3.018586713	3.053909257
LOC100505915	2.055076828	3.014799202	4.045661833
TVP23C-CDRT4	6.113169477	2.99146021	1.246361187
BMPR1B	3.08382596	2.989896717	3.9009104
RPL36A-HNRNPH2	1.252241884	2.982427285	-0.22971876
CTNND2	4.219432215	2.973701295	3.356392381
ANGPTL1	2.312799902	2.970135383	4.281770968
SOST	2.916147402	2.937280564	2.840679002
TCERG1L	2.654173979	2.933001917	2.341526586
RPL7P6	-0.134963986	2.916233517	-1.077228326
EMILIN2	3.141183506	2.908819657	3.654664773
RPS2P40	0.289619426	2.90687683	3.806533009
GKN1	2.17237726	2.898600355	2.606119557
NPM1P7	0.796025985	2.888491524	2.43183432
SH3GL3	0.7948202	2.887371784	1.05618143
LOC100131471	-2.430903814	2.874043048	3.335762857
THEMIS2	2.662135748	2.873236995	2.217054684
CLCA2	0.630218666	2.842321909	0.329847758
GJA5	2.946737626	2.832821651	2.698643217

MIR3614	0.821029859	2.825166198	1.185555653
UBE2F-SCLY	-0.186610005	2.819001833	-2.113126876
MRPL42P5	3.290045509	2.813860542	1.333331432
H2AC21	0.574302674	2.790516846	-0.894306625
TMEM255A	1.468767871	2.780829757	4.601560548
MIR600	-3.192848146	2.77340759	-0.208345919
MIR4775	0.09606741	2.760740228	0.968402262
LRRTM4	2.609202222	2.747778588	3.29629
RPS4XP1	0.234639258	2.746335241	-1.001568903
ST13P6	-0.676424229	2.745771337	1.49051748
EPHA7	3.152885253	2.743753255	4.005248009
LINC00326	2.268228356	2.739741644	2.37298339
TSPAN1	1.489203076	2.736772345	0.566206341
SLC26A9	2.903004177	2.732752546	2.827143785
GPC3	2.842094279	2.719327079	3.608783281
CYP27A1	1.181004697	2.716609344	-0.094896892
SNORA72	0.932165709	2.714289439	0.397367688
LINC02861	1.689572625	2.708070477	1.594846369
FRMD3	2.90548667	2.695586644	2.915072597
FABP5P9	-0.764336966	2.686172287	2.586742289
SNORA56	-0.508483404	2.677079228	-0.579953779
MIR4763	1.819756844	2.669646875	-1.178841716
MGAT4C	3.080959507	2.669117606	3.573699807
RNU1-1	1.257653128	2.635806899	4.440778817
SNORA12	-0.694782524	2.635084975	1.047763368
RNU1-3	1.683433194	2.62478696	3.776639965
LMO1	1.889507521	2.622365951	1.152787902
SNORD7	-0.295624674	2.622204394	1.726400299
SNORA71D	0.142339376	2.619705882	1.102549106
TGM2	2.751127554	2.60179992	3.035536278
ITPR1-DT	-0.672445462	2.601138283	0.705018162
C2orf27B	0.433928598	2.596649385	-1.260181985
AFF2	2.97697642	2.593888024	2.689010954
H4C2	-0.281417836	2.592820791	0.041537173
LOC100422556	0.284569492	2.589918062	0.952766046
C1orf229	-0.20856583	2.582624469	-0.371675221
RPL13AP14	0.727264343	2.562952473	1.1340957
C10orf62	-0.445334217	2.554303639	2.496575159
RNVU1-18	1.670616796	2.545352248	4.708251242
HSPB2	2.126644714	2.544294917	2.092613651
RPS2P11	-1.442288094	2.540711369	-0.094090424
LOC100887068	-0.545277461	2.523315164	-0.681662687
SPARCL1	2.111246857	2.512172914	1.519544061
RPL17-C18orf32	-0.833220625	2.506026482	0.526414165

HCP5	1.985571953	2.480148245	0.867821579
SNORD124	-1.603370502	2.478459301	0.714581446
MIR6763	-0.104033277	2.470586067	-0.591741953
CFAP52	1.026903459	2.464149621	1.893690583
SNORA20	0.214418259	2.452970229	0.56690644
MMRN2	2.23997329	2.440616583	0.113732626
ZNF738	1.965915924	2.436126319	2.361962281
ABCB1	1.926237014	2.429556078	2.220045838
RPL12P28	0.35313751	2.425428337	0.36150875
PPIAP46	2.66833162	2.422246475	2.514880775
RPL26P32	2.577806798	2.417372873	0.248439052
LOC100129129	0.46561347	2.406444056	-1.193319809
BMPER	2.502261217	2.371564387	2.700823944
RPS10P13	1.760506422	2.368336024	-0.312652882
COL9A2	2.072959944	2.362779387	1.457752988
LPL	1.830637041	2.346144201	4.144873005
RN7SL3	0.895963108	2.341469559	2.071820925
HCAR2	2.573662488	2.336438618	1.724964735
KCNK15	1.051742395	2.324417405	1.2761545
GRIN2D	1.767488029	2.315907676	3.183137336
LOC729839	2.923363231	2.314013771	0.011037473
RNU1-4	1.376437784	2.313245723	3.671689408
MIR6729	0.715051409	2.309398639	0.169402198
FAUP1	1.47940056	2.305621773	-1.94049398
HEY2	2.18015242	2.305011398	1.912007559
RNU1-2	1.767387469	2.300864851	4.462556759
LUM	1.890351993	2.300765106	2.152593651
PLS3-AS1	0.889083174	2.297859759	0.97762304
MIR7705	0.502500341	2.280107919	-0.038474148
RNA5S17	1.408961577	2.274808164	1.486884066
BGLAP	1.158210199	2.265338258	-0.071780523
SCARF2	2.163008073	2.261775446	1.21420031
LOC101927830	-0.402465944	2.25443163	0.232271698
NPIPA8	0.56719208	2.253280141	0.893955673
STRA6	2.280148187	2.245950347	2.261031208
MIRLET7D	2.410005875	2.243333378	0.974225325
IGSF1	1.83958907	2.240044066	2.665091123
MIR6834	0.11284	2.234013377	0.722525481
EHF	1.10944137	2.208566131	1.949415608
MRC2	2.442304324	2.208277489	2.741141084
KRT81	2.1070417	2.205740185	1.736753075
SNORD3B-2	0.243873877	2.200772084	2.321306888
SNORD51	0.581975014	2.199328877	-0.347259682
SEMA6D	2.521399818	2.195215757	2.679309499

PLAU	2.095071554	2.175283877	1.940665714
RPRM	1.545970011	2.16991949	0.821796045
NAALAD2	3.333933191	2.166619785	2.492897866
EXO5	1.931359233	2.165858624	1.219301266
FBLN5	2.051543351	2.164772855	2.248563729
PTMAP12	1.813870867	2.161491336	0.828865388
IGFBP4	2.22633503	2.160009738	1.765665204
C3orf49	2.182835197	2.159760116	1.892395933
HMGB1P3	-1.413360375	2.156421431	-2.248882934
CENPVL3	0.739611814	2.154845002	-1.310285245
NT5C3AP1	0.825458631	2.154773852	1.002066204
GMPSP1	2.970655035	2.154075383	0.40038742
RDH10	2.178145895	2.144793916	2.515786034
MEST	1.926362031	2.14477938	1.966504792
RPL26P4	0.903405574	2.144459672	-0.418121751
SEMA6A-AS1	0.091046866	2.137563072	1.217538371
FAM181B	2.107095231	2.137487529	0.80333732
MIR4793	0.602664502	2.135436625	-1.915387772
SNORD3B-1	0.75386573	2.120318441	1.946401177
ACKR3	3.386805365	2.113824794	1.61158005
LOC100287467	-0.980135515	2.11329228	0.498894249
FOXP2	0.997862426	2.108710886	2.455425719
MIR29C	1.25968625	2.104988872	-0.724716999
LIPG	2.324467662	2.103600187	2.367189409
LOC100128086	2.349358639	2.097165241	0.770228502
CCN4	-0.110341627	2.092266963	1.754064681
IL1R2	1.140370085	2.091841379	1.504396589
MIRLET7B	0.078369889	2.084277586	-0.992528751
ODC1-DT	0.22454059	2.080879523	-0.729500868
LINC01351	1.225841599	2.078892309	0.598570239
LOC101929128	2.07224241	2.077923417	1.733757113
ID4	2.309014218	2.069773754	1.856975252
SNORD138	2.245128296	2.06729062	0.49530423
GABRE	2.272298348	2.057826119	1.968026251
MCAM	2.242894082	2.056603603	2.323306514
GLT8D2	2.253493219	2.037059081	1.013832022
RORA-AS1	-0.655770537	2.033307384	0.46647959
PCF11-AS1	-0.061700524	2.033221067	1.343873776
SLC35G5	-0.062150829	2.027665037	-0.697852376
RIPOR2	0.466512578	2.023900947	0.049184012
SOX7	2.21415192	2.022822486	2.216007233
FLJ22447	1.954468384	2.02203948	1.937759854
MIR6777	0.609097018	2.013618296	0.033621078
H3C2	0.311885547	2.012003417	1.187734532

RNU6-1099P	-0.970090076	2.010733247	-0.118005937
HEY1	2.063788969	2.010029782	1.885409914
RN7SL4P	0.950570514	2.006840854	1.519243592
RNA5S7	1.673454594	2.003748861	1.008725136
HPD	1.852162782	2.000595946	1.6705732
MIR4426	1.526594834	1.996251298	-0.569626292
ATP6V0A4	1.023864291	1.995549617	0.342878976
DSE	2.240339033	1.985757034	2.316108785
HHIP-AS1	2.839871128	1.981595575	1.460752934
MIR4482	-2.791073692	1.981408284	1.463815285
HBE1	0.978022778	1.97954653	0.255043594
MMP2	2.666156458	1.978597356	2.886709139
TRABD2B	1.394854893	1.978525786	1.840289225
RPL39P34	-0.105580956	1.976428329	-0.208107697
SNAR-B2	1.420120679	1.975688711	-1.619896612
SNAR-B1	1.870934816	1.975688711	-1.271978275
AGAP1-IT1	1.800593999	1.962627297	1.06694159
LOC100507564	0.031991762	1.958666718	3.624044569
CYP24A1	2.146321538	1.955205859	2.408775778
RPS9P4	0.25272559	1.954921644	0.86073463
RPS2P35	-0.260552792	1.95456344	4.040428214
MIR663A	1.638057743	1.9344801	3.001211681
LOC441241	5.087462841	1.934257797	2.005817837
STK24-AS1	-0.104250828	1.933674005	0.21951785
YPEL3	1.05644276	1.931916267	0.929673301
TCAP	-0.163595581	1.931238411	-0.162575369
EEF1B2P2	1.022294448	1.924384216	-0.321420805
SNORD3C	0.885059759	1.920099537	1.24886868
CHURC1-FNTB	2.428266016	1.91897995	3.190191234
CKLF-CMTM1	-0.226017383	1.916371075	-3.268671462
LOC100507250	0.957447561	1.910079322	1.131540683
MIR4680	1.124258103	1.902702799	2.664396968
MIR573	3.050136951	1.898600746	1.72988958
LOC100420528	1.960693043	1.897576181	1.31113326
MIR24-1	-0.243074742	1.895807134	0.190230983
HES1	2.210588221	1.895311003	1.361770684
CXCL14	1.96899941	1.893084126	1.022305434
S100A3	1.13766551	1.889560325	0.978725863
USP10P1	0.728107181	1.874542305	0.65436568
ANXA3	1.351294587	1.874071988	1.15957442
SNORD3D	-0.111484135	1.871274428	1.715178451
OLA1P1	-0.348663887	1.870166097	0.293701542
COL14A1	1.831182665	1.866614191	2.458981865
RPS25P6	1.183094343	1.857885641	0.795390554

TLL1	1.836666637	1.848029717	2.21145119
POU3F1	2.097506494	1.847766646	-0.068135156
RNA5S13	1.268847431	1.844986599	0.878150109
CPA2	1.585239725	1.841957189	2.284330976
HAPLN3	1.79068798	1.835668316	1.208023987
NRADDP	0.726836877	1.832157066	-0.126688696
TMLHE-AS1	-0.267963878	1.827737874	0.441821165
VAMP8	2.941815563	1.82452468	1.087745473
MIR4709	0.127073562	1.823599257	-1.072303599
MICALCL	1.916751454	1.815126271	2.77050167
LOC152594	0.639000412	1.812959506	-0.339605099
HOXC13-AS	1.969460972	1.812472569	1.078909591
PPP1R3B	1.956688981	1.806045649	2.537301635
HMGN2P15	0.177603119	1.803212011	1.59324601
RN7SK	0.723500534	1.801276096	2.51863364
H4C5	-0.750161171	1.800389475	0.086939288
RN7SL2	0.782234822	1.799683603	1.543923804
FLI1	3.362972373	1.798359949	4.651182429
NDNF	0.969546373	1.797126546	1.187180127
OLIG3	1.696694858	1.796437668	0.929405504
SNORA100	1.220421975	1.795136464	0.799962788
HUS1B	1.645950275	1.790974647	1.374834529
LIN28B	2.013723949	1.779350497	2.125583171
GHRLOS	0.37384107	1.775429086	0.888621469
SNAP25	1.834384127	1.773775634	1.648520056
LOC105377102	0.912161791	1.771309696	-0.309995685
RPL21P98	3.76011272	1.770399467	0.04686101
SLC12A3	2.182740656	1.764105601	2.59057255
GP1BB	-0.715791169	1.758567145	0.762574079
GALNT4	-0.226593897	1.751037667	0.454296185
LINC01176	0.584057279	1.750698339	0.983337292
KCNK1	1.832888366	1.747084989	1.496135765
FOXQ1	1.371388299	1.727948568	1.262754597
SPDYE12P	-0.589318906	1.727203371	-0.13826594
NYAP1	0.101425901	1.727136713	0.524576213
TRNP	1.363373912	1.720757159	0.731011387
SNORD102	1.061543856	1.71848887	1.326575544
KRT87P	1.401016811	1.716131101	1.547888651
MIR7106	-1.206112142	1.710785037	1.765552954
DEF6	2.01855597	1.71007715	1.008948317
SNORD64	-4.937917491	1.705189864	-0.980308152
HMGN2P21	1.711783989	1.704356204	0.809615782
FAR2P2	2.577693013	1.701869154	2.416015966
CHRDL1	2.355653622	1.700971284	3.290634706

SDC2	1.771098103	1.697843072	2.173529695
RPS15AP19	1.133215393	1.696464507	0.559431254
RN7SL1	0.643296614	1.696198218	1.641283534
CAMK4	0.386035341	1.695451299	1.354489287
CAPN12	1.733770255	1.693800821	-1.260809504
KCNH1	2.375325266	1.693665869	3.340237575
GSTT2	1.267450533	1.693064583	1.451225982
TAS2R19	-0.045295669	1.688890042	-0.687819227
LOC646644	-2.242898568	1.687774225	1.30042613
ADAMTS3	1.881156584	1.687117388	1.509511297
NKX1-2	1.687093799	1.668104562	-0.381558381
MYO16-AS1	0.0541404	1.663059314	0.475991138
MIR564	0.401709513	1.659147982	-1.545464914
CD24P4	1.832717944	1.654980855	1.57757572
SPEF1	1.751059018	1.654653102	0.326139623
KCNMB3P1	0.065749208	1.651205749	-0.294860519
S100A1	0.87653954	1.643803888	0.137888919
NDST3	0.072550633	1.643197776	-2.023057103
NPPA-AS1	0.884711571	1.635380769	0.506975409
SOCS1	1.650629597	1.634040341	0.774934128
RGS17	0.885403633	1.629352387	1.488188201
SNORD3A	1.233519459	1.62453737	3.083108168
PDGFA	1.879960512	1.619547678	1.794741586
SNORA105C	0.938699493	1.611591529	0.366574362
SNORD38A	1.255114504	1.608591014	0.073318856
GACAT2	2.320221573	1.608081971	0.187283731
LINC00624	3.112211456	1.60161723	0.584859512
PTMAP4	1.581895753	1.601306444	1.465499423
EEF1A1P3	3.381342568	1.59803511	2.41288253
FOXD4L6	0.930801162	1.595599713	0.599012715
NFE2	1.802837391	1.595334567	1.302804774
BANF1P2	-1.374889823	1.594902488	-0.7391647
PRKG1	0.361212392	1.593010541	0.983434556
FRG1-DT	-0.174122723	1.591843853	0.412636597
SCOC-AS1	0.555796	1.587776502	0.673021694
TRNM	1.149128258	1.584286446	2.122259863
LSP1	0.554751773	1.579897048	1.334610602
MIR6807	0.670343792	1.579611368	-0.427768927
PCGF7P	-1.043157554	1.577292316	-0.327138423
IPO11-LRRC70	2.07940361	1.571441767	-0.865183417
COX7CP1	-0.33026965	1.570098419	-0.765821833
WNT11	0.574707827	1.56744754	-0.481873355
H1-5	-0.842199025	1.566623928	-1.534939085
BCYRN1P1	0.852085582	1.56049546	0.991174596

TRNQ	1.013187765	1.557233122	1.856401461
LOC100419622	-0.347761991	1.555273508	0.868804541
PILRB	1.052519763	1.552195677	0.451298831
SFT2D3	1.454486953	1.549369536	0.791019212
RCAN3AS	-1.255904159	1.542138957	0.977305125
RPS3AP20	-0.104217938	1.541944109	1.895769042
PLAC8	1.442058202	1.540702256	1.063525701
RPL39P15	2.445201264	1.538802937	-0.836112796
ZRSR2P1	-2.706208574	1.537718062	-0.991960593
RNU5A-1	-0.093326313	1.537545353	0.505287563
CABLES1	1.053373916	1.537022929	0.92856179
PRAG1	1.243413172	1.53474409	1.969431568
SPARC	0.987244335	1.532947485	-0.622697373
PHBP4	0.36913704	1.532170179	2.712845172
FAM223B	0.266895643	1.531739465	1.608455862
RNU1-39P	1.033048672	1.530859098	-2.261286428
OTULINL	-0.366553128	1.529954017	0.43977684
MYL9	2.100542357	1.528015705	1.2922343
PCDH18	0.722388522	1.524217145	1.439161854
TRNS1	1.338110853	1.522035245	0.657437365
LOC105374727	0.445705972	1.517489374	-0.512573595
MIR198	0.494395291	1.514573173	0.609007975
PTOV1-AS1	1.148600185	1.512810613	0.247450975
PTPN6	2.000094253	1.507624596	2.237194585
HYI	1.560152196	1.507587114	-0.43886041
NANOGNBP3	-1.002413148	1.50000589	0.92297353
MCC	1.520440451	1.496427373	1.964132175
RNA5S12	2.246423756	1.495963994	0.855039125
LOC100506083	-0.389024134	1.495079069	0.637305513
CRYAB	1.843110382	1.494741418	1.746253183
ZFP82	0.977428387	1.492051816	-1.155924185
TERC	1.10180274	1.491451195	2.034627833
MAP1LC3C	-0.339262592	1.491409602	-0.892695903
CPB2-AS1	-0.201722861	1.491248066	0.626216103
SNORA105B	0.753662575	1.488827357	0.554814086
SNORA2C	0.865938223	1.488257186	-0.628459922
LSR	1.044376756	1.487183797	0.421058934
HMCN2	2.319638763	1.486343115	3.685935388
MIR6758	0.90305057	1.485451904	-0.437161822
DEPP1	1.537818156	1.485235058	2.143291356
LINC01503	1.955464137	1.484818964	2.003050059
COLEC10	1.527603121	1.483492487	1.097736802
TRK-CTT3-1	0.032572005	1.482754152	-0.391861911
DTYMK	1.286406843	1.481123867	1.034689407

RNU6-7	0.415860066	1.480393655	-2.803307056
LOC100129083	0.754775231	1.48013786	1.443872364
RNVU1-7	1.643193274	1.478341922	3.229493017
IGSF11	1.756900715	1.477921656	2.046339158
QPRT	1.584916314	1.476415737	1.303257976
DENND2B	0.852345544	1.476047441	0.89940431
LOC100287049	0.156402248	1.469352434	2.433926021
P2RY6	1.704375555	1.467056154	0.958252276
DIRAS2	1.120406183	1.462633675	1.320952851
TRI-AAT4-1	1.193194781	1.462507073	-0.506591437
MIR1229	0.703815352	1.459494963	0.743806628
C15orf48	1.349445322	1.45847812	0.720098817
FUNDC2P1	0.853977021	1.454280037	-1.142439662
MIR4647	0.602017287	1.45087545	-0.052702138
ZFAND2B	1.273498151	1.448835624	1.039791641
RPL21P28	0.713092589	1.442867576	0.321118508
CERS4	1.187226029	1.440844549	0.518296903
MIR6858	2.311388063	1.437830028	0.414902701
EIF4EBP3	2.390665997	1.437631408	1.365602858
PSMC6P1	0.677362228	1.437382329	1.007773087
CLDN11	0.366380656	1.435688045	0.116327088
SNORA69	1.530600004	1.435630908	0.49021805
H3C15	0.841918568	1.429898964	0.5120507
TRNN	0.360823628	1.426863758	0.811037607
RNA5S10	2.237717743	1.426737857	1.519493357
RPL24P2	1.600042841	1.425778295	1.168142566
GBP1	1.126024658	1.423824905	0.602672819
SYTL3	0.642632721	1.422424892	-0.278900525
SNORD54	0.924038444	1.421290616	-0.120938903
STON2	0.733316055	1.420625471	1.930785782
MIR7109	-0.173334099	1.418768915	-0.235933224
ARL4C	1.024552308	1.418725844	0.776327673
LZTS1	2.323700364	1.416574089	2.296025802
MYLK3	0.391654572	1.413854393	-1.236895876
C1DP1	1.045631856	1.409575053	-1.451728423
SH3BP2	1.554469833	1.408257865	1.753472477
CP	1.734539562	1.405932075	2.497365148
MIR1227	-0.475999545	1.404395344	-0.453666549
LOC146253	-0.756489205	1.40364552	-0.960408655
SNORA57	1.20757902	1.403388363	0.578411852
TNS4	1.877810274	1.402784096	1.179022194
ZNF443	-1.658127684	1.401442069	-0.208242033
MIR6748	0.35170667	1.400533522	-0.518844008
RNA5S14	1.231580987	1.396802453	0.860487739

UBA7	-0.263602112	1.385488096	-0.144523951
HMGN3-AS1	-0.104122359	1.38382777	-0.302589331
OR7E37P	0.020210631	1.382257116	2.987983818
EPSTI1	1.002637893	1.378832224	1.101351886
RN7SL521P	0.926308753	1.378543265	-0.025655238
KRT7	1.64433924	1.377526997	0.89884766
SERINC4	-0.061315819	1.377247207	-0.283949346
HOXA11	1.218336436	1.377124855	0.807790696
CILP	1.050944936	1.375557945	-0.01219624
LOC105274304	-0.56437907	1.373901766	-1.414468915
MIRLET7I	-0.272671349	1.372175782	0.857782583
LIFR-AS1	0.351589625	1.372167723	0.204351652
MIR6768	-1.753667863	1.371828192	-0.674932973
ZNF577	1.085764088	1.370346743	-0.119532559
THAP5P1	1.386723918	1.369311125	-0.783570908
MMP7	0.699699563	1.368191234	1.714574297
BCYRN1	0.728981934	1.368133375	0.601449144
HOXA13	2.29221051	1.364180328	2.027297845
FBLIM1	0.218178641	1.363528009	0.209343086
PLEKHO1	1.314897623	1.363073534	1.311020209
NLGN1	2.083484	1.362853512	2.348500672
NGFR	-1.037750357	1.361989842	-0.805451471
TMEM86A	0.258170092	1.355538416	-0.357887437
SCARNA17	2.393079921	1.355336185	-0.207739473
AKR7L	0.367072602	1.355264764	0.47831709
RPL35P5	-2.462449511	1.354423386	-0.043926087
DACH1	1.635346455	1.351297903	1.840448848
SNORD36C	0.370003111	1.348069553	0.188750044
FAM223A	0.141925652	1.346809792	2.188821421
LOC105372990	0.399704608	1.343971279	0.657486297
RBM20	1.332273493	1.343002724	2.114896221
HSPE1-MOB4	2.416479176	1.341935649	0.087988233
LOC101928595	-1.069389291	1.339199448	1.323933097
SNORA33	0.918882622	1.338198835	0.604387522
HKDC1	1.306963354	1.337795297	1.716687089
LOC105371795	0.566059235	1.337627485	1.121050569
TRNI	0.982340079	1.335949026	1.344055427
KLHDC8B	1.183092031	1.335937923	0.676544995
ATP1B1P1	0.602235171	1.335821895	-4.11899021
ALOX12P2	0.401390592	1.33578419	0.918683853
NDN	0.50140913	1.335195548	0.542465235
ZNF280A	-0.543005396	1.334643301	1.175842717
RNA5S8	1.416875932	1.332006475	1.265406276
ALG1L	2.428734414	1.328611799	0.280103457

PCDHA1	-1.652169333	1.32718216	-0.005211247
LOC100289361	0.464163348	1.326093998	-1.186848341
LOC100500934	-1.011365902	1.324308577	-0.662329528
RPS24P17	1.59677989	1.32098922	2.248110147
PTMAP2	1.325534425	1.320809009	0.85112102
SNORD19C	0.424654752	1.319616972	-0.540127327
RPL17P18	2.911669854	1.31933237	0.011805181
RPL12P35	-0.096067613	1.318445401	-0.658986984
SV2A	1.963403433	1.318252975	1.088125235
HOPX	1.138406605	1.316950057	1.36663488
ARMC4	-0.564273833	1.314630056	0.327575147
MARCKS	1.293443478	1.314014227	1.303055666
ATF7-NPFF	-0.355428618	1.313241032	-0.108290164
SNORD50A	1.727876297	1.311558511	0.323447509
NOP56P1	0.584141297	1.309222757	0.203645997
ALX1	1.586486773	1.309144123	0.431416484
SMIM1	-0.368892716	1.308311138	-0.679693
RPL5P4	-0.717440656	1.306357283	-0.559003135
C7orf61	0.820250762	1.306178873	0.316930367

Supplementary Table S12. 500 ea down-regulated genes based on TLR4 in A549 cells

Gene_Symbol	Fold change (TLRs vs. vehicle)		
	TLR2	TLR4	TLR7
MIR6510	-17.95158069	-16.95158069	-16.95158069
MIR5187	-6.722285856	-15.55200859	-6.662504628
MIR34A	-3.848014197	-15.44156094	-9.212742251
MIR3940	-1.575827107	-11.4779772	-12.2221383
MIR4721	-5.654783238	-9.140499502	2.325496738
MIR4761	-7.94677805	-8.093167537	-7.897025015
TBCAP3	-3.583062198	-7.539890364	-2.868532894
DUX4L12	-0.469368593	-6.419370343	-3.485797704
MIR29B2	0.132965192	-5.230888767	-3.832594531
SNORD140	-1.047221463	-4.933850862	0.362074123
MIR1287	-6.58128135	-4.830879616	-1.041820176
TPTEP2	-0.967992623	-3.985073762	-5.032137518
HCAR2	-0.095871276	-3.958463605	-3.057456849
HCAR3	-2.714172348	-3.865477195	-2.84213916
ID4	-3.192909101	-3.842342997	-3.975313409
FABP5P2	-0.73300623	-3.826461555	-2.266787225
TGFB2-OT1	-1.557504342	-3.818700595	-2.330395539
RPS2P27	-3.976833752	-3.802751125	0.047406475
SEPTIN14P7	-3.323450193	-3.707352793	-1.123163293
USP17L18	1.904578954	-3.701826258	-1.192447109
BNIP3P5	0.587916106	-3.674556393	1.718616847
MIR4720	-0.033015212	-3.648807612	-2.249868171
THAP5P1	-1.921166363	-3.602843274	-0.163788353
PAPPA-AS1	-2.243594973	-3.574882216	-0.288146683
KRT18P4	0.603130179	-3.558913849	-1.534017881
MIR10394	3.422514147	-3.336429968	1.616040678
PDXDC2P	-0.477686849	-3.314305649	0.504675545
RPS3AP49	0.475036499	-3.286649259	-0.50440517
SERF2-C15ORF63	-0.225250166	-3.242567902	-4.759209965
LINC00312	0.347824023	-3.238231015	2.135227381
LOC389473	-2.711058294	-3.218053384	-0.685485177
PPP1R14BP1	-1.855293458	-3.216284378	0.612698727
OR2B6	-2.745311771	-3.183753272	-1.987170172
RPL23AP43	-2.386249473	-3.145547898	0.412083936
SLC7A5P1	-0.110448157	-3.091903745	-0.028294535
RPS7P3	-1.512792303	-3.039689259	-2.154702293
KPNA2P3	-3.74305512	-3.039342323	-4.374640577
KRT18P10	1.212102495	-3.027275835	1.21180679
MIR935	-1.345995855	-3	-2.661198087
SNORD108	-3.153401872	-2.990054813	-2.659945385
MIR10400	-2.662965013	-2.925999419	-0.788495895

RNU7-1	-1.157620726	-2.916587307	-2.558356164
LOC102724908	-2.015494396	-2.901594627	-1.880571083
BANF1P2	-1.597631858	-2.899229264	0.411421243
CTNNA1P1	-0.963482129	-2.886778313	-0.43356793
SNORD51	-0.516525962	-2.789272002	-1.656782712
RPL39P19	-1.22737801	-2.752894312	-1.047883001
LOC646050	0.186796687	-2.730284532	0.751768587
DUX4L11	-1.159126003	-2.719973605	-3.253680447
DUX4L10	-1.159126003	-2.719973605	-3.253680447
PRKCZ-AS1	-1.274929147	-2.701863417	-0.65344004
DUX4L13	-2.603632028	-2.696299362	-2.445496275
DUX4L14	-1.634878735	-2.695614494	-2.137284674
MIR2110	-0.666360923	-2.644987272	1.465585595
CPA4	-2.628476226	-2.62352	-3.301884199
LOC100422094	-1.36347363	-2.574169918	-4.319642449
CACYBPP2	-0.087696875	-2.545172652	0.490716127
SNORA14B	-0.298358544	-2.543955718	-0.89690741
LOC105378577	-6.411702121	-2.534679959	-2.196451063
LOC100130172	-0.540487347	-2.504850326	0.214609782
NASPP1	-0.606334352	-2.49444695	1.017551324
LINC00592	-0.264417779	-2.492063841	-1.751364114
IRF8	-1.886803758	-2.478136126	-1.340303534
LYPD6	-1.069534823	-2.470880047	-1.120796731
MIR339	-0.536117925	-2.445965311	-2.024937033
SNORD118	-2.834474716	-2.4394501	-2.745240852
ACTN4P1	-0.144456775	-2.437897098	-1.15935576
RN7SL49P	-0.553454969	-2.437416285	-1.030859528
HAVCR2	-0.798963614	-2.432919506	-1.591707222
SCARNA2	3.060728038	-2.429396154	0.608828531
GUCA2B	0.151796915	-2.426922409	-1.041351218
MIR1178	-2.361837986	-2.414893139	-12.96115916
DYNLL1P1	-0.568704317	-2.414705911	-0.738317369
DANT2	-2.404630462	-2.397768565	-1.709046475
TRS-AGA2-6	1.145050333	-2.378511623	-3.530514717
RPS29P16	-0.218285109	-2.37505414	-0.248376327
LOC102723493	-1.614622055	-2.374823557	-0.924950272
MIR4512	-0.791443286	-2.367495919	-1.786544271
ATOH8	-2.506874845	-2.360253626	-2.884331601
RPS26P37	2.268932872	-2.353424578	2.470348465
MIR7705	-1.041820176	-2.321928095	-0.374395515
RPL12P25	-0.683304449	-2.309609951	-0.882462889
NPM1P29	0.975705505	-2.300367936	1.853873045
ATP5MF-PTCD1	-0.587719753	-2.288913769	0.776438735
KRT8P30	-0.085880852	-2.282291535	-1.13853302

RPL26P6	-1.411129485	-2.280813322	-1.360196035
MIR6781	-0.681829839	-2.276408617	0.746942121
CT75	1.844387188	-2.274121357	1.002728467
MIR6721	-2.571549523	-2.273128986	-0.882305207
MT1XP1	0.466181419	-2.244722109	-0.153343881
FRMD3	-0.891512257	-2.233504689	-1.85934198
MRPL36P1	-1.10463558	-2.217942933	-0.90760842
MIR4315-1	-2.166682989	-2.196126767	0.100292795
RPL23AP5	0.752953661	-2.18961469	-0.843928541
RPSAP11	-1.619117536	-2.186402662	-2.013207864
ID2	-2.160991695	-2.162481362	-3.619587608
KRT18P15	-0.506241994	-2.15649202	-0.529420037
CCNI2	-0.83838155	-2.150583279	-0.434243357
SENP3-EIF4A1	-0.145513629	-2.105808961	0.421595106
SYNDIG1	-1.759497671	-2.102319202	-1.152163174
RIPPLY1	-0.684071836	-2.101635348	-1.556853275
RN7SKP175	-2.678751353	-2.097163327	-1.002036618
MTHFD1P1	-0.670213991	-2.089659936	-0.23029607
RGMA	0.384103925	-2.081566385	-1.097874858
SNORD88A	-0.116679345	-2.077251175	-0.458133464
APLN	-2.010597207	-2.071858299	-0.891396338
SYPL2	-0.53937841	-2.071326516	0.250482256
FOXD4L6	-2.524737849	-2.065569793	-0.575005916
SBK2	-1.03241476	-2.058031901	-0.748637988
ID1	-1.925339144	-2.029942802	-2.768633875
VWA5A	-1.253215411	-2.025997707	-2.568702376
GRIN2B	-1.838336889	-2.019017257	-0.590128943
MIR5001	-2.074159213	-2.007329043	-2.020625291
ATRNL1	0.173263567	-1.998985909	-1.840015936
SNORD3D	-1.812494131	-1.998702436	-0.724951319
SNORD58C	-1.062542216	-1.987343073	-0.489629852
EEF1A1P22	1.926333678	-1.978507473	1.333831539
MIR4697	1.31021743	-1.972375752	1.098013576
UGT1A4	1.044163866	-1.963734598	-0.443210328
DUS4L-BCAP29	-2.293730555	-1.962989874	-0.437895211
SNORA56	-1.916708914	-1.961085064	-2.869755666
SNORD3B-1	-2.629148282	-1.953229817	-1.567944827
MAN1C1	-1.062852049	-1.94042089	-1.995098709
VDAC1P2	0.353337009	-1.91790094	0.286561093
LOC643339	0.345456439	-1.917380081	-0.039927562
CYP1B1	-1.953713182	-1.904470923	0.889388812
FAM43B	-1.41492748	-1.901547854	-0.40564766
DCAF13P3	-1.217389525	-1.886429267	-0.675831565
FBLN7	-0.089114604	-1.866814607	-0.026359185

SAPCD1	-1.780724953	-1.858236212	-2.002608507
ANKRD2	-2.477755398	-1.85749453	-2.302897494
ADORA1	-1.54896752	-1.856995921	-1.5390038
LOC101928841	-0.985618726	-1.856985278	-1.527700252
LOC648927	0.904798903	-1.852035862	-0.261963456
FAM186B	-1.688839074	-1.851270372	-1.252114304
SCARNA21	-0.771021112	-1.850595092	-0.601700412
TGFB2-AS1	-0.807313764	-1.841310412	-1.142561807
NDUFB4P12	-0.935228305	-1.830580019	0.518062815
RPS19P3	0.513829545	-1.824853903	-1.413171757
EDN2	-1.587047254	-1.80344048	-2.403227628
SNORD135	-0.579986326	-1.796068503	-0.716056458
CYP51A1-AS1	0.270038398	-1.775551501	-0.097150064
MIR12121	-3.357552005	-1.772589504	-2.772589504
LOC442064	0.152003093	-1.771375625	-0.901436166
TAS2R10	-1.1009939	-1.769989064	-1.737846759
NUDT10	-1.130033633	-1.768429694	-0.707740024
HSPA6	-1.90057907	-1.767927285	0.011110326
SV2A	-2.717625731	-1.767096252	-2.004626679
DERL3	0.41416252	-1.759889322	-4.526675408
RPS25P6	-0.106592012	-1.755261155	-0.539964768
LY6K	-0.7358713	-1.752634255	-0.79917632
PCDHB18P	-0.876810109	-1.745679918	0.088770792
HOXB-AS3	-1.589265773	-1.744341582	-2.324192285
LOC100419814	0.801489183	-1.737484113	1.205425299
LINC02012	-0.962479263	-1.736159249	0.906281636
SYT12	-1.624048957	-1.73246394	-0.757557456
RNF139-AS1	-1.930227179	-1.731206523	-1.664636548
KCNJ2-AS1	-1.119721427	-1.728707783	-0.631835904
MIR4737	-1.98799064	-1.722021796	0.225200544
SUSD4	-0.708425287	-1.721799482	-0.46591187
MIR4680	-0.485426827	-1.719892081	-0.347923303
SNORD14E	-0.800796113	-1.705897322	-1.428795739
ZNF284	-1.109194518	-1.70514875	-0.474611967
NCALD	-1.880653225	-1.700636688	-1.3766195
JPH2	-1.105075086	-1.700154518	-1.463584584
BEST3	-0.800351731	-1.69961712	-3.287046886
LOC105376772	-0.465672898	-1.697491844	0.423029011
GCSHP3	-0.921989156	-1.684746971	-2.453559664
MIR503	-0.298094278	-1.679940216	-0.673181947
DIO2	-1.625184003	-1.678510681	-1.19824793
SP140	-1.312221583	-1.675734237	-0.326550227
NEK10	-1.56017973	-1.671864668	-1.47991741
SLC16A10	-1.790916421	-1.664076021	-0.248138918

LRRC70	-1.111839474	-1.664034655	-1.168952661
TGIF2P1	-0.90768869	-1.66330039	0.440576243
KRT6A	-0.982835636	-1.659525659	-1.008409465
MIR106B	-1.758925572	-1.652799232	-0.159015428
CEMIP	-0.666563191	-1.638079742	-0.689577932
UBE2FP1	-0.152928959	-1.635282162	0.840243986
HMGB2P1	-0.502299583	-1.63414295	-0.663291304
UNC79	1.254714497	-1.633332365	-0.955468027
DSCR9	-0.598014603	-1.632581582	-0.058519439
ADAM22	-1.628437213	-1.632248538	-1.570584079
MT2P1	-0.86857942	-1.612467329	-0.897493952
RNU2-59P	-0.665733555	-1.611812716	-0.690865406
CD101	-1.062189537	-1.609566925	-0.827773536
RPS17P16	-0.558485487	-1.603172102	-0.855778889
TLR5	-1.854402458	-1.594861592	-1.780842742
MIR1306	-0.408933613	-1.58780525	-0.894302432
CCBE1	-2.071038997	-1.587093214	-1.3023867
PLEKHA4	-0.202611985	-1.584020972	-0.432014148
SNORD100	-1.4279726	-1.578611035	-1.277330606
SNORA58B	-0.17973227	-1.576024763	-0.040145193
H2BC7	-1.532659125	-1.574578928	-1.023829588
SLC4A8	-1.876766428	-1.571424377	-3.430313441
EIF4HP2	0.507180357	-1.564464175	0.423560001
NAIPP3	-0.680459228	-1.555164034	-1.138275739
SNORA41	-1.049678571	-1.554541135	-1.132962155
LOC100287430	1.993692562	-1.546927436	-2.186349015
DIXDC1	-1.836736894	-1.545569641	-1.062413116
MIR4657	-2.401744466	-1.542634984	-1.068944465
SLC6A13	0.333341762	-1.541576188	-0.071970509
LOC100128361	-0.00144239	-1.540697086	-1.35631786
HSPA8P8	-1.690763276	-1.540418855	-0.826438279
PDE6G	0.049766623	-1.533018486	-1.182356564
ID3	-1.508722162	-1.53105167	-1.779484388
LOC146253	-0.773830339	-1.527902237	-1.419948687
FXYD3	0.307253773	-1.525787486	1.373941911
GPR20	-2.556778327	-1.523373353	-1.956797994
SNORD42B	-0.796750822	-1.522315592	-0.950641971
LOC100128775	-1.449847915	-1.519176229	0.158799976
CRYAB	-0.741839455	-1.51001665	-1.425133731
SOWAHA	-1.292766294	-1.51000553	-0.466558808
FBLL1	-0.424633713	-1.505414176	0.617343934
HPDL	-0.949448873	-1.488877525	-0.127940193
NPHP3-ACAD11	-1.093500447	-1.487665176	-0.632952515
PRDM12	-0.089999271	-1.486754509	-0.445982437

GAS5-AS1	-0.862455466	-1.485775846	-0.192235362
MARCHF4	-0.750158504	-1.48547261	-1.458103332
MIR8072	-2.846087317	-1.483517237	0.419806743
LOC101927551	-1.510213813	-1.478081192	0.023695189
GUCY1A2	-1.08557188	-1.477659417	-1.039655158
RPL4P3	-0.265325859	-1.4743493	-0.712298271
STAG3	0.362154051	-1.474118225	0.053105742
MIR10399	0.294719251	-1.471355932	-1.098273749
PRELID1P4	-1.61917596	-1.467898021	-0.776121985
FEN1P1	-0.722244252	-1.466395404	0.851298545
IMPDH1P10	-1.641479702	-1.465968288	-0.102523188
MIR374B	-0.704254211	-1.459505102	-0.702448527
TIPARP	-1.089124393	-1.453672144	1.589118564
CAVIN4	-1.241796817	-1.453087459	0.936631605
PCDHB17P	-1.170095765	-1.45117376	-1.015335181
MCPH1-AS1	-0.006064584	-1.447664213	-1.442955504
WNT2B	0.465318697	-1.445833598	-0.233189105
LOC644285	-1.378254652	-1.441972299	-0.432782267
MIR7106	0.250026199	-1.441051572	-2.470511903
LINC00565	-1.697005952	-1.439705322	-1.667690385
SNORD97	0.069255537	-1.430711661	-0.30911394
IPO7P2	1.336189767	-1.425860469	1.466967176
EEF1B2P2	0.403622349	-1.425620552	1.314918537
ALX4	-1.432713585	-1.424787148	-1.165289722
SNORD3C	-3.104032699	-1.418098291	-0.229604907
ACOT4	-0.977366032	-1.417535096	-1.308752234
PPIAP4	-2.059049308	-1.415261596	-0.683948752
FTH1P11	-0.914829638	-1.413898239	-2.160047402
FTO-IT1	-0.914915978	-1.411645656	-0.710871518
TOMM20P4	-1.000747187	-1.409980317	0.418048507
USP10P2	0.467870627	-1.409041849	0.888712451
ESRRAP2	-0.34669286	-1.406271553	0.084397683
ESRRAP1	-0.34670424	-1.406268348	0.217082126
SOWAHCP5	-0.737922469	-1.405190379	-2.246672578
PLAAT1	-1.217610531	-1.402773087	-0.74416828
MIR4292	-0.096802901	-1.394396207	0.277735346
KRT222	-0.966006918	-1.393759986	-0.967959734
SHISAL1	-0.979574145	-1.393644803	0.891144685
NAIPP1	-0.512530285	-1.387251454	-0.970412751
MIR922	-0.521442763	-1.385314035	1.537786788
C11orf86	-1.280703944	-1.384774219	-0.922965047
VWA5B2	-1.250368211	-1.379104609	-0.61268803
APLNR	-1.43596435	-1.376241009	-1.962934179
LINC00602	-1.057261146	-1.375568319	-0.444869272

HSPH1	-1.356374494	-1.372917877	-0.523894605
LINC01719	-0.581727458	-1.37270492	-1.031850424
HOXA11	0.512954918	-1.371069349	0.699517269
MIRLET7I	-0.508806409	-1.368978718	0.347642315
ANKRD20A13P	-1.241396978	-1.364889453	-1.490035214
SEPTIN14P4	-0.094822918	-1.362091511	0.141014336
LOC644456	-0.058112942	-1.356809842	-0.3695085
TNKS2-AS1	-0.911917617	-1.354419869	-1.472561379
MIR5587	-1.610742431	-1.354022607	-0.030210171
CHCHD2P6	0.001592066	-1.352150972	-2.103924884
SAMD11	-1.539439017	-1.34934996	-1.695806932
DPRXP4	-0.222344529	-1.347018673	-1.183192769
SNORD167	-0.725562162	-1.34542204	-0.529588286
SNORD42A	-0.244584684	-1.344651814	0.064100596
BZW1P1	-1.166241814	-1.344127832	0.016102925
HNRNPA1P77	-0.4628436	-1.342598491	-1.071258845
MIR221	-0.501955023	-1.339929141	-0.167400325
TAS2R3	-0.74909925	-1.337398631	-2.598929
NOTUM	-1.523251748	-1.337208418	-2.216487826
RASSF1-AS1	-0.596585658	-1.335604411	-1.46995633
PIWIL2	-1.174713129	-1.329802013	-1.815979102
SLCO1B3-SLCO1B7	-1.138050464	-1.329277698	-1.625284612
RPL10P3	-1.00933603	-1.326481004	-0.685557697
SUMO2P17	-1.15812371	-1.323974065	0.119543674
RPS26P3	-3.108294857	-1.322103919	-0.658682637
LOC646012	0.190461496	-1.319284392	-0.461660214
ACTBP7	-0.672772566	-1.316748872	-0.843969898
TIMM8A	-0.483316702	-1.313463658	-0.171592216
TNNC1	-1.61160163	-1.313236556	-2.264297816
SNORD3A	-0.616089405	-1.312041814	-0.52782408
MIR589	0.904579188	-1.311212628	-0.162642304
NCKAP5	-0.986526999	-1.310479468	-0.916164018
DPF1	-1.338340083	-1.309818249	-0.290938373
LINC00473	-1.53023538	-1.302064014	-0.974311935
SNORD12C	0.657397958	-1.30032226	-0.100055048
CRYBB3	0.680397408	-1.298260372	-1.780547439
NKILA	-1.127788945	-1.297747921	-0.43596255
MIR1248	-1.106745011	-1.294615087	-0.166881017
GFRA2	-1.429300435	-1.2935316	-0.339031021
GALNTL6	-1.614442728	-1.288717258	-0.799942412
GRHL1	-1.038750522	-1.288715011	0.337028884
SNORD13H	-0.391441606	-1.285156172	-0.220779435
NOS3	0.170761052	-1.283909844	-0.790742805
CLVS1	0.012118029	-1.283765224	-0.054477585

DNAJC28	-0.173729047	-1.28353679	-0.395989597
DUX4L3	0.963739684	-1.28251971	-1.587628399
MIR7110	-0.408405094	-1.280235574	-2.197848124
ABCC6P2	-0.725510994	-1.280121619	-0.942544474
POU5F1P3	0.236861279	-1.279022464	-0.693371852
ZNF583	-0.907125201	-1.275207034	-0.990693334
NDUFB3P4	0.025442073	-1.273177531	0.884608905
ONECUT1	-1.557250778	-1.270895714	-0.348375641
PAIP1P1	-1.188750453	-1.268408175	-1.279734371
SNORD1C	0.549575226	-1.267846198	0.556793113
HSPA1A	-1.492480188	-1.266225782	-0.989899086
USP2	-1.845251085	-1.263732219	-0.007754193
TENT5C	-0.187999205	-1.26049411	-1.369963439
ACTG1P4	0.701057883	-1.260438123	-0.803952211
LSAMP	0.012391383	-1.257529409	-0.482463045
HSPA8	-1.321439781	-1.254142975	-0.548210238
HES1	-0.869491765	-1.251438039	-0.646491865
BNC2	-1.301704747	-1.248094099	-1.348977135
CREB3L1	-0.292819685	-1.247300549	-1.065871222
PRRG2	-0.682243476	-1.244036535	-1.048258097
HCG4B	-0.279948916	-1.242790131	0.519854734
LOC645967	-1.331918109	-1.242216195	-0.621707196
HSPA1B	-1.462406134	-1.240949783	-0.996185367
EFR3B	-1.16819221	-1.237334444	-0.557995398
HENMT1	-0.937754364	-1.236714273	-0.72313511
TENT5B	-1.407526457	-1.235316367	-1.146890436
PCDHB9	0.453550199	-1.234582211	-0.155996627
RPL13AP14	-1.178708509	-1.234421397	-2.117347347
MIR4745	0.604715206	-1.234284937	-1.55218966
CDC42P6	-1.307580505	-1.232485342	-0.94509014
SCARNA15	-0.908916478	-1.232054089	-0.13913671
NR4A3	-1.977308035	-1.231456602	-0.172791803
MYOCD	-1.9944511	-1.23090072	-0.794942569
MYO1G	-0.988520796	-1.230366494	-1.180175121
NPIPB1P	0.024032671	-1.229135712	-0.041997383
SNORD142	0.354494591	-1.22865549	-1.292363843
MIR570	0.159795063	-1.227939349	0.157622937
NPM1P7	-2.078708094	-1.226984226	-1.354875579
ALDH8A1	-0.288729223	-1.226082482	-0.441948826
SNORD18A	0.709269487	-1.223382915	0.051544171
GALNT9	-0.786341101	-1.22267296	-0.886493051
EVA1A	-1.093775851	-1.220068312	-1.173635974
AOX1	-0.626424794	-1.218415615	-1.432690902
CHST6	0.725428925	-1.218092284	-0.565663733

MIR943	-1.144652128	-1.214942697	-1.858404453
LNCSRLR	-1.085488613	-1.213023344	-1.264651744
SNAI2	-1.093997164	-1.212783655	-0.794146412
UGT1A5	-1.903171768	-1.211604478	0.572900168
LINC01559	-1.147621808	-1.208047866	-0.311018065
ZDHHC8P1	-1.748007864	-1.207069764	-1.634585475
MIR6752	-1.019692156	-1.204772858	-0.992547698
SNORD15A	-1.917244607	-1.201416175	-1.080642123
SCARNA10	0.536902765	-1.200168779	-1.218227108
SNORA103	-0.540902718	-1.198695966	-0.671991232
DNAJB5	-1.182669296	-1.195481732	-0.561581501
TTC41P	-1.506489329	-1.193443042	0.318109101
MIR6125	-0.162576522	-1.18862388	0.505060581
LYPD1	-0.95224407	-1.188439827	-0.9145818
SNORD6	0.017685459	-1.187849498	-0.446607004
GRAMD1C	-0.777165367	-1.185648787	-0.728943588
NES	-1.07386354	-1.184391669	-0.239623335
TSPAN18	-0.99470688	-1.182196932	-0.610649369
PTGS1	-0.872824339	-1.181525725	-0.256198033
DHRS9	-0.920829282	-1.178481966	-0.489849501
FAM25A	0.886705475	-1.178407478	-0.124828969
KIF26B	-0.568208231	-1.175446879	-1.399436086
FOXD4L1	-1.144233106	-1.175304303	-1.592337701
LOC730668	-1.255806552	-1.174777082	-2.929090457
TMEM255A	-0.160442398	-1.17473252	-1.973808713
LOC100130238	-1.338947042	-1.174305862	-1.273386739
HOXA10-AS	-0.916711122	-1.173733933	-0.263269494
RPS20P14	-0.091691569	-1.171324516	-0.642232453
IQCN	-1.493058549	-1.170932465	0.695020484
OR7E37P	-0.352618447	-1.170270666	0.772786011
LOC101927402	-1.683540733	-1.169357622	-0.322964469
RPGRIP1	-0.472524137	-1.169213695	-1.189215975
SNORD3B-2	-2.26798476	-1.168935019	-0.674228733
SNORA5C	-1.278206815	-1.159298847	-1.154181666
LOC441086	0.08188403	-1.157691133	-0.742801803
RPSAP17	-1.625917082	-1.151736565	0.450878349
LOC105274304	-1.245574485	-1.149773543	-6.441992582
LOC100506691	-0.781960014	-1.149371166	-0.48008008
RPS26P50	-0.831456095	-1.148783548	1.832510919
MIRLET7B	-0.139619369	-1.146486088	0.425261731
TRPM2	-0.476753997	-1.143481762	-0.526602269
ISM1	-0.872084393	-1.143214862	0.199557385
MIR4426	0.682807463	-1.142942953	0.47792913
RPS20P21	-0.720109698	-1.141763909	0.338113523

LOXL1-AS1	0.545597983	-1.139830439	0.51618401
LINC01842	-0.503066215	-1.139745631	0.20307007
CHORDC1	-1.080740928	-1.13915946	-0.510576047
PRH1	0.139063623	-1.138370106	-1.461839012
ZDHHC14	-0.810880204	-1.136554209	-0.318763983
SHISA8	-0.808578816	-1.135779596	0.17241378
CCDC138	-1.117069194	-1.135393969	-0.124612454
RPS3P3	0.204499041	-1.13501917	-0.343427313
TMEM81	-0.845667415	-1.132824388	-0.509532343
RPL31P4	0.680189297	-1.132660877	0.384963857
ZNF774	-0.83850069	-1.132567492	-0.333798664
UNC5D	0.016971786	-1.132264373	0.533225754
NPM1P9	-1.191847976	-1.129782369	-1.247316577
PDE3B	-0.943663793	-1.128666324	-0.350016383
IL11	-0.986106397	-1.124979606	-0.124324735
MAGOH2P	0.581050734	-1.124400024	-4.925250538
RPL15P22	0.172102667	-1.121914069	-0.387450161
TMPOP2	-0.521770054	-1.118921627	0.524371504
FSCN1P1	-0.668364665	-1.117530884	-0.595947114
PCOLCE-AS1	0.71989641	-1.117323397	-1.210035516
VN1R103P	-0.290066238	-1.116476939	0.762008703
TRIM34	-0.420597431	-1.115146537	-0.115996083
MIR4658	-2.894563976	-1.11211195	0.115258037
MB21D2	-1.208411041	-1.111630052	-0.107103912
SNORA50C	-0.718441774	-1.108755454	-0.216006184
NEXN	-0.799674543	-1.108060999	-1.091761463
STC1	-0.573551111	-1.107870413	-0.233861332
PDE3A	-1.054555321	-1.106276544	-0.743175141
LOC102724064	-1.813109214	-1.102297421	-0.989302431
SNORD119	0.118513919	-1.101466486	-0.278122388
L3MBTL3	-0.890028595	-1.101357765	0.629402382
LRRC37A9P	-1.420037331	-1.099900971	-0.68987378
PTH1R	-1.895732408	-1.099517227	-1.428068767
LINC00537	-1.619829628	-1.098601807	-2.936016633
SOX8	-0.577314463	-1.098160347	0.243824338
LINC002481	-0.619824466	-1.098134799	0.327028082
LOC100419620	-0.943011897	-1.098028788	-1.284865478
SNX18P12	-0.531755907	-1.096665042	-1.261690628
DRAIC	-0.572039181	-1.095199644	-0.145204662
MIR6776	-0.151598917	-1.095050761	0.829255797
PDLIM1P4	-2.405881414	-1.091433397	-2.603064032
TCF21	-1.655469192	-1.091332726	-0.900703411
PCDHB8	-1.600402215	-1.089950894	-1.497526585
FRMPD2	-0.165843429	-1.087976352	0.288623775

CGB5	-0.654443423	-1.087866536	-1.716533619
MASCRNA	-0.628031223	-1.087462841	-3.087462841
STIM2-AS1	-1.109930277	-1.087426907	-1.040521504
RPS4XP17	-0.2568427	-1.084926793	-0.709508453
RPL31P59	0.003600103	-1.081032896	0.922283103
PLEKHG4B	-0.924627675	-1.080057008	-0.438784174
SNORD69	-0.194782032	-1.07900215	-1.187744489
ST13P6	-3.158702363	-1.078951341	0.294915348
SNORD14B	-0.294920239	-1.07810908	-0.250262867
FTH1P1	-2.304108508	-1.074806507	-0.827308595
MCM10	-1.063989159	-1.071996274	0.070134231
NONOP2	-0.50303917	-1.070976085	-0.68145255
NR2F2-AS1	-0.421719616	-1.069474597	-1.772982261
RMRP	-0.130583696	-1.067428867	-1.378149337
RAD51L3-RFFL	-1.393562687	-1.066877984	-0.591336325
OR11H13P	-1.282573081	-1.065306367	-0.72145241
KIRREL3	-0.862168579	-1.063073344	-1.029757633
LINC02735	-0.804353326	-1.060677026	-0.42199416
CFAP45	-0.562728044	-1.059176065	-0.534604528
ALPP	-0.644098919	-1.05816366	-0.845960031
RPL17P38	0.437306228	-1.057392629	-0.621111267
MIR5010	-0.30991822	-1.057182655	-0.882625426
TRIM69	0.159477245	-1.056696895	0.568039976
SLC51B	-0.404738953	-1.056516433	-1.362547846
CRYZL2P-SEC16B	-0.440731542	-1.055190219	-0.533995943
PLXNA4	-0.538876605	-1.052339407	-0.934445109
KBTBD8	-0.805011144	-1.051262039	0.027686071
POLR3G	-1.148656542	-1.048514361	-0.557094696
CMPK2	-0.242078863	-1.046784543	-0.384341212
CIRBP-AS1	-1.265863432	-1.043068529	-0.36160924
SNORD15B	-1.459359471	-1.041926348	-0.376548707
NR1H4	-1.972442817	-1.041375346	-1.960003439
SCAT1	-1.286299067	-1.03641387	-0.505643345
GHR	-1.583768661	-1.035364166	-1.244038735
IFFO1	0.375953362	-1.034662638	-0.558579451
RPS2P8	0.687625928	-1.034480458	-1.684862923
OTUB2	-0.482320829	-1.033632512	-0.341728294
CPNE4	-1.143912755	-1.031298227	-0.907755352
RTP4	0.647318586	-1.031080396	-0.587060154
LINC01119	-1.311509983	-1.031062295	-0.995251157
LOC100287653	-0.322845435	-1.031057539	0.305819737
TNFAIP8L3	-0.144802329	-1.031027455	-0.003535806
SNRPEP4	-1.794839128	-1.029720988	-0.362208216
RBMS3	-1.458314313	-1.029053864	-1.324161245

FAM201B	-0.018959934	-1.026775027	-0.167912061
ZNF141	-0.758519932	-1.024407052	-0.221472668
PITRM1-AS1	-0.703240608	-1.022895296	-0.136669352
KSR2	-1.007357478	-1.022396901	-0.735310069
TNFRSF8	-0.174194533	-1.022094852	-2.394298395
CHRM4	-0.637982274	-1.02172785	0.493990218
LOC101928673	-0.735552957	-1.019755669	-1.688410191
COL26A1	-0.316271947	-1.018155195	0.678417797
LINC00862	-1.410210109	-1.018132722	-0.725697116
SALL1	-1.064829987	-1.015272267	-0.334444865
HMGN2P15	-0.441877175	-1.014771267	1.024365847
MIR181A2HG	-1.227557017	-1.013922945	-0.41637436
EPHX3	-0.033657429	-1.01312675	-0.723837895
MIR675	-0.691576579	-1.013109541	-0.983133769
ACTBP2	0.47888817	-1.011869469	-0.270050065
ETS2	-0.940601944	-1.010239703	-0.697032175
DGAT2	-0.863592422	-1.009953073	-3.32962117
HSP90AA1	-1.020622299	-1.005848187	-0.539104493
CGB3	-1.057022496	-1.005766876	-0.962801147

Supplementary Table S13. 500 ea down-regulated genes based on TLR4 in H1299 cells

Gene_Symbol	Fold change (TLRs vs. vehicle)		
	TLR2	TLR4	TLR7
MIR5187	-0.472130223	-15.31189019	-1.124768075
MIR6848	-0.832398135	-14.60613451	-0.086944788
MIR3605	-0.040587617	-12.55293392	2.010737166
SNORD121B	-10.54719897	-8.633308438	1.610605067
MIR4315-2	1.299560282	-8.029378108	-5.863645349
COMMD3-BMI1	-1.171119225	-7.425962046	4.329394249
MIR639	-2.245113866	-6.580173909	-2.528447818
ASS1P2	0.244659319	-6.528876867	1.083269291
SLFN11	-5.117794025	-6.266169588	-5.537786855
SLC38A5	-6.888686591	-6.169136549	-6.749001635
SNORA92	0.247341523	-5.994077768	-0.062275566
SPTB	-2.489993354	-5.546529791	-2.547424476
CD36	-4.503004007	-5.536855085	-5.249959155
SFMBT2	-6.10742406	-5.206117929	-4.360559269
LOC729291	-1.224006693	-5.085614008	-0.386959539
MYOM3	-5.508626969	-4.980258905	-4.303871502
P2RX7	-4.514207747	-4.935520532	-4.843815882
HNRNPA3P10	-2.580521114	-4.902287261	-2.211364334
NHS	-4.578923772	-4.716371171	-3.476585418
IQGAP2	-2.865574182	-4.692615827	-4.3414314
UNC13A	-4.411768718	-4.691138905	-3.719856021
NPM1P44	-1.798326522	-4.67917139	-0.025023888
PPIP5K1P1	-4.222271843	-4.624176237	-0.123255795
H2BC11	-3.071002457	-4.620754671	2.040994178
LRRK2	-2.905599937	-4.508183855	-1.645313286
H2BE1	-1.174015173	-4.501612448	-0.47612332
LTBP1	-4.633080086	-4.305036362	-3.345263718
TMEM51-AS1	-1.898557046	-4.303274273	0.268584371
MIR943	0.208892669	-4.265226702	-1.015461306
LOC100420326	-2.601704472	-4.263646635	-3.641736762
EYS	-4.872744942	-4.228493041	-4.885635813
LRRK1	-1.4291604	-4.225638879	-2.413546393
CLDN4	-0.994481064	-4.225616404	-1.447358786
WNT2B	-1.407007881	-4.214668151	0.880753604
ZNF831	-4.16215439	-4.094914278	-2.855046236
INHBA	-3.953443474	-4.094355215	-2.660685805
PGBD3	3.553477057	-4.066338653	-5.073776566
ACTG1P14	-0.621608833	-4.053227237	-0.294165403
FSTL4	-4.792701469	-4.035426136	-3.613100293
SNORA119	-2.077819241	-4.019180536	-1.476504138
IL1B	-1.458620326	-3.941297826	-1.312856401

DSP	-2.641423837	-3.901651936	-2.84033399
TARID	-1.356803136	-3.901563772	-0.643905801
ZNF717	-3.431757496	-3.901418206	-3.605234719
PCDHA4	-2.772077326	-3.821458238	-0.651077417
MIR6800	-1.442717595	-3.772499478	-9.864014139
SYNC	-1.738195946	-3.765699155	0.047365793
DAZ2	-3.945576313	-3.72082635	-4.817807456
MIR24-2	-2.567040593	-3.700439718	-0.919217345
XK	-3.929878085	-3.664453441	-2.738170024
C19orf33	-4.358149938	-3.659653786	-4.430025096
IMPDH1P10	-2.094602969	-3.642268317	0.349476585
CRACR2A	-1.837919023	-3.639071897	-2.190390246
SNORA74A	0.00345525	-3.623383479	2.637503621
CDH11	-3.654564248	-3.615410089	-3.165763427
HHIPL1	-2.978010954	-3.611490916	-2.251566598
RPE65	-2.360660644	-3.587974523	-2.942058751
SNORD116-23	-1.615830345	-3.566265837	-0.257553653
SH3RF3	-1.650463423	-3.56596177	-0.961916908
ONECUT1	-1.23779648	-3.542612288	-1.276279276
RPS10P5	0.110198405	-3.510425525	0.027016687
BMS1P4-AGAP5	-1.552081937	-3.487556621	-0.512143946
AOC4P	-1.902194236	-3.477445673	0.071764598
PTCHD1	-4.61967473	-3.469542729	-2.284080123
GPR4	-3.105199929	-3.423704324	-2.456487815
HMGN2P46	0.939019939	-3.41753252	0.664628808
TNFRSF10D	-2.993513345	-3.411904321	-2.637641095
SNORD109A	-2.541814325	-3.405519379	-1.542135953
ARHGAP31	-2.683654317	-3.403977649	-2.895080452
NPAS3	-2.903497583	-3.393978985	-2.815016329
SNORD116-1	-4.148485596	-3.389246239	-1.247353146
ANO3	-3.376938224	-3.383437289	-2.319960593
PIK3AP1	-1.695447374	-3.377198388	-2.025220951
MIR6501	-5.287752408	-3.365453944	0.619715692
DAZ4	-3.432681122	-3.347750099	-4.108153155
ST13P7	-0.608012503	-3.340942349	-1.117339122
TENM1	-4.675098404	-3.335885879	-2.163593688
KRT8P30	0.637179145	-3.325403191	-1.022055246
RAB4B-EGLN2	0.068729188	-3.279713453	-0.836122736
AHNAK2	-2.836271091	-3.269468638	-1.642749971
LINC01771	-0.463426099	-3.260104041	0.045558814
NNT	-2.787947614	-3.259193553	-2.690108368
SLITRK4	-3.63457096	-3.256286142	-3.528004613
MIR3936	0.704036802	-3.254457302	-2.576304582
DAZ3	-3.564311466	-3.240611867	-3.98359835

ADGRL3	-3.591492011	-3.227336176	-0.878328571
SEPTIN14P10	0.265366977	-3.212730407	-1.098634646
GTF2IP23	-1.271081632	-3.198444821	-0.226452052
ADORA2A	-3.054391288	-3.194127833	2.405599215
NRIP3	-2.988694855	-3.178284169	-3.260887025
DAZ1	-3.783326354	-3.176926659	-4.161686361
ABCA3	-2.36321311	-3.168254129	-2.209817862
CLEC2D	-0.209393856	-3.149759717	-0.948263223
H2AJ	-2.368649122	-3.146201976	-3.708994444
CCDC144B	-2.263472658	-3.138945697	0.06764695
ARHGAP28	-2.634546413	-3.13203865	-1.656943015
ARHGEF4	-3.177285005	-3.118322941	-2.839188252
IGFBP2	-3.153189954	-3.034319897	-3.336443259
LYPD6	-2.86980901	-3.034007744	-2.580041088
OSBP2	-2.438707937	-3.02385593	-2.014091114
BTBD11	-2.012858031	-3.021878128	0.033028616
LMNTD1	-1.130750322	-3.017241891	-0.753112603
LOC729674	-1.092180038	-3.011258738	-0.654175459
NNT-AS1	-2.58695544	-3.008615301	-2.230181385
EIF4A1P13	-4.314584604	-3.006505611	-0.117314413
MIR4523	-1.899198496	-2.996733359	0.864132205
SEPTIN3	-2.555956347	-2.989596761	-0.185918485
SCN9A	-3.191712736	-2.983718864	-1.415647117
ANP32A-IT1	-1.587905922	-2.974273792	0.077495856
SNORD62B	-2.486824443	-2.965181332	-1.052411343
CNTN1	-2.860871869	-2.96129013	-2.523813678
LAMB3	-2.726382102	-2.955139373	-2.6709333
NID2	-2.805963371	-2.95467528	-2.012612261
H2AC15	-1.397458065	-2.950592352	-0.227518716
RBMS2P1	-1.240734832	-2.949083032	-0.18025179
FAM106B	-1.538153231	-2.947511048	-0.221902441
LOC100190986	-2.556541124	-2.940599092	0.415286858
SCN3A	-2.89010623	-2.913239331	-2.583653278
RAB9B	-2.067012236	-2.910713807	-1.155016473
BHLHB9	0.285250422	-2.906797305	1.043420632
MIR5010	-2.749424023	-2.905509444	-0.385943471
ADAMTS9	-1.134106399	-2.888131031	-1.598138552
CCDC144CP	-0.270055621	-2.88506512	0.566045605
SPRY4-IT1	-2.928636293	-2.882279548	0.854748536
FBXL7	-3.779978483	-2.882102562	-4.367365545
GNAO1	-0.96336408	-2.863770123	-0.073541904
GLDC	-2.743345707	-2.861623344	-2.754817757
NACAD	-0.249279228	-2.85576024	-0.386296272
ATP1A3	-3.01650365	-2.848536147	-2.74969935

NXPH1	-1.034096555	-2.84467327	0.016049974
TNXA	-2.662921539	-2.842171285	-2.795573194
LOC112694756	0.357843389	-2.835591778	-0.13227796
KLK1	-2.182241756	-2.818357403	-2.017006385
ZNRF2P2	-1.014458382	-2.81007752	0.991662371
PREX1	-1.732302482	-2.796269105	-1.523508223
PCDHB4	-0.873727944	-2.793190318	2.60208776
HSPA8P1	-0.774375445	-2.782353742	1.532645562
TPBGL	-0.044180593	-2.78207186	-0.094639782
MIR4444-1	-0.200912694	-2.777741155	0.809057217
SNORD116-28	-4.215612952	-2.774220322	-1.30333682
ZNF365	-1.489835184	-2.77184845	-3.751301454
ADCY2	-1.68109269	-2.76951359	-0.832181705
SLC2A12	-2.532747499	-2.768575301	-0.846125902
MIR935	-1.235812937	-2.755599768	2.584962501
TENM2	-3.025098528	-2.751363526	-2.375069397
THRB	-1.072298948	-2.727117938	-1.941341538
FANCD2P2	-1.181952612	-2.712518033	-0.102673029
LHX1-DT	0.183810504	-2.708131414	0.621106628
MIR6720	1.959730135	-2.707803866	-0.06584974
GCSHP3	-1.391225487	-2.689737966	-1.01293057
ALPL	-2.211642291	-2.684343402	-2.149427021
PLIN2	-0.830829214	-2.684058347	-0.802634908
ZNF221	-1.471813372	-2.678660117	-0.121226119
IGSF3	-2.348347129	-2.678492527	-2.171211469
LOC440568	-3.757282372	-2.674936462	-2.284963745
G0S2	-2.187808939	-2.674121223	-2.693043035
DCAF12L2	-1.695212783	-2.663231898	-2.41845655
UTP14C	-1.068496796	-2.659507892	0.2355363
NRAP	-2.315205582	-2.651208546	-1.768588051
KIF4B	-2.252566723	-2.645476114	-1.141101811
TAS2R4	-0.112317753	-2.64367213	0.559084482
SNORD134	-0.731413036	-2.628290153	-0.010573486
ICA1	-2.258981557	-2.627323076	-2.166217086
NECTIN3	-3.126840741	-2.624383247	-0.911824615
TAS1R3	-2.434973868	-2.621497203	-1.695865061
CTSH	-1.9083529	-2.619513292	-2.281396876
CELSR2	-1.957599893	-2.613150298	-1.198728776
KIAA0754	-3.046352933	-2.612761017	0.583133756
CSPG4P12	-0.230189398	-2.611292283	-0.433048358
TMEM145	-0.405072792	-2.610053909	-0.640789996
MIR5004	-1.144389909	-2.606657572	0.331205908
THBD	-1.183035551	-2.606260271	-1.996552501
F2R	-2.198358886	-2.591546608	-2.484849221

SYNE3	-1.49033973	-2.574106645	-0.800575853
SCARNA8	-1.143328712	-2.56903576	-0.201699679
OSBPL5	-2.22264328	-2.568149335	-1.897145007
TNXB	-3.61286873	-2.561096203	-3.650146138
RPL12P12	-0.128495534	-2.554037991	-0.720856469
IGF2BP2	-2.166021989	-2.550961734	-1.904505915
KCND1	-1.751756614	-2.545845021	-1.09950212
CRACD	-1.464061734	-2.543562526	-1.032660477
NCR3LG1	-2.493659456	-2.543134124	0.072500314
MIR3164	1.090537581	-2.540667041	0.529732317
COL25A1	-0.819901117	-2.535212857	-2.180513331
KCNK6	-0.979739901	-2.534217938	-2.244818371
SLCO2B1	-1.575607296	-2.525270672	-0.42342255
LOC100422717	-0.957114876	-2.520876427	-0.36343071
HNRNPA1P15	-1.829372626	-2.514365282	-0.412294176
LINC00707	-2.34141105	-2.511760263	-2.522494399
FAM131C	-2.853361141	-2.510199476	-2.091390173
FST	-2.023409975	-2.506519622	-2.674459775
DYSF	-2.07281243	-2.505612942	-1.454788134
PGAM1P7	-2.499905991	-2.503447133	-0.829460883
SLC35F1	1.081757528	-2.498750456	-0.416903329
LARGE1	-3.740134234	-2.48301322	-2.753734256
NLGN4X	-4.019710801	-2.478250291	-0.851422603
KCNQ5	-3.142507066	-2.475991791	-1.859365478
HYAL1	-2.294324112	-2.473086087	-2.305229189
PPP1R3G	-0.113925049	-2.472726623	1.200982534
STX1B	-1.569355117	-2.460640201	-1.652686918
PRC1-AS1	2.097028963	-2.456595121	2.01341864
LAMA3	-1.953504567	-2.454727212	-1.295358344
LOC100422296	0.413701344	-2.451740737	0.252949528
S100A16	-1.641259858	-2.446196302	-1.94479289
LOC105372493	0.583854643	-2.445884732	-0.140964228
KCNN4	-1.786402143	-2.444100396	-2.077468917
LYPLAL1-DT	-2.760687112	-2.436545988	-2.387908512
C9orf47	-1.461310255	-2.434894177	-0.159732428
LSAMP	-1.951323539	-2.432438875	-1.60106864
EHD2	-1.763782546	-2.425338126	-1.443692364
HPGDS	-2.201768457	-2.417849116	-2.108975568
KRT8P33	-0.904281046	-2.415091463	0.240078791
MTMR7	-1.51333811	-2.409289018	-1.98962859
CRYZL2P-SEC16B	-1.794289614	-2.408809225	1.783815434
RTL8B	-1.925423569	-2.404420396	-1.034574815
SLC15A5	-0.960985273	-2.404404621	-0.887545315
SLC35F3	-2.370687835	-2.401489555	-2.499597066

CNNM1	-1.766760041	-2.400312498	-1.78630054
CPZ	-0.820438082	-2.395962835	-1.076077729
ZNF471	-2.697622827	-2.390582235	-1.440148015
SLC2A13	-2.070160167	-2.38828968	-1.406756315
NAV1	-2.154639815	-2.385190919	-1.254831321
HSPD1P11	-1.841162388	-2.38144225	0.291639851
KCNQ2	-1.640355789	-2.380707089	-2.293595857
ISY1-RAB43	0.546266633	-2.378063332	-0.294319754
LOC101928728	-0.247881254	-2.372534491	0.568485695
SYNPO2	-1.752232004	-2.369372565	-1.552310153
SHC3	-1.626493161	-2.349839118	-1.142665203
PEG10	-2.32735056	-2.345631985	-1.754229583
RPS19P3	-0.916229116	-2.344988341	0.385840571
SNORD131	3.707634977	-2.338781772	-0.590155413
RAB3C	-2.191973118	-2.338384162	-1.950618419
RAPGEF5	-1.958911085	-2.336308554	-1.579261744
LRRC4B	-1.876406614	-2.336031111	-2.274479042
ATF4P1	0.116629401	-2.332980219	0.836116493
EIF2S3B	-2.9633146	-2.331320056	-1.43721028
AGPAT4-IT1	-2.75262406	-2.324771863	-1.406537584
SNORD116-14	0.807135501	-2.323746547	-0.319889522
PDE8B	-1.681058534	-2.320988484	-1.436259324
FAM174B	-2.945277168	-2.302633937	-2.134133095
LOC100421976	-1.588490183	-2.302234497	0.189474653
SPINK6	-2.286378408	-2.301381629	-2.455451751
RAPH1	-2.248939119	-2.295947481	-0.788720647
SH3PXD2A	-1.87273439	-2.290776676	-1.359070705
PCDH11X	-1.761863795	-2.287349587	-0.151333961
EML5	-2.88321053	-2.278423577	-0.223235043
F2RL1	-2.66392003	-2.274147732	-2.609575675
CGNL1	-1.813429351	-2.266395863	-0.935468218
ME3	-2.795974376	-2.266143894	-3.995909034
SEC14L4	-1.813228448	-2.263494438	-0.923011142
VGF	-2.093798031	-2.263460754	-1.970652481
ZNF837	-0.551204219	-2.256382136	-0.087142819
MATN2	-1.033010389	-2.25485105	-0.638602896
HERC2P10	-1.813849476	-2.254540878	0.191996734
ZNF573	-2.169041659	-2.254112563	-0.647824015
PPFIA4	-0.431121421	-2.251279752	-0.00248932
BICDL1	-1.928585233	-2.244843881	-2.025400302
PCDH11Y	-2.306197739	-2.236950166	-0.715073292
TRIM67	-2.141868293	-2.22729478	-1.423714853
LOC102724434	-1.089600883	-2.220866855	-2.165539608
AFF3	-1.881870261	-2.206494041	-1.471227545

POC1B-GALNT4	-0.17874697	-2.20290429	0.425323962
AMOTL1	-1.816593258	-2.201800031	-1.044679427
MIR6895	0.228266097	-2.201278096	0.005146049
BSN	-1.767515571	-2.199360814	-0.548452349
GHR	-0.510784099	-2.198646759	-0.482070324
MIR196A1	0.614515613	-2.192296951	1.01989988
ATF4P2	0.116614832	-2.190054534	0.836136055
BMS1P2	-1.582840613	-2.189748441	0.242706372
H2BW4P	0.062626131	-2.18957652	-0.303795614
MAOA	-1.833611843	-2.189337254	-1.400862156
FAM106A	0.540507124	-2.185834427	1.219665285
LOC100507437	-1.060959332	-2.182378432	-0.745459964
GREB1L	-1.332582312	-2.181397221	-0.592373969
RLIMP3	-2.286602764	-2.179540023	-1.079047616
RNF212	-2.541905332	-2.176886266	-3.410319386
L1CAM	-2.071059675	-2.175364509	-1.507492408
ELOCP19	-1.047366898	-2.169552251	-0.436784095
CDKL5	-1.840596402	-2.169365423	-0.715415364
ITGA11	-0.710181682	-2.167475958	-0.549513569
SNX18P17	-3.194440044	-2.166390089	-0.634079978
PNPLA3	-1.630222037	-2.160011807	-0.670671542
LOC100131465	2.089272327	-2.153736755	0.163505326
RNF165	-0.822568542	-2.151582519	-1.5420971
ZNF876P	0.450089409	-2.143726237	0.764247729
IER3-AS1	-1.697733981	-2.142366483	-0.213133014
FALEC	0.988093847	-2.138904089	-0.127624036
SNORD136	-1.149992956	-2.134714974	0.125997881
APC2	0.310675578	-2.132240827	1.031273352
TAS2R15P	0.468111931	-2.12995334	1.219829172
BMS1P7	-2.26498173	-2.12692547	0.530784129
SNRK-AS1	0.21126805	-2.116276917	2.177580594
CDH7	-2.336486762	-2.115606084	-1.517492127
FRY	-1.871632472	-2.112890502	-1.522804981
PWARSN	-1.581266105	-2.107955725	-0.261472951
SNCB	-0.673917442	-2.106259888	-1.481864792
SLC35E1P1	-1.472606413	-2.105817479	-0.358309207
PRPH	-0.948331835	-2.105532924	-0.305997046
SENP3-EIF4A1	0.955159778	-2.10512116	1.08110717
DFFBP1	-0.622356924	-2.104950636	-0.007508965
LOC158948	-1.675375369	-2.103680026	-0.718493707
DAGLA	-1.607611018	-2.099785071	-0.739311072
COL1A2	-1.988788354	-2.094517546	-1.904957289
PRRG2	-0.83153733	-2.094159804	0.972739055
LINC00342	-1.273821294	-2.092563844	0.362114023

ZNF252P-AS1	1.351810097	-2.0891709	-0.086475182
PARD3B	-1.301946259	-2.084230211	-2.203562366
PWAR5	-2.063876154	-2.080463942	-0.475667463
HERC2P8	-0.546154481	-2.080186109	-0.346216553
EPS8L2	-2.421181663	-2.077346362	-1.732307325
ACTG1P20	-2.046552942	-2.07723954	0.51614368
TIMP4	-2.899610601	-2.072276848	-1.841136619
SEPTIN14P15	-0.180347536	-2.071594478	1.51788425
LOC114483834	-0.529631238	-2.070786033	0.517978861
SDK1	-2.041040305	-2.069680244	-2.126177527
GRAMD1C	-1.211129516	-2.068738103	1.175763516
PLEKHH2	-1.640916812	-2.066002388	-1.941474301
CA5BP1-CA5B	-1.43324781	-2.057963092	-0.689557309
CACNA1H	-1.499334902	-2.054427779	-1.044075206
SLC6A9	-1.88374463	-2.049110351	-0.710039337
HECW2	-1.925776898	-2.046030402	-0.482180137
TM4SF19	-1.77344018	-2.043421401	-1.197914408
CSNK2A3	-1.507542802	-2.042920617	-0.990807538
LOC100505938	-1.97397494	-2.040673059	-0.193144875
NPHP3-ACAD11	-0.557621419	-2.038446857	0.357493152
ANAPC1P1	-3.710975718	-2.036048262	0.189504155
GUSBP9	-1.447642523	-2.032906939	-0.035052456
DLGAP1	-2.282735577	-2.031997272	-1.618061105
ZNF558	-1.958310943	-2.030809137	-1.077417779
FAM66E	-2.513610716	-2.030151129	-0.387340891
AKAP6	-0.975893504	-2.028818352	0.085226447
LOC100129917	-1.477636648	-2.023299851	-0.047534298
CBLB	-1.838731526	-2.022939091	-0.847133783
PCDHB18P	-0.162847631	-2.01983404	1.35303195
ADAM19	-1.819637109	-2.016794461	-0.650682948
RETREG1	-1.776324883	-2.01447629	-1.468916513
LINC01537	-2.663971905	-2.011681443	-2.327872259
KCNC3	-0.923278651	-2.009894477	0.182947827
LRP1	-1.679810018	-1.997397656	-0.650836227
PHKA1P1	-2.403575241	-1.997214436	-0.233511737
LINC00266-1	-1.787535383	-1.996920537	-0.29674327
ZNF81	-1.763755163	-1.996008656	-0.73232338
FOXO1B	-0.585508891	-1.995423481	1.220008966
MAP3K15	-1.244748654	-1.994577235	-1.292454848
BMF	-0.446881112	-1.993616421	0.209848039
ADARB2	-1.55767471	-1.99332823	-1.079832009
CTBP2P8	1.276089169	-1.99173926	-1.175156797
LINC00999	-1.746957845	-1.98974155	-0.423117199
CPED1	-1.916257805	-1.987718444	-0.955511886

MAGEB2	-1.908208951	-1.981730316	-2.201232718
ITGA1	-2.118985414	-1.980348068	-1.323151173
LINC00926	-0.119240388	-1.974558108	0.472677612
BRSK2	-1.813253822	-1.973642824	-1.788412044
CRLF2	-2.472444161	-1.972791345	-0.906326578
KCNJ11	-1.769285169	-1.969704409	-2.321889806
AFAP1L2	-1.396096058	-1.96815586	-2.035481562
PNMA2	-1.751852052	-1.967428351	-1.097148923
KCNMA1	-2.400095551	-1.961286686	-1.2568443
C14orf132	-1.486779118	-1.959201945	-2.272522736
FES	-0.219189193	-1.956590224	1.229326248
MIR6759	-0.742425825	-1.952552838	2.186358815
ADARB1	-1.782168672	-1.950965158	-1.324246241
TMEM151A	0.045367028	-1.948225335	-0.43769306
SLC7A5P2	-1.733315662	-1.947046233	0.108272758
NKD1	-2.387603109	-1.945543529	-0.659088531
KCNE3	-0.083183224	-1.942492235	-1.561188682
PPEF1	-1.931990482	-1.940156231	-1.895935904
LOC151457	-0.929208515	-1.939484309	-0.952132118
RNF148	-1.130526165	-1.936525589	2.271820154
PSD	-1.86315721	-1.935995598	-1.793894363
ESPN	-1.684716876	-1.935550916	-0.977242358
MTAPP2	-1.337399429	-1.933970978	-0.644810218
SNORD143	-0.256541745	-1.932995908	-0.719953718
CSPG4P10	-2.029545611	-1.931864568	-0.777198392
SNORA84	-0.771679703	-1.93027677	1.426460955
MYH15	-1.527562263	-1.928282089	-1.712799131
SHC4	-1.742532116	-1.927724739	-1.598138394
RAD51-AS1	-1.708268712	-1.924367271	0.3338849
SNORA71E	-0.253340408	-1.922172691	0.386181381
ATP2C2-AS1	-0.285227552	-1.918027365	-0.949817995
CAMSAP3	-1.763076124	-1.917810804	-2.831649663
SNORA23	-1.809823468	-1.913605346	1.065193663
MRAS	-1.407229956	-1.911173002	-1.368920723
NDRG1	-1.397673001	-1.90710394	-1.120367867
RPS6KA2	-1.894723467	-1.905418621	-1.29057773
CREB5	-1.925623524	-1.90398245	-0.947026465
SNORA63E	-0.308920752	-1.903610192	0.243394033
LINC02535	-2.220557848	-1.90360618	-1.950553699
SNORA74D	-0.544896136	-1.902375395	-0.491299191
RINL	-0.939011371	-1.90231343	-1.01767322
TSPAN5	-0.904772119	-1.895702839	-0.29093947
SORL1	-3.064251297	-1.895568526	-1.046005289
FERMT1	-1.660317306	-1.895326185	-0.935581487

FOXP1	-2.12247306	-1.890889211	-1.21943449
FAM239A	-1.370858827	-1.889728305	-0.649943541
DNM1P47	-1.261836132	-1.886985713	0.003907399
TMSB4XP6	0.737351005	-1.886950882	-1.475855308
PDZD4	-2.05776351	-1.884378072	-1.970500082
HSFX1	-0.339581999	-1.882494038	-0.673013641
HSP90AA6P	0.034896516	-1.882270159	1.555205775
ARID5B	-0.879237177	-1.881341251	-0.974201317
KANTR	-2.17524293	-1.881247991	0.821859784
TAS2R31	-1.054885988	-1.879826513	-1.014619059
HOXC5	-1.805615601	-1.878532178	-0.931041286
ABI3BP	-1.296811613	-1.873583474	-1.698963268
LOC101929269	-0.706655747	-1.870958091	1.024076038
SYNE1	0.428671566	-1.870910016	-0.153558918
OBSL1	-1.692080951	-1.869187082	-2.614259366
ATP10A	-0.74443098	-1.865751321	0.852080565
SMG1P1	-1.611222297	-1.863657459	0.287223953
ADAMTS6	-1.527861525	-1.862507203	-0.711051125
LOC100420174	0.333345235	-1.86191949	0.673965959
C10orf143	0.438237832	-1.858419614	-0.862512625
SLC17A7	0.058570048	-1.856573757	-0.417066561
SIX3	-1.407483371	-1.855887211	-1.047645233
NBEA	-1.828677972	-1.855314689	-0.042150878
ZNF852	-1.581106263	-1.854100087	0.580342231
LINC01126	-1.022273924	-1.852155146	-1.27835034
POMK	-1.576216887	-1.851697656	0.188300765
C3orf80	-1.731039427	-1.848970132	-1.405410902
MIR374B	-0.323309975	-1.848790295	0.034911216
TUBB4A	-1.316619564	-1.846741822	-1.360786935
ABCC9	-1.386176907	-1.84611313	-0.762727325
PRKXP1	-1.73419983	-1.84581224	0.277858142
ZNRD2-AS1	-1.305727988	-1.842711604	-0.152020993
SMG1P4	-1.622847421	-1.841233053	0.228543527
ARHGEF37	-0.764875118	-1.835581656	-0.845616743
SNTB1	-2.188591723	-1.834858207	-0.572411874
CAMK2B	-1.481676515	-1.834140524	-2.724935252
GAB3	-2.322927234	-1.832935454	-0.07999361
SEMA6A	-1.041313094	-1.832628518	-1.550568767
LOC780780	0.728422593	-1.831072494	0.871511143
TEX11	-1.415844245	-1.830210934	-1.565070254
PTK6	-1.285677561	-1.829352151	-1.67502162
LINC02875	-0.345122668	-1.829009812	-0.336422641
MPP4	-1.166229371	-1.827740229	-1.287851296
ZBED6	-1.678992212	-1.824789773	0.591443826

KIAA0825	-1.89594489	-1.821841624	-1.091567576
EPHA4	-1.500407949	-1.817583765	-0.721920328
LOC107985246	-1.113147982	-1.816959151	-0.023173539
DNM1P41	-0.555478985	-1.815634984	0.002081723
B4GALNT3	-1.561120769	-1.815304668	-1.673488588
PAPPA2	-1.554834771	-1.812871357	-0.785905826
LOC105370708	-1.6361317	-1.81234344	-2.095155647
BMS1P1	-1.543854655	-1.812262032	0.040646261
ACTBP2	-0.607179412	-1.8100029	-1.430850027
LOC647150	0.973159561	-1.808041316	1.129835108
DKFZP434A062	-1.287392732	-1.805493758	-1.373330023
PLCXD2	-2.040700045	-1.805287568	-1.337447506
ZNF582-AS1	-0.33039792	-1.804941812	-2.036272125
ARFGEF3	-1.828161922	-1.799929654	-1.224001294
MIR4315-1	-1.119909464	-1.799440086	-0.325788855
ADAMTS16	-0.870174577	-1.799005881	-0.739390907
PLTP	-1.53291367	-1.797261763	-1.768521731
CSPG4P11	-0.41214528	-1.794835324	-0.04589096
LOC100506178	-1.561981192	-1.793150397	-1.310275345
PACERR	-1.308217603	-1.792760864	-1.411066176
LOC402096	-3.063047657	-1.787460017	-0.062202658
LOC440300	-0.910813943	-1.787247124	-0.540932995
EP300	-1.303072348	-1.783516033	-0.008065
ALDH1A3	-2.749692527	-1.783352952	-1.609786852
MED14	-1.542692492	-1.783031137	-0.696571956
LOC202181	-1.931470938	-1.781998984	-0.926293876
OLFML2A	-1.149515737	-1.780733345	-0.508443218
ECHDC1	-1.800361064	-1.779469226	-1.691329813
CCDC87	-1.9195678	-1.776105853	-0.241189006
TLN2	-1.838698266	-1.775742373	-1.098655913
IGSF9B	-1.393764978	-1.775509502	-0.210280891
LOC283922	-1.431470958	-1.773435989	0.071165108
SMG1P3	-1.402694927	-1.771043083	0.326722745
LOC284009	-0.58342342	-1.769905912	2.060809229
EPHA8	-1.427382721	-1.767217158	-1.74175492
TMEM170B	-1.25787619	-1.767198232	-1.065940949
AREG	-1.202385118	-1.766354013	-0.084137671
CXCL11	-0.816199798	-1.765529741	-0.951248975
USP51	-0.789784359	-1.762767952	-2.302985981
EIF1B-AS1	1.554343158	-1.762202593	-0.24529176
CARD14	-1.486474329	-1.759118982	-0.613381575
MYO5C	-2.345151395	-1.758182226	-0.62179294
SNORD103B	-0.677140982	-1.75652405	0.194264671
LOC317727	-2.405037807	-1.755184653	-0.660918357

AGGF1P1	-3.981249158	-1.754159847	-1.176707655
ACTG1P4	-5.352159119	-1.752479458	-0.530058485
KCNH3	-0.912077608	-1.751568422	-0.988104734
NLRP1	-0.989464437	-1.750733797	-0.596277431
LOC104968399	1.427737511	-1.748493358	1.385606194
NSFP1	-1.075249256	-1.746089887	0.002977251
MSI1	-1.783350624	-1.745955633	-1.150708985
PRICKLE1	-1.756828956	-1.74524868	-1.464205455
CYFIP2	-1.649227441	-1.745243709	-1.052183533
N4BP3	-0.539706547	-1.743859928	-1.572307106
SLC52A1	-0.181257624	-1.742775025	-1.176261781
MUC12	0.866267305	-1.742430953	-0.204009951
CYP19A1	-0.711724881	-1.738674061	-0.747659948
HTR1B	-2.33914198	-1.738027079	-1.579637167
DOCK9-DT	0.797654824	-1.733494129	-1.782374137
MIR4784	-0.790041634	-1.732364233	1.045352532
ZBTB32	-1.340471962	-1.731504174	-1.864548437
KMT2D	-1.469713567	-1.731087307	0.102881278
SLC9A2	-2.124163649	-1.729067882	-1.545853794

Supplementary Table S14. Up-regulated genes in A549 cells treated with TLR2, TLR4, and TLR7

Gene_Symbol	Fold increase (TLRs vs. vehicle)		
	TLR2	TLR4	TLR7
MIR4260	14.30908363	15.85570351	13.09135709
MIR611	15.72162792	14.62601698	8.816343705
USP17L13	5.300804803	6.756439956	7.823441302
USP17L17	4.953113612	6.481001494	8.012786926
RNU1-39P	1.99619843	6.310875827	7.147820815
PGBD3	4.826213949	6.267448306	5.746755268
USP17L12	6.601450624	5.899409014	5.721963431
ST13P3	4.847739994	5.479757738	3.176166199
CCDC58P3	4.314642605	5.465956809	5.569290762
RPL12P12	3.79824497	4.67930415	5.839817905
ST7-OT3	3.099142158	4.488409839	5.726410155
CDK2AP2P2	6.637216313	4.283790775	5.70986452
LOC440896	3.729999266	4.135036958	3.121027357
MIR6733	6.028201354	4.124542849	5.252424896
RPL17P5	3.740205159	3.915968004	4.150681107
SNORA31B	5.491853096	3.906890596	3
RBBP4P1	1.908928997	3.735072563	3.945328379
CPAMD8	3.146657547	3.731274046	1.286646068
NDUFA4L2	3.338800279	3.728079614	2.115470007
ISCA1P4	2.804583348	3.727063295	2.3467195
HNRNPA1P21	3.327146939	3.434487339	4.247306688
MIR6746	1.474242341	3.418737352	2.37351761
LINC01252	2.02790356	3.396550065	2.215164504
RAB4B-EGLN2	1.518567999	3.340628963	3.540005517
CCDC58P4	4.641696819	3.291017582	2.524919173
CBS	3.067855578	3.173325725	1.996986135
DUSP8P2	3.711630659	3.089711881	1.055764549
INHBE	2.904142312	3.088724936	2.279592604
MIR7111	2.923264194	3.083944626	2.772171864
EIF1P3	4.479796481	3.051073576	3.397031986
THCAT158	1.905357655	3.027083602	1.457967609
MEF2B	2.847304618	3.023100092	2.254849549
SNTB1	2.692287097	3.009457312	2.481829205
MIR10393	4.75470263	2.969563597	4.047806947
HMGN2P1	1.552144939	2.952670709	1.596334145
MIR6750	1.800691002	2.926653897	1.062199854
YRDCP1	1.65397347	2.918368868	2.272779543
MIR4519	2.128931118	2.917785823	1.865645791
USP17L19	4.953113612	2.89566334	6.727287277
RPS2P35	3.68740977	2.863784022	2.582566429
PCDHGB6	2.910991838	2.822532072	1.317173565

FUT1	2.821208457	2.804692933	1.882317037
MIR138-1	3.316820946	2.788009	2.654025245
ADM2	3.13743589	2.777131042	2.602196484
MIR628	1.245464429	2.774696491	1.585693464
MIR4315-2	1.5978162	2.746762554	1.444052897
YPEL1	2.508176001	2.731606447	1.037753436
PTMAP7	2.032203882	2.71461441	2.992102988
C11orf96	2.547088721	2.705947557	3.167993416
PPIAP54	2.91881819	2.681664414	2.639158911
ASNS	2.540844374	2.581906163	1.461765621
IGFBP1	3.068320255	2.568683147	1.244697304
GOLGA6A	2.348698749	2.549787865	2.351929739
NPM1P35	3.400729663	2.539062237	2.771932199
NUPR1	2.481264874	2.529977966	1.387542595
PPIAP19	1.245705839	2.495392535	1.52692251
FLRT1	2.466591475	2.475419703	1.831229878
ATP5F1AP8	2.649982074	2.393318959	1.002385953
ARPC3P1	2.91791599	2.39171991	2.224183477
MIR3620	1.914090357	2.390235329	2.497212365
LGI4	1.535234812	2.363010346	2.231590465
LINC00605	2.671466636	2.359965347	1.026536488
COMMD3-BMI1	3.004214806	2.34336234	5.087890846
BCAT1	2.153024313	2.335229414	1.343981952
ULBP1	2.412397518	2.319197351	1.402530394
TDRKH-AS1	2.366850566	2.316328082	1.563330048
ACTG1P24	2.110176197	2.311025415	2.263296593
RPL18P11	2.697601049	2.261855452	2.016519473
NUDT9P1	1.746927481	2.260103063	1.929532879
LERFS	1.191996868	2.221066057	1.288285383
SOWAHD	2.045408021	2.214466783	1.750177706
MIR30C2	3.506222064	2.199400295	3.562247971
THEG	2.673021343	2.181749485	1.194602169
LOC100133007	2.307305772	2.16245584	2.862588888
MIR3189	2.278338022	2.125713521	2.349822198
LTB	1.898298268	2.1116942	1.304911357
NUDCP1	1.840840225	2.086894002	1.698829681
MIR3615	2.094312607	2.080612863	1.104151424
LOC101927040	2.78268923	2.079368522	1.373053252
BTF3L4P3	1.774215426	2.066093157	2.89673918
RPL36AP46	1.366338849	2.057651706	3.166857147
TCP11L2	1.786161761	2.004439293	1.102226713
PSAT1	2.004954936	1.994283386	1.037021676
SUMO2P3	1.822013609	1.982088013	2.229153663
ZNF396	1.621761271	1.981921142	1.752257339

MIR22	1.587921876	1.970823425	2.107021
ENPP4	2.049199364	1.968117777	1.045880052
LOC152594	1.665783032	1.93843114	1.793903469
ICAM1	1.783736853	1.930853369	2.051574251
SCARNA5	2.536666362	1.923386887	2.205617247
EEF1A1P10	3.032209335	1.920448701	1.76380364
GNG12-AS1	1.936880385	1.902228619	1.16156493
ALDH1L2	1.766549328	1.900985158	1.099858405
TRIB3	2.02901893	1.896375009	1.750801687
ABCG1	1.899072494	1.89231458	1.718546066
KIF21B	2.049119323	1.890770657	1.528099465
LCN12	1.165442391	1.881925456	1.146586563
H4C2	1.494470389	1.880032877	1.41260628
MIR6858	1.565528078	1.877159557	1.126002824
LOC101929243	1.661703477	1.876002411	1.820938723
MYO16-AS1	1.581860674	1.851177685	1.646370195
PRDM8	1.969047848	1.847513313	1.703057403
GPT2	1.898564595	1.831033699	1.339627355
MUSTN1	2.225541256	1.817456577	1.291025314
bA395L14.12	1.028038724	1.805949206	1.846306762
RPL26P4	2.048109166	1.802864934	1.152064268
MIR4648	1.799553895	1.79662549	1.075885514
CXCL3	1.626171015	1.777565301	1.865190939
RNU6ATAC	1.494963479	1.777310218	1.603375559
RELB	1.683565233	1.768867442	1.075113262
BGLAP	2.076206015	1.765437405	1.091912777
IRAK2	1.626983413	1.734761725	1.384299424
DUSP6	2.203441824	1.725013986	1.649385456
RPL7AP45	2.029445768	1.705960541	3.408150407
MTVR2	1.625503377	1.694627908	1.459962575
PCBP2P2	2.407122395	1.654128863	1.770995039
SDR16C5	1.919583779	1.650013527	1.002212666
LOC100419799	2.335344032	1.629143995	2.086477965
SNORD20	2.155618726	1.628830863	1.745510459
LOC101927989	1.740688932	1.623242063	1.637108102
CHAC1	1.915635069	1.620662067	2.607663509
MTHFD2	1.713166808	1.599431866	1.104579374
C5AR1	1.559090362	1.594127457	1.025866458
TLDC2	1.179664012	1.583286679	1.226728798
LOC100420326	1.179017031	1.577458866	1.88582606
KIRREL1-IT1	2.276593267	1.55519795	1.643327472
CXCL1	1.264081372	1.537416094	1.482991413
KLK14	3.078171321	1.527513602	3.358683974
SEPTIN14P2	1.083257764	1.5115339	1.265451206

NOS1	1.234469456	1.510861641	1.312550932
MAFF	2.125486796	1.507089729	2.130612528
XPOTP1	1.724906226	1.500151182	1.54657931
PCDHA13	1.343542976	1.495987531	1.721447043
NUDCP2	2.066347312	1.49511039	2.085882567
C15orf65	1.775319794	1.486866171	1.073473831
MIR1182	1.099276461	1.484630039	1.482691055
TLE1P1	1.465444313	1.471584902	1.216285714
ZNF91	1.472952291	1.467978619	1.178259779
CXCL2	1.438890085	1.463175711	1.487052114
RPL10AP9	1.790289676	1.462910915	2.737677481
NFKB2	1.450281584	1.462125915	1.17625291
HSPA8P1	1.96037858	1.452185766	1.278583233
ZCCHC12	1.948969891	1.451940047	1.860467606
CYP4F2	1.167885449	1.442775866	1.264447971
SETP22	1.50825146	1.440428239	1.61091004
MAPK10	1.509368816	1.433413218	1.270916311
GRM2	1.138191694	1.412898107	1.286475729
SYT5	1.379451328	1.411637016	1.328497612
POM121L9P	2.1712179	1.39356795	1.398740205
EFCAB10	1.54159862	1.389447433	1.791563205
NKX2-8	1.268659913	1.36173891	1.262029989
ERN1	1.428587199	1.351903486	1.005281383
ARHGAP9	1.965589124	1.344849076	1.043168649
LINC02447	2.024102159	1.336543515	1.008013046
MIR3605	1.634242032	1.329636178	2.402995002
LOC100420429	1.653923148	1.318137745	1.192813655
OSTCP7	2.611914282	1.313660479	2.218736892
BHLHA15	1.339573344	1.306951375	4.70889103
LINC02762	1.231511648	1.305429716	1.052762917
LOC646890	1.404849038	1.297260673	1.682953923
SPRY4-AS1	1.623835445	1.294982025	1.085848902
NKPD1	1.238978441	1.285422473	1.146242893
RNASEK-C17orf49	1.872866551	1.284018277	1.397349471
NME2P1	2.157325214	1.265764924	1.966120785
ATF4P3	1.479837274	1.259159714	1.731850964
KRT8P36	1.135372444	1.246313681	2.128588416
USP17L14P	2.954381711	1.238315919	4.678685471
CCL20	1.131146007	1.212615577	1.768227231
SPRY4	1.564561723	1.212556233	1.709167461
EMC1-AS1	1.891377696	1.209893184	2.125875666
EXTL3-AS1	1.311290892	1.200058326	1.177724922
MIR6743	1.5669352	1.195546405	2.303477881
DRD4	1.397005781	1.193140302	1.072844714

MUC17	1.016861036	1.191898961	1.262523947
CARD9	1.279031533	1.189665689	1.661096363
UNC13A	1.490637717	1.143303287	1.026072376
SPRY4-IT1	1.850981156	1.142908078	2.047799637
NTSR1	2.103830202	1.132301797	1.883410509

Supplementary Table S15. Up-regulated genes in H1299 cells treated with TLR2, TLR4, and TLR7

Gene_Symbol	Fold increase (TLRs vs. vehicle)		
	TLR2	TLR4	TLR7
MIR6861	1.321928095	10.83819094	13.69359585
MIR3940	3.703350844	7.95368076	2.814854986
MIR10394	2.861530197	7.186663328	2.10203985
RGCC	4.506800817	5.587387632	3.409382002
CATSPERB	4.548904951	5.258747891	5.572652256
SNORA86	1.569443083	4.724714464	2.922197848
ADORA1	4.70110749	4.683755215	2.816682636
RASGRF2	2.284561835	4.676123533	3.689372168
JMJD1C-AS1	2.026365143	4.377881937	2.395240818
GRAP	4.104498992	4.06491423	4.066941074
ANKRD22	4.216921296	4.036535747	3.400686485
FAR2P1	2.921162794	3.876255369	4.876789786
RPPH1	2.795572485	3.862917075	2.880286076
COL26A1	3.277047706	3.839431112	2.995194802
TC2N	3.974802525	3.779394119	3.987394994
OLFML3	4.277099042	3.776344223	4.109535313
HHIP	3.749292816	3.668328287	4.048338873
PPIAP42	2.319551016	3.570981308	1.167030423
SLITRK6	1.901689192	3.555651109	1.927280903
TGFA	3.463682548	3.553403968	3.229606397
HNRNPA1P33	2.930235002	3.518128206	2.113525665
VGLL3	5.34335333	3.482383661	4.424480687
VILL	3.732761472	3.45319649	2.756011566
ADAM12	3.800634487	3.42564738	4.176119892
ACOT12	3.371201219	3.399137652	3.003857713
RMRP	2.154526003	3.38534358	4.466412598
MAL2	3.14517484	3.294041855	3.624418878
C10orf90	3.294961503	3.275774932	3.086863298
NNMT	2.815201813	3.230155659	2.797932209
AGAP5	1.341458956	3.228627263	1.162486712
FUT9	1.713578284	3.209117317	3.377180366
TESC	2.965102106	3.16525331	2.392320758
MIR664B	5.023846742	3.065643616	4.209453366
CASP1	1.785339094	3.021508308	2.739729731
IGFBP3	3.309400737	3.018586713	3.053909257
LOC100505915	2.055076828	3.014799202	4.045661833
TVP23C-CDRT4	6.113169477	2.99146021	1.246361187
BMPR1B	3.08382596	2.989896717	3.9009104
CTNND2	4.219432215	2.973701295	3.356392381
ANGPTL1	2.312799902	2.970135383	4.281770968
SOST	2.916147402	2.937280564	2.840679002

TCERG1L	2.654173979	2.933001917	2.341526586
EMILIN2	3.141183506	2.908819657	3.654664773
GKN1	2.17237726	2.898600355	2.606119557
THEMIS2	2.662135748	2.873236995	2.217054684
GJA5	2.946737626	2.832821651	2.698643217
MRPL42P5	3.290045509	2.813860542	1.333331432
TMEM255A	1.468767871	2.780829757	4.601560548
LRRTM4	2.609202222	2.747778588	3.29629
EPHA7	3.152885253	2.743753255	4.005248009
LINC00326	2.268228356	2.739741644	2.37298339
SLC26A9	2.903004177	2.732752546	2.827143785
GPC3	2.842094279	2.719327079	3.608783281
LINC02861	1.689572625	2.708070477	1.594846369
FRMD3	2.90548667	2.695586644	2.915072597
MGAT4C	3.080959507	2.669117606	3.573699807
RNU1-1	1.257653128	2.635806899	4.440778817
RNU1-3	1.683433194	2.62478696	3.776639965
LMO1	1.889507521	2.622365951	1.152787902
TGM2	2.751127554	2.60179992	3.035536278
AFF2	2.97697642	2.593888024	2.689010954
RNVU1-18	1.670616796	2.545352248	4.708251242
HSPB2	2.126644714	2.544294917	2.092613651
SPARCL1	2.111246857	2.512172914	1.519544061
CFAP52	1.026903459	2.464149621	1.893690583
ZNF738	1.965915924	2.436126319	2.361962281
ABCB1	1.926237014	2.429556078	2.220045838
PPIAP46	2.66833162	2.422246475	2.514880775
BMPER	2.502261217	2.371564387	2.700823944
COL9A2	2.072959944	2.362779387	1.457752988
LPL	1.830637041	2.346144201	4.144873005
HCAR2	2.573662488	2.336438618	1.724964735
KCNK15	1.051742395	2.324417405	1.2761545
GRIN2D	1.767488029	2.315907676	3.183137336
RNU1-4	1.376437784	2.313245723	3.671689408
HEY2	2.18015242	2.305011398	1.912007559
RNU1-2	1.767387469	2.300864851	4.462556759
LUM	1.890351993	2.300765106	2.152593651
RNA5S17	1.408961577	2.274808164	1.486884066
SCARF2	2.163008073	2.261775446	1.21420031
STRA6	2.280148187	2.245950347	2.261031208
IGSF1	1.83958907	2.240044066	2.665091123
EHF	1.10944137	2.208566131	1.949415608
MRC2	2.442304324	2.208277489	2.741141084
KRT81	2.1070417	2.205740185	1.736753075

SEMA6D	2.521399818	2.195215757	2.679309499
PLAU	2.095071554	2.175283877	1.940665714
NAALAD2	3.333933191	2.166619785	2.492897866
EXO5	1.931359233	2.165858624	1.219301266
FBLN5	2.051543351	2.164772855	2.248563729
IGFBP4	2.22633503	2.160009738	1.765665204
C3orf49	2.182835197	2.159760116	1.892395933
RDH10	2.178145895	2.144793916	2.515786034
MEST	1.926362031	2.14477938	1.966504792
ACKR3	3.386805365	2.113824794	1.61158005
LIPG	2.324467662	2.103600187	2.367189409
IL1R2	1.140370085	2.091841379	1.504396589
LOC101929128	2.07224241	2.077923417	1.733757113
ID4	2.309014218	2.069773754	1.856975252
GABRE	2.272298348	2.057826119	1.968026251
MCAM	2.242894082	2.056603603	2.323306514
GLT8D2	2.253493219	2.037059081	1.013832022
SOX7	2.21415192	2.022822486	2.216007233
FLJ22447	1.954468384	2.02203948	1.937759854
HEY1	2.063788969	2.010029782	1.885409914
RNA5S7	1.673454594	2.003748861	1.008725136
HPD	1.852162782	2.000595946	1.6705732
DSE	2.240339033	1.985757034	2.316108785
HHIP-AS1	2.839871128	1.981595575	1.460752934
MMP2	2.666156458	1.978597356	2.886709139
TRABD2B	1.394854893	1.978525786	1.840289225
AGAP1-IT1	1.800593999	1.962627297	1.06694159
CYP24A1	2.146321538	1.955205859	2.408775778
MIR663A	1.638057743	1.9344801	3.001211681
LOC441241	5.087462841	1.934257797	2.005817837
CHURC1-FNTB	2.428266016	1.91897995	3.190191234
MIR4680	1.124258103	1.902702799	2.664396968
MIR573	3.050136951	1.898600746	1.72988958
LOC100420528	1.960693043	1.897576181	1.31113326
HES1	2.210588221	1.895311003	1.361770684
CXCL14	1.96899941	1.893084126	1.022305434
ANXA3	1.351294587	1.874071988	1.15957442
COL14A1	1.831182665	1.866614191	2.458981865
TLL1	1.836666637	1.848029717	2.21145119
CPA2	1.585239725	1.841957189	2.284330976
HAPLN3	1.79068798	1.835668316	1.208023987
VAMP8	2.941815563	1.82452468	1.087745473
MICALCL	1.916751454	1.815126271	2.77050167
HOXC13-AS	1.969460972	1.812472569	1.078909591

PPP1R3B	1.956688981	1.806045649	2.537301635
FLI1	3.362972373	1.798359949	4.651182429
HUS1B	1.645950275	1.790974647	1.374834529
LIN28B	2.013723949	1.779350497	2.125583171
SNAP25	1.834384127	1.773775634	1.648520056
SLC12A3	2.182740656	1.764105601	2.59057255
KCNK1	1.832888366	1.747084989	1.496135765
FOXQ1	1.371388299	1.727948568	1.262754597
SNORD102	1.061543856	1.71848887	1.326575544
KRT87P	1.401016811	1.716131101	1.547888651
DEF6	2.01855597	1.71007715	1.008948317
FAR2P2	2.577693013	1.701869154	2.416015966
CHRDL1	2.355653622	1.700971284	3.290634706
SDC2	1.771098103	1.697843072	2.173529695
KCNH1	2.375325266	1.693665869	3.340237575
GSTT2	1.267450533	1.693064583	1.451225982
ADAMTS3	1.881156584	1.687117388	1.509511297
CD24P4	1.832717944	1.654980855	1.57757572
SNORD3A	1.233519459	1.62453737	3.083108168
PDGFA	1.879960512	1.619547678	1.794741586
PTMAP4	1.581895753	1.601306444	1.465499423
EEF1A1P3	3.381342568	1.59803511	2.41288253
NFE2	1.802837391	1.595334567	1.302804774
TRNM	1.149128258	1.584286446	2.122259863
TRNQ	1.013187765	1.557233122	1.856401461
PLAC8	1.442058202	1.540702256	1.063525701
PRAG1	1.243413172	1.53474409	1.969431568
MYL9	2.100542357	1.528015705	1.2922343
PTPN6	2.000094253	1.507624596	2.237194585
MCC	1.520440451	1.496427373	1.964132175
CRYAB	1.843110382	1.494741418	1.746253183
TERC	1.10180274	1.491451195	2.034627833
HMCN2	2.319638763	1.486343115	3.685935388
DEPP1	1.537818156	1.485235058	2.143291356
LINC01503	1.955464137	1.484818964	2.003050059
COLEC10	1.527603121	1.483492487	1.097736802
DTYMK	1.286406843	1.481123867	1.034689407
RNVU1-7	1.643193274	1.478341922	3.229493017
IGSF11	1.756900715	1.477921656	2.046339158
QPRT	1.584916314	1.476415737	1.303257976
DIRAS2	1.120406183	1.462633675	1.320952851
ZFAND2B	1.273498151	1.448835624	1.039791641
EIF4EBP3	2.390665997	1.437631408	1.365602858
RNA5S10	2.237717743	1.426737857	1.519493357

RPL24P2	1.600042841	1.425778295	1.168142566
LZTS1	2.323700364	1.416574089	2.296025802
SH3BP2	1.554469833	1.408257865	1.753472477
CP	1.734539562	1.405932075	2.497365148
TNS4	1.877810274	1.402784096	1.179022194
EPSTI1	1.002637893	1.378832224	1.101351886
HOXA13	2.29221051	1.364180328	2.027297845
PLEKHO1	1.314897623	1.363073534	1.311020209
NLGN1	2.083484	1.362853512	2.348500672
DACH1	1.635346455	1.351297903	1.840448848
RBM20	1.332273493	1.343002724	2.114896221
HKDC1	1.306963354	1.337795297	1.716687089
RNA5S8	1.416875932	1.332006475	1.265406276
RPS24P17	1.59677989	1.32098922	2.248110147
SV2A	1.963403433	1.318252975	1.088125235
HOPX	1.138406605	1.316950057	1.36663488
MARCKS	1.293443478	1.314014227	1.303055666

Supplementary Table S16. Down-regulated genes in A549 cells treated with TLR2, TLR4, and TLR7

Gene_Symbol	Fold decrease (TLRs vs. vehicle)		
	TLR2	TLR4	TLR7
RN7SKP175	-2.678751353	-2.097163327	-1.002036618
PCDHB17P	-1.170095765	-1.45117376	-1.015335181
H2BC7	-1.532659125	-1.574578928	-1.023829588
GUCY1A2	-1.08557188	-1.477659417	-1.039655158
STIM2-AS1	-1.109930277	-1.087426907	-1.040521504
MIR1287	-6.58128135	-4.830879616	-1.041820176
RPL39P19	-1.22737801	-2.752894312	-1.047883001
DIXDC1	-1.836736894	-1.545569641	-1.062413116
MIR4657	-2.401744466	-1.542634984	-1.068944465
SNORD15A	-1.917244607	-1.201416175	-1.080642123
LYPD6	-1.069534823	-2.470880047	-1.120796731
SEPTIN14P7	-3.323450193	-3.707352793	-1.123163293
SNORA41	-1.049678571	-1.554541135	-1.132962155
TENT5B	-1.407526457	-1.235316367	-1.146890436
SYNDIG1	-1.759497671	-2.102319202	-1.152163174
SNORA5C	-1.278206815	-1.159298847	-1.154181666
ALX4	-1.432713585	-1.424787148	-1.165289722
LRRC70	-1.111839474	-1.664034655	-1.168952661
EVA1A	-1.093775851	-1.220068312	-1.173635974
DIO2	-1.625184003	-1.678510681	-1.19824793
GHR	-1.583768661	-1.035364166	-1.244038735
NPM1P9	-1.191847976	-1.129782369	-1.247316577
FAM186B	-1.688839074	-1.851270372	-1.252114304
LNCSRLR	-1.085488613	-1.213023344	-1.264651744
LOC100130238	-1.338947042	-1.174305862	-1.273386739
SNORD100	-1.4279726	-1.578611035	-1.277330606
PAIP1P1	-1.188750453	-1.268408175	-1.279734371
CCBE1	-2.071038997	-1.587093214	-1.3023867
RBMS3	-1.458314313	-1.029053864	-1.324161245
IRF8	-1.886803758	-2.478136126	-1.340303534
BNC2	-1.301704747	-1.248094099	-1.348977135
NPM1P7	-2.078708094	-1.226984226	-1.354875579
RPL26P6	-1.411129485	-2.280813322	-1.360196035
NCALD	-1.880653225	-1.700636688	-1.3766195
PTH1R	-1.895732408	-1.099517227	-1.428068767
JPH2	-1.105075086	-1.700154518	-1.463584584
NEK10	-1.56017973	-1.671864668	-1.47991741
ANKRD20A13P	-1.241396978	-1.364889453	-1.490035214
PCDHB8	-1.600402215	-1.089950894	-1.497526585
ADORA1	-1.54896752	-1.856995921	-1.5390038
SNORD3B-1	-2.629148282	-1.953229817	-1.567944827

ADAM22	-1.628437213	-1.632248538	-1.570584079
FOXD4L1	-1.144233106	-1.175304303	-1.592337701
SLCO1B3-SLCO1B7	-1.138050464	-1.329277698	-1.625284612
ZDHHC8P1	-1.748007864	-1.207069764	-1.634585475
RNF139-AS1	-1.930227179	-1.731206523	-1.664636548
LINC00565	-1.697005952	-1.439705322	-1.667690385
SAMD11	-1.539439017	-1.34934996	-1.695806932
DANT2	-2.404630462	-2.397768565	-1.709046475
TAS2R10	-1.1009939	-1.769989064	-1.737846759
ID3	-1.508722162	-1.53105167	-1.779484388
TLR5	-1.854402458	-1.594861592	-1.780842742
PIWIL2	-1.174713129	-1.329802013	-1.815979102
MIR943	-1.144652128	-1.214942697	-1.858404453
LOC102724908	-2.015494396	-2.901594627	-1.880571083
GPR20	-2.556778327	-1.523373353	-1.956797994
NR1H4	-1.972442817	-1.041375346	-1.960003439
APLNR	-1.43596435	-1.376241009	-1.962934179
OR2B6	-2.745311771	-3.183753272	-1.987170172
MAN1C1	-1.062852049	-1.94042089	-1.995098709
SAPCD1	-1.780724953	-1.858236212	-2.002608507
SV2A	-2.717625731	-1.767096252	-2.004626679
RPSAP11	-1.619117536	-2.186402662	-2.013207864
MIR5001	-2.074159213	-2.007329043	-2.020625291
RPL13AP14	-1.178708509	-1.234421397	-2.117347347
DUX4L14	-1.634878735	-2.695614494	-2.137284674
RPS7P3	-1.512792303	-3.039689259	-2.154702293
LOC105378577	-6.411702121	-2.534679959	-2.196451063
NOTUM	-1.523251748	-1.337208418	-2.216487826
TNNC1	-1.61160163	-1.313236556	-2.264297816
ANKRD2	-2.477755398	-1.85749453	-2.302897494
HOXB-AS3	-1.589265773	-1.744341582	-2.324192285
TGFB2-OT1	-1.557504342	-3.818700595	-2.330395539
EDN2	-1.587047254	-1.80344048	-2.403227628
DUX4L13	-2.603632028	-2.696299362	-2.445496275
RNU7-1	-1.157620726	-2.916587307	-2.558356164
VWA5A	-1.253215411	-2.025997707	-2.568702376
PDLIM1P4	-2.405881414	-1.091433397	-2.603064032
SNORD108	-3.153401872	-2.990054813	-2.659945385
MIR935	-1.345995855	-3	-2.661198087
SNORD118	-2.834474716	-2.4394501	-2.745240852
ID1	-1.925339144	-2.029942802	-2.768633875
MIR12121	-3.357552005	-1.772589504	-2.772589504
HCAR3	-2.714172348	-3.865477195	-2.84213916
TBCAP3	-3.583062198	-7.539890364	-2.868532894

SNORA56	-1.916708914	-1.961085064	-2.869755666
ATOH8	-2.506874845	-2.360253626	-2.884331601
LOC730668	-1.255806552	-1.174777082	-2.929090457
LINC00537	-1.619829628	-1.098601807	-2.936016633
DUX4L11	-1.159126003	-2.719973605	-3.253680447
DUX4L10	-1.159126003	-2.719973605	-3.253680447
CPA4	-2.628476226	-2.62352	-3.301884199
SLC4A8	-1.876766428	-1.571424377	-3.430313441
ID2	-2.160991695	-2.162481362	-3.619587608
ID4	-3.192909101	-3.842342997	-3.975313409
LOC100422094	-1.36347363	-2.574169918	-4.319642449
KPNA2P3	-3.74305512	-3.039342323	-4.374640577
LOC105274304	-1.245574485	-1.149773543	-6.441992582
MIR5187	-6.722285856	-15.55200859	-6.662504628
MIR4761	-7.94677805	-8.093167537	-7.897025015
MIR34A	-3.848014197	-15.44156094	-9.212742251
MIR3940	-1.575827107	-11.4779772	-12.2221383
MIR1178	-2.361837986	-2.414893139	-12.96115916
MIR6510	-17.95158069	-16.95158069	-16.95158069

Supplementary Table S17. Down-regulated genes in H1299 cells treated with TLR2, TLR4, and TLR7

Gene_Symbol	Fold decrease (TLRs vs. vehicle)		
	TLR2	TLR4	TLR7
GCSHP3	-1.391225487	-2.689737966	-1.01293057
TAS2R31	-1.054885988	-1.879826513	-1.014619059
CRACD	-1.464061734	-2.543562526	-1.032660477
RTL8B	-1.925423569	-2.404420396	-1.034574815
CACNA1H	-1.499334902	-2.054427779	-1.044075206
AMOTL1	-1.816593258	-2.201800031	-1.044679427
SORL1	-3.064251297	-1.895568526	-1.046005289
SIX3	-1.407483371	-1.855887211	-1.047645233
CYFIP2	-1.649227441	-1.745243709	-1.052183533
SNORD62B	-2.486824443	-2.965181332	-1.052411343
TMEM170B	-1.25787619	-1.767198232	-1.065940949
ZNF558	-1.958310943	-2.030809137	-1.077417779
RLIMP3	-2.286602764	-2.179540023	-1.079047616
ADARB2	-1.55767471	-1.99332823	-1.079832009
KIAA0825	-1.89594489	-1.821841624	-1.091567576
PNMA2	-1.751852052	-1.967428351	-1.097148923
TLN2	-1.838698266	-1.775742373	-1.098655913
KCND1	-1.751756614	-2.545845021	-1.09950212
NDRG1	-1.397673001	-1.90710394	-1.120367867
KIF4B	-2.252566723	-2.645476114	-1.141101811
SHC3	-1.626493161	-2.349839118	-1.142665203
MSI1	-1.783350624	-1.745955633	-1.150708985
RAB9B	-2.067012236	-2.910713807	-1.155016473
AGGF1P1	-3.981249158	-1.754159847	-1.176707655
TM4SF19	-1.77344018	-2.043421401	-1.197914408
CELSR2	-1.957599893	-2.613150298	-1.198728776
FOXP1	-2.12247306	-1.890889211	-1.21943449
ARFGEF3	-1.828161922	-1.799929654	-1.224001294
SNORD116-1	-4.148485596	-3.389246239	-1.247353146
NAV1	-2.154639815	-2.385190919	-1.254831321
KCNMA1	-2.400095551	-1.961286686	-1.2568443
ONECUT1	-1.23779648	-3.542612288	-1.276279276
LINC01126	-1.022273924	-1.852155146	-1.27835034
MPP4	-1.166229371	-1.827740229	-1.287851296
RPS6KA2	-1.894723467	-1.905418621	-1.29057773
MAP3K15	-1.244748654	-1.994577235	-1.292454848
LAMA3	-1.953504567	-2.454727212	-1.295358344
SNORD116-28	-4.215612952	-2.774220322	-1.30333682
LOC100506178	-1.561981192	-1.793150397	-1.310275345
IL1B	-1.458620326	-3.941297826	-1.312856401
ITGA1	-2.118985414	-1.980348068	-1.323151173

ADARB1	-1.782168672	-1.950965158	-1.324246241
PLCXD2	-2.040700045	-1.805287568	-1.337447506
SH3PXD2A	-1.87273439	-2.290776676	-1.359070705
TUBB4A	-1.316619564	-1.846741822	-1.360786935
MRAS	-1.407229956	-1.911173002	-1.368920723
DKFZP434A062	-1.287392732	-1.805493758	-1.373330023
MAOA	-1.833611843	-2.189337254	-1.400862156
C3orf80	-1.731039427	-1.848970132	-1.405410902
AGPAT4-IT1	-2.75262406	-2.324771863	-1.406537584
SLC2A13	-2.070160167	-2.38828968	-1.406756315
PACERR	-1.308217603	-1.792760864	-1.411066176
SCN9A	-3.191712736	-2.983718864	-1.415647117
TRIM67	-2.141868293	-2.22729478	-1.423714853
PDE8B	-1.681058534	-2.320988484	-1.436259324
EIF2S3B	-2.9633146	-2.331320056	-1.43721028
ZNF471	-2.697622827	-2.390582235	-1.440148015
EHD2	-1.763782546	-2.425338126	-1.443692364
DYSF	-2.07281243	-2.505612942	-1.454788134
PRICKLE1	-1.756828956	-1.74524868	-1.464205455
RETREG1	-1.776324883	-2.01447629	-1.468916513
AFF3	-1.881870261	-2.206494041	-1.471227545
SNORA119	-2.077819241	-4.019180536	-1.476504138
L1CAM	-2.071059675	-2.175364509	-1.507492408
CDH7	-2.336486762	-2.115606084	-1.517492127
FRY	-1.871632472	-2.112890502	-1.522804981
PREX1	-1.732302482	-2.796269105	-1.523508223
SNORD109A	-2.541814325	-3.405519379	-1.542135953
SLC9A2	-2.124163649	-1.729067882	-1.545853794
SEMA6A	-1.041313094	-1.832628518	-1.550568767
SYNPO2	-1.752232004	-2.369372565	-1.552310153
TEX11	-1.415844245	-1.830210934	-1.565070254
RAPGEF5	-1.958911085	-2.336308554	-1.579261744
HTR1B	-2.33914198	-1.738027079	-1.579637167
SHC4	-1.742532116	-1.927724739	-1.598138394
ADAMTS9	-1.134106399	-2.888131031	-1.598138552
LSAMP	-1.951323539	-2.432438875	-1.60106864
ALDH1A3	-2.749692527	-1.783352952	-1.609786852
DLGAP1	-2.282735577	-2.031997272	-1.618061105
AHNAK2	-2.836271091	-3.269468638	-1.642749971
LRRK2	-2.905599937	-4.508183855	-1.645313286
STX1B	-1.569355117	-2.460640201	-1.652686918
ARHGAP28	-2.634546413	-3.13203865	-1.656943015
B4GALNT3	-1.561120769	-1.815304668	-1.673488588
PTK6	-1.285677561	-1.829352151	-1.67502162

ECHDC1	-1.800361064	-1.779469226	-1.691329813
TAS1R3	-2.434973868	-2.621497203	-1.695865061
ABI3BP	-1.296811613	-1.873583474	-1.698963268
MYH15	-1.527562263	-1.928282089	-1.712799131
EPS8L2	-2.421181663	-2.077346362	-1.732307325
EPHA8	-1.427382721	-1.767217158	-1.74175492
PEG10	-2.32735056	-2.345631985	-1.754229583
PLTP	-1.53291367	-1.797261763	-1.768521731
NRAP	-2.315205582	-2.651208546	-1.768588051
CNNM1	-1.766760041	-2.400312498	-1.78630054
BRSK2	-1.813253822	-1.973642824	-1.788412044
PSD	-1.86315721	-1.935995598	-1.793894363
TIMP4	-2.899610601	-2.072276848	-1.841136619
KCNQ5	-3.142507066	-2.475991791	-1.859365478
ZBTB32	-1.340471962	-1.731504174	-1.864548437
PPEF1	-1.931990482	-1.940156231	-1.895935904
OSBPL5	-2.22264328	-2.568149335	-1.897145007
IGF2BP2	-2.166021989	-2.550961734	-1.904505915
COL1A2	-1.988788354	-2.094517546	-1.904957289
THRB	-1.072298948	-2.727117938	-1.941341538
PLEKHH2	-1.640916812	-2.066002388	-1.941474301
S100A16	-1.641259858	-2.446196302	-1.94479289
LINC02535	-2.220557848	-1.90360618	-1.950553699
RAB3C	-2.191973118	-2.338384162	-1.950618419
PDZD4	-2.05776351	-1.884378072	-1.970500082
VGF	-2.093798031	-2.263460754	-1.970652481
MTMR7	-1.51333811	-2.409289018	-1.98962859
THBD	-1.183035551	-2.606260271	-1.996552501
NID2	-2.805963371	-2.95467528	-2.012612261
OSBP2	-2.438707937	-3.02385593	-2.014091114
KLK1	-2.182241756	-2.818357403	-2.017006385
PIK3AP1	-1.695447374	-3.377198388	-2.025220951
BICDL1	-1.928585233	-2.244843881	-2.025400302
AFAP1L2	-1.396096058	-1.96815586	-2.035481562
KCNN4	-1.786402143	-2.444100396	-2.077468917
FAM131C	-2.853361141	-2.510199476	-2.091390173
LOC105370708	-1.6361317	-1.81234344	-2.095155647
HPGDS	-2.201768457	-2.417849116	-2.108975568
SDK1	-2.041040305	-2.069680244	-2.126177527
FAM174B	-2.945277168	-2.302633937	-2.134133095
ALPL	-2.211642291	-2.684343402	-2.149427021
TENM1	-4.675098404	-3.335885879	-2.163593688
LOC102724434	-1.089600883	-2.220866855	-2.165539608
ICA1	-2.258981557	-2.627323076	-2.166217086

IGSF3	-2.348347129	-2.678492527	-2.171211469
CRACR2A	-1.837919023	-3.639071897	-2.190390246
MAGEB2	-1.908208951	-1.981730316	-2.201232718
PARD3B	-1.301946259	-2.084230211	-2.203562366
ABCA3	-2.36321311	-3.168254129	-2.209817862
HNRNPA3P10	-2.580521114	-4.902287261	-2.211364334
NNT-AS1	-2.58695544	-3.008615301	-2.230181385
HHIPL1	-2.978010954	-3.611490916	-2.251566598
C14orf132	-1.486779118	-1.959201945	-2.272522736
LRRC4B	-1.876406614	-2.336031111	-2.274479042
CTSH	-1.9083529	-2.619513292	-2.281396876
PTCHD1	-4.61967473	-3.469542729	-2.284080123
LOC440568	-3.757282372	-2.674936462	-2.284963745
KCNQ2	-1.640355789	-2.380707089	-2.293595857
HYAL1	-2.294324112	-2.473086087	-2.305229189
ANO3	-3.376938224	-3.383437289	-2.319960593
KCNJ11	-1.769285169	-1.969704409	-2.321889806
LINC01537	-2.663971905	-2.011681443	-2.327872259
TENM2	-3.025098528	-2.751363526	-2.375069397
LYPLAL1-DT	-2.760687112	-2.436545988	-2.387908512
LRRK1	-1.4291604	-4.225638879	-2.413546393
DCAF12L2	-1.695212783	-2.663231898	-2.41845655
SPINK6	-2.286378408	-2.301381629	-2.455451751
GPR4	-3.105199929	-3.423704324	-2.456487815
F2R	-2.198358886	-2.591546608	-2.484849221
SLC35F3	-2.370687835	-2.401489555	-2.499597066
LINC00707	-2.34141105	-2.511760263	-2.522494399
CNTN1	-2.860871869	-2.96129013	-2.523813678
MIR639	-2.245113866	-6.580173909	-2.528447818
SPTB	-2.489993354	-5.546529791	-2.547424476
LYPD6	-2.86980901	-3.034007744	-2.580041088
SCN3A	-2.89010623	-2.913239331	-2.583653278
F2RL1	-2.66392003	-2.274147732	-2.609575675
OBSL1	-1.692080951	-1.869187082	-2.614259366
TNFRSF10D	-2.993513345	-3.411904321	-2.637641095
INHBA	-3.953443474	-4.094355215	-2.660685805
LAMB3	-2.726382102	-2.955139373	-2.6709333
FST	-2.023409975	-2.506519622	-2.674459775
NNT	-2.787947614	-3.259193553	-2.690108368
G0S2	-2.187808939	-2.674121223	-2.693043035
CAMK2B	-1.481676515	-1.834140524	-2.724935252
XK	-3.929878085	-3.664453441	-2.738170024
ATP1A3	-3.01650365	-2.848536147	-2.74969935
LARGE1	-3.740134234	-2.48301322	-2.753734256

GLDC	-2.743345707	-2.861623344	-2.754817757
TNXA	-2.662921539	-2.842171285	-2.795573194
NPAS3	-2.903497583	-3.393978985	-2.815016329
CAMSAP3	-1.763076124	-1.917810804	-2.831649663
ARHGEF4	-3.177285005	-3.118322941	-2.839188252
DSP	-2.641423837	-3.901651936	-2.84033399
ZNF831	-4.16215439	-4.094914278	-2.855046236
ARHGAP31	-2.683654317	-3.403977649	-2.895080452
RPE65	-2.360660644	-3.587974523	-2.942058751
CDH11	-3.654564248	-3.615410089	-3.165763427
NRIP3	-2.988694855	-3.178284169	-3.260887025
IGFBP2	-3.153189954	-3.034319897	-3.336443259
LTBP1	-4.633080086	-4.305036362	-3.345263718
RNF212	-2.541905332	-2.176886266	-3.410319386
NHS	-4.578923772	-4.716371171	-3.476585418
SLITRK4	-3.63457096	-3.256286142	-3.528004613
ZNF717	-3.431757496	-3.901418206	-3.605234719
FSTL4	-4.792701469	-4.035426136	-3.613100293
LOC100420326	-2.601704472	-4.263646635	-3.641736762
TNXB	-3.61286873	-2.561096203	-3.650146138
H2AJ	-2.368649122	-3.146201976	-3.708994444
UNC13A	-4.411768718	-4.691138905	-3.719856021
ZNF365	-1.489835184	-2.77184845	-3.751301454
DAZ3	-3.564311466	-3.240611867	-3.98359835
ME3	-2.795974376	-2.266143894	-3.995909034
DAZ4	-3.432681122	-3.347750099	-4.108153155
DAZ1	-3.783326354	-3.176926659	-4.161686361
MYOM3	-5.508626969	-4.980258905	-4.303871502
IQGAP2	-2.865574182	-4.692615827	-4.3414314
SFMBT2	-6.10742406	-5.206117929	-4.360559269
FBXL7	-3.779978483	-2.882102562	-4.367365545
C19orf33	-4.358149938	-3.659653786	-4.430025096
DAZ2	-3.945576313	-3.72082635	-4.817807456
P2RX7	-4.514207747	-4.935520532	-4.843815882
EYS	-4.872744942	-4.228493041	-4.885635813
CD36	-4.503004007	-5.536855085	-5.249959155
SLFN11	-5.117794025	-6.266169588	-5.537786855
SLC38A5	-6.888686591	-6.169136549	-6.749001635
MIR6800	-1.442717595	-3.772499478	-9.864014139

Supplementary Table S18. Genes related to lung cancer progression in A549 cells

Gene_Symbol	Fold increase (TLRs vs. vehicle)		
	TLR2	TLR4	TLR7
NDUFA4L2	3.338800279	3.728079614	2.115470007
ASNS	2.540844374	2.581906163	1.461765621
NUPR1	2.481264874	2.529977966	1.387542595
BCAT1	2.153024313	2.335229414	1.343981952
TDRKH-AS1	2.366850566	2.316328082	1.563330048
PSAT1	2.004954936	1.994283386	1.037021676
TRIB3	2.02901893	1.896375009	1.750801687
ABCG1	1.899072494	1.89231458	1.718546066
KIF21B	2.049119323	1.890770657	1.528099465
RELB	1.683565233	1.768867442	1.075113262
C5AR1	1.559090362	1.594127457	1.025866458
NTSR1	2.103830202	1.132301797	1.883410509

Gene	Reference
NDUFA4L2	Thorac Cancer. 2019 Apr;10(4):676-685.
ASNS	Cancer Gene Ther. 2016 Sep;23(9):287-94.
NUPR1	Anat Rec (Hoboken). 2012 Dec;295(12):2114-21.
BCAT1	Onco Targets Ther. 2020 Apr 29;13:3583-3594.
TDRKH-AS1	Front Oncol. 2020 May 15;10:639.
PSAT1	Oncogene. 2020 Mar;39(12):2509-2522.
TRIB3	Nat Commun. 2020 Jul 21;11(1):3660.
ABCG1	Exp Ther Med. 2017 Jun;13(6):31893194.
KIF21B	Cancer Cell Int. 2020 Jun 12;20:233.
RELB	Cancer Cell Int. 2018 Jun 26;18:88.
C5AR1	Am J Respir Crit Care Med. 2018 May 1;197(9):1164-1176.
NTSR1	Clin Cancer Res. 2010 Sep 1;16(17):4401-10.

Supplementary Table S19. Genes related to lung cancer progression in H1299 cells

Gene_Symbol	Fold increase (TLRs vs. vehicle)		
	TLR2	TLR4	TLR7
ANKRD22	4.216921296	4.036535747	3.400686485
RPPH1	2.795572485	3.862917075	2.880286076
RMRP	2.154526003	3.38534358	4.466412598
IGFBP3	3.309400737	3.018586713	3.053909257
GPC3	2.842094279	2.719327079	3.608783281
LMO1	1.889507521	2.622365951	1.152787902
KCNK15	1.051742395	2.324417405	1.2761545
LUM	1.890351993	2.300765106	2.152593651
EHF	1.10944137	2.208566131	1.949415608
MEST	1.926362031	2.14477938	1.966504792
HPD	1.852162782	2.000595946	1.6705732
MMP2	2.666156458	1.978597356	2.886709139
CYP24A1	2.146321538	1.955205859	2.408775778
HOXC13-AS	1.969460972	1.812472569	1.078909591
FLI1	3.362972373	1.798359949	4.651182429
LIN28B	2.013723949	1.779350497	2.125583171
FOXQ1	1.371388299	1.727948568	1.262754597
SDC2	1.771098103	1.697843072	2.173529695
PLAC8	1.442058202	1.540702256	1.063525701
LINC01503	1.955464137	1.484818964	2.003050059
HKDC1	1.306963354	1.337795297	1.716687089

Gene	Reference
ANKRD22	Sci Rep. 2017 Jun 30;7(1):4430.
RPPH1	Oncol Lett. 2020 Oct;20(4):105.
RMRP	J Cell Biochem. 2019 Sep;120(9):15170-15181.
IGFBP3	Mol Cancer Res. 2017 Jul;15(7):896-904.
GPC3	Biosci Rep. 2019 Jun 25;39(6):BSR20181147.
LMO1	Oncotarget. 2018 Jul 3;9(51):29601-29618.
KCNK15	Oncol Lett. 2019 Dec;18(6):5968-5976.
LUM	Cancers (Basel). 2020 Jan 17;12(1):233.
EHF	Oncol Rep. 2021 Jun;45(6):102.
MEST	J Exp Clin Cancer Res. 2021 Sep 24;40(1):301.
HPD	Cell Death Dis. 2019 Jul 8;10(7):525.
MMP2	BMC Pulm Med. 2020 Oct 28;20(1):283.
CYP24A1	J Thorac Oncol. 2017 Feb;12(2):269-280.
HOXC13-AS	Cell Cycle. 2021 Jan;20(2):236-246.
FLI1	Int J Cancer. 2020 Jul 1;147(1):189-201.
LIN28B	Cell Cycle. 2018;17(11):1372-1380.
FOXQ1	PLoS One. 2012;7(6):e39937.
SDC2	Am J Respir Cell Mol Biol. 2019 Jun;60(6):659-666.

PLAC8	J Immunol Res. 2022 Apr 22;2022:8854196.
LINC01503	Respir Res. 2020 Sep 16;21(1):235.
HKDC1	Cancer Cell Int. 2020 Sep 12;20:450.

Supplementary Table S20. Genes related to anti-lung cancer progression in A549 cells

Gene_Symbol	Fold decrease (TLRs vs. vehicle)		
	TLR2	TLR4	TLR7
MIR1287	-6.58128135	-4.830879616	-1.041820176
ALX4	-1.432713585	-1.424787148	-1.165289722
IRF8	-1.886803758	-2.478136126	-1.340303534
BNC2	-1.301704747	-1.248094099	-1.348977135
NCALD	-1.880653225	-1.700636688	-1.3766195
NEK10	-1.56017973	-1.671864668	-1.47991741
ID3	-1.508722162	-1.53105167	-1.779484388
TNNC1	-1.61160163	-1.313236556	-2.264297816
MIR935	-1.345995855	-3	-2.661198087
MIR34A	-3.848014197	-15.44156094	-9.212742251

Gene	Reference
MIR1287	Biomed Pharmacother. 2019 Apr;112:108743.
ALX4	Int J Cancer. 2014 Mar 15;134(6):1311-22.
IRF8	Cell Cycle. 2019 Dec;18(23):3300-3312.
BNC2	Cancer Cell Int. 2017 Feb 6;17:18.
NCALD	Oncotarget. 2016 May 3;7(18):25558-75.
NEK10	Oncogene. 2020 Jul;39(30):5252-5266.
ID3	Mol Med Rep. 2016 Jul;14(1):313-8.
TNNC1	Mol Cells. 2020 Jul 31;43(7):619-631.
MIR935	Br J Biomed Sci. 2019 Jan;76(1):17-23.
MIR34A	Int J Cancer. 2012 Dec 1;131(11):2668-77.

Supplementary Table S21. Genes related to anti-lung cancer progression in H1299 cells

Gene_Symbol	Fold decrease (TLRs vs. vehicle)		
	TLR2	TLR4	TLR7
SIX3	-1.407483371	-1.855887211	-1.047645233
FOXP1	-2.12247306	-1.890889211	-1.21943449
LAMA3	-1.953504567	-2.454727212	-1.295358344
ADARB1	-1.782168672	-1.950965158	-1.324246241
SEMA6A	-1.041313094	-1.832628518	-1.550568767
LSAMP	-1.951323539	-2.432438875	-1.60106864
NID2	-2.805963371	-2.95467528	-2.012612261
LINC01537	-2.663971905	-2.011681443	-2.327872259
FST	-2.023409975	-2.506519622	-2.674459775
CAMSAP3	-1.763076124	-1.917810804	-2.831649663
DSP	-2.641423837	-3.901651936	-2.84033399

Gene	Reference
SIX3	PLoS One. 2013 Aug 16;8(8):e71816.
FOXP1	Cancer Biol Ther. 2019;20(4):537-545.
LAMA3	Mol Ther Nucleic Acids. 2019 Dec 6;18:166-182.
ADARB1	PLoS One. 2019 Sep 6;14(9):e0222298.
SEMA6A	Sci Rep. 2019 Sep 16;9(1):13302.
LSAMP	J Pers Med. 2021 Jun 20;11(6):578.
NID2	Pathol Oncol Res. 2020 Apr;26(2):801-811.
LINC01537	Int J Mol Sci. 2019 Aug 1;20(15):3713.
FST	Clin Cancer Res. 2008 Feb 1;14(3):660-7.
CAMSAP3	Cell Sci. 2018 Oct 29;131(21):jcs216168.
DSP	Carcinogenesis. 2012 Oct;33(10):1863-70.

Supplementary Table S22. Genes related to autophagy regulation

Gene_Symbol	Fold increase (TLRs vs. vehicle)		
	TLR2	TLR4	TLR7
ASNS	2.540844374	2.581906163	1.461765621
NUPR1	2.481264874	2.529977966	1.387542595
BCAT1	2.153024313	2.335229414	1.343981952
PSAT1	2.004954936	1.994283386	1.037021676
TRIB3	2.02901893	1.896375009	1.750801687
RELB	1.683565233	1.768867442	1.075113262
NTSR1	2.103830202	1.132301797	1.883410509
ANKRD22	4.216921296	4.036535747	3.400686485
RMRP	2.154526003	3.38534358	4.466412598
IGFBP3	3.309400737	3.018586713	3.053909257
GPC3	2.842094279	2.719327079	3.608783281
MMP2	2.666156458	1.978597356	2.886709139
PLAC8	1.442058202	1.540702256	1.063525701

Gene	Reference
**ASNS**	Autophagy. 2018;14(9):1481-1498
**NUPR1**	Autophagy. 2018;14(4):654-670
**BCAT1**	Cell Death Dis. 2021;12(2):169
**PSAT1**	Autophagy. 2020;16(1):106-122
**TRIB3**	J Biol Chem. 2017;292(7):2571-2585
**RELB**	Cancer Cell Int. 2014;14:67
**NTSR1**	Br J Cancer. 2017;116(12):1572-1584
**ANKRD22**	Theranostics. 2020;10(2):516-536
**RMRP**	Biosci Rep. 2019;39(6):BSR20181367
**IGFBP3**	Oncogene. 2013;32(19):2412-2420
**GPC3**	Onco Targets Ther. 2018;11:193-200
**MMP2**	Biochem Biophys Res Commun. 2015;466(3):376-380
**PLAC8**	J Cell Mol Med. 2020;24(14):7778-7788
